# Regioselective Fluoroalkylarylation of Enamides Enabled by an Iron-Catalyzed Multicomponent Radical Cross-Coupling Strategy

**DOI:** 10.1021/acs.orglett.3c03059

**Published:** 2023-09-29

**Authors:** Ángel Rentería-Gómez, Macayla Guerrero, Mireya Ramirez-Lopez, Osvaldo Gutierrez

**Affiliations:** Department of Chemistry, Texas A&M University, College Station, Texas 77843, United States

## Abstract

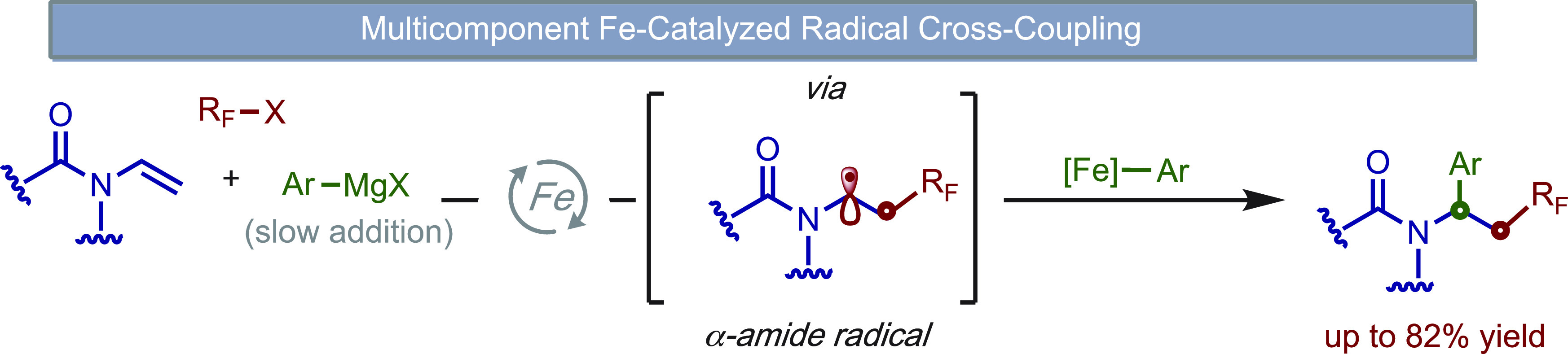

Fluoroalkylated compounds are important entities in agrochemicals, pharmaceuticals, and materials. The catalytic dicarbofunctionalization of alkenes represents a powerful strategy for the rapid construction and diversification of compounds. In this vein, multicomponent cross-coupling reactions (MC-CCR) can provide an efficient synthetic route to build molecular complexity. In this work, we report the first iron-catalyzed three-component fluoroalkylarylation of enamides via selective formation and trapping of α-amide radicals under mild conditions and fast reaction times. The reaction tolerates a variety of commercially available aryl Grignard reagents and fluoroalkyl halides. Finally, the use of a removable phthalimido group provides an efficient strategy to prepare highly valuable γ-difluoroalkylated amines.

Difunctionalization of alkenes represents a powerful synthetic strategy to build molecular complexity.^1^ In the past decade, the use of alkenes in transition-metal-catalyzed multicomponent cross-coupling reactions has emerged as an efficient and modular method to access diverse molecular structures, including fluoroalkylated compounds, through the simultaneous construction of two new bonds across the π system.^2^ Fluoroalkylated compounds are appealing due to the properties of the difluoromethylene group (−CF_2_−) and derivatives that can, inter alia, induce conformation changes, increase dipole moments, and modulate the acidity of neighboring C–H bonds.^3^ Notably, some perfluoroalkyl-substituted compounds have shown more exceptional properties than the CF_3_ or CF_2_H substituents.^4,5^ Recently, Zhang^6a−6c^ and Nevado^6d^ reported the use of enamides as effective lynchpins in nickel-catalyzed three-component cross-coupling reactions (Scheme 1A). However, a major drawback of these methods is the need for long reaction times, high temperatures, and/or limited substrate scope. Further, despite the importance of *N*-alkylated phthalimides and, in particular, widespread application in the practical synthesis of amines,^7,8^ the use of *N*-vinylphthalimide in these transition-metal-catalyzed cross-coupling reactions has been severely limited.^6e,6f^

**Scheme 1:**
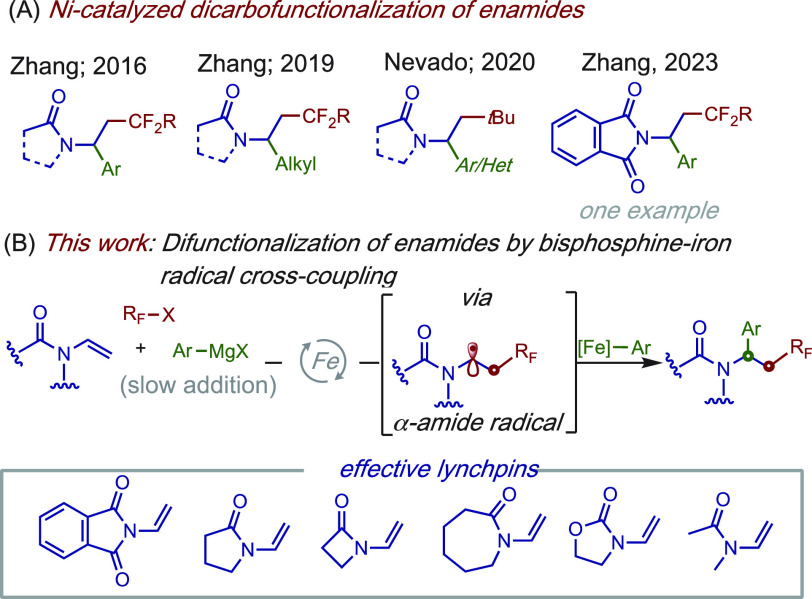
Transition Metal-Catalyzed Dicarbofunctionalization of Enamides

Iron is an attractive transition metal catalyst in cross-coupling reactions due to its low cost, abundance within the Earth’s crust, and lower toxicity in comparison to traditional palladium and nickel systems.^9^ Previously, our group reported the use of iron catalysts to promote three-component cross-coupling reactions with α-heteroatom alkyl radicals including α-boryl and α-alkoxy radicals.^9^ Inspired by this work and recent works using nickel catalysts by the Zhang and Nevado groups (Scheme 1A; inset),^6a−6c^ we hypothesized that bisphosphine–iron complexes could serve as effective catalysts to promote fluoroalkylarylation of enamides via selective formation and cross-coupling of α-amide radicals (Scheme 1B). If successful, such an approach would provide a complementary approach to nickel-based systems and expand the utility of iron catalysts in multicomponent cross-couplings. Herein, we describe the first examples of an iron-catalyzed three-component cross-coupling of *N*-vinyl π-systems including *N*-vinylphthalimides, *N*-vinyl lactams, *N*-vinyloxazolidinone, and *N*-vinylacetamides with readily available fluoroalkyl bromides and aryl Grignard reagents.

To evaluate the feasibility of our designed multicomponent strategy, we selected *N*-vinylphthalimide (**1a**), 2-(2-bromo-1,1,2,2-tetrafluoroethoxy) anisole (**2a**), and 3-methoxyphenylmagnesium bromide (**3a**) as the model substrates (Table 1). Pleasingly, using dcpe and FeCl_3_, we were able to observe the formation of the desired product in good yield (60%) (entry 1). Remarkably, the alcohol product of the nucleophilic addition of **3a** over **1a** was observed only in very low yield (∼10%). In addition, we screened different iron sources (entries 2–3) and found that FeCl_3_ gives the best yield for the reaction.

**Table 1:**
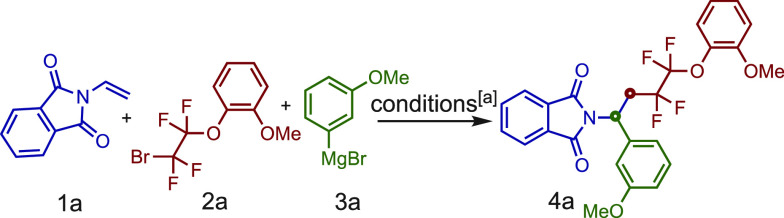

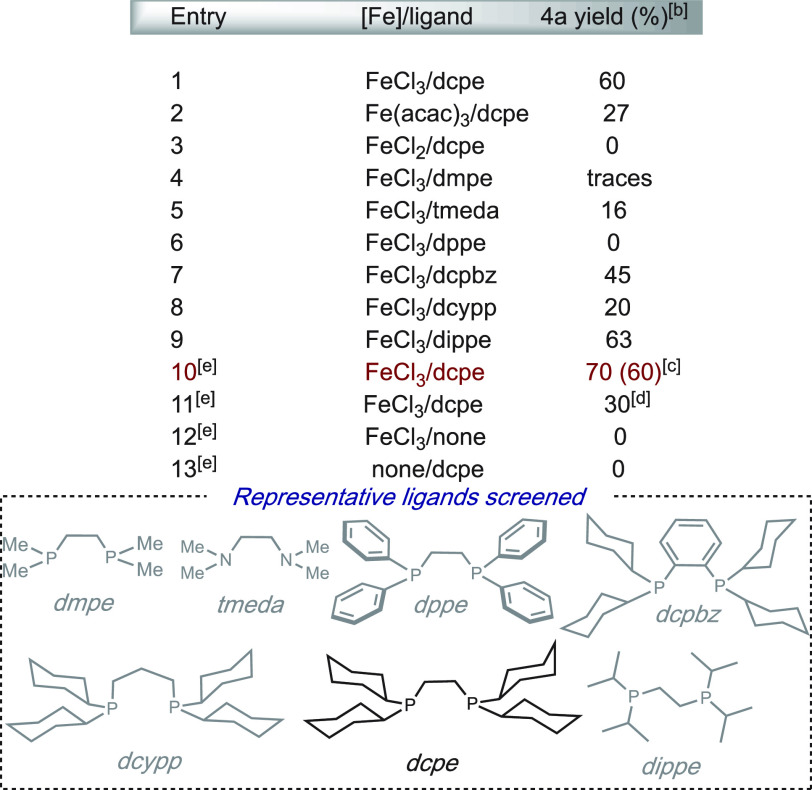
Optimization of Reaction Conditions.^a^

a Reaction conditions: **1a** (0.2 mmol, 1.0 equiv), **2a** (0.4 mmol, 2.0 equiv), Fe catalyst (10 mol %), ligand (20 mol %), THF (*c* 1.0 M), 0 °C, slow addition of **3a** (0.6 mmol, 3.0 equiv) in 1 h, nitrogen atmosphere.

b Determined by ^1^H NMR using 1,2-dibromomethane as an internal standard.

c Yield (%) of isolated product.

d FeCl_3_ (5 mol %), dcpe (10 mol %).

e 0.8 mmol of **3a** was used.

Finally, to assess the ligand effect on this MC-CCR, we screened several commercially available bidentate ligands (entries 4–9). Notably, only 1,2-bis(diisopropylphosphino)ethane (dippe) also led to a high yield (63%, entry 9) as with the dcpe ligand. Notably, we observed that increasing the loading of Grignard reagent (added via a syringe pump over the course of 1 h) improved the overall yield (up to 70%) (entry 10). Finally, the reaction proceeded smoothly even with only 5 mol % of FeCl_3_ albeit with reduced yield (entry 11). Additional control experiments (entries 12–13) confirmed that both iron salt and ligand are essential to promote the MC-CCR. In this context, we attribute the reactivity to form **4a** to a unique bisphosphine–iron catalytic system that under slow addition of Grignard reagent generates the active monoaryl and bisaryl bisphosphine iron(II) species that are responsible for C–C bond formation and radical generation, respectively.^9^ Overall, the optimized conditions consisted of performing the reaction in THF at 0 °C in the presence of FeCl_3_ (10 mol %) and using dcpe (20 mol %). Under these optimized conditions (see Supporting Information for full screening details), the three-component adduct **4a** was isolated in 60% yield (dcpe).

With the optimized conditions in hand, the scope of this novel three-component reaction was examined. Overall, a wide range of aryl Grignard reagents were compatible in this one-pot procedure to form two carbon–carbon bonds with the fluoroalkyl bromide compound **2a** and the *N*-vinylphthalimide **1a** (Scheme 2). For example, *meta*-substituted electron-rich (**4a** and **4h**) and electron-poor (**4f**, **4i**, and **4j**) aryl Grignards gave the desired MC-CCR products in good yields (up to 60%). In addition, this method tolerates a range of electron-rich and -poor *para*-substituents including -Cl (**4b**), -OCF_3_ (**4c**) -Me (**4g**), and -SMe (**4l**). Notably, we found that lower loadings of aryl Grignards for some systems (**4e**, **4l**, and **4m**) improve the over yield. Also, extended π-systems work well in this transformation (**4n**). Although *meta*- and *para*-substituted Grignard reagents alike afforded products, *ortho*-substituted Grignard reagents were not compatible (**4o**), presumably due to steric effects. Unfortunately, despite numerous attempts, we found that heteroaryl Grignard reagents were not compatible in this transformation.

**Scheme 2:**
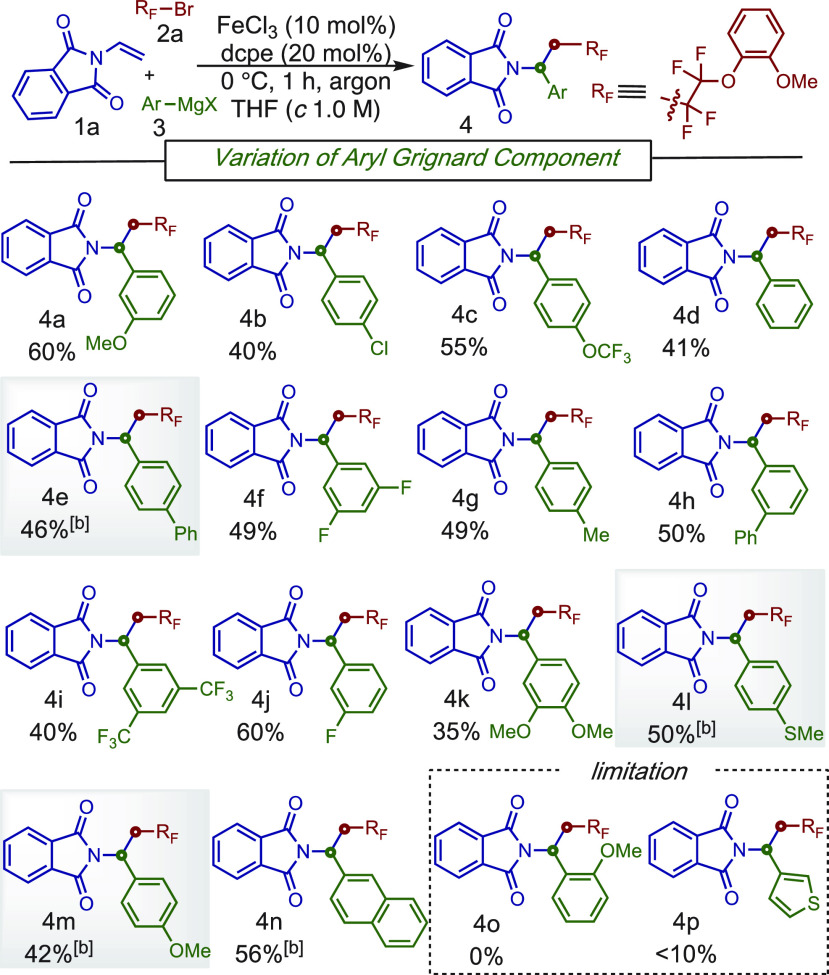
Scope of the Aryl Grignard Reagents for the Three-Component Reaction Conditions: **1a** (0.2 mmol), **2a** (0.4 mmol), **3** (0.8 mmol), FeCl_3_(10 mol %), dcpe (20 mol %), THF (*c* 1.0 M), 0 °C, slow addition of **3** in 1 h, argon atmosphere. 0.4 mmol of **3** was used.

Having investigated the aryl Grignard scope, we then turned our attention to exploring the scope of fluoroalkyl halides as radical precursors in this MC-CCR (Scheme 3). Overall, a broad range of di-, tetra-, and perfluoro alkyl bromides as radical precursors bearing various functionalities including fluoroalkyl-rich (**5a** and **5b**) chains, extended alkyls (**5c** and **5d**), aryl (**5e**), aryl ethers with relevant functionalities (**5f**–**h**), heteroaryl (**5i**), diethoxyalkyl as protected aldehydes (**5j**), aryl (thio)ethers (**5l**), and olefins (**5m**) were compatible in this transformation. Notably, an α,α-difluoro ester bromide engages in the MC–CCR to obtain **5k** in acceptable yield (40%). Furthermore, the resulting β-difluoroalkylated compound **5k** could be used in the synthesis of fluorinated lactams,^6a^ an interesting motif for drug discovery.^10^ As expected, alkyl radicals (e.g., tertiary)that are hesitant to undergo two-component cross-coupling reactions with Grignard reagents under iron catalysis, were also compatible in this three-component transformation (**5n**), but a high amount of the nucleophilic addition to the C=O bond was also observed (22%). Unfortunately, difluoroalkyl amides failed to give the corresponding products (**5o** and **5p**).

**Scheme 3:**
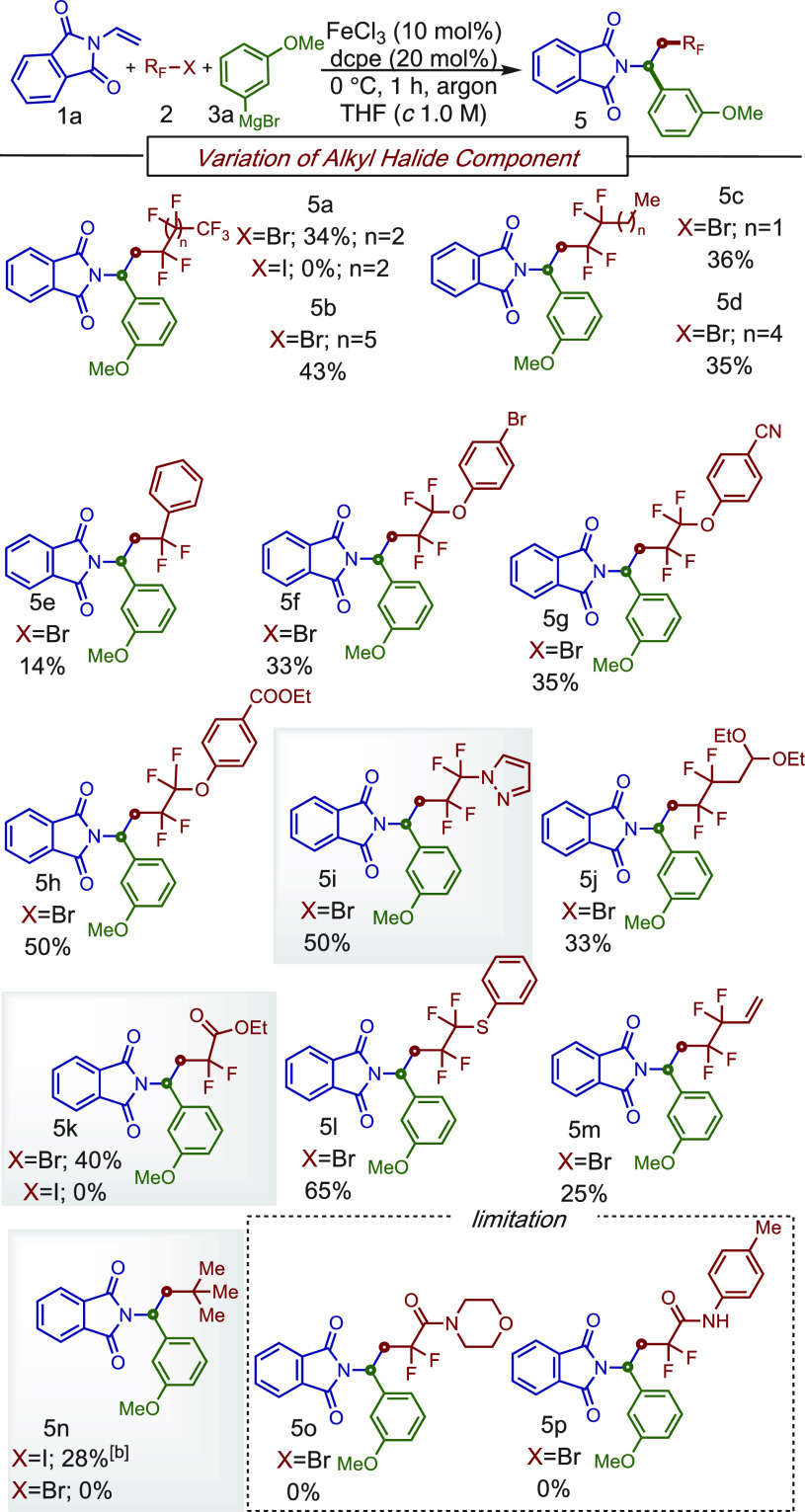
Scope of Fluoroalkyl Halides for a Three-Component Reaction Conditions: **1a** (0.2 mmol), **2** (0.4 mmol), **3a** (0.8 mmol), FeCl_3_ (10 mol %), dcpe (20 mol %), THF (*c* 1.0 M), 0 °C, slow addition of **3** in 1 h, argon atmosphere. 0.4 mmol of **3a** was used.

Next, due to the importance of the lactams,^11^ the scope of this MC-CCR was examined (Scheme 4). Overall, a wide range of different sizes of *N*-vinyl lactams were compatible in this one-pot procedure including γ-lactams (**6a**–**e**), β-lactams (**6g**), and ε-lactams (**6h**). Remarkably, compound **6f** was formed with a modest diastereomeric ratio (3:2) using the corresponding chiral *N*-alkenylpyrrolidinone. Remarkably, the *N*-vinyloxazolidinone and *N*-methyl-*N*-vinylacetamide were also applicable to the reaction (**6i** and **6j**), thus demonstrating complementary reactivity of this iron-catalyzed MC-CCR in comparison to nickel-based systems. Notably, at larger scale (1.0 mmol), the reaction proceeded smoothly, forming the desired product **6h** in 67% isolated yield.

**Scheme 4:**
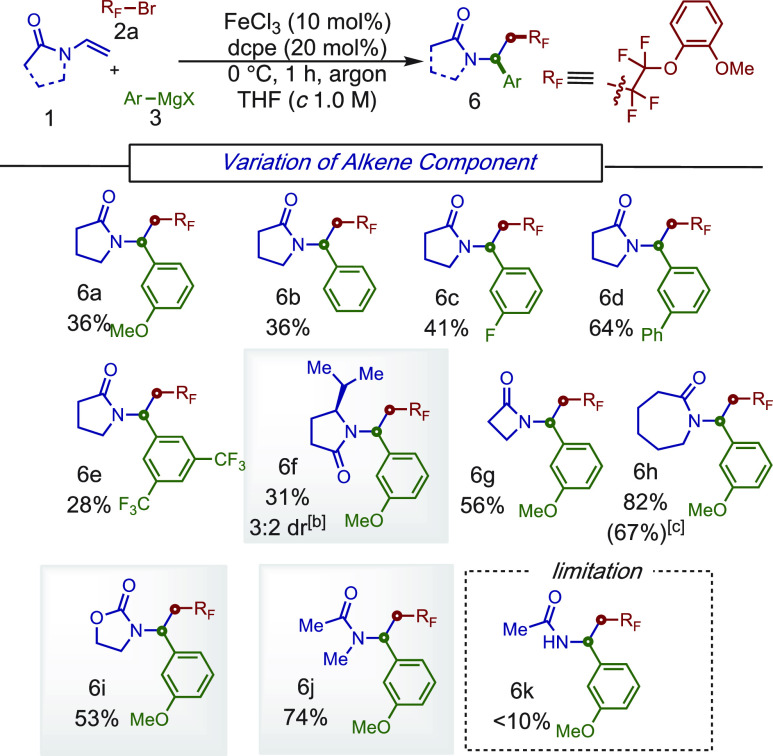
Scope of the *N*-Vinyl Amide Component for the Three-Component Reaction Conditions: **1a** (0.2 mmol), **2** (0.4 mmol), **3** (0.8 mmol), FeCl_3_(10 mol %), dcpe (20 mol %), THF (*c* 1.0 M), 0 °C, slow addition of **3** in 1 h, argon atmosphere and 0.4 mmol of **3** was used. Diastereomeric ratio (dr) was determined by crude ^1^H NMR. Reaction was performed on a 1 mmol scale.

Also, the value of this methodology for access to complex amines is supported by the deprotecting phthalimido group. In this regard, another important structural motif seen in medicinal chemistry are γ-difluoroalkylated amines, but methods to access these valuable structures are sparse.^12^ As shown in Scheme 5, benzyl amine **8** was obtained from **4a** in 98% yield using the Ing-Manske procedure,^13^ offering potential opportunities for applications in the synthesis of highly valuable γ-difluoroalkylated amines and fluorinated amino acids.^14^

**Scheme 5:**
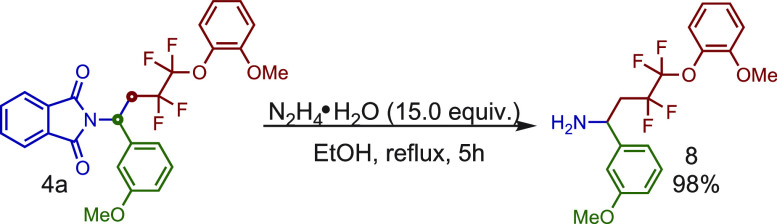
Deprotection of the Phthaloyl Group

Finally, to establish the formation of the **Int-1** radical in this transformation, we used tetrafluoroalkyl halide **2b** with a pendent alkene as a “radical trap” (Scheme 6A). Notably, under our optimized reaction conditions, the use of this bifunctional reagent led to the formation of cyclic compound **7** in moderate yield with 1:3.5 dr. The major diastereomer was confirmed by X-ray diffraction analysis as *cis*-**7**. Based on this result and prior mechanistic studies,^9,15^ a proposed mechanism is shown in Scheme 6B. Formation of Fe(I) **A** can undergo halogen-atom abstraction to form the radical **2**^●^ and Fe(II) **B** species. Then **2**^●^ can escape the solvent cage to undergo radical addition to *N*-vinyl compound **1** to form the **Int**^●^ with the concomitant formation of Fe(II) **B**. In parallel, slow addition of Grignard reagent can promote selective monotransmetalation of **B** to form **C**.^9c,15^^12^ Finally, **Int**^●^ will then undergo selective and reversible radical addition to monoaryl Fe(II) **C** to form Fe(III) **D**. Finally, reductive elimination from **D** will yield the desired multicomponent product **4** and Fe(I) species **A** that can then restart the catalytic cycle.

**Scheme 6:**
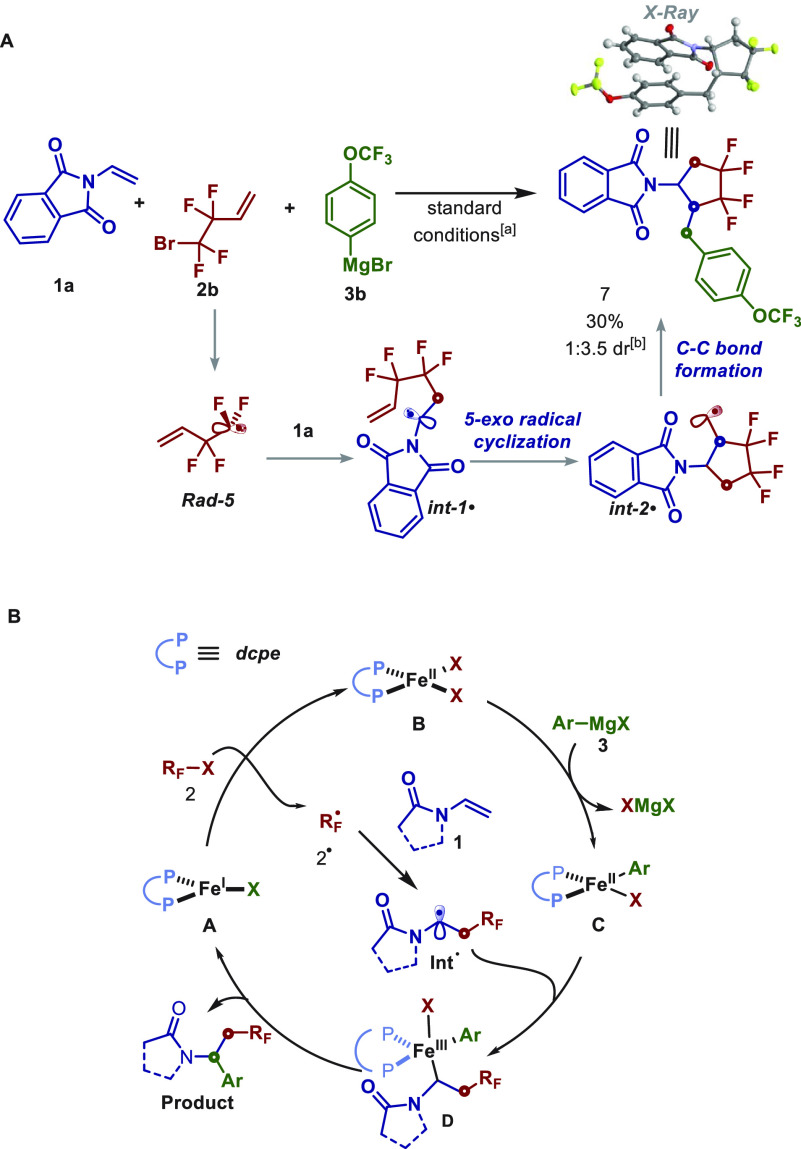
(A) Radical Cascade Annulation and (B) Proposed Catalytic Cycle Conditions: **1a** (0.2 mmol), **2b** (0.4 mmol), **3**b (0.8 mmol), FeCl_3_(10 mol %), dcpe (20 mol %), THF (*c* 1.0 M), 0 °C, slow addition of **3b** in 1 h, argon atmosphere. 0.4 mmol of **3b** was used. Diastereomeric ratio (dr) was determined by crude ^1^H NMR.

In summary, we have developed an iron-catalyzed three-component reaction that utilizes *N*-vinylphthalimide as a lynchpin.^16^ The reaction tolerates a variety of aryl Grignard reagents and fluoroalkyl bromides. The use of a removable phthalimido group can provide an efficient strategy to prepare highly valuable γ-difluoroalkylated amines. The reaction can also extend to different ring sizes of *N*-vinyl lactams, *N*-vinyloxazolidinone, and *N*-vinylacetamide. Further studies to develop catalytic asymmetric synthesis are in progress in our lab and will be reported in due course.

## Data Availability

The data underlying this study are available in the published article and its Supporting Information.

## Supporting Information Available

Experimental procedures, product characterizations, crystallographic data, and copies of ^1^H and ^13^C NMR spectra (PDF)
