# Supplementary material for: Regioselective Fluoroalkylarylation of Enamides Enabled by an Iron-Catalyzed Multicomponent Radical Cross-Coupling Strategy
Source: Org Lett. 2023 Sep 29;25(40):7440–5. doi: 10.1021/acs.orglett.3c03059 (PMC10863403; doi:10.1021/acs.orglett.3c03059)
Supplement: Supplementary file 1 — ol3c03059_si_001.pdf [file ol3c03059_si_001.pdf]

Regioselective Fluoroalkylarylation of Enamides Enabled by an Iron-Catalyzed Multicomponent Radical Cross-Coupling Strategy

Ángel Rentería-Gómez, Macayla Guerrero, Mireya Ramirez-Lopez, Osvaldo Gutierrez*

Table of Contents

1. General Considerations S3
2. General Procedure for preparation of *N*-vinylamides S3
3. General Procedure for Iron-Catalyzed Multicomponent Cross-Coupling Reaction S3
4. Optimization of Reaction Conditions S4
5. Product Characterization Data S4-S21
6. Procedure for the deprotection of 4a S21
7. Spectral Data S22-S105
8. Crystallographic Data S106-S113
9. Current Limitations S114
10. References

1. General Considerations.

Unless otherwise indicated, all reactions were carried out under a nitrogen atmosphere in oven- (110 °C) or flame-dried glassware. When necessary, solvents and reagents were dried prior to use. Tetrahydrofuran (THF) was dried by passage through activated alumina in Inert's PureSolv MB-SPS solvent purification system. All solvents were obtained from VWR, Sigma-Aldrich, or Fisher. Organometallic reagents were purchased from Sigma-Aldrich and Synthonix. Silicycle 250 µm silica-gel F-254 plates were used to perform analytical thin layer chromatography (TLC). Column chromatography was performed with Silica gel (230-400 mesh) and Biotage® Selekt Flash Systems silica gel chromatography was performed with prepacked silica-gel cartridges (Sfar; Biotage). NMR (^{1}H, ^{13}C and ^{19}F) spectra were recorded on Acsend TM 400 (Bruker) NMR spectrometer. Chemical shifts (δ) are reported in parts per million (ppm) relative to the internal residual solvent resonance peak δ 7.26 ($CDCl_3$) and δ 0.00 (TMS) for all ^{1}H and δ 77.16 ($CDCl_3$) and δ 0.00 (TMS) for all ^{13}C. Other data are designated as follows: multiplicity (s = singlet, d = doublet, t = triplet, q = quartet, qu = quintet, sept = septet, oct = octet, m = multiplet, dd= doublet of doublets, dt = doublet of triplets, td = triplet of doublets, dq = doublet of quartets, qd = quartet of doublets, tt = triplet of triplets, tdd = triplet of doublet of doublets, bs = broad singlet), coupling constants (*J*) are reported in Hertz (Hz), and number of protons. Electrospray ionization (ESI) and atmospheric chemical ionization (APCI) mass spectrometry experiments were performed using a Thermo Scientific Q Exactive Focus. Sample was loop injected (10 µL) and methanol was used as a mobile solvent at a flow rate of 600 µL/min. The Q Exactive Focus HESI source was operated in full MS in positive mode. The mass resolution was tuned to 70000 FWHM at m/z 200. The electrospray spray voltage was set to 3.5 kV and for APCI the discharge current was set at 5 µA. The sheath gas and auxiliary gas flow rates were set to 40 and 10 arbitrary units, respectively. The transfer capillary temperature was held at 320 °C and the S-Lens RF level was set at 50 v. Exactive Series 2.11 /Xcalibur 4.2.47 software was used for data acquisition and processing. Thermo Scientific Nicolet Summit FTIR spectrometer was employed for recording IR spectra and are reported in wavenumbers (cm-1). Melting points were obtained and are uncorrected. A BRUKER Venture X-ray (kappa geometry) diffractometer was used for crystal screening, unit cell determination, and data collection. Main Text Paragraph.

2. General Procedure A for preparation of N-vinylamides.[1]

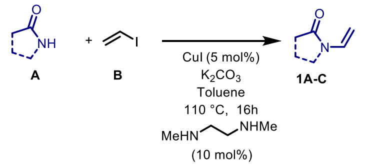

A mixture of amide **A** (3.9 mmol, 1.0 equiv), vinyl iodide (7.9 mmol, 2.0 equiv), *N,N'*-dimethylethylenediamine (0.4 mmol, 0.1 equiv), K_2CO_3 (2.2 mmol, 0.55 equiv), CuI (0.20 mmol, 0.05 equiv), and toluene (1.2 mL) was placed in a tightly closed sealed tube fitted with a magnetic stir bar. The mixture was stirred for 16 hours at 110°C in an oil bath, then cooled to room temperature. The mixture was stirred for 16 hours at 110°C in an oil bath, then cooled to room temperature. The tube was carefully opened, and its contents were filtered through celite, the solid residue was rinsed with CH_2Cl_2, and the combined filtrates were evaporated and purified on silica gel column chromatography with hexane/ethyl acetate to yield the vinylamides **1A-C**.

3. General Procedure 1 for Iron-Catalyzed Multicomponent Cross-Coupling Reaction.

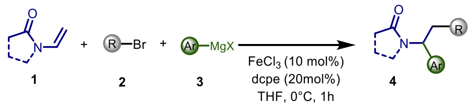

Standard-scale: A flame-dried 5 mL microwave vial with a stir bar was transferred into an argon-filled glovebox and the vial was charged with $FeCl_3$ (3.2 mg, 0.02 mmol), 1,2-bis(dicyclohexylphosphanyl)ethane (dcpe) (16.9 mg, 0.04 mmol), alkyl halide 2 (0.4 mmol, 2.0 equiv) and amide 1 (0.2 mmol, 1.0 equiv) (using oven-dried glass pipette to transfer alkyl halide to the vial). The vial was sealed with a Teflon cap and brought out of the glovebox without solvent. THF (0.2 mL) was then added into the reaction mixture. The resulting green solution was stirred at room temperature for 5 min. The reaction mixture was then cooled to 0 °C and a ArMgBr 3 (0.5–1.0 M solution in THF, 2.0 or 4.0 equiv) was added slowly for 1 h using a syringe pump. Over which time the heterogeneous solution turned from red to yellow, brown, grass green or orange color (depending on ArMgBr and substrate). The resulting reaction mixture was then stirred at 0 °C for an additional 10 min. After completion, reaction mixture was quenched with a 1.0 M aqueous solution of hydrochloric acid (0.5 mL) and water (0.5 mL) and then extracted with ethyl acetate (3 x 2 mL). The combined organic solution was filtered through a short pad of silica and evaporation of solvent gave a residue that was purified on silica gel column chromatography with hexane/ethyl acetate to obtain product.

4. Optimization of Reaction Conditions.[a]

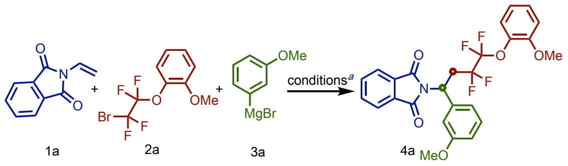

Entry	1a/2a/3a (equiv)	[Fe]/lignad	4a yield (%)[b]
1	1.0/2.0/2.0	$FeCl_3$/dcpe	55
2	2.0/1.0/2.0	$FeCl_3$/dcpe	12
3	1.0/3.0/2.0	$FeCl_3$/dcpe	56
4	1.0/2.0/3.0	$FeCl_3$/dcpe	60
5	1.0/2.0/3.0	$FeBr_3$/dcpe	49
6	1.0/2.0/3.0	$Fe(acac)_3$/dcpe	27
7	1.0/2.0/3.0	$Fe(OTf)_3$/dcpe	39
8	1.0/2.0/3.0	$Fe(dibm)_3$/dcpe	44
9	1.0/2.0/3.0	$FeCl_2$/dcpe	0
10	1.0/2.0/3.0	$FeCl_3$/dmpe	traces
11	1.0/2.0/3.0	$FeCl_3$/tmeda	16
12	1.0/2.0/3.0	$FeCl_3$/dppe	0
13	1.0/2.0/3.0	$FeCl_3$/dcpbz	45
14	1.0/2.0/3.0	$FeCl_3$/dcypp	20
15	1.0/2.0/3.0	$FeCl_3$/dippe	63
16	1.0/2.0/4.0	$FeCl_3$/dcpe	70 (60)[c]
17	1.0/2.0/4.0	$FeCl_3$/dcpe	30[d]
18	1.0/2.0/4.0	$FeCl_3$/none	0
19	1.0/2.0/4.0	none/dcpe	0

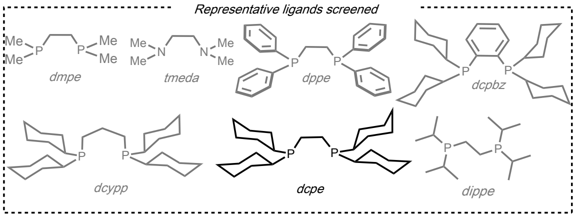

[a]Reaction conditions: Fe catalyst (10 mol %), ligand (20 mol %), THF (*c* 1.0 M), 0 °C, slow addition of **3a** in 1 h, nitrogen atmosphere. [b] Determined by ^{1}H NMR using 1,2-dibromomethane as an internal standard. [c] Yield (%) of isolated product. [d] $FeCl_3$ (5 mol %), dcpe (10 mol %).

5. Product Characterization Data.

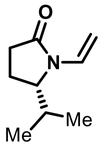

(*S*)-5-isopropyl-1-vinylpyrrolidin-2-one (1A): Compound **1A** was synthesized following the general procedure A, using (*S*)-5-isopropylpyrrolidin-2-one (500 mg, 3.9 mmol), vinyl iodide (0.6 mL, 7.9 mmol), *N,N'*-dimethylethylenediamine (43 mL, 0.4 mmol), K_2CO_3 (299 mg, 2.2 mmol), CuI (38 mg, 0.20 mmol), and toluene (1.2 mL). The product **1A** was obtained as a colorless liquid (204.0 mg, 34% yield) after purification by column chromatography on silica gel with hexane/EtOAc (8:2).
^{1}H NMR (400 MHz, $CDCl_3$) δ = 6.96 (dd, *J* = 16.3, 9.4 Hz, 1H), 4.50 – 4.42 (m, 2H), 3.93 – 3.87 (m, 1H), 2.50 – 2.33 (m, 3H), 2.03 – 1.89 (m, 2H), 0.94 (d, *J* = 7.0 Hz, 3H), 0.74 (d, *J* = 6.9 Hz, 3H);
^{13}C NMR (100 MHz, $CDCl_3$) δ = 173.8, 128.4, 95.4, 61.0, 31.2, 27.3, 19.0, 18.0, 14.7;
IR (film) 2960.77, 1675.23, 1655.62, 1631.10, 1469.49, 1404.32, 1388.98, 1323.93, 1294.70, 1277.52, 1245.03, 1228.39, 1187.61, 1154.78, 1099.98, 954.95, 670.97 cm^{-1};
HRMS (ESI+) calcd for $C_9H_{16}NO$ $[M+H]^+$ m/z = 154.1226; found: 154.1225.

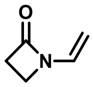

1-vinylazetidin-2-one (1B): Compound **1B** was synthesized following the general procedure A, using azetidin-2-one (277 mg, 3.9 mmol), vinyl iodide (0.6 mL, 7.9 mmol), *N,N'*-dimethylethylenediamine (43 mL, 0.4 mmol), K_2CO_3 (299 mg, 2.2 mmol), CuI (38 mg, 0.20 mmol), and toluene (1.2 mL). The product **1B** was obtained as a colorless liquid (113.6 mg, 30% yield) after purification by column chromatography on silica gel with hexane/EtOAc (8:2).
The spectroscopic data for this compound were identical to those reported in the literature.[2]
^{1}H NMR (400 MHz, $CDCl_3$) δ = 6.67 (dd, *J* = 15.8, 8.9 Hz, 1H), 4.39 (d, *J* = 15.7 Hz, 1H), 4.32 (d, *J* = 8.9 Hz, 1H), 3.35 (t, *J* = 4.5 Hz, 2H), 2.95 (t, *J* = 4.5 Hz, 2H);

^{13}C NMR (100 MHz, CDCl$_3$) δ = 164.3, 127.6, 93.7, 37.8, 36.1;

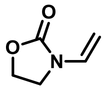

3-vinyloxazolidin-2-one (1C): Compound **1C** was synthesized following the general procedure A, using oxazolidin-2-one (340 mg, 3.9 mmol), vinyl iodide (0.6 mL, 7.9 mmol), *N,N'*-dimethylethylenediamine (43 mL, 0.4 mmol), K_2CO_3 (299 mg, 2.2 mmol), CuI (38 mg, 0.20 mmol), and toluene (1.2 mL). The product **1C** was obtained as a colorless liquid (551.4 mg, 80% yield) after purification by column chromatography on silica gel with hexane/EtOAc (8:2).
The spectroscopic data for this compound were identical to those reported in the literature.[2]
^{1}H NMR (400 MHz, CDCl$_3$) δ = 6.85 (dd, *J* = 15.8, 9.0 Hz, 1H), 4.46 – 4.40 (m, 3H), 4.28 (dd, *J* = 15.8, 0.8 Hz, 1H), 3.72 – 3.67 (m, 2H);
^{13}C NMR (100 MHz, CDCl$_3$) δ = 155.4, 129.8, 93.5, 62.2, 41.9;

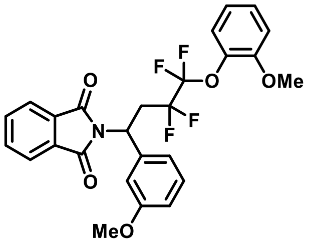

2-(3,3,4,4-tetrafluoro-4-(2-methoxyphenoxy)-1-(3-methoxyphenyl)butyl)isoindoline-1,3-dione (4a): Compound **4a** was synthesized following the general procedure 1 (standard-scale), using 1-(2-bromo-1,1,2,2-tetrafluoroethoxy)-2-methoxybenzene (120.4 mg, 0.4 mmol), *N*-Vinylphthalimide (34.6 mg, 0.2 mmol) and 3-methoxyphenylmagnesium bromide (0.8 mL, 1.0 M solution in THF, 0.8 mmol). The product **4a** was obtained as a colorless liquid (60.4 mg, 60% yield) after purification by column chromatography on silica gel with hexane/EtOAc (8:2).
^{1}H NMR (400 MHz, CDCl$_3$) δ = 7.85 – 7.79 (m, 2H), 7.72 – 7.66 (m, 2H), 7.31 – 7.14 (m, 5H), 7.00 – 6.88 (m, 2H), 6.84 (dd, *J* = 8.2, 1.7 Hz, 1H), 5.94 (dd, *J* = 10.8, 3.8 Hz, 1H), 4.01 – 3.83 (m, 1H), 3.82 (s, 1H), 3.80 (s, 1H), 3.04 (m, 1H);
^{13}C NMR (100 MHz, CDCl$_3$) δ = 168.0, 160.0, 152.6, 140.4, 137.8, 134.2, 132.0, 130.0, 127.7, 124.0, 123.5, 120.7, 120.2, 113.8, 112.9, 56.1, 55.4, 48.0, 32.1 (t, *J* = 21.0 Hz);
^{19}F NMR (376 MHz, CDCl$_3$) δ = -88.13 to -89.13 (m, 2F), -116.81 to -118.84 (m, 2F);
IR (film) 3006.42, 2958.75, 2839.85, 1774.05, 1709.70, 1601.58, 1586.72, 1502.37, 1466.53, 1438.41, 1384.81, 1356.67, 1334.16, 1306.82, 1282.38, 1261.56, 1190.65, 1171.36, 1108.49, 1044.30, 966.59, 930.53, 871.67, 750.58, 721.48, 695.69 cm^{-1};
HRMS (ESI+) calcd for $C_{26}H_{22}F_4NO_5$ $[M+H]^+$ m/z = 504.1429; found: 504.1422.

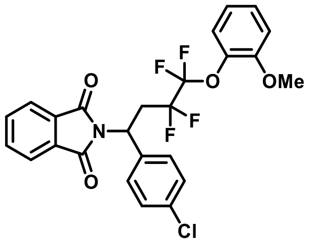

2-(1-(4-chlorophenyl)-3,3,4,4-tetrafluoro-4-(2-methoxyphenoxy)butyl)isoindoline-1,3-dione (4b): Compound **4b** was synthesized following the general procedure 1 (standard-scale), using 1-(2-bromo-1,1,2,2-tetrafluoroethoxy)-2-methoxybenzene (120.4 mg, 0.4 mmol), *N*-Vinylphthalimide (34.6 mg, 0.2 mmol) and 4-chlorophenylmagnesium bromide (0.8 mL, 1.0 M solution in diethyl ether, 0.8 mmol). The product **4b** was obtained as a colorless liquid (40.6 mg, 40% yield) after purification by column chromatography on silica gel with hexane/EtOAc (8:2).
^{1}H NMR (400 MHz, CDCl$_3$) δ = 7.85 – 7.79 (m, 2H), 7.73 – 7.67 (m, 2H), 7.55 (d, *J* = 8.5 Hz, 2H), 7.33 (d, *J* = 8.6 Hz, 2H), 7.23 – 7.18 (m, 2H), 6.99 – 6.87 (m, 2H), 5.93 (dd, *J* = 10.3, 4.2 Hz, 1H), 3.90 – 3.72 (m, 4H), 3.14 – 2.97 (m, 1H);
^{13}C NMR (100 MHz, CDCl$_3$) δ = 167.9, 152.6, 137.7, 137.4, 134.4, 134.3, 131.9, 129.5, 129.1, 127.7, 124.0, 123.6, 120.7, 112.9, 56.1, 47.5, 32.1, 32.1 (t, *J* = 21.0 Hz);
^{19}F NMR (376 MHz, CDCl$_3$) δ = -88.16 to -89.12 (m, 2F), -116.81 to -118.53 (m, 2F);
IR (film) 2946.98, 2841.94, 1774.44, 1711.76, 1604.70, 1501.36, 1466.40, 1439.82, 1383.42, 1356.38, 1315.75, 1281.93, 1260.65, 1189.41, 1170.02, 1104.30, 1058.76, 1043.04, 1015.11, 960.02, 895.29, 832.69, 770.25, 747.39, 730.96, 714.71, 612.67, 559.84, 529.89 cm^{-1};
HRMS (+APCI) calcd for $C_{25}H_{19}ClF_4NO_4$ $[M+H]^+$ m/z = 508.0933; found: 508.0935.

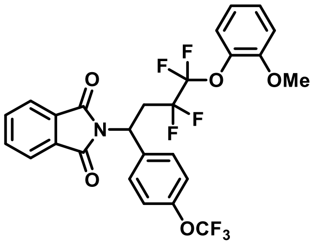

2-(3,3,4,4-tetrafluoro-4-(2-methoxyphenoxy)-1-(4 (trifluoromethoxy)phenyl)butyl)isoindoline-1,3-dione (4c): Compound **4c** was synthesized following the general procedure 1 (standard-scale), using 1-(2-bromo-1,1,2,2-tetrafluoroethoxy)-2-methoxybenzene (120.4 mg, 0.4 mmol), *N*-Vinylphthalimide (34.6 mg, 0.2 mmol) and 4-(Trifluoromethoxy)phenylmagnesium bromide (1.6 mL, 0.5 M solution in THF, 0.8 mmol). The product **4c** was obtained as a colorless liquid (61.3 mg, 55% yield) after purification by column chromatography on silica gel with hexane/EtOAc (8:2).
^{1}H NMR (400 MHz, CDCl$_3$) δ = 7.86 – 7.80 (m, 2H), 7.73 – 7.68 (m, 2H), 7.66 (d, *J* = 8.8 Hz, 2H), 7.26 – 7.16 (m, 4H), 6.99 – 6.89 (m, 2H), 5.98 (dd, *J* = 10.4, 4.3 Hz, 1H), 3.92 – 3.76 (m, 4H), 3.16 – 3.00 (m, 1H);
^{13}C NMR (100 MHz, CDCl$_3$) δ = 167.91, 152.5, 149.1 (q, *J* = 1.8 Hz)., 137.7, 137.6, 134.3, 131.9, 129.7, 127.7, 123.9, 123.6, 121.3, 120.7, 120.5 (q, *J* = 256.0 Hz), 120.3 – 114.0 (m), 112.9, 56.1, 47.4, 32.2 (t, *J* = 21.0 Hz);
^{19}F NMR (376 MHz, CDCl$_3$) δ = -57.84 (s, 3F), -88.18 to -89.06 (m, 2F), -116.81 to -118.56 (m, 2F);
IR (film) 1775.60, 1712.74, 1608.92, 1503.36, 1467.91, 1440.94, 1385.09, 1362.81, 1257.90, 1215.59, 1169.95, 1108.48, 1059.82, 1044.17, 1021.19, 961.73, 895.85, 855.04, 768.02, 750.58, 718.41, 615.99, 563.75, 530.37 cm^{-1};
HRMS (+APCI) calcd for $C_{26}H_{19}F_7NO_5$ $[M+H]^+$ m/z = 558.1146; found: 558.1147.

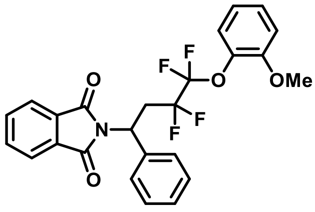

2-(3,3,4,4-tetrafluoro-4-(2-methoxyphenoxy)-1-phenylbutyl)isoindoline-1,3-dione (4d): Compound **4d** was synthesized following the general procedure 1 (standard-scale), using 1-(2-bromo-1,1,2,2-tetrafluoroethoxy)-2-methoxybenzene (120.4 mg, 0.4 mmol), *N*-Vinylphthalimide (34.6 mg, 0.2 mmol) and Phenylmagnesium bromide (0.8 mL, 1.0 M solution in THF, 0.8 mmol). The product **4d** was obtained as a colorless liquid (38.8 mg, 41% yield) after purification by column chromatography on silica gel with hexane/EtOAc (8:2).
^{1}H NMR (400 MHz, CDCl$_3$) δ = 7.85 – 7.79 (m, 2H), 7.73 – 7.65 (m, 2H), 7.61 (d, *J* = 7.4 Hz, 2H), 7.41 – 7.33 (m, 2H), 7.33 – 7.27 (m, 1H), 7.24 – 7.18 (m, 2H), 7.00 – 6.88 (m, 2H), 5.97 (dd, *J* = 10.7, 3.7 Hz, 1H), 4.01 – 3.85 (m, 1H), 3.82 (s, 3H), 3.14 – 2.97 (m, 1H);
^{13}C NMR (100 MHz, CDCl$_3$) δ = 168.0, 152.6, 138.9, 137.8, 134.2, 132.0, 128.9, 128.4, 128.0, 127.7, 124.0, 123.5, 120.7, 119.6 – 115.7 (m), 112.9, 56.1, 48.0, 32.1 (t, *J* = 21.0 Hz);
^{19}F NMR (376 MHz, CDCl$_3$) δ = -88.12 to -89.16 (m, 2F), -116.87 to -118.77 (m, 2F);
IR (film) 1773.53, 1712.50, 1605.14, 1502.77, 1457.52, 1440.16, 1386.09, 1358.20, 1334.20, 1282.80, 1262.47, 1192.04, 1172.18, 1109.29, 1057.51, 1027.79, 960.62, 892.20, 751.23, 720.69, 698.61 cm^{-1};
HRMS (+APCI) calcd for $C_{25}H_{20}F_4NO_4$ $[M+H]^+$ m/z = 474.1323; found: 474.1325.

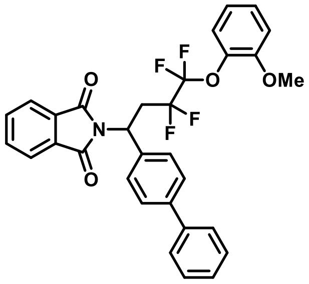

2-(1-([1,1'-biphenyl]-4-yl)-3,3,4,4-tetrafluoro-4-(2-methoxyphenoxy)butyl)isoindoline-1,3-dione (4e): Compound **4e** was synthesized following the general procedure 1 (standard-scale), using 1-(2-bromo-1,1,2,2-tetrafluoroethoxy)-2-methoxybenzene (120.4 mg, 0.4 mmol), *N*-Vinylphthalimide (34.6 mg, 0.2 mmol) and 4-Biphenylmagnesium bromide (0.8 mL, 0.5 M solution in THF, 0.4 mmol). The product **4e** was obtained as a colorless liquid (49.0 mg, 46% yield) after purification by column chromatography on silica gel with hexane/EtOAc (8:2).

^{1}H NMR (400 MHz, CDCl$_3$) δ = 7.87 – 7.81 (m, 2H), 7.73 – 7.64 (m, 4H), 7.62 – 7.52 (m, 4H), 7.43 (t, *J* = 7.5 Hz, 2H), 7.36 – 7.30 (m, 1H), 7.22 (d, *J* = 7.6 Hz, 2H), 6.99 – 6.89 (m, 2H), 6.02 (dd, *J* = 10.6, 3.8 Hz, 1H), 4.01 – 3.87 (m, 1H), 3.83 (s, 3H), 3.18 – 3.02 (m, 1H);
^{13}C NMR (100 MHz, CDCl$_3$) δ = 168.1, 152.6, 141.4, 140.6, 137.9, 137.8, 134.2, 132.0, 128.9, 128.5, 127.7, 127.3, 124.0, 123.6, 120.7, 119.2 – 116.7 (m), 112.9, 56.1, 47.8, 32.1 (t, *J* = 21.0 Hz);
^{19}F NMR (376 MHz, CDCl$_3$) δ = -88.05 to -89.27 (m, 2F), -116.73 to -118.68 (m, 2F);
IR (film) 1773.81, 1711.70, 1604.83, 1502.54, 1488.44, 1466.82, 1439.83, 1385.00, 1355.45, 1333.60, 1282.43, 1261.76, 1191.38, 1171.60, 1109.00, 1059.18, 1043.53, 1025.43, 1007.21, 960.81, 896.43, 846.11, 749.30, 737.97, 718.52, 698.23, 621.69, 570.16, 529.50 cm^{-1};
HRMS (+APCI) calcd for $C_{31}H_{24}F_4NO_4$ $[M+H]^+$ m/z = 550.1636; found: 550.1624.

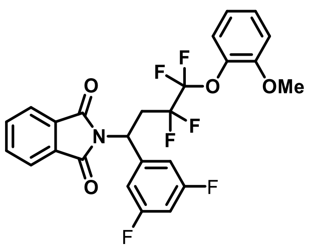

2-(1-(3,5-difluorophenyl)-3,3,4,4-tetrafluoro-4-(2-methoxyphenoxy)butyl)isoindoline-1,3-dione (4f): Compound **4f** was synthesized following the general procedure 1 (standard-scale), using 1-(2-bromo-1,1,2,2-tetrafluoroethoxy)-2-methoxybenzene (120.4 mg, 0.4 mmol), *N*-Vinylphthalimide (34.6 mg, 0.2 mmol) and 3,5-Difluorophenylmagnesium bromide (1.6 mL, 0.5 M solution in THF, 0.8 mmol). The product **4f** was obtained as a colorless liquid (50.0 mg, 49% yield) after purification by column chromatography on silica gel with hexane/EtOAc (8:2).
^{1}H NMR (400 MHz, CDCl$_3$) δ = 7.92 – 7.81 (m, 2H), 7.76 – 7.68 (m, 2H), 7.26 – 7.19 (m, 2H), 7.17 – 7.11 (m, 2H), 7.00 – 6.89 (m, 2H), 6.82 – 6.70 (m, 1H), 5.92 (dd, *J* = 10.4, 4.2 Hz, 1H), 3.84 (s, 3H), 3.82 – 3.72 (m, 1H), 3.16 – 2.97 (m, 1H);
^{13}C NMR (100 MHz, CDCl$_3$) δ = 167.8, 163.2 (dd, *J* = 248.1, 12.6 Hz) 152.5, 142.4 (t, *J* = 8.8Hz) 137.7, 134.5, 131.8, 127.8, 123.9, 123.7, 120.7, 120.2 – 116.0 (m), 112.9, 111.1 (dd, 25.9, 7.2 Hz) 104.0 (t, *J* = 25.0), 56.1, 47.4, 32.11 (t, *J* = 21.0 Hz);
^{19}F NMR (376 MHz, CDCl$_3$) δ = -88.05 to -89.27 (m, 2F), -108.32 to -108.42 (m, 2F), -116.73 to -118.68 (m, 2F);
IR (film) 1777.07, 1712.87, 1625.07, 1599.00, 1502.95, 1466.60, 1440.53, 1385.30, 1358.80, 1303.30, 1283.85, 1262.61, 1191.50, 1172.25, 1120.75, 1109.66, 1061.77, 1043.81, 1024.75, 990.01, 858.29, 749.50, 727.55, 716.70, 690.78 cm^{-1};
HRMS (+APCI) calcd for $C_{25}H_{18}F_6NO_4$ $[M+H]^+$ m/z = 510.1135; found: 510.1124.

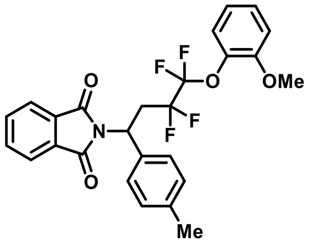

2-(3,3,4,4-tetrafluoro-4-(2-methoxyphenoxy)-1-(p-tolyl)butyl)isoindoline-1,3-dione (4g): Compound **4g** was synthesized following the general procedure 1 (standard-scale), using 1-(2-bromo-1,1,2,2-tetrafluoroethoxy)-2-methoxybenzene (120.4 mg, 0.4 mmol), *N*-Vinylphthalimide (34.6 mg, 0.2 mmol) and *p*-Tolylmagnesium bromide (0.8 mL, 1.0 M solution in THF, 0.8 mmol). The product **4g** was obtained as a colorless liquid (47.8 mg, 49% yield) after purification by column chromatography on silica gel with hexane/EtOAc (8:2).
^{1}H NMR (400 MHz, CDCl$_3$) δ = 7.84 – 7.78 (m, 2H), 7.71 – 7.65 (m, 2H), 7.49 (d, *J* = 8.3 Hz, 2H), 7.24 – 7.15 (m, 4H), 6.98 – 6.94 (m, 1H), 6.91 (td, *J* = 8.0, 1.5 Hz, 1H), 5.94 (dd, *J* = 10.7, 3.9 Hz, 1H), 3.97 – 3.83 (m, 1H), 3.82 (s, 3H), 2.32 (s, 3H);
^{13}C NMR (100 MHz, CDCl$_3$) δ = 168.0, 152.6, 138.2, 137.8, 136.0, 134.1, 132.0, 129.6, 127.9, 127.7, 124.0, 123.5, 120.7, 120.4 – 116.7 (m), 112.9, 56.1, 47.7, 32.1 (t, *J* = 21.0 Hz), 21.2;
^{19}F NMR (376 MHz, CDCl$_3$) δ = -88.18 to -89.11 (m, 2F), -116.97 to -118.72 (m, 2F);
IR (film) 1773.86, 1713.61, 1609.59, 1503.04, 1467.11, 1440.11, 1385.87, 1358.20, 1321.68, 1282.63, 1262.59, 1191.02, 1172.24, 1109.78, 1059.75, 1043.97, 1024.33, 960.40, 895.44, 821.54, 750.46, 734.30, 716.84, 615.79, 566.38 cm^{-1};
HRMS (+ESI) calcd for $C_{26}H_{22}F_4NO_4$ $[M+H]^+$ m/z = 488.1479; found: 488.1486.

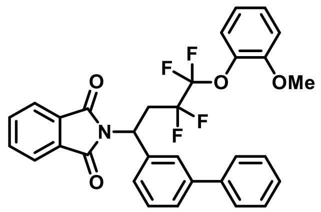

2-(1-([1,1'-biphenyl]-3-yl)-3,3,4,4-tetrafluoro-4-(2-methoxyphenoxy)butyl)isoindoline-1,3-dione (4h): Compound **4h** was synthesized following the general procedure 1 (standard-scale), using 1-(2-bromo-1,1,2,2-tetrafluoroethoxy)-2-methoxybenzene (120.4 mg, 0.4 mmol), *N*-Vinylphthalimide (34.6 mg, 0.2 mmol) and 3-Biphenylmagnesium bromide (0.8 mL, 0.5 M solution in THF, 0.4 mmol). The product **4h** was obtained as a colorless liquid (54.4 mg, 50% yield) after purification by column chromatography on silica gel with hexane/EtOAc (8:2).

^{1}H NMR (400 MHz, CDCl$_3$) δ =.7.86 – 7.81 (m, 3H), 7.72 – 7.66 (m, 2H), 7.63 – 7.54 (m, 4H), 7.45 (td, *J* = 7.8, 1.9 Hz, 3H), 7.38 – 7.33 (m, 1H), 7.24 – 7.19 (m, 2H), 6.98 – 6.90 (m, 2H), 6.06 (dd, *J* = 10.7, 3.8 Hz, 1H), 4.05 – 3.90 (m, 1H), 3.81 (s, 3H), 3.21 – 3.05 (m, 1H);

^{13}C NMR (100 MHz, CDCl$_3$) *δ* = 168.0, 152.6, 142.0, 140.8, 139.4, 137.8, 134.2, 132.0, 129.4, 129.0, 127.7, 127.4, 127.2, 127.0, 126.9, 123.9, 123.5, 120.7, 117.94, 117.6 – 114.2 (m), 112.9, 56.1, 48.1, 32.1. (t, *J* = 21.0 Hz);

^{19}F NMR (376 MHz, CDCl$_3$) δ = -88.10 to -89.11 (m, 2F), -116.87 to -118.65 (m, 2F);

IR (film) 3033.56, 2962.34, 2840.63, 1773.63, 1710.15, 1604.62, 1502.31, 1481.28, 1466.81, 1439.54, 1384.43, 1355.08, 1334.12, 1302.04, 1282.46, 1261.56, 1191.12, 1171.17, 1108.81, 1060.49, 1044.25, 1025.97, 962.37, 907.71, 750.71, 726.84, 701.60, 649.93 cm^{-1};

HRMS (+APCI) calcd for $C_{31}H_{24}F_4NO_4$ $[M+H]^+$ m/z = 550.1636; found: 550.1638.

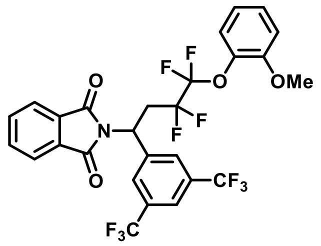

2-(1-(3,5-bis(trifluoromethyl)phenyl)-3,3,4,4-tetrafluoro-4-(2-methoxyphenoxy)butyl)isoindoline-1,3-dione (4i): Compound **4i** was synthesized following the general procedure 1 (standard-scale), using 1-(2-bromo-1,1,2,2-tetrafluoroethoxy)-2-methoxybenzene (120.4 mg, 0.4 mmol), *N*-Vinylphthalimide (34.6 mg, 0.2 mmol) and 3,5-Bis(trifluoromethyl)phenylmagnesium bromide (1.6 mL, 0.5 M solution in THF, 0.8 mmol). The product **4i** was obtained as a colorless liquid (49.1 mg, 40% yield) after purification by column chromatography on silica gel with hexane/EtOAc (8:2).

^{1}H NMR (400 MHz, CDCl$_3$) δ = 8.09 (s, 2H), 7.88 – 7.83 (m, 3H), 7.76 – 7.71 (m, 2H), 7.25 – 7.19 (m, 2H), 7.00 – 6.96 (d, *J* = 8.1 Hz, 1H), 6.95 – 6.90 (m, 1H), 6.08 (dd, *J* = 10.2, 4.4 Hz, 1H), 3.90 – 3.78 (m, 4H), 3.20 – 3.07 (m, 1H);

^{13}C NMR (100 MHz, CDCl$_3$) δ = 167.7, 152.5, 141.2, 137.6, 134.6, 132.4 (q, *J* = 33.3 Hz), 131.66, 128.6, 127.8, 123.9, 123.8, 123.2 (q, *J* = 271.1 Hz), 122.8 – 122.5 (m), 120.8, 117.4 – 114.7 (m), 112.8, 56.0, 47.3, 32.1 (t, *J* = 21.0 Hz);

^{19}F NMR (376 MHz, CDCl$_3$) δ = -62.71 to -63.09 (m, 6F), -88.30 to -89.06 (m, 2F), -116.64 to -118.22 (m, 2F);

IR (film) 1776.72, 1713.51, 1609.06, 1503.50, 1467.86, 1441.38, 1382.49, 1354.40, 1306.27, 1276.54, 1262.94, 1170.47, 1127.35, 1109.65, 1061.05, 1043.86, 1024.64, 972.95, 932.43, 902.22, 870.46, 844.61, 795.29, 749.30, 722.59, 704.65, 682.87, 665.01, 607.84, 529.46 cm^{-1};

HRMS (+APCI) calcd for $C_{27}H_{18}F_{10}NO_4$ $[M+H]^+$ m/z = 610.1071; found: 610.1056.

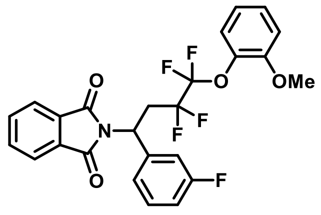

2-(3,3,4,4-tetrafluoro-1-(3-fluorophenyl)-4-(2-methoxyphenoxy)butyl)isoindoline-1,3-dione (4j): Compound **4j** was synthesized following the general procedure 1 (standard-scale), using 1-(2-bromo-1,1,2,2-tetrafluoroethoxy)-2-methoxybenzene (120.4 mg, 0.4 mmol), *N*-Vinylphthalimide (34.6 mg, 0.2 mmol) and 3-Fluorophenylmagnesium bromide (1.6 mL, 0.5 M solution in THF, 0.8 mmol). The product **4j** was obtained as a colorless liquid (59.0 mg, 60% yield) after purification by column chromatography on silica gel with hexane/EtOAc (8:2).

^{1}H NMR (400 MHz, CDCl$_3$) δ =7.86 – 7.81 (m, 2H), 7.74 – 7.68 (m, 2H), 7.39 – 7.29 (m, 3H), 7.26 – 7.19 (m, 2H), 7.04 – 6.95 (m, 2H), 6.92 (td, *J* = 8.1, 1.4 Hz, 1H), 5.96 (dd, *J* = 10.6, 4.1 Hz, 1H), 3.93 – 3.76 (m, 4H), 3.14 – 2.98 (m, 1H);
^{13}C NMR (100 MHz, CDCl$_3$) δ = 167.9, 163.0 (d, *J* = 245.3 Hz), 152.5, 142.2 (d, *J* = 7.0 Hz), 137.7, 134.3, 131.9, 130.6 (d, *J* = 8.1 Hz), 127.7, 123.9, 123.7, 123.6, 120.7, 119.1 - 115.6 (m), 115.3 (d, *J* = 20.8 Hz), 114.9 (d, *J* = 22.3 Hz) 112.9, 56.1, 47.6, 32.1 (t, *J* = 21.0 Hz);
^{19}F NMR (376 MHz, CDCl$_3$) δ = -88.14 to -89.10 (m, 2F), -111.78 to -111.96 (m, 1F), -116.73 to -118.61 (m, 2F);
IR (film) 1775.76, 1710.00, 1592.66, 1502.25, 1467.26, 1456.14, 1440.53, 1384.37, 1356.85, 1334.37, 1303.55, 1282.14, 1261.45, 1189.86, 1171.36, 1107.55, 1058.73, 1043.75, 1024.80, 972.11, 945.38, 875.44, 790.20, 749.90, 721.31, 692.61, 640.56, 606.67, 559.16, 529.87, 483.60 cm^{-1};
HRMS (+APCI) calcd for $C_{25}H_{19}F_5NO_4$ [M+H]$^+$ m/z = 492.1229; found: 492.1217.

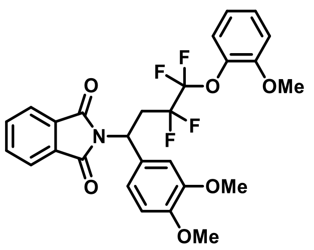

2-(1-(3,4-dimethoxyphenyl)-3,3,4,4-tetrafluoro-4-(2-methoxyphenoxy)butyl)isoindoline-1,3-dione (4k): Compound **4k** was synthesized following the general procedure 1 (standard-scale), using 1-(2-bromo-1,1,2,2-tetrafluoroethoxy)-2-methoxybenzene (120.4 mg, 0.4 mmol), *N*-Vinylphthalimide (34.6 mg, 0.2 mmol) and 3,4-Dimethoxyphenylmagnesium bromide (0.8 mL, 0.5 M solution in THF, 0.4 mmol). The product **4k** was obtained as a colorless liquid (37.8 mg, 35% yield) after purification by column chromatography on silica gel with hexane/EtOAc (8:2).
^{1}H NMR (400 MHz, CDCl$_3$) δ = 7.85 – 7.77 (m, 2H), 7.72 – 7.65 (m, 2H), 7.25 – 7.15 (m, 4H), 6.98 – 6.88 (m, 2H), 6.84 (d, *J* = 8.9 Hz, 1H), 5.89 (dd, *J* = 10.6, 3.9 Hz, 1H), 3.96 – 3.87 (m, 4H), 3.86 (s, 3H), 3.82 (s, 3H), 3.12 – 2.95 (m, 1H);
^{13}C NMR (100 MHz, CDCl$_3$) δ = 168.1, 152.6, 149.1, 149.1, 137.8, 134.2, 132.0, 131.6, 127.7, 124.0, 123.5, 120.7, 120.5, 119.2 – 116.3 (m), 112.9, 111.4, 111.1, 56.1, 56.0, 48.0, 32.3 (t, *J* = 21.0 Hz);
^{19}F NMR (376 MHz, CDCl$_3$) δ = -88.11 to -89.18 (m, 2F), -117.05 to -118.69 (m, 2F);
IR (film) 3008.01, 2958.89, 2938.22, 2839.11, 1774.15, 1708.17, 1605.33, 1502.11, 1465.09, 1441.06, 1425.50, 1384.94, 1356.30, 1331.92, 1300.17, 1281.68, 1260.46, 1189.11, 1169.82, 1106.93, 1058.74, 1042.27, 1024.94, 968.65, 942.71, 876.05, 856.49, 811.41, 749.79, 736.75, 718.65, 655.19, 559.27, 529.77 cm^{-1};
HRMS (+APCI) calcd for $C_{27}H_{24}F_4NO_6$ [M+H]$^+$ m/z = 534.1534; found: 534.1522.

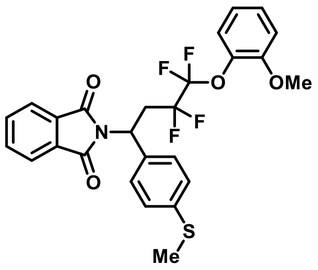

2-(3,3,4,4-tetrafluoro-4-(2-methoxyphenoxy)-1-(4-(methylthio)phenyl)butyl)isoindoline-1,3-dione (4l): Compound **4l** was synthesized following the general procedure 1 (standard-scale), using 1-(2-bromo-1,1,2,2-tetrafluoroethoxy)-2-methoxybenzene (120.4 mg, 0.4 mmol), *N*-Vinylphthalimide (34.6 mg, 0.2 mmol) and 4-Thioanisolephenylmagnesium bromide (0.8 mL, 0.5 M solution in THF, 0.4 mmol). The product **4l** was obtained as a colorless liquid (52.3 mg, 50% yield) after purification by column chromatography on silica gel with hexane/EtOAc (8:2).
^{1}H NMR (400 MHz, CDCl$_3$) δ = 7.86 – 7.78 (m, 2H), 7.73 – 7.66 (m, 2H), 7.52 (d, *J* = 8.3 Hz, 2H), 7.25 – 7.18 (m, 4H), 6.98 – 6.89 (m, 2H), 5.92 (dd, *J* = 10.5, 4.1 Hz, 1H), 3.93 - 3.75 (m, 4H), 3.12 – 2.98 (m, 1H), 2.46 (s, 3H);
^{13}C NMR (100 MHz, CDCl$_3$) *δ* = 168.0, 152.6, 139.0, 137.8, 135.6, 134.2, 132.0, 128.5, 127.7, 126.8, 124.0, 123.5, 120.7, 119.2 – 116.3 (m), 112.9, 56.1, 47.6, 32.1 (t, *J* = 21.0 Hz), 15.8;
^{19}F NMR (376 MHz, CDCl$_3$) δ = -88.01 to -89.35 (m, 2F), -116.92 to -118.75 (m, 2F);
IR (film) 2840.72, 2923.26, 2964.21, 1773.42, 1713.04, 1601.83, 1502.50, 1466.88, 1439.49, 1385.54, 1357.75, 1282.66, 1262.11, 1191.83, 1172.09, 1109.10, 1059.07, 1017.55, 959.91, 894.41, 771.17, 750.37, 733.41, 717.20 cm^{-1};
HRMS (+APCI) calcd for $C_{26}H_{22}F_4NO_4S$ [M+H]$^+$ m/z = 520.1200; found: 520.1206.

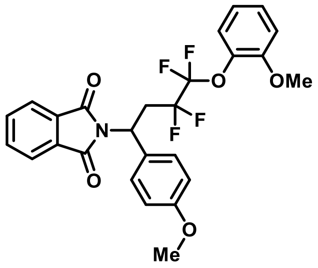

2-(3,3,4,4-tetrafluoro-4-(2-methoxyphenoxy)-1-(4-methoxyphenyl)butyl)isoindoline-1,3-dione (4m): Compound **4m** was synthesized following the general procedure 1 (standard-scale), using 1-(2-bromo-1,1,2,2-tetrafluoroethoxy)-2-methoxybenzene (120.4 mg, 0.4 mmol), *N*-Vinylphthalimide (34.6 mg, 0.2 mmol) and 4-methoxyphenylmagnesium bromide (0.8 mL, 0.5 M solution in THF, 0.4 mmol). The product **4m** was obtained as a colorless liquid (41.8 mg, 42% yield) after purification by column chromatography on silica gel with hexane/EtOAc (8:2).

^{1}H NMR (400 MHz, CDCl$_3$) δ = 7.83 – 7.77 (m, 2H), 7.70 – 7.65 (m, 2H), 7.54 (d, *J* = 8.7 Hz, 2H), 7.21 (d, *J* = 7.6 Hz, 2H), 6.98 – 6.87 (m, 4H), 5.93 (dd, *J* = 10.6, 3.9 Hz, 1H), 3.95 – 3.80 (m, 4H), 3.78 (s, 3H), 3.11 – 2.97 (m, 1H);

^{13}C NMR (100 MHz, CDCl$_3$) δ = 168.1, 159.7, 137.9, 134.2, 132.1, 131.3, 129.4, 127.8, 124.1, 123.6, 120.8, 118.0 – 116.4 (m), 114.3, 113.0, 56.2, 55.5, 47.6, 32.3 (t, *J* = 21.0 Hz);

^{19}F NMR (376 MHz, CDCl$_3$) δ = -88.01 to -89.28 (m, 2F), -117.00 to -118.79 (m, 2F);

IR (film) 2957.74, 2934.12, 2840.22, 1773.24, 1709.06, 1610.89, 1514.31, 1502.01, 1466.11, 1440.50, 1385.21, 1355.92, 1331.83, 1305.09, 1282.15, 1260.26, 1170.81, 1144.16, 1106.51, 1059.04, 1028.05, 1003.06, 958.62, 894.28, 833.97, 810.36, 795.37, 749.77, 739.03, 716.15, 614.64, 567.47, 529.21cm^{-1};

HRMS (+APCI) calcd for $C_{26}H_{22}F_4NO_5$ [M+H]$^+$ m/z = 504.1429; found: 504.1416.

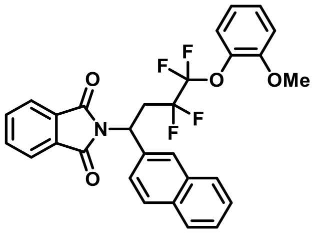

2-(3,3,4,4-tetrafluoro-4-(2-methoxyphenoxy)-1-(naphthalen-2-yl)butyl)isoindoline-1,3-dione (4n): Compound **4n** was synthesized following the general procedure 1 (standard-scale), using 1-(2-bromo-1,1,2,2-tetrafluoroethoxy)-2-methoxybenzene (120.4 mg, 0.4 mmol), *N*-Vinylphthalimide (34.6 mg, 0.2 mmol) and 2-naphthylmagnesium bromide (0.8 mL, 0.5 M solution in THF, 0.4 mmol). The product **4n** was obtained as a colorless liquid (59.1 mg, 56% yield) after purification by column chromatography on silica gel with hexane/EtOAc (8:2).

^{1}H NMR (400 MHz, CDCl$_3$) δ = 8.06 (s, 1H), 7.89 – 7.79 (m, 5H), 7.76 (dd, *J* = 8.6, 1.8 Hz, 1H), 7.71 – 7.65 (m, 2H), 7.52 – 7.46 (m, 2H), 7.26 – 7.20 (m, 2H), 6.99 – 6.95 (m, 1H), 6.92 (td, *J* = 7.8, 1.4 Hz, 1H), 6.17 (dd, *J* = 10.7, 3.9 Hz, 1H), 4.14 – 3.93 (m, 1H), 3.82 (s, 3H), 3.28 – 3.12 (m, 1H);

^{13}C NMR (100 MHz, CDCl$_3$) δ = 168.1, 152.6, 137.8, 136.2, 134.2, 133.3, 133.1, 132.0, 128.8, 128.3, 127.7, 127.7, 127.1, 126.6, 126.5, 125.6, 123.9, 123.5, 120.7, 119.3 – 114.2 (m), 112.9, 56.1, 48.2, 32.1 (t, *J* = 21.0 Hz);

^{19}F NMR (376 MHz, CDCl$_3$) δ = -87.98 to -89.07 (m, 2F), -116.68 to -118.58 (m, 2F);

IR (film) 3058.97, 3010.59, 2960.24, 2840.70, 1774.25, 1709.03, 1602.84, 1501.30, 1466.69, 1439.21, 1380.57, 1353.76, 1333.79, 1282.55, 1261.61, 1189.26, 1170.69, 1106.06, 1058.08, 1043.08, 1024.27, 1005.10, 968.88, 950.40, 918.91, 894.67, 857.97, 821.28, 746.21, 717.33, 662.20, 607.41, 561.99, 529.77, 477.84 cm^{-1};

HRMS (+APCI) calcd for $C_{29}H_{22}F_4NO_4$ [M+H]$^+$ m/z = 524.1479; found: 524.1468.

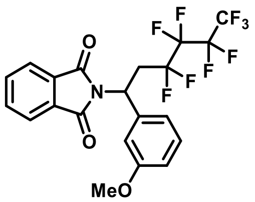

2-(3,3,4,4,5,5,6,6,6-nonafluoro-1-(3-methoxyphenyl)hexyl)isoindoline-1,3-dione (5a): Compound **5a** was synthesized following the general procedure 1 (standard-scale), using Perfluorobutyl bromide (119.6 mg, 0.4 mmol), *N*-Vinylphthalimide (34.6 mg, 0.2 mmol) and 3-methoxyphenylmagnesium bromide (0.8 mL, 1.0 M solution in THF, 0.8 mmol). The product **5a** was obtained as a colorless liquid (34.3 mg, 34% yield) after purification by column chromatography on silica gel with hexane/EtOAc (9:1).

^{1}H NMR (400 MHz, CDCl$_3$) δ = 7.82 – 7.72 (m, 2H), 7.70 – 7.64 (m, 2H), 7.23 (d, *J* = 7.3 Hz, 1H), 7.13 – 7.06 (m, 2H), 6.81 (dd, *J* = 8.2, 1.8 Hz, 1H), 5.79 (dd, *J* = 10.6, 3.7 Hz, 1H), 3.90 – 3.72 (m, 4H), 2.88 – 2.71 (m, 1H);
^{13}C NMR (100 MHz, CDCl$_3$) δ = 167.9, 160.1, 139.8, 134.4, 131.8, 130.2, 123.7, 120.1, 118.9 – 115.7 (m), 114.1, 113.7, 55.4, 47.6, 31.6 (t, *J* = 21.0 Hz);
^{19}F NMR (376 MHz, CDCl$_3$) δ = -80.88 to -81.12 (m, 3F), -114.05 to -116.64 (m, 2F), -124.21 to -124.64 (m, 2F), -125.74 to -126.13 (m, 2F);
IR (film) 1775.97, 1713.06, 1601.86, 1587.75, 1492.24, 1468.57, 1437.55, 1386.03, 1357.03, 1326.13, 1220.19, 1133.97, 1050.48, 1021.52, 971.40, 932.21, 882.10, 723.09, 695.06 cm^{-1};
HRMS (+APCI) calcd for $C_{21}H_{15}F_9NO_3$ $[M+H]^+$ m/z = 500.0903; found: 500.0895.

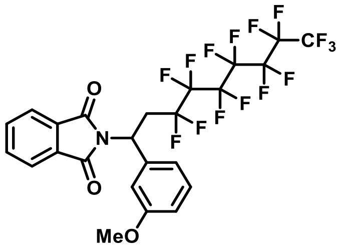

2-(3,3,4,4,5,5,6,6,7,7,8,8,9,9,9-pentadecafluoro-1-(3-methoxyphenyl)nonyl)isoindoline-1,3-dione (5b): Compound **5b** was synthesized following the general procedure 1 (standard-scale), using 1-Bromoperfluoroheptane (179.6 mg, 0.4 mmol), *N*-Vinylphthalimide (34.6 mg, 0.2 mmol) and 3-methoxyphenylmagnesium bromide (0.8 mL, 1.0 M solution in THF, 0.8 mmol). The product **5b** was obtained as a colorless liquid (69.2 mg, 53% yield) after purification by column chromatography on silica gel with hexane/EtOAc (9:1).
^{1}H NMR (400 MHz, CDCl$_3$) δ = 7.86 – 7.79 (m, 2H), 7.74 – 7.68 (m, 2H), 7.31 – 7.26 (m, 1H), 7.17 – 7.09 (m, 2H), 6.85 (dd, *J* = 8.3, 1.9 Hz, 1H), 5.83 (dd, *J* = 10.7, 3.7 Hz, 1H), 3.94 – 3.75 (m, 4H), 2.92 – 2.75 (m, 1H);
^{13}C NMR (100 MHz, CDCl$_3$) δ = 167.9, 160.1, 139.8, 134.4, 131.8, 130.2, 123.7, 120.1, 118.3 – 109.9 (m), 114.1, 113.8, 55.5, 47.6, 31.1 (t, *J* = 21.0 Hz);
^{19}F NMR (376 MHz, CDCl$_3$) δ = -80.70 to -80.84 (m, 3F), -113.77 to -116.37 (m, 2F), -121.42 to -121.68 (m, 2F), -121.95 to -121.16 (m, 2F), -122.57 to -122.85 (m, 2F), -123.37 to -123.58 (m, 2F), -126.01 to -126.22 (m, 2F);
IR (film) 1777.36, 1715.09, 1601.56, 1492.29, 1468.35, 1438.33, 1386.52, 1362.71, 1330.36, 1240.45, 1208.94, 1147.75, 1063.62, 970.52, 931.12, 871.65, 793.82, 722.17, 696.33, 662.31 cm^{-1};
HRMS (+APCI) calcd for $C_{24}H_{15}F_{15}NO_3$ $[M+H]^+$ m/z = 650.0807; found: 650.0794.

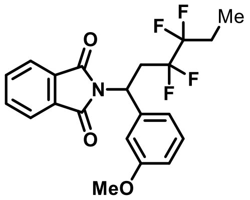

2-(3,3,4,4-tetrafluoro-1-(3-methoxyphenyl)hexyl)isoindoline-1,3-dione (5c): Compound **5c** was synthesized following the general procedure 1 (standard-scale), using 1-Bromo-1,1,2,2-tetrafluorobutane (83.6 mg, 0.4 mmol), *N*-Vinylphthalimide (34.6 mg, 0.2 mmol) and 3-methoxyphenylmagnesium bromide (0.8 mL, 1.0 M solution in THF, 0.8 mmol). The product **5c** was obtained as a colorless liquid (29.8 mg, 36% yield) after purification by column chromatography on silica gel with hexane/EtOAc (9:1).
^{1}H NMR (400 MHz, CDCl$_3$) δ = 7.84 – 7.78 (m, 2H), 7.71 – 7.65 (m, 2H), 7.28 – 7.23 (m, 1H), 7.18 – 7.11 (m, 2H), 6.83 (ddd, *J* = 8.3, 2.5, 0.9 Hz, 1H), 5.82 (dd, *J* = 10.8, 3.8 Hz, 1H), 3.82 – 3.63 (m, 4H), 2.85 – 2.68 (m, 1H), 2.13 – 1.86 (m, 2H), 1.06 (t, *J* = 7.5 Hz, 3H);
^{13}C NMR (100 MHz, CDCl$_3$) *δ* = 168.0, 160.0, 140.4, 134.2, 131.9, 130.0, 123.5, 121.6 – 116.7 (m), 120.2, 113.9, 113.8, 55.4, 48.0, 30.8 (t, *J* = 20.9 Hz), 23.3 (t, *J* = 23.5 Hz), 4.9 (t, *J* = 5.0 Hz);
^{19}F NMR (376 MHz, CDCl$_3$) δ = -115.02 to -117.13 (m, 2F), -117.21 to -117.46 (m, 2F);
IR (film) 2994.63, 2952.58, 2892.73, 2838.74, 1773.87, 1709.67, 1600.81, 1586.86, 1491.27, 1467.50, 1436.98, 1385.95, 1358.74, 1332.93, 1290.13, 1261.97, 1172.76, 1158.43, 1139.83, 1050.01, 1003.71, 968.58, 925.99, 871.06, 785.50, 738.02, 721.67, 697.04, 530.09, 513.44 cm^{-1};
HRMS (+APCI) calcd for $C_{21}H_{20}F_4NO_3$ $[M+H]^+$ m/z = 410.1374; found: 410.1372.

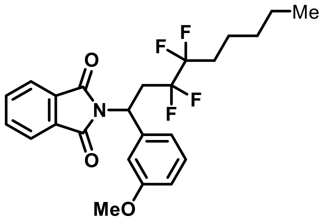

2-(3,3,4,4-tetrafluoro-1-(3-methoxyphenyl)nonyl)isoindoline-1,3-dione (5d): Compound **5d** was synthesized following the general procedure 1 (standard-scale), using 1-Bromo-1,1,2,2-tetrafluoroheptane (100.4 mg, 0.4 mmol), *N*-Vinylphthalimide (34.6 mg, 0.2 mmol) and 3-methoxyphenylmagnesium bromide (0.8 mL, 1.0 M solution in THF, 0.8 mmol). The product **5d** was obtained as a colorless liquid (31.3 mg, 35% yield) after purification by column chromatography on silica gel with hexane/EtOAc (9:1).

^{1}H NMR (400 MHz, CDCl$_3$) δ = 7.78 – 7.70 (m, 2H), 7.65 – 7.57 (m, 2H), 7.21 – 7.15 (m, 1H), 7.10 – 7.01 (m, 2H), 6.75 (dd, *J* = 8.2, 2.3 Hz, 1H), 5.74 (dd, *J* = 10.8, 3.6 Hz, 1H), 3.72 (s, 3H), 3.70 – 3.52 (m, 1H), 2.78 – 2.59 (m, 1H), 1.96 – 1.74 (m, 2H), 1.52 – 1.38 (m, 2H), 1.27 – 1.18 (m, 4H), 0.80 (t, *J* = 6.6 Hz, 3H);

^{13}C NMR (100 MHz, CDCl$_3$) δ = 168.0, 160.0, 140.4, 134.2, 131.9, 130.0, 123.5, 121.1 – 118.7 (m), 120.2, 113.9, 113.8, 55.4, 48.0, 31.5, 30.8 (t, *J* = 21.0 Hz), 29.8 (t, *J* = 22.8 Hz), 22.4, 20.2, 14.0;

^{19}F NMR (376 MHz, CDCl$_3$) δ = -115.09 to -117.01 (m, 2F), -115.27 to -115.49 (m, 2F);

IR (film) 2957.75, 2873.30, 1774.39, 1711.68, 1601.05, 1587.03, 1491.51, 1467.60, 1436.26, 1386.04, 1359.63, 1332.46, 1262.52, 1173.77, 1139.78, 1046.65, 967.27, 928.95, 722.39, 695.49, 529.72 cm^{-1};

HRMS (+APCI) calcd for $C_{24}H_{26}F_4NO_3$ [M+H]$^+$ m/z = 452.1843; found: 452.1842.

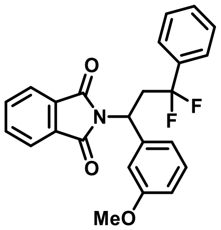

2-(3,3-difluoro-1-(3-methoxyphenyl)-3-phenylpropyl)isoindoline-1,3-dione (5e): Compound **5e** was synthesized following the general procedure 1 (standard-scale), using (bromodifluoromethyl)benzene (82.8 mg, 0.4 mmol), *N*-Vinylphthalimide (34.6 mg, 0.2 mmol) and 3-methoxyphenylmagnesium bromide (0.8 mL, 1.0 M solution in THF, 0.8 mmol). The product **5e** was obtained as a colorless liquid (11.0 mg, 14% yield) after purification by column chromatography on silica gel with hexane/EtOAc (9:1).

^{1}H NMR (400 MHz, CDCl$_3$) δ = 7.72 – 7.66 (m, 2H), 7.66 – 7.59 (m, 2H), 7.44 – 7.38 (m, 2H), 7.22 (t, *J* = 7.9 Hz, 3H), 7.17 – 7.04 (m, 3H), 6.80 (dd, *J* = 8.2, 2.3 Hz, 1H), 5.67 (d, *J* = 11.2 Hz, 1H), 4.02 – 3.86 (m, 1H), 3.78 (s, 3H), 2.88 – 2.74 (m, 1H).

^{13}C NMR (100 MHz, CDCl$_3$) δ = 168.0, 159.9, 140.7, 136.4. 133.9, 131.8, 129.9, 129.7, 128.5, 125.0 (t, *J* = 5.9 Hz), 123.20, 122.0 (t, *J* = 241.1 Hz), 120.2, 113.8, 113.7, 55.4, 49.2, 39.4 (t, *J* = 27.1 Hz).

^{19}F NMR (376 MHz, CDCl$_3$) δ = -90.75 to -91.87 (m, 1F), -99.11 to -100.19 (m, 1F).

IR (film) 2941.65, 1772.45, 1708.79, 1600.64, 1586.48, 1491.16, 1467.41, 1452.11, 1436.58, 1385.97, 1357.82, 1322.90, 1270.19, 1156.06, 1115.96, 1087.04, 1043.33, 955.30, 926.09, 872.43, 784.93, 735.66, 720.63, 697.35, 570.83, 530.57, 417.13 cm^{-1};

HRMS (-APCI) calcd for $C_{24}H_{19}F_2NO_3$ [M]$^-$ m/z = 407.1328; found: 407.1340.

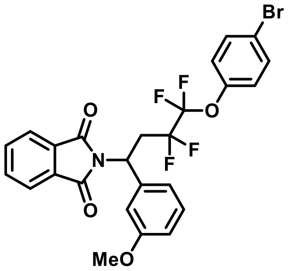

2-(4-(4-bromophenoxy)-3,3,4,4-tetrafluoro-1-(3-methoxyphenyl)butyl)isoindoline-1,3-dione (5f): Compound **5f** was synthesized following the general procedure 1 (standard-scale), using 1-bromo-4-(2-bromo-1,1,2,2-tetrafluoroethoxy)benzene (140.6 mg, 0.4 mmol), *N*-Vinylphthalimide (34.6 mg, 0.2 mmol) and 3-methoxyphenylmagnesium bromide (0.8 mL, 1.0 M solution in THF, 0.8 mmol). The product **5f** was obtained as a colorless liquid (36.7 mg, 33% yield) after purification by column chromatography on silica gel with hexane/EtOAc (9:1).

^{1}H NMR (400 MHz, CDCl$_3$) δ = 7.81 – 7.75 (m, 2H), 7.68 – 7.62 (m, 2H), 7.46 – 7.41 (m, 2H), 7.23 (d, *J* = 8.1 Hz, 1H), 7.16 – 7.09 (m, 2H), 7.03 (d, *J* = 8.9 Hz, 2H), 6.81 (dd, *J* = 8.2, 1.7 Hz, 1H), 5.84 (dd, *J* = 10.6, 3.8 Hz, 1H), 3.95 – 3.79 (m, 1H), 3.77 (s, 3H), 2.96 – 2.79 (m, 1H);
^{13}C NMR (100 MHz, CDCl$_3$) *δ* = 168.0, 160.0, 148.1, 140.1, 134.3, 132.8, 131.9, 130.1, 123.59, 123.57, 120.2, 119.99, 118.8 – 114.7 (m), 113.9, 113.8, 55.4, 48.0, 32.0 (t, *J* = 21.0 Hz);
^{19}F NMR (376 MHz, CDCl$_3$) δ = -87.98 to -88.95 (m, 2F), -117.03 to -119.15 (m, 2F);
IR (film) 2959.54, 2838.30, 1773.87, 1708.99, 1600.46, 1586.11, 1484.56, 1467.52, 1436.20, 1384.29, 1354.60, 1333.47, 1308.27, 1262.36, 1184.58, 1110.66, 1093.63, 1066.80, 1046.34, 1012.18, 965.77, 929.92, 870.85, 853.67, 825.62, 780.77, 720.92, 694.51, 647.89, 529.39, 493.24 cm^{-1};
HRMS (+ESI) calcd for $C_{25}H_{19}BrF_4NO_4$ [M+H]$^+$ m/z = 552.0428; found: 552.0417.

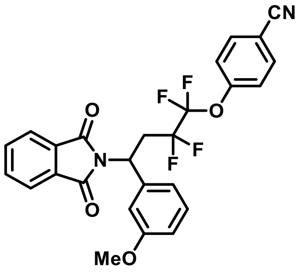

4-(4-(1,3-dioxoisoindolin-2-yl)-1,1,2,2-tetrafluoro-4-(3-methoxyphenyl)butoxy)benzonitrile (5g): Compound **5g** was synthesized following the general procedure 1 (standard-scale), using 4-(2-bromo-1,1,2,2-tetrafluoroethoxy)benzonitrile (119.2 mg, 0.4 mmol), *N*-Vinylphthalimide (34.6 mg, 0.2 mmol) and 3-methoxyphenylmagnesium bromide (0.8 mL, 1.0 M solution in THF, 0.8 mmol). The product **5g** was obtained as a colorless liquid (35.0 mg, 35% yield) after purification by column chromatography on silica gel with hexane/EtOAc (9:1).
^{1}H NMR (400 MHz, CDCl$_3$) δ = 7.85 – 7.80 (m, 2H), 7.73 – 7.65 (m, 4H), 7.35 – 7.26 (m, 3H), 7.20 – 7.11 (m, 2H), 6.85 (dd, *J* = 7.9, 2.1 Hz, 1H), 5.87 (dd, *J* = 10.6, 3.9 Hz, 1H), 3.96 – 3.82 (m, 1H), 3.81 (s, 3H), 2.98 – 2.82 (m, 1H);
^{13}C NMR (100 MHz, CDCl$_3$) *δ* = 168.0, 160.0, 152.4, 140.0, 134.3, 134.1, 131.9, 130.1, 123.6, 122.2, 120.1, 118.0, 117.5 - 116.1 (m), 113.9, 113.9, 110.6, 55.5, 47.9, 31.9 (t, *J* = 21.0 Hz);
^{19}F NMR (376 MHz, CDCl$_3$) δ = -88.38 (s, 2F), -117.09 to -119.04 (m, 2F);
IR (film) 2232.26, 1773.82, 1709.67, 1601.63, 1586.40, 1493.48, 1467.57, 1436.67, 1384.89, 1356.31, 1334.55, 1308.09, 1263.29, 1172.01, 1119.96, 1047.14, 1018.34, 966.78, 930.59, 869.68, 842.48, 786.90, 722.71, 695.35, 649.22 cm^{-1};
HRMS (+APCI) calcd for $C_{26}H_{19}F_4N_2O_4$ [M+H]$^+$ m/z = 499.1275; found: 499.1275.

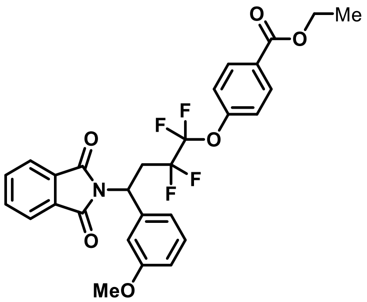

Ethyl 4-(4-(1,3-dioxoisoindolin-2-yl)-1,1,2,2-tetrafluoro-4-(3-methoxyphenyl)butoxy)benzoate (5h): Compound **5h** was synthesized following the general procedure 1 (standard-scale), using ethyl 4-(2-bromo-1,1,2,2-tetrafluoroethoxy)benzoate (138.0 mg, 0.4 mmol), *N*-Vinylphthalimide (34.6 mg, 0.2 mmol) and 3-methoxyphenylmagnesium bromide (0.8 mL, 1.0 M solution in THF, 0.8 mmol). The product **5h** was obtained as a colorless liquid (54.5 mg, 50% yield) after purification by column chromatography on silica gel with hexane/EtOAc (9:1).
^{1}H NMR (400 MHz, CDCl$_3$) δ = 8.05 (d, *J* = 8.8 Hz, 2H), 7.86 – 7.79 (m, 2H), 7.72 – 7.66 (m, 2H), 7.31 – 7.21 (m, 3H), 7.20 – 7.14 (m, 2H), 6.85 (dd, *J* = 8.3, 1.8 Hz, 1H), 5.89 (dd, *J* = 10.7, 3.8 Hz, 1H), 4.37 (q, *J* = 7.1 Hz, 2H), 3.98 – 3.82 (m, 1H), 3.80 (s, 3H), 3.01 – 2.83 (m, 1H), 1.39 (t, *J* = 7.1 Hz, 3H);
^{13}C NMR (100 MHz, CDCl$_3$) *δ* = 168.0, 165.7, 160.0, 152.6, 140.1, 134.2, 131.9, 131.4, 130.1, 128.7, 123.6, 121.2, 120.2, 117.9 – 114.8 (m), 113.9, 113.8, 61.3, 55.4, 48.0, 32.0 (t, *J* = 21.0 Hz), 14.4;
^{19}F NMR (376 MHz, CDCl$_3$) δ = -88.34 (s, 2F), -117.12 to -119.19 (m, 2F);
IR (film) 2981.43, 2940.66, 1774.32, 1709.29, 1604.57, 1504.63, 1491.85, 1467.45, 1437.17, 1384.80, 1357.26, 1334.28, 1308.00, 1274.75, 1164.66, 1106.14, 1048.09, 1018.51, 966.93, 930.77, 872.08, 757.32, 726.39, 695.15 cm^{-1};
HRMS (+APCI) calcd for $C_{28}H_{24}F_4NO_6$ [M+H]$^+$ m/z = 546.1534; found: 546.1522.

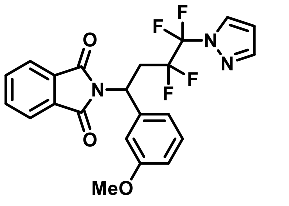

2-(3,3,4,4-tetrafluoro-1-(3-methoxyphenyl)-4-(1H-pyrazol-1-yl)butyl)isoindoline-1,3-dione (5i): Compound **5i** was synthesized following the general procedure 1 (standard-scale), using 1-(2-bromo-1,1,2,2-tetrafluoroethyl)-1H-pyrazole (98.8 mg, 0.4 mmol), *N*-Vinylphthalimide (34.6 mg, 0.2 mmol) and 3-methoxyphenylmagnesium bromide (0.8 mL, 1.0 M solution in THF, 0.8 mmol). The product **5i** was obtained as a colorless liquid (44.6 mg, 50% yield) after purification by column chromatography on silica gel with hexane/EtOAc (9:1).

^{1}H NMR (400 MHz, $CDCl_3$) δ = 7.89 – 7.75 (m, 3H), 7.73 (s, 1H), 7.72 – 7.65 (m, 2H), 7.29 – 7.22 (m, 1H), 7.15 – 7.09 (m, 2H), 6.83 (dd, *J* = 8.2, 2.4 Hz, 1H), 6.44 – 6.42 (m, 1H), 5.81 (dd, *J* = 10.8, 3.7 Hz, 1H), 3.97 – 3.82 (m, 1H), 3.79 (s, 3H), 3.06 – 2.90 (m, 1H);

^{13}C NMR (100 MHz, $CDCl_3$) *δ* = 167.9, 160.0, 143.3, 139.9, 134.2, 131.9, 130.0, 129.2, 123.5, 120.1, 117.7 – 113.6 (m), 113.9, 113.7, 108.2, 55.4, 47.9, 31.8 (t, *J* = 21.0 Hz);

^{19}F NMR (376 MHz, $CDCl_3$) δ = -97.63 to -99.15 (s, 2F), -114.88 to -116.66 (m, 2F);

IR (film) 2958.97, 2838.55, 1773.77, 1709.57, 1600.52, 1587.01, 1525.22, 1491.52, 1467.66, 1426.98, 1386.34, 1359.02, 1262.30, 1206.91, 1162.79, 1143.25, 1118.26, 1047.72, 963.10, 917.21, 900.77, 871.40, 760.17, 728.53, 695.54, 639.47 cm^{-1};

HRMS (+APCI) calcd for $C_{22}H_{18}F_4N_3O_3$ $[M+H]^+$ m/z = 448.1279; found: 448.1267.

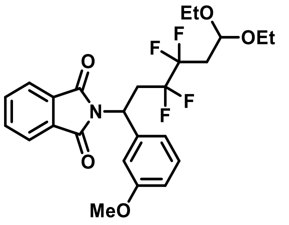

2-(6,6-diethoxy-3,3,4,4-tetrafluoro-1-(3-methoxyphenyl)hexyl)isoindoline-1,3-dione (5j): Compound **5j** was synthesized following the general procedure 1 (standard-scale), using 1-bromo-4,4-diethoxy-1,1,2,2-tetrafluorobutane (98.8 mg, 0.4 mmol), *N*-Vinylphthalimide (34.6 mg, 0.2 mmol) and 3-methoxyphenylmagnesium bromide (0.8 mL, 1.0 M solution in THF, 0.8 mmol). The product **5j** was obtained as a colorless liquid (32.5 mg, 33% yield) after purification by column chromatography on silica gel with hexane/EtOAc (8:2).

^{1}H NMR (400 MHz, $CDCl_3$) δ = 7.83 – 7.78 (m, 2H), 7.71 – 7.65 (m, 2H), 7.29 – 7.22 (m, 1H), 7.16 – 7.09 (m, 2H), 6.83 (dd, *J* = 8.3, 1.8 Hz, 1H), 5.80 (dd, *J* = 10.8, 3.8 Hz, 1H), 4.87 (t, *J* = 5.3 Hz, 1H), 3.80 (s, 3H), 3.77 – 3.58 (m, 3H), 3.56 – 3.47 (m, 2H), 2.86 – 2.70 (m, 1H), 2.34 (td, *J* = 18.9, 5.2 Hz, 2H), 1.18 (td, *J* = 7.1, 2.4 Hz, 6H).

^{13}C NMR (100 MHz, $CDCl_3$) δ = 168.0, 160.0, 140.3, 134.2, 131.9, 130.0, 123.5, 120.2, 118.7 – 114.8 (m), 113.9, 113.7, 97.4, 61.8, 55.4, 47.9, 34.6 (t, *J* = 20.5 Hz), 30.5 (t, *J* = 21.2 Hz), 15.2.

^{19}F NMR (376 MHz, $CDCl_3$) δ = -114.14 to -114.38 (s, 2F), -115.17 to -117.21 (m, 2F).

IR (film) 2977.15, 2932.17, 2899.69, 1774.37, 1711.74, 1600.77, 1587.10, 1491.79, 1467.86, 1437.04, 1385.75, 1359.96, 1333.54, 1263.00, 1174.22, 1138.10, 1095.95, 1054.45, 969.82, 871.42, 787.23, 723.21, 695.36, 529.93 cm^{-1};

HRMS (-APCI) calcd for $C_{25}H_{27}F_4NO_5$ $[M]^-$ m/z = 497.1820; found: 497.1831.

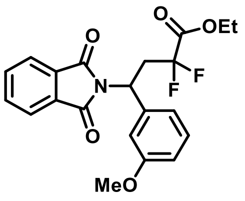

Ethyl 4-(1,3-dioxoisoindolin-2-yl)-2,2-difluoro-4-(3-methoxyphenyl)butanoate (5k): Compound **5k** was synthesized following the general procedure 1 (standard-scale), using ethyl 2-bromo-2,2-difluoroacetate (81.2 mg, 0.4 mmol), *N*-Vinylphthalimide (34.6 mg, 0.2 mmol) and 3-methoxyphenylmagnesium bromide (0.8 mL, 1.0 M solution in THF, 0.8 mmol). The product **5k** was obtained as a colorless liquid (32.3 mg, 40% yield) after purification by column chromatography on silica gel with hexane/EtOAc (9:1).

^{1}H NMR (400 MHz, CDCl$_3$) δ = 7.84 – 7.78 (m, 2H), 7.72 – 7.66 (m, 2H), 7.28 – 7.22 (m, 1H), 7.14 – 7.08 (m, 2H), 6.83 (dd, *J* = 8.3 Hz, 2.0 Hz, 1H), 5.70 (dd, *J* = 10.4, 4.1 Hz, 1H), 4.18 (q, *J* = 7.1 Hz, 2H), 3.79 (s, 3H), 3.75 – 3.63 (m, 1H), 3.01 – 2.84 (m, 1H), 1.29 (t, *J* = 7.2 Hz, 3H);
^{13}C NMR (100 MHz, CDCl$_3$) *δ* = 167.9, 163.7 (t, *J* = 32.3 Hz), 160.0, 139.9, 134.3, 131.9, 130.0, 123.5, 120.3, 115.2 (t, *J* = 250.1 Hz), 114.0, 113.8, 112.7, 63.3, 55.4, 48.5, 35.5 (t, *J* = 23.1 Hz), 14.0;
^{19}F NMR (376 MHz, CDCl$_3$) δ = -104.78 to -106.89 (s, 2F);
IR (film) 1768.95, 1713.00, 1601.19, 1586.80, 1492.33, 1467.82, 1385.92, 1331.08, 1262.87, 1191.01, 1138.75, 1089.02, 1051.05, 965.61, 872.12, 789.89, 723.07, 696.17 cm^{-1};
HRMS (+APCI) calcd for $C_{21}H_{20}F_2NO_5$ $[M+H]^+$ m/z = 404.1304; found: 404.1304.

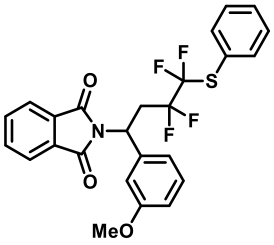

2-(3,3,4,4-tetrafluoro-1-(3-methoxyphenyl)-4-(phenylthio)butyl)isoindoline-1,3-dione (5l): Compound **5l** was synthesized following the general procedure 1 (standard-scale), using (2-bromo-1,1,2,2-tetrafluoroethyl)(phenyl)sulfane (81.2 mg, 0.4 mmol), *N*-Vinylphthalimide (34.6 mg, 0.2 mmol) and 3-methoxyphenylmagnesium bromide (0.8 mL, 1.0 M solution in THF, 0.8 mmol). The product **5l** was obtained as a colorless liquid (64.1 mg, 65% yield) after purification by column chromatography on silica gel with hexane/EtOAc (9:1).
^{1}H NMR (400 MHz, CDCl$_3$) δ = 7.85 – 7.79 (m, 2H), 7.72 – 7.66 (m, 2H), 7.64 – 7.58 (m, 2H), 7.48 – 7.42 (m, 1H), 7.41 – 7.34 (m, 2H), 7.30 – 7.24 (m, 1H), 7.16 – 7.09 (m, 2H), 6.84 (dd, *J* = 8.2, 1.7 Hz, 1H), 5.82 (dd, *J* = 10.8, 3.6 Hz, 1H), 3.91 – 3.71 (m, 4H), 2.90 – 2.72 (m, 1H);
^{13}C NMR (100 MHz, CDCl$_3$) δ = 168.0, 160.0, 140.2, 137.3, 134.2, 131.9, 130.6, 130.0, 129.3, 124.2 – 118.5 (m), 123.6, 120.2, 113.9, 113.8, 55.4, 48.0, 31.9 (t, *J* = 21.3 Hz);
^{19}F NMR (376 MHz, CDCl$_3$) δ = -88.73 to -88.81 (m, 2F), -111.88 to -113.94 (m, 2F);
IR (film) 3061.43, 3004.00, 2958.17, 2837.96, 1773.64, 1710.28, 1600.66, 1586.62, 1491.16, 1467.89, 1441.08, 1386.09, 1359.03, 1332.74, 1289.62, 1262.91, 1172.53, 1141.30, 1089.66, 1058.86, 925.61, 750.96, 725.47, 691.94 cm^{-1};
HRMS (-APCI) calcd for $C_{25}H_{19}F_4NO_3S$ $[M]^-$ m/z = 489.1016; found: 489.1029.

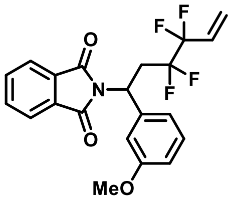

2-(3,3,4,4-tetrafluoro-1-(3-methoxyphenyl)hex-5-en-1-yl)isoindoline-1,3-dione (5m): Compound **5m** was synthesized following the general procedure 1 (standard-scale), using 4-bromo-3,3,4,4-tetrafluorobut-1-ene (82.8 mg, 0.4 mmol), *N*-Vinylphthalimide (34.6 mg, 0.2 mmol) and 3-methoxyphenylmagnesium bromide (0.8 mL, 1.0 M solution in THF, 0.8 mmol). The product **5m** was obtained as a colorless liquid (20.1 mg, 25% yield) after purification by column chromatography on silica gel with hexane/EtOAc (9:1).
^{1}H NMR (400 MHz, CDCl$_3$) δ = 7.85 – 7.78 (m, 2H), 7.72 – 7.66 (m, 2H), 7.30 – 7.23 (m, 1H), 7.17 – 7.10 (m, 2H), 6.83 (dd, *J* = 8.2, 1.7 Hz, 1H), 5.98 – 5.79 (m, 3H), 5.68 (d, *J* = 10.3 Hz, 1H), 3.80 (s, 3H), 3.78 – 3.60 (m, 1H), 2.86 – 2.68 (m, 1H);
^{13}C NMR (100 MHz, CDCl$_3$) δ = 168.0, 160.0, 140.3, 134.2, 131.9, 130.0, 126.1 (t, *J* = 24.2 Hz), 124.5 (t, *J* = 9.3 Hz), 123.5, 120.2, 118.5 – 112.6 (m), 113.9, 113.8, 55.4, 48.0, 31.0 (t, *J* = 21.1 Hz);
^{19}F NMR (376 MHz, CDCl$_3$) δ = -114.28 to -117.27 (m, 4F);
IR (film) 1774.28, 1712.35, 1601.15, 1586.91, 1491.92, 1468.00, 1437.10, 1386.92, 1360.71, 1331.71, 1261.49, 1189.20, 1140.65, 1115.09, 1051.92, 1011.25, 958.94, 723.61, 695.57 cm^{-1};
HRMS (+APCI) calcd for $C_{21}H_{18}F_4NO_3$ $[M+H]^+$ m/z = 408.1217; found: 408.1211.

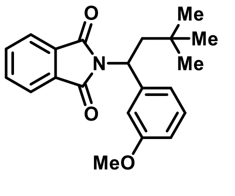

2-(1-(3-methoxyphenyl)-3,3-dimethylbutyl)isoindoline-1,3-dione (5n): Compound **5n** was synthesized following the general procedure 1 (standard-scale), using 2-iodo-2-methylpropane (73.6 mg, 0.4 mmol), *N*-Vinylphthalimide (34.6 mg, 0.2 mmol) and 3-methoxyphenylmagnesium bromide (0.4 mL, 1.0 M solution in THF, 0.4 mmol). The product **5n** was obtained as a colorless liquid (18.9 mg, 28% yield) after purification by column chromatography on silica gel with hexane/EtOAc (9:1).

^{1}H NMR (400 MHz, $CDCl_3$) δ = 7.82 – 7.75 (m, 2H), 7.69 – 7.63 (m, 2H), 7.24 – 7.18 (m, 1H), 7.17 – 7.12 (m, 2H), 6.78 (d, *J* = 8.1 Hz, 1H), 5.47 (dd, *J* = 9.2, 4.4 Hz, 1H), 3.80 (s, 3H), 2.75 (dd, *J* = 14.7, 9.2 Hz, 1H), 2.09 (dd, *J* = 14.7, 4.5 Hz, 1H), 0.93 (s, 9H);

^{13}C NMR (100 MHz, $CDCl_3$) δ = 168.5, 159.8, 143.0, 134.0, 132.1, 129.6, 123.3, 120.7, 114.1, 113.2, 55.4, 52.1, 43.9, 30.9, 29.7;

IR (film) 2955.36, 2867.50, 2836.30, 1769.63, 1706.63, 1599.54, 1585.27, 1491.41, 1467.13, 1385.46, 1365.02, 1331.61, 1267.10, 1087.57, 1037.45, 741.59, 720.44, 697.05 cm^{-1};

HRMS (+APCI) calcd for $C_{21}H_{24}NO_3[M+H]^+$ m/z = 338.1751; found: 338.1752.

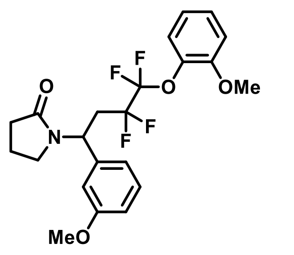

1-(3,3,4,4-tetrafluoro-4-(2-methoxyphenoxy)-1-(3-methoxyphenyl)butyl)pyrrolidin-2-one (6a): Compound **6a** was synthesized following the general procedure 1 (standard-scale), using 1-(2-bromo-1,1,2,2-tetrafluoroethoxy)-2-methoxybenzene (120.4 mg, 0.4 mmol), 1-vinylpyrrolidin-2-one (22.2 mg, 0.2 mmol) and 3-methoxyphenylmagnesium bromide (0.8 mL, 1.0 M solution in THF, 0.8 mmol). The product **6a** was obtained as a colorless liquid (31.8 mg, 36% yield) after purification by column chromatography on silica gel with hexane/EtOAc (8:2).

^{1}H NMR (400 MHz, $CDCl_3$) δ = 7.31 – 7.20 (m, 3H), 6.99 – 6.91 (m, 4H), 6.85 (dd, *J* = 8.2, 2.0 Hz, 1H), 5.80 (dd, *J* = 10.6, 4.1 Hz, 1H), 3.83 (s, 3H), 3.81 (s, 3H), 3.48 – 3.41 (m, 1H), 3.18 – 3.09 (m, 1H), 3.09 – 2.94 (m, 1H), 2.92 – 2.75 (m, 1H), 2.45 – 2.31 (m, 2H), 2.06 – 1.86 (m, 2H);

^{13}C NMR (100 MHz, $CDCl_3$) δ = 174.7, 160.0, 152.5, 140.3, 137.8, 129.9, 127.7, 123.9, 120.7, 120.4 – 115.0 (m), 119.5, 113.5, 113.3, 112.9, 56.1, 55.4, 48.4, 43.2, 31.5, 31.1 (t, *J* = 21.2 Hz), 18.2;

^{19}F NMR (376 MHz, $CDCl_3$) δ = -88.55 to -89.40 (m, 2F), -117.22 to -119.40 (m, 2F).

IR (film) 2948.59, 1682.54, 1601.23, 1585.90, 1501.54, 1457.78, 1420.12, 1282.63, 1259.94, 1187.33, 1168.69, 1105.83, 1063.86, 1042.06, 1014.76, 749.27, 729.78, 702.19, 564.88, 483.55 cm^{-1};

HRMS (+APCI) calcd for $C_{22}H_{24}F_4NO_4[M+H]^+$ m/z = 442.1636; found: 442.1630.

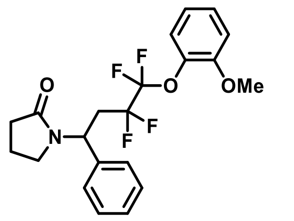

1-(3,3,4,4-tetrafluoro-4-(2-methoxyphenoxy)-1-phenylbutyl)pyrrolidin-2-one (6b): Compound **6b** was synthesized following the general procedure 1 (standard-scale), using 1-(2-bromo-1,1,2,2-tetrafluoroethoxy)-2-methoxybenzene (120.4 mg, 0.4 mmol), 1-vinylpyrrolidin-2-one (22.2 mg, 0.2 mmol) and Phenylmagnesium bromide (0.8 mL, 1.0 M solution in THF, 0.8 mmol). The product **6b** was obtained as a colorless liquid (29.6 mg, 36% yield) after purification by column chromatography on silica gel with hexane/EtOAc (8:2).

^{1}H NMR (400 MHz, $CDCl_3$) δ = 7.39 – 7.29 (m, 5H), 7.29 – 7.20 (m, 3H), 6.99 – 6.91 (m, 2H), 5.83 (dd, *J* = 10.5, 4.2 Hz, 1H), 3.83 (s, 3H), 3.50 – 3.41 (m, 1H), 3.18 – 3.00 (m, 2H), 2.92 – 2.79 (m, 1H), 2.47 – 2.32 (m, 2H), 2.04 – 1.90 (m, 2H);

^{13}C NMR (100 MHz, $CDCl_3$) δ = 174.7, 152.5, 138.8, 137.8, 128.9, 128.1, 127.7, 127.4, 123.9, 120.8, 119.1 – 115.0 (m), 112.9, 56.1, 48.5, 43.3, 31.5, 31.1 (t, *J* = 21.2 Hz), 18.2.

^{19}F NMR (376 MHz, $CDCl_3$) δ = -88.48 to -89.44 (m, 2F), -117.23 to -119.27 (m, 2F);

IR (film) 2954.16, 2925.55, 1682.98, 1501.07, 1457.33, 1419.98, 1282.73, 1261.33, 1187.54, 1169.61, 1106.26, 1074.55, 1043.01, 1016.34, 750.82, 700.95 cm^{-1};

HRMS (+APCI) calcd for $C_{21}H_{22}F_4NO_3[M+H]^+$ m/z = 412.1530; found: 412.1524.

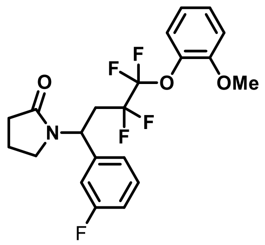

1-(3,3,4,4-tetrafluoro-1-(3-fluorophenyl)-4-(2-methoxyphenoxy)butyl)pyrrolidin-2-one (6c): Compound **6c** was synthesized following the general procedure 1 (standard-scale), using 1-(2-bromo-1,1,2,2-tetrafluoroethoxy)-2-methoxybenzene (120.4 mg, 0.4 mmol), 1-vinylpyrrolidin-2-one (22.2 mg, 0.2 mmol) and 3-Fluorophenylmagnesium bromide (1.6 mL, 0.5 M solution in THF, 0.8 mmol). The product **6c** was obtained as a colorless liquid (35.2 mg, 41% yield) after purification by column chromatography on silica gel with hexane/EtOAc (8:2).

^{1}H NMR (400 MHz, CDCl$_3$) δ = 7.37 – 7.30 (m, 1H), 7.27 – 7.21 (m, 2H), 7.18 – 7.13 (m, 1H), 7.11 – 7.05 (m, 1H), 7.04 – 6.92 (m, 3H), 5.79 (dd, *J* = 10.3, 4.2 Hz, 1H), 3.83 (s, 3H), 3.50 – 3.41 (m, 1H), 3.18 – 3.11 (m, 1H), 3.10 – 2.95 (m, 1H), 2.91 – 2.77 (m, 1H), 2.49 – 2.33 (m, 2H), 2.06 – 1.92 (m, 2H);

^{13}C NMR (100 MHz, CDCl$_3$) δ = 174.8l, 163.1 (d, *J* = 245.4 Hz), 152.5, 141.3 (d, *J* = 6.6 Hz), 137.8, 130.5 (d, *J* = 8.2 Hz), 127.7, 123.9, 132.1 (d, *J* = 2.9 Hz), 120.8, 118.9 - 116.4 (m), 115.1 (d, *J* = 20.9 Hz), 114.4 (d, *J* = 21.9 Hz), 112.9, 56.1, 48.2, 43.4, 31.4, 31.2 (t, *J* = 22.7 Hz), 18.3;

^{19}F NMR (376 MHz, CDCl$_3$) δ = -88.40 to -89.49 (m, 2F), -111.99 to -112.16 (m, 1F), -117.12 to -119.13 (m, 2F);

IR (film) 2951.21, 1683.11, 1591.41, 1502.08, 1458.13, 1440.75, 1419.85, 1283.23, 1261.60, 1188.42, 1170.46, 1107.48, 1062.65, 1043.26, 1017.50, 750.62, 701.22 cm^{-1};

HRMS (+APCI) calcd for $C_{21}H_{21}F_5NO_3[M+H]^+$ m/z = 430.1436; found: 430.1432.

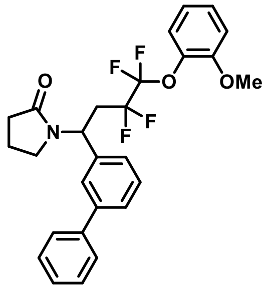

1-(1-([1,1'-biphenyl]-3-yl)-3,3,4,4-tetrafluoro-4-(2-methoxyphenoxy)butyl)pyrrolidin-2-one (6d): Compound **6d**was synthesized following the general procedure 1 (standard-scale), using 1-(2-bromo-1,1,2,2-tetrafluoroethoxy)-2-methoxybenzene (120.4 mg, 0.4 mmol), 1-vinylpyrrolidin-2-one (22.2 mg, 0.2 mmol) and 3-Biphenylmagnesium bromide (1.6 mL, 0.5 M solution in THF, 0.8 mmol). The product **6d** was obtained as a colorless liquid (62.4 mg, 64% yield) after purification by column chromatography on silica gel with hexane/EtOAc (8:2).

^{1}H NMR (400 MHz, CDCl$_3$) δ = 7.60 – 7.52 (m, 4H), 7.47 – 7.41 (m, 3H), 7.38 – 7.33 (m, 2H), 7.28 – 7.20 (m, 2H), 6.99 – 6.92 (m, 2H), 5.91 (dd, *J* = 10.4, 4.1 Hz, 1H), 3.81 (s, 3H), 3.53 – 3.45 (m, 1H), 3.23 – 3.06 (m, 2H), 2.97 – 2.83 (m, 1H), 2.47 – 2.33 (m, 2H), 2.06 – 1.91 (m, 2H);

^{13}C NMR (100 MHz, CDCl$_3$) δ = 174.7, 152.5, 142.0, 140.8, 139.3, 137.8, 129.3, 129.0, 127.7, 127.7, 127.4, 127.0, 126.3, 126.2, 123.9, 120.8, 112.9, 56.1, 48.6, 43.3, 31.5, 31.2 (t, *J* = 22.7 Hz), 18.2;

^{19}F NMR (376 MHz, CDCl$_3$) δ = -88.47 to -89.38 (m, 2F), -117.14 to -119.29 (m, 2F);

IR (film) 2952.00, 1682.43, 1501.24, 1457.46, 1419.69, 1282.58, 1261.00, 1187.61, 1168.90, 1106.53, 1042.85, 1014.59, 748.75, 700.56 cm^{-1};

HRMS (+APCI) calcd for $C_{27}H_{26}F_4NO_3[M+H]^+$ m/z = 488.1843; found: 488.1836.

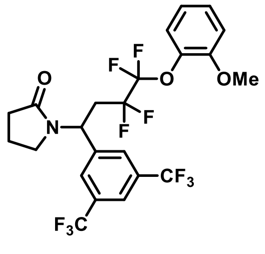

1-(1-(3,5-bis(trifluoromethyl)phenyl)-3,3,4,4-tetrafluoro-4-(2-methoxyphenoxy)butyl)pyrrolidin-2-one (6e): Compound **6e** was synthesized following the general procedure 1 (standard-scale), using 1-(2-bromo-1,1,2,2-tetrafluoroethoxy)-2-methoxybenzene (120.4

mg, 0.4 mmol), 1-vinylpyrrolidin-2-one (22.2 mg, 0.2 mmol) and 3,5-Bis(trifluoromethyl)phenylmagnesium bromide (1.6 mL, 0.5 M solution in THF, 0.8 mmol). The product **6e** was obtained as a colorless liquid (30.7 mg, 28% yield) after purification by column chromatography on silica gel with hexane/EtOAc (8:2).

^{1}H NMR (400 MHz, CDCl$_3$) δ = 7.88 – 7.81 (m, 3H), 7.27 – 7.20 (m, 2H), 7.01 – 6.92 (m, 2H), 5.86 (dd, *J* = 10.1, 4.4 Hz, 1H), 3.83 (s, 3H), 3.55 – 3.48 (m, 1H), 3.23 – 3.08 (m, 2H), 2.96 – 2.81 (m, 1H), 2.49 – 2.34 (m, 2H), 2.11 – 1.97 (m, 2H);

^{13}C NMR (100 MHz, CDCl$_3$) δ = 175.1, 152.4, 141.6, 137.6, 132.4 (q, *J* = 33.2 Hz), 127.8, 127.7 (q, *J* = 2.9 Hz), 123.8, 123.2 (q, *J* = 271.3 Hz), 122.3 (qu, *J* = 3.8 Hz), 120.9 117.8 - 114.8 (m), 112.8, 56.0, 48.6, 43.8, 31.21 (t, *J* = 22.7 Hz), 31.18, 18.3;

^{19}F NMR (376 MHz, CDCl$_3$) δ = -62.56 to -62.97 (m, 6F), -88.55 to -89.38 (m, 2F), -116.75 to -118.93 (m, 2F);

IR (film) 2954.45, 2926.93, 1685.32, 1503.45, 1375.74, 1276.21, 1168.31, 1123.73, 1107.96, 1043.12, 1016.87, 904.20, 844.40, 749.04, 707.22, 682.12 cm^{-1};

HRMS (+APCI) calcd for $C_{23}H_{20}F_{10}NO_3[M+H]^+$ m/z = 548.1278; found: 548.1270.

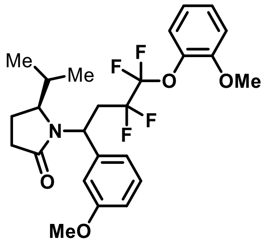

(5*S*)-5-isopropyl-1-(3,3,4,4-tetrafluoro-4-(2-methoxyphenoxy)-1-(3-methoxyphenyl)butyl)pyrrolidin-2-one (6f): Compound **6f** was synthesized following the general procedure 1 (standard-scale), using 1-(2-bromo-1,1,2,2-tetrafluoroethoxy)-2-methoxybenzene (120.4 mg, 0.4 mmol), (*S*)-5-isopropyl-1-vinylpyrrolidin-2-one (30.6 mg, 0.2 mmol) and 3-methoxyphenylmagnesium bromide (0.8 mL, 1.0 M solution in THF, 0.8 mmol). The product **6f** was obtained as a colorless liquid (30.2 mg, 31% yield) after purification by column chromatography on silica gel with hexane/EtOAc (8:2). The compounds are formed as a mixture of separable diastereomers in a ratio 3:2 as determined by crude ^{1}H NMR.

^{1}H NMR (400 MHz, CDCl$_3$, major) δ = 7.26 – 7.19 (m, 4H), 7.16 – 7.11 (m, 1H), 6.97 – 6.90 (m, 2H), 6.82 (dd, *J* = 7.9, 2.3 Hz, 1H), 4.78 (dd, *J* = 9.8, 3.9 Hz, 1H), 4.06 – 3.87 (m, 1H), 3.81 (s, 3H), 3.80 (s, 3H), 3.68 – 3.57 (m, 1H), 2.77 – 2.58 (m, 1H), 2.42 – 2.34 (m, 2H), 2.04 – 1.92 (m, 2H), 1.81 – 1.71 (m, 1H), 0.86 (d, *J* = 6.9 Hz, 3H), 0.56 (d, *J* = 6.8 Hz, 3H);

^{1}H NMR (400 MHz, CDCl$_3$, minor) δ = 7.25 – 7.18 (m, 3H), 7.09 – 7.04 (m, 2H), 6.98 – 6.90 (m, 2H), 6.85 – 6.80 (m, 1H), 5.15 (dd, *J* = 7.8, 5.8 Hz, 1H), 3.84 (s, 3H), 3.60 – 3.53 (m, 1H), 3.52 – 3.36 (m, 1H), 3.33 – 3.15 (m, 1H), 2.48 – 2.36 (m, 1H), 2.33 – 2.21 (m, 1H), 2.03 – 1.92 (m, 1H), 1.90 – 1.73 (m, 2H), 0.87 (d, *J* = 6.9 Hz, 3H), 0.68 (d, *J* = 6.6 Hz, 3H);

^{13}C NMR (100 MHz, CDCl$_3$, major) δ = 176.3, 159.7, 152.4, 142.4, 137.7, 129.5, 127.5, 123.9, 120.6, 120.2, 119.4 – 114.9 (m), 113.7, 113.1, 112.8, 67.0, 56.0, 55.3, 54.2, 33.0 (t, *J* = 21.0 Hz), 31.8, 30.1, 18.9, 18.3, 14.3;

^{13}C NMR (100 MHz, CDCl$_3$, minor) δ = 176.8, 160.0, 152.7, 142.4, 138.0, 129.8, 127.7, 124.0, 120.8, 120.6 – 166.9 (m), 120.0, 117.84, 116.93, 113.7, 113.5, 113.0, 63.1, 56.2, 55.5, 50.5, 32.7 (t, *J* = 21.0 Hz), 31.5, 29.1, 19.3, 18.4, 14.3;

^{19}F NMR (376 MHz, CDCl$_3$, major) δ = -88.00 to -88.94 (m, 2F), -116.45 to -118.08 (m, 2F);

^{19}F NMR (376 MHz, CDCl$_3$, minor) δ = -88.29 to -89.20 (m, 2F), -116.83 to -118.94 (m, 2F);

IR (film, major) 2962.02, 1682.12, 1602.39, 1586.19, 1503.29, 1458.47, 1438.12, 1303.65, 1281.97, 1261.85, 1190.11, 1172.06, 1109.07, 1044.13, 1026.41, 751.37, 709.43 cm^{-1};

IR (film, minor) 2962.50, 1683.69, 1602.60, 1586.31, 1503.24, 1457.98, 1438.85, 1282.35, 1261.71, 1219.94, 1190.19, 1172.13, 1109.94, 1043.88, 751.31, 707.40 cm^{-1};

HRMS (+ESI) calcd for $C_{25}H_{30}F_4NO_4[M+H]^+$ m/z = 484.2105; found: 484.2100.

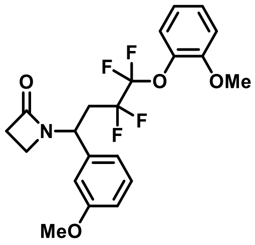

1-(3,3,4,4-tetrafluoro-4-(2-methoxyphenoxy)-1-(3-methoxyphenyl)butyl)azetidin-2-one (6g): Compound **6g** was synthesized following the general procedure 1 (standard-scale), using 1-(2-bromo-1,1,2,2-tetrafluoroethoxy)-2-methoxybenzene (120.4 mg, 0.4 mmol), 1-vinylazetidin-2-one (19.4 mg, 0.2 mmol) and 3-methoxyphenylmagnesium bromide (0.8 mL, 1.0 M solution in THF, 0.8 mmol). The product **6g** was obtained as a colorless liquid (47.6 mg, 56% yield) after purification by column chromatography on silica gel with hexane/EtOAc (8:2).

^{1}H NMR (400 MHz, CDCl$_3$) δ = 7.34 – 7.20 (m, 3H), 6.99 – 6.85 (m, 5H), 5.14 (dd, J = 9.8, 4.3 Hz, 1H), 3.82 (s, 6H), 3.31 (q, J = 4.7 Hz, 1H), 3.27 – 3.11 (m, 2H), 2.93 – 2.68 (m, 3H);
^{13}C NMR (100 MHz, CDCl$_3$) δ = 167.1, 160.2, 152.5, 140.7, 137.7, 130.2, 127.7, 123.9, 120.7, 120.4 – 113.8 (m), 119.2, 113.4, 113.1, 112.9, 56.1, 55.4, 51.0 (t, J = 3.2 Hz), 37.6, 36.1, 33.6 (t, J = 21.0 Hz);
^{19}F NMR (376 MHz, CDCl$_3$) δ = -88.43 to -89.34 (m, 2F), -116.63 to -118.24 (m, 2F);
IR (film) 2961.11, 2912.38, 2840.25, 1740.31, 1602.17, 1586.81, 1502.12, 1457.39, 1438.99, 1389.40, 1339.95, 1303.99, 1282.29, 1260.94, 1242.03, 1190.04, 1170.59, 1107.94, 1042.98, 1025.33, 992.88, 751.43, 708.28 cm^{-1};
HRMS (+APCI) calcd for $C_{21}H_{22}F_4NO_4$[M+H]$^+$ m/z = 428.1479; found: 428.1474.

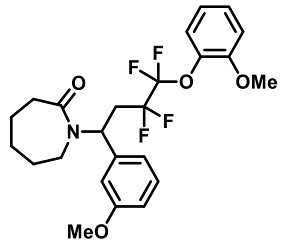

1-(3,3,4,4-tetrafluoro-4-(2-methoxyphenoxy)-1-(3-methoxyphenyl)butyl)azepan-2-one (6h): Compound **6h** was synthesized following the general procedure 1 (standard-scale), using 1-(2-bromo-1,1,2,2-tetrafluoroethoxy)-2-methoxybenzene (120.4 mg, 0.4 mmol), 1-vinylazepan-2-one (27.8 mg, 0.2 mmol) and 3-methoxyphenylmagnesium bromide (0.8 mL, 1.0 M solution in THF, 0.8 mmol). The product **6h** was obtained as a colorless liquid (77.2 mg, 82% yield) after purification by column chromatography on silica gel with hexane/EtOAc (8:2).

1-(3,3,4,4-tetrafluoro-4-(2-methoxyphenoxy)-1-(3-methoxyphenyl)butyl)azepan-2-one (6h) [1 mmol scale]: Compound **6h** was synthesized following the general procedure 1 (standard-scale), using 1-(2-bromo-1,1,2,2-tetrafluoroethoxy)-2-methoxybenzene (602.1 mg, 2.0 mmol), 1-vinylazepan-2-one (139.2 mg, 1.0 mmol) and 3-methoxyphenylmagnesium bromide (4.0 mL, 1.0 M solution in THF, 4.0 mmol). The product **6h** was obtained as a colorless liquid (313.8mg, 67% yield) after purification by column chromatography on silica gel with hexane/EtOAc (8:2).

^{1}H NMR (400 MHz, CDCl$_3$) δ = 7.28 – 7.20 (m, 3H), 6.99 – 6.90 (m, 4H), 6.83 (dd, J = 8.1, 2.1 Hz, 1H), 6.45 – 6.39 (m, 1H), 3.83 (s, 3H), 3.80 (s, 3H), 3.31 – 3.10 (m, 2H), 2.93 – 2.77 (m, 2H), 2.63 – 2.51 (m, 2H), 1.74 – 1.50 (m, 5H), 1.20 – 1.09 (m, 1H);
^{13}C NMR (100 MHz, CDCl$_3$) δ = 175.7, 159.9, 152.5, 141.3, 137.9, 129.6, 127.6, 123.8, 120.7, 119.8, 119.2 – 114.1 (m), 113.7, 113.0, 112.9, 56.1, 55.4, 49.5, 44.6, 37.7, 30.8 (t, J = 21.4 Hz), 30.0, 28.4, 23.2;
^{19}F NMR (376 MHz, CDCl$_3$) δ = -88.68 to -89.60 (m, 2F), -116.90 to -118.70 (m, 2F);
IR (film) 2857.28, 2840.54, 1637.78, 1601.70, 1585.73, 1501.92, 1456.14, 1438.24, 1417.12, 1304.34, 1281.71, 1260.00, 1222.36, 1186.06, 1169.93, 1107.19, 1067.83, 1042.54, 1026.90, 972.32, 929.99, 784.09, 749.18, 732.08, 708.79, 565.02, 487.74 cm^{-1};
HRMS (+APCI) calcd for $C_{24}H_{28}F_4NO_4$[M+H]$^+$ m/z = 470.1949; found: 470.1936.

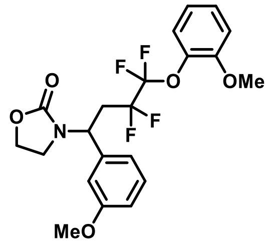

3-(3,3,4,4-tetrafluoro-4-(2-methoxyphenoxy)-1-(3-methoxyphenyl)butyl)oxazolidin-2-one (6i): Compound **6i** was synthesized following the general procedure 1 (standard-scale), using 1-(2-bromo-1,1,2,2-tetrafluoroethoxy)-2-methoxybenzene (120.4 mg, 0.4 mmol), 3-vinyloxazolidin-2-one (22.6 mg, 0.2 mmol) and 3-methoxyphenylmagnesium bromide (0.8 mL, 1.0 M solution in THF, 0.8 mmol). The product **6i** was obtained as a colorless liquid (46.9 mg, 53% yield) after purification by column chromatography on silica gel with hexane/EtOAc (7:3).
^{1}H NMR (400 MHz, CDCl$_3$) δ = 7.35 – 7.21 (m, 3H), 7.01 – 6.87 (m, 5H), 5.50 (dd, J = 10.4, 4.1 Hz, 1H), 4.35 – 4.23 (m, 2H), 3.83 (s, 3H), 3.82 (s, 3H), 3.61 (q, J = 8.5 Hz, 1H), 3.34 (q, J = 8.4 Hz, 1H), 3.17 – 3.00 (m, 1H), 2.93 – 2.78 (m, 1H);
^{13}C NMR (100 MHz, CDCl$_3$) δ = 160.2, 157.6, 152.5, 139.4, 137.8, 130.1, 127.7, 123.9, 120.8, 119.4, 117.7 – 116.4 (m), 113.8, 113.5, 112.9, 62.1, 56.1, 55.5, 50.9, 41.1, 31.7 (t, J = 20.0 Hz);
^{19}F NMR (376 MHz, CDCl$_3$) δ = -88.43 to -89.34 (m, 2F), -116.62 to -117.88 (m, 2F);
IR (film) 2946.94, 2840.49, 1742.83, 1602.15, 1586.73, 1502.14, 1457.16, 1422.38, 1306.21, 1282.44, 1259.40, 1189.74, 1170.27, 1107.78, 1040.86, 1026.04, 752.06, 705.32 cm^{-1};
HRMS (+APCI) calcd for $C_{21}H_{22}F_4NO_5$[M+H]$^+$ m/z = 444.1429; found: 444.1423.

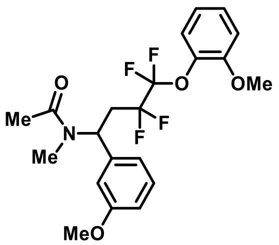

***N*-methyl-N-(3,3,4,4-tetrafluoro-4-(2-methoxyphenoxy)-1-(3-methoxyphenyl)butyl)acetamide (6j):** Compound **6j** was synthesized following the general procedure 1 (standard-scale), using 1-(2-bromo-1,1,2,2-tetrafluoroethoxy)-2-methoxybenzene (120.4 mg, 0.4 mmol), *N*-methyl-*N*-vinylacetamide (19.8 mg, 0.2 mmol) and 3-methoxyphenylmagnesium bromide (0.8 mL, 1.0 M solution in THF, 0.8 mmol). The product **6j** was obtained as a colorless liquid (63.8 mg, 74% yield) after purification by column chromatography on silica gel with hexane/EtOAc (8:2). The compounds are formed as rotamers in the ratio 2:1 as determined by crude ^{1}H NMR.

^{1}H NMR (400 MHz, CDCl$_3$) δ = 7.38 – 7.17 (m, 5H), 7.01 – 6.80 (m, 8H), 6.42 (dd, *J* = 10.0, 4.7 Hz, 1H), 5.52 (dd, *J* = 9.7, 3.7 Hz, 0.53 X 2.0, 1H), 3.83 (s, 3H), 3.82 (s, 1.53 X 2.0, 3H), 3.81 (s, 1.51 X 2.0, 3H), 3.80 (s, 3H), 3.02 – 2.78 (m, 6H), 2.76 (s, 1.55 X 2.0, 3H), 2.32 (s, 1.51 X 2.0, 3H), 2.12 (s, 3H);

^{13}C NMR (100 MHz, CDCl$_3$) δ = 170.9, 170.8, 160.2, 160.0, 152.5, 152.4, 140.9, 140.2, 137.9, 137.6, 130.2, 129.7, 127.8, 127.6, 123.9, 123.8, 120.8, 120.8, 119.5, 118.8, 117.7 – 114.4 (m), 113.7, 113.3, 113.0, 112.9, 112.8, 112.7, 56.12, 56.06, 55.44, 55.41, 54.3, 49.0, 31.9 (t, *J* = 22.1 Hz), 31.0, 30.5 (t, *J* = 21.6 Hz), 28.2, 22.5, 21.6;

^{19}F NMR (376 MHz, CDCl$_3$) δ = -88.74 to -89.22 (m, 2F), -116.63 to -119.78 (m, 2F);

IR (film) 3004.15, 2946.02, 2840.30, 1645.35, 1601.79, 1585.85, 1501.48, 1456.38, 1438.14, 1398.70, 1302.75, 1282.01, 1259.86, 1221.46, 1187.62, 1169.41, 1106.23, 1041.85, 1005.56, 927.00, 749.74, 710.18, 695.58, 598.69, 559.66, 516.52, 491.74 cm^{-1};

HRMS (+ESI) calcd for $C_{21}H_{24}F_4NO_4[M+H]^+$ m/z = 430.1636; found: 430.1626.

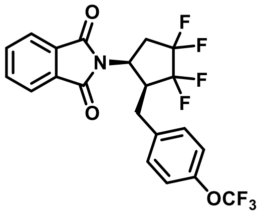

2-(3,3,4,4-tetrafluoro-2-(4-(trifluoromethoxy)benzyl)cyclopentyl)isoindoline-1,3-dione (7): Compound **7** was synthesized following the general procedure 1 (standard-scale), using 4-bromo-3,3,4,4-tetrafluorobut-1-ene (82.8 mg, 0.4 mmol), *N*-Vinylphthalimide (34.6 mg, 0.2 mmol) and 4-(Trifluoromethoxy)phenylmagnesium bromide (1.6 mL, 0.5 M solution in THF, 0.8 mmol). The product **7** was obtained as a white solid (27.7 mg, 30% yield) after purification by column chromatography on silica gel with hexane/EtOAc (8:2). The compounds are formed as a mixture of separable diastereomers in a ratio 1:3.5 as determined by crude ^{1}H NMR.

^{1}H NMR (400 MHz, CDCl$_3$, major) δ = 7.80 – 7.65 (m, 4H), 7.00 (d, *J* = 8.3 Hz, 2H), 6.87 (d, *J* = 8.1 Hz, 2H), 5.03 (q, *J* = 10.4 Hz, 1H), 3.68 – 3.47(m, 1H), 3.38 – 3.14 (m, 1H), 3.06 (dd, *J* = 15.1, 6.7 Hz, 1H), 2.75 (dd, *J* = 15.0, 9.9 Hz, 1H), 2.57 – 2.40 (m, 1H);

^{13}C NMR (100 MHz, CDCl$_3$, major) δ = 168.1, 147.9, 135.4, 134.7, 131.1, 130.1 – 119.1 (m), 129.5, 128.1, 123.6, 121.1, 120.4 (t, *J* = 255.5 Hz), 44.8(t, *J* = 19.6 Hz), 43.0 (t, *J* = 10.3 Hz), 31.8 (t, *J* = 21.9 Hz), 28.2 (d, *J* = 7.9 Hz);

^{19}F NMR (376 MHz, CDCl$_3$, major) δ = -57.71 to -58.09 (m, 3F), -115.13 to 116.11 (m, 1F), -121.58 to -122.56 (m, 1F), -124.22 to -125.49 (m, 2F);

IR (film) 2953.66, 2921.81, 2852.81, 1777.93, 1716.81, 1509.56, 1465.21, 1406.91, 1377.27, 1354.65, 1257.99, 1215.66, 1153.11, 1091.58, 1017.64, 982.15, 921.27, 839.89, 722.03, 675.93, 530.56 cm^{-1};

HRMS (+ESI) calcd for $C_{21}H_{15}F_7NO_3[M+H]^+$ m/z = 461.0856; found: 461.0870.

6. Procedure for the deprotection of 4a.

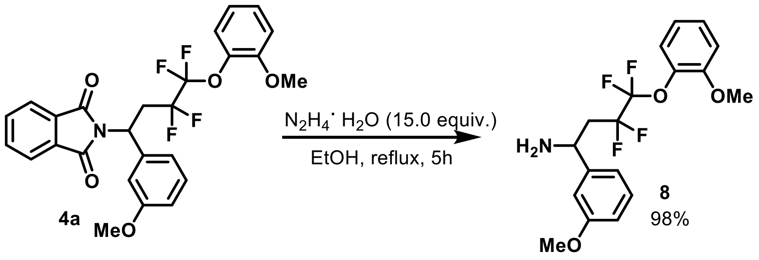

To a stirred solution of 2-(3,3,4,4-tetrafluoro-4-(2-methoxyphenoxy)-1-(3-methoxyphenyl)butyl)isoindoline-1,3-dione **4a** (118.6 mg, 0.24 mmol) in EtOH (0.5 mL) was added hydrazine hydrate (w=65%, 0.27 mL, 15 equiv). The mixture was refluxed for 5 h. After complete, the solvent was evaporated in vacuo and the crude was filtered over Al_2O_3 using chloroform to give the product **8** (89.0 mg, 98% yield) as colorless liquid.

^{1}H NMR (400 MHz, $CDCl_3$) δ = 7.35 – 7.22 (m, 3H), 7.08 – 7.02 (m, 2H), 7.02 – 6.93 (m, 2H), 6.89 – 6.84 (m, 1H), 4.61 (dd, *J* = 7.9, 4.5 Hz, 1H), 3.86 (s, 3H), 3.85 (s, 3H), 2.70 – 2.52 (m, 2H), 1.97 (s, 2H);

^{13}C NMR (100 MHz, $CDCl_3$) δ = 160.0, 152.5, 146.8, 137.8, 129.8, 127.6, 123.9, 120.7 120.5 – 114.4 (m), 118.7, 113.0, 112.9, 112.0, 56.0, 55.3, 50.0, 40.9 (t, *J* = 20.2 Hz);

^{19}F NMR (376 MHz, $CDCl_3$) δ = -88.10 to -89.04 (m, 2F), -113.56 to -117.38 (m, 2F);

IR (film) 3005.03, 2944.75, 2839.64, 1601.39, 1501.83, 1456.87, 1438.51, 1301.99, 1282.40, 1259.47, 1186.54, 1169.13, 1103.31, 1041.93, 1024.53, 968.94, 853.37, 780.25, 746.98, 699.06, 555.80 cm^{-1};

HRMS (+ESI) calcd for $C_{18}H_{20}F_4NO_3[M+H]^+$ m/z = 374.1374; found: 374.1367.

7. Spectral Data

Compound 1A. Top: ^{1}H NMR ($CDCl_3$, 400 MHz). Bottom: ^{13}C NMR ($CDCl_3$, 100 MHz).

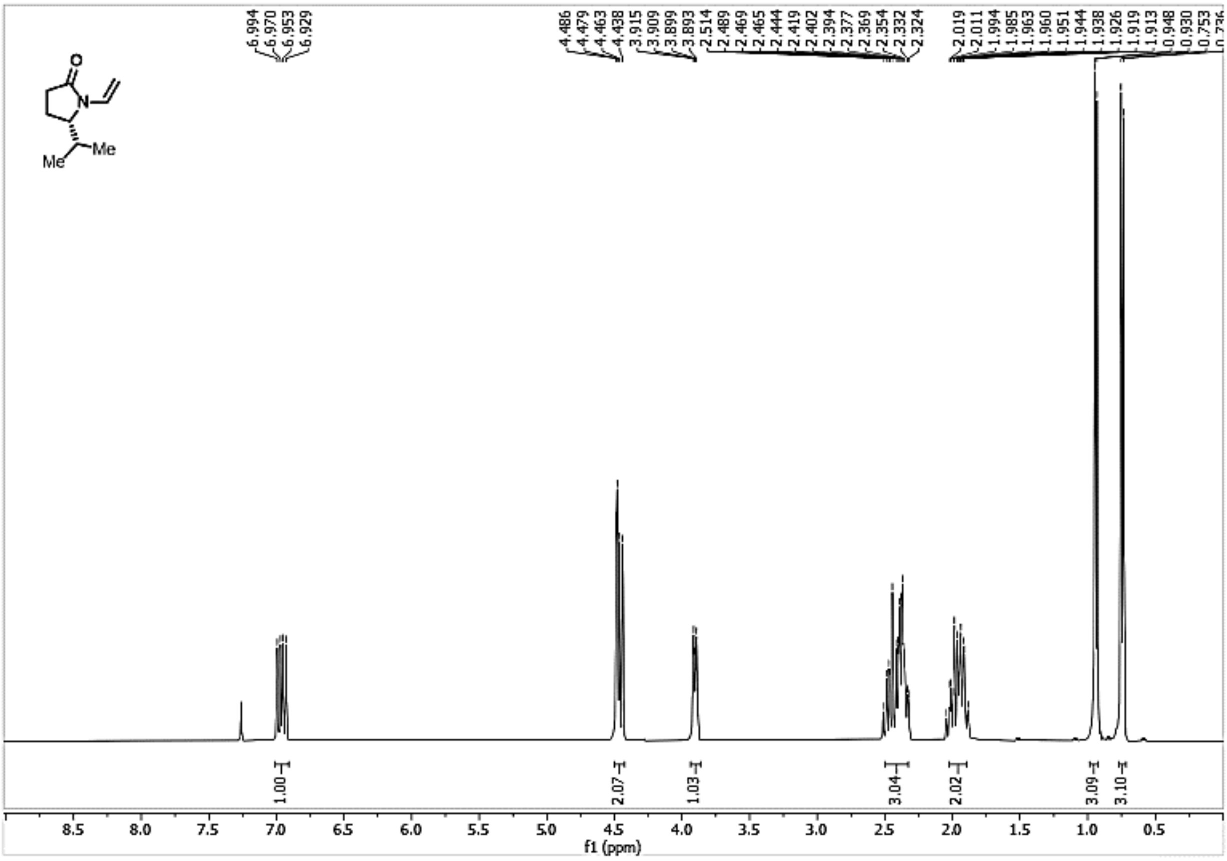

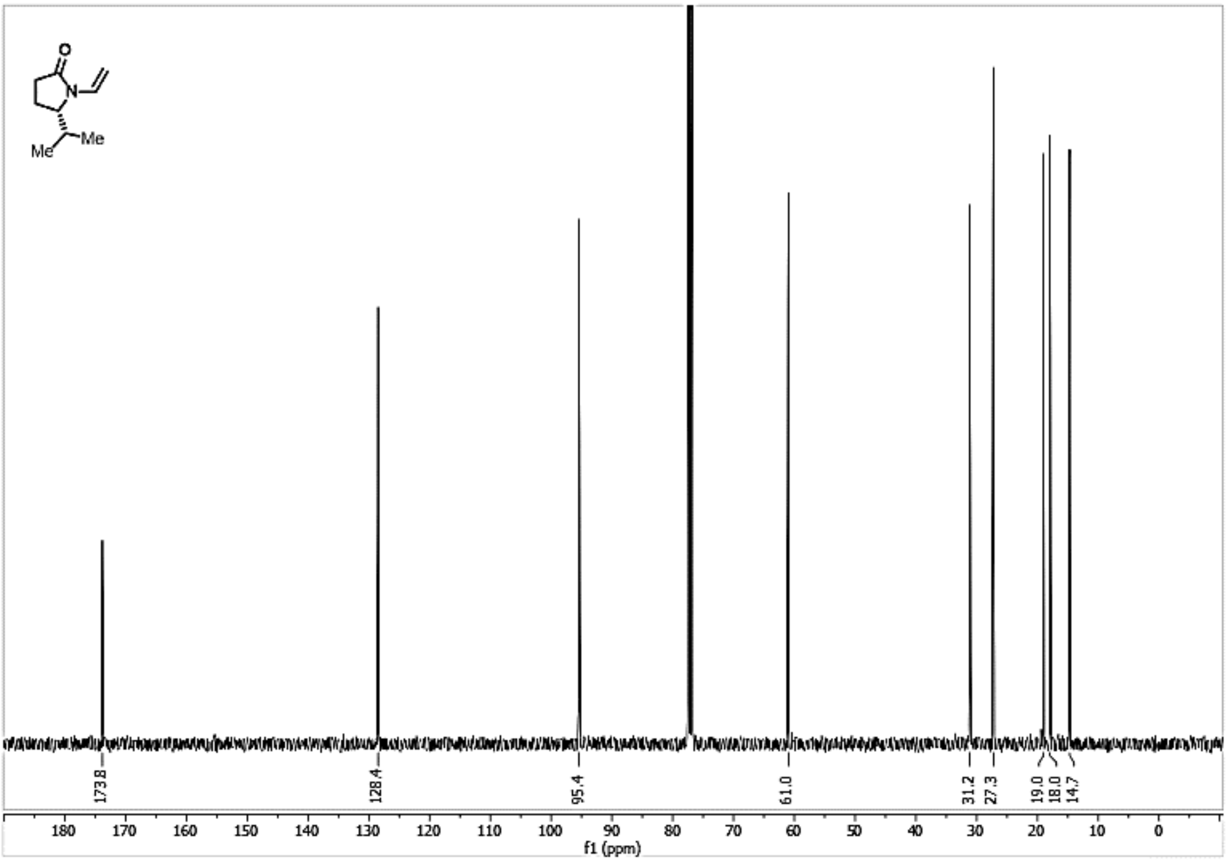

Compound 1B. Top: ^{1}H NMR ($CDCl_3$, 400 MHz). Bottom: ^{13}C NMR ($CDCl_3$, 100 MHz).

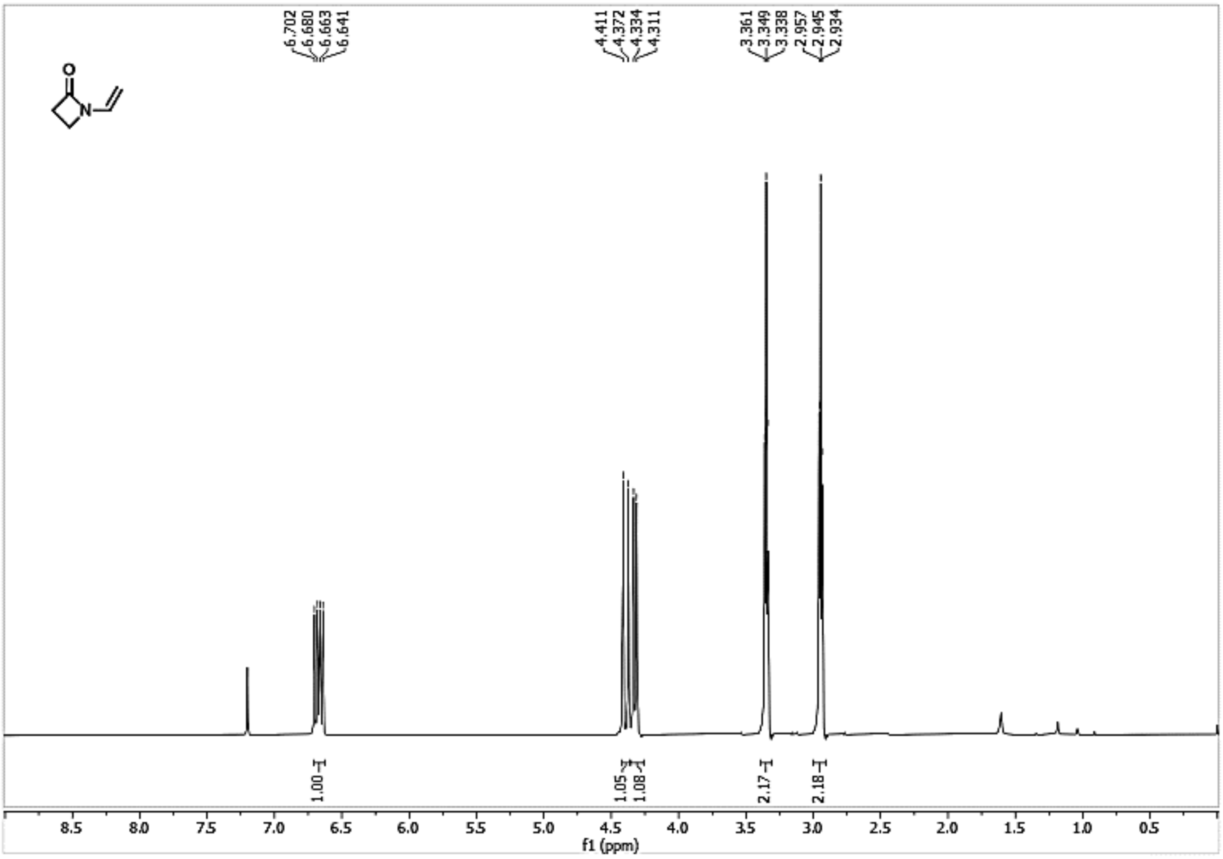

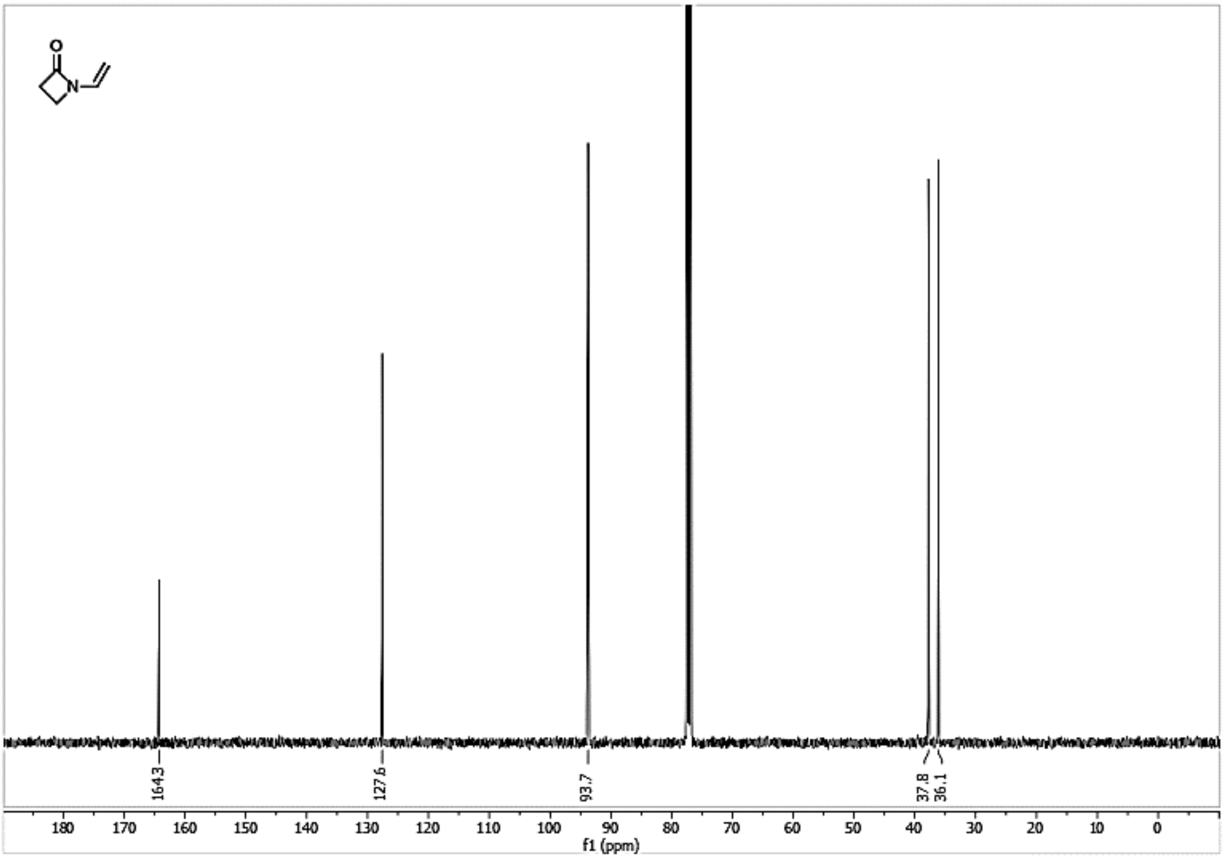

Compound 1C. Top: ^{1}H NMR ($CDCl_3$, 400 MHz). Bottom: ^{13}C NMR ($CDCl_3$, 100 MHz).

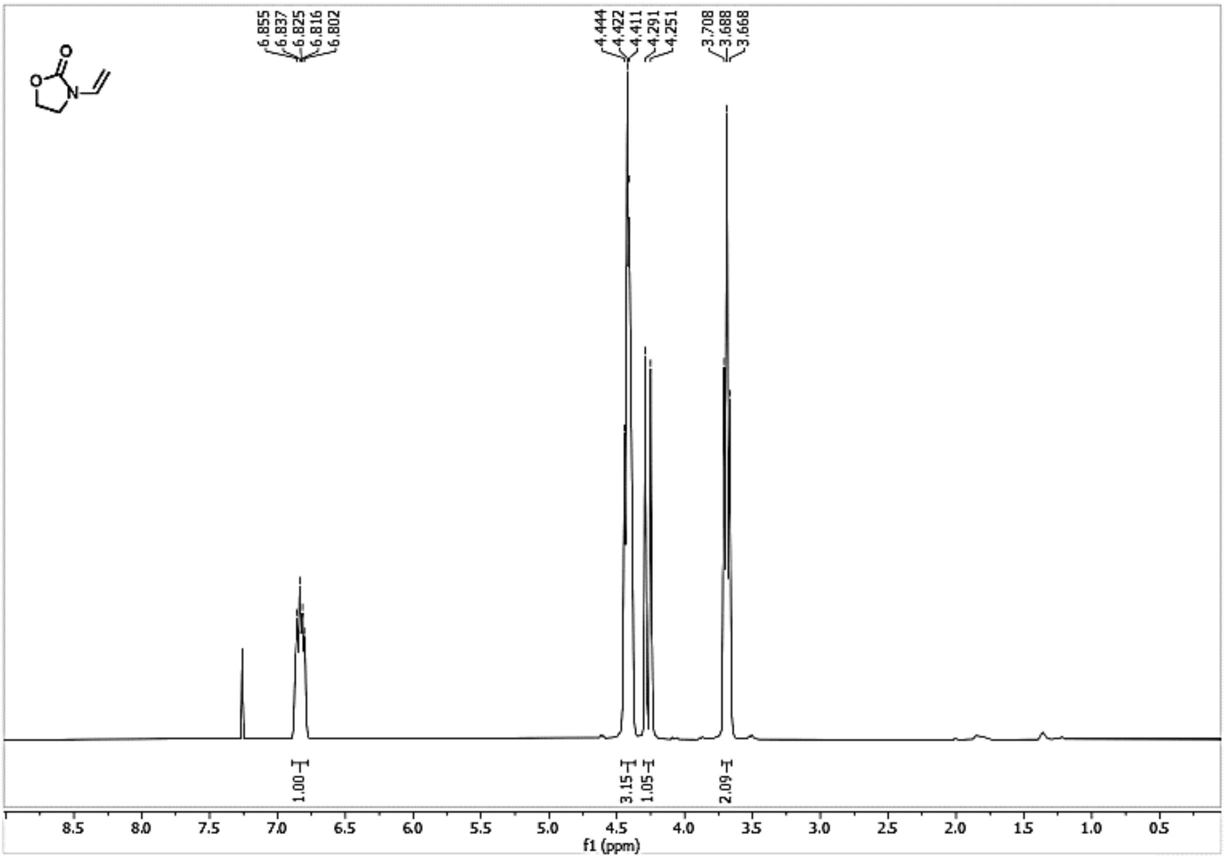

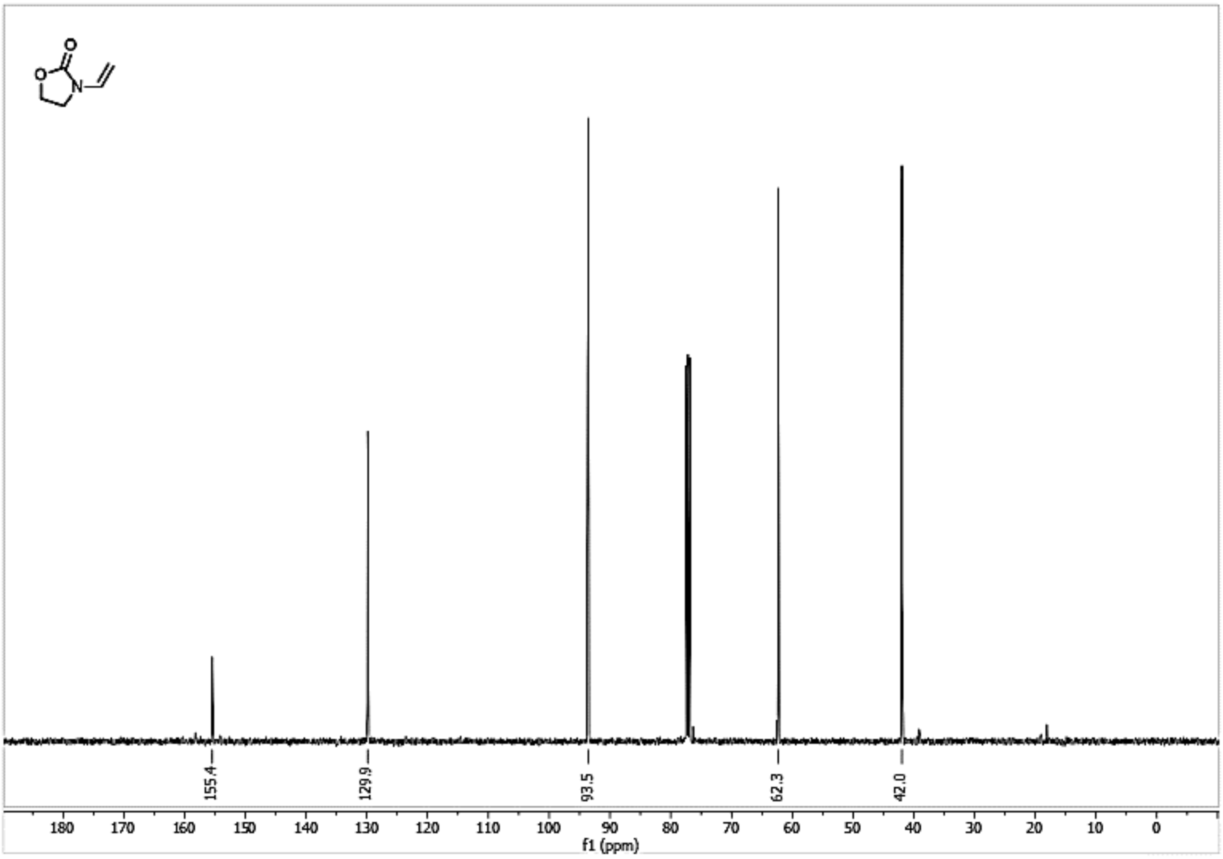

Compound 4a. Top: ^{1}H NMR ($CDCl_3$, 400 MHz). Bottom: ^{13}C NMR ($CDCl_3$, 100 MHz).

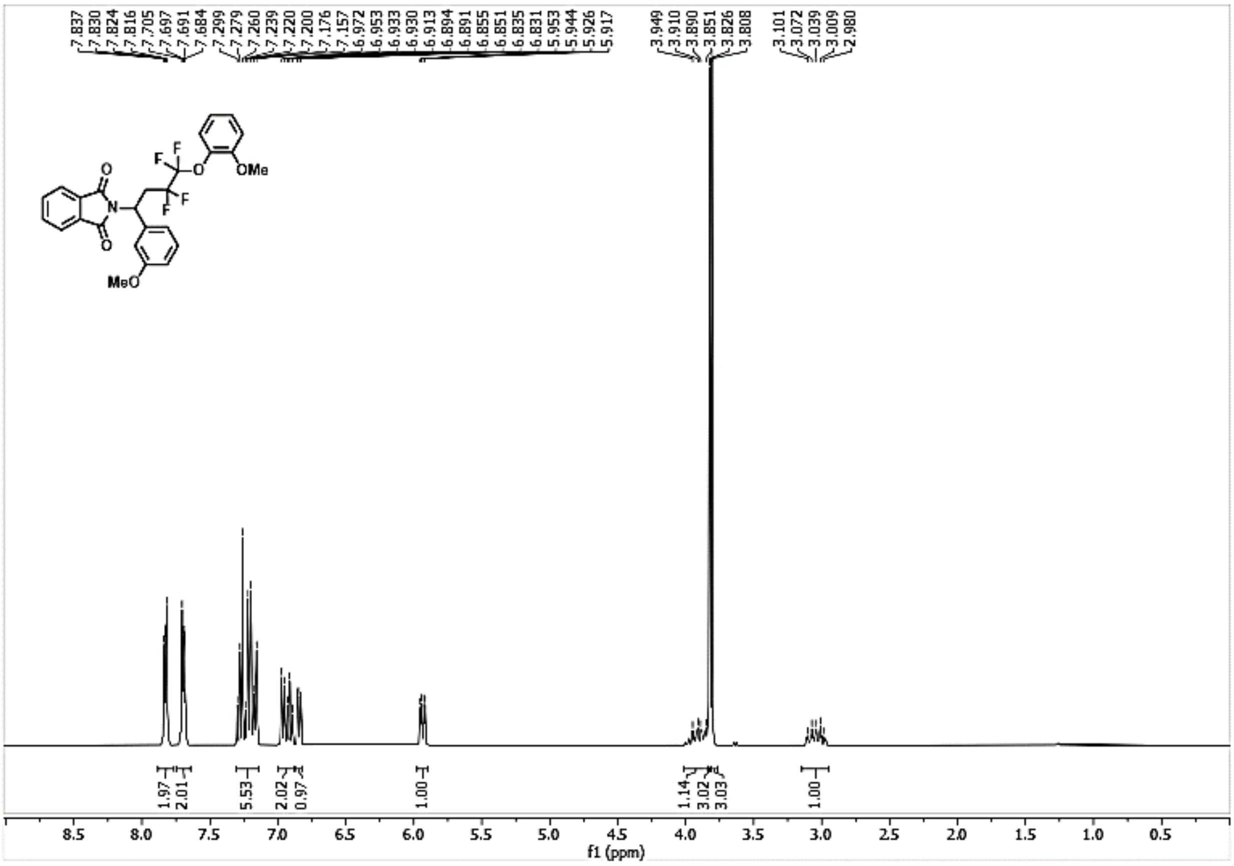

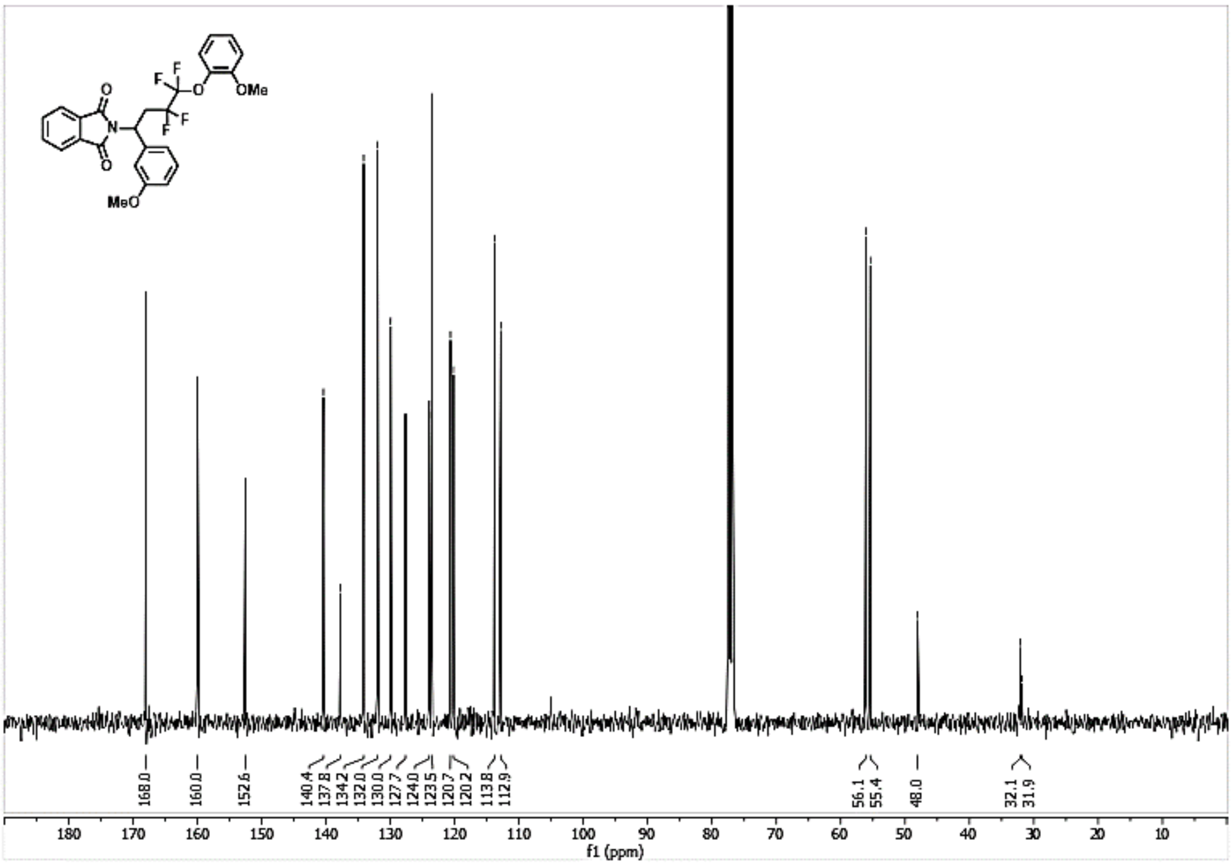

Compound 4a. ^{19}F NMR ($CDCl_3$, 376 MHz).

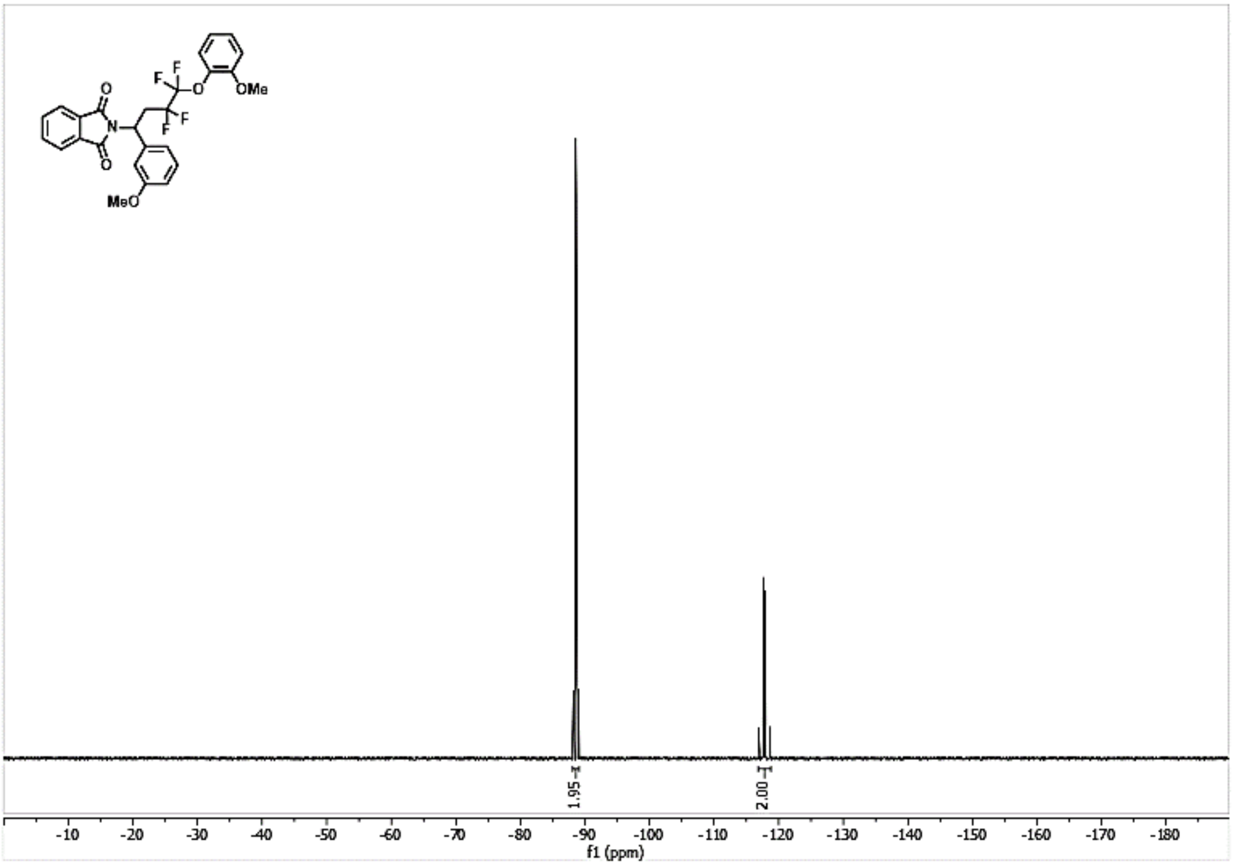

Compound 4b. Top: ^{1}H NMR ($CDCl_3$, 400 MHz). Bottom: ^{13}C NMR ($CDCl_3$, 100 MHz).

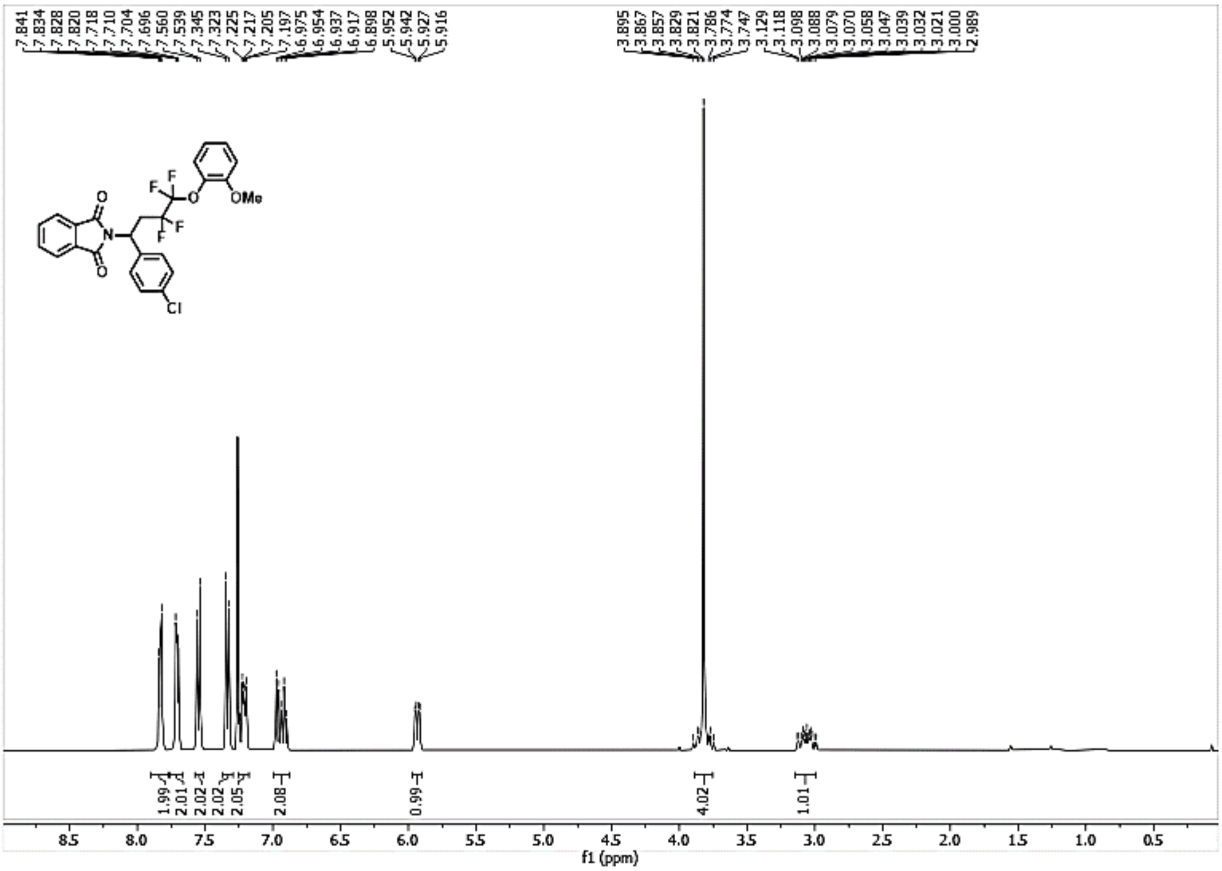

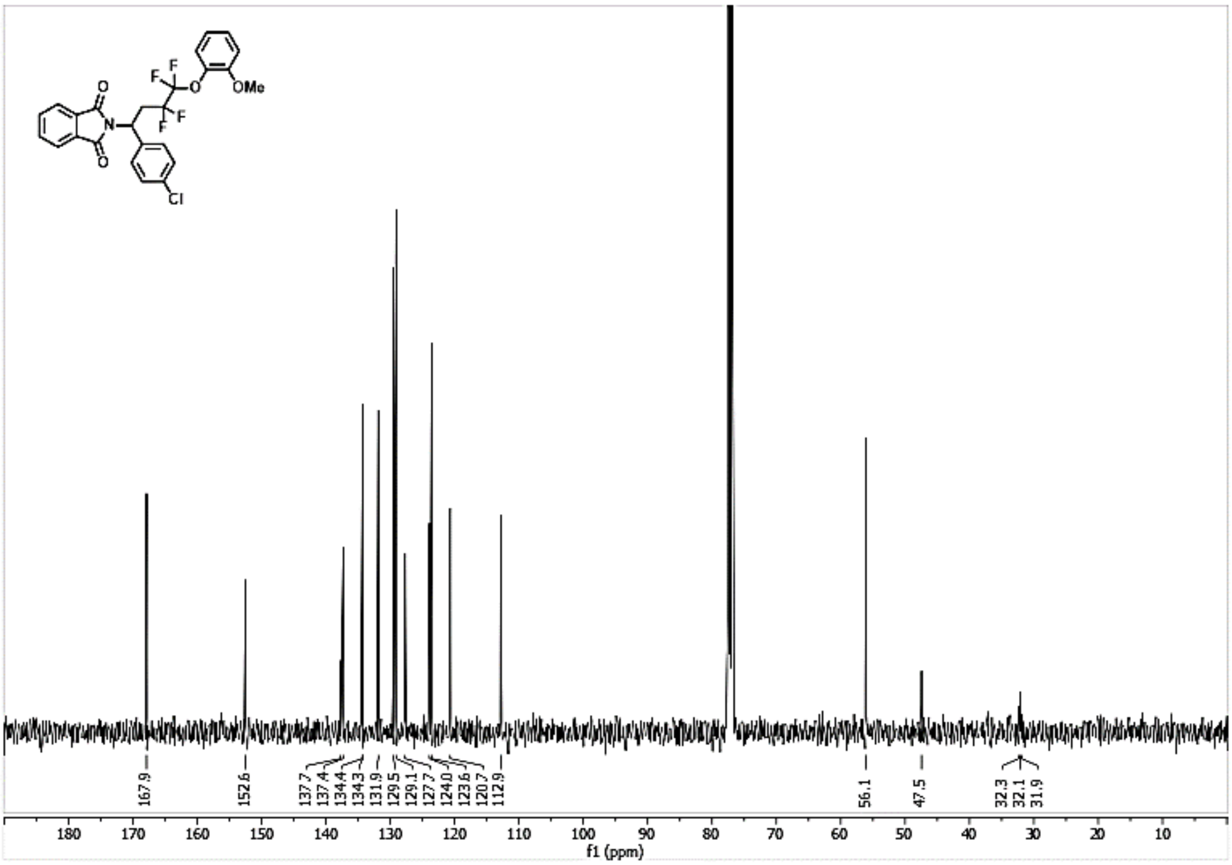

Compound 4b. ^{19}F NMR ($CDCl_3$, 376 MHz).

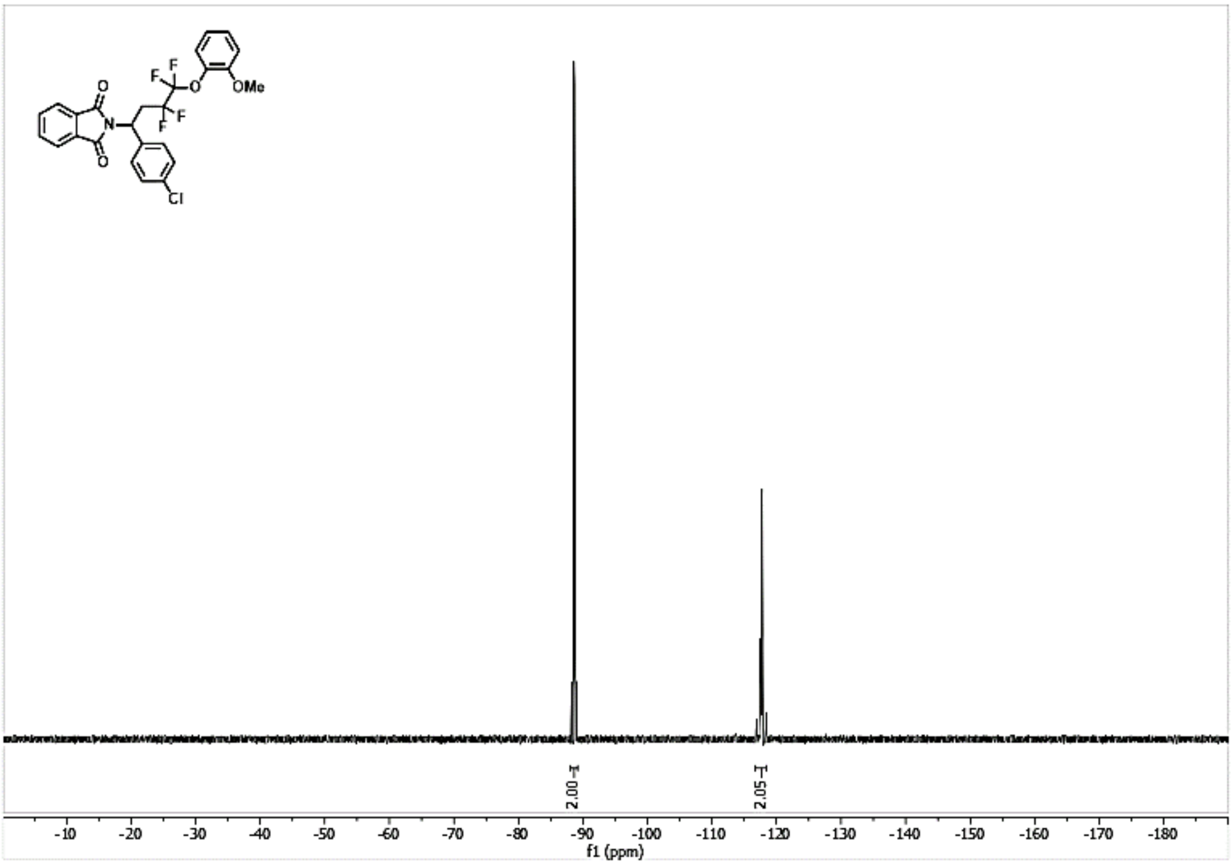

Compound 4c. Top: ^{1}H NMR ($CDCl_3$, 400 MHz). Bottom: ^{13}C NMR ($CDCl_3$, 100 MHz).

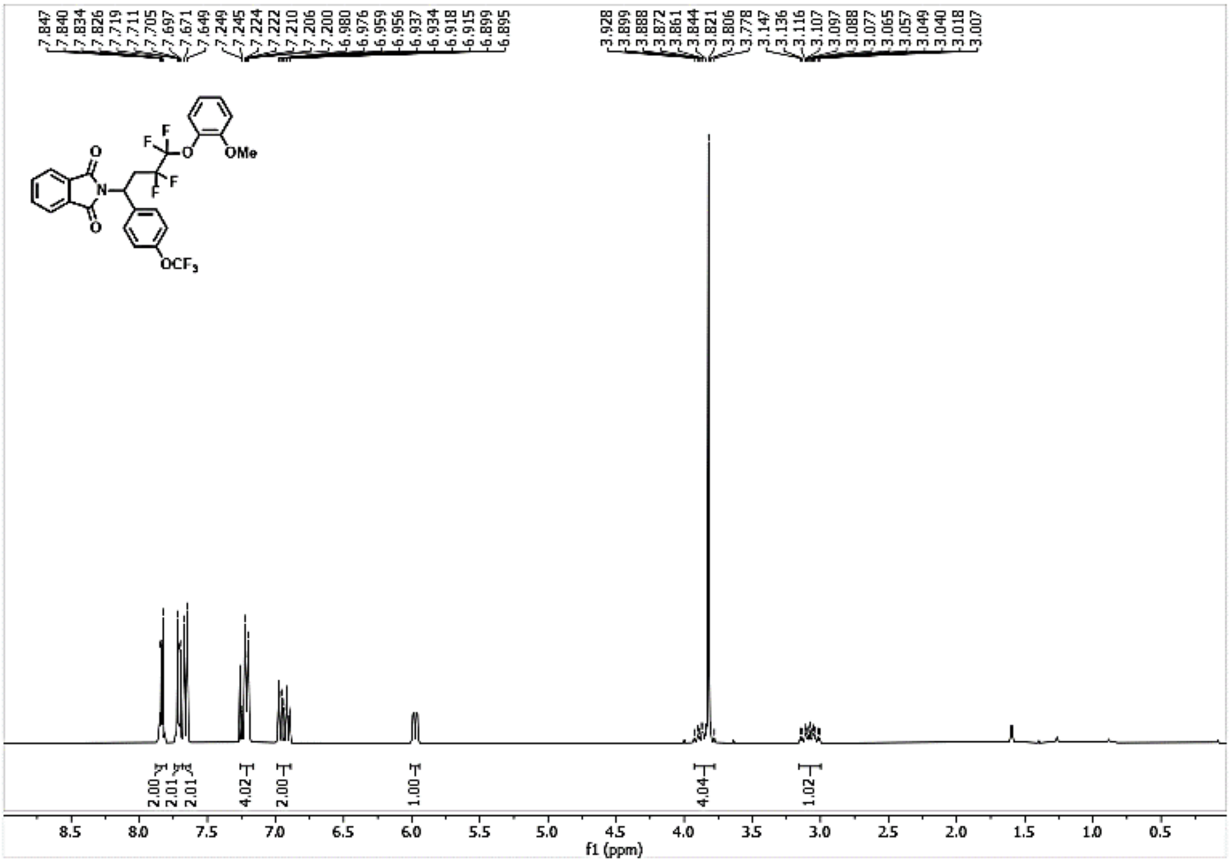

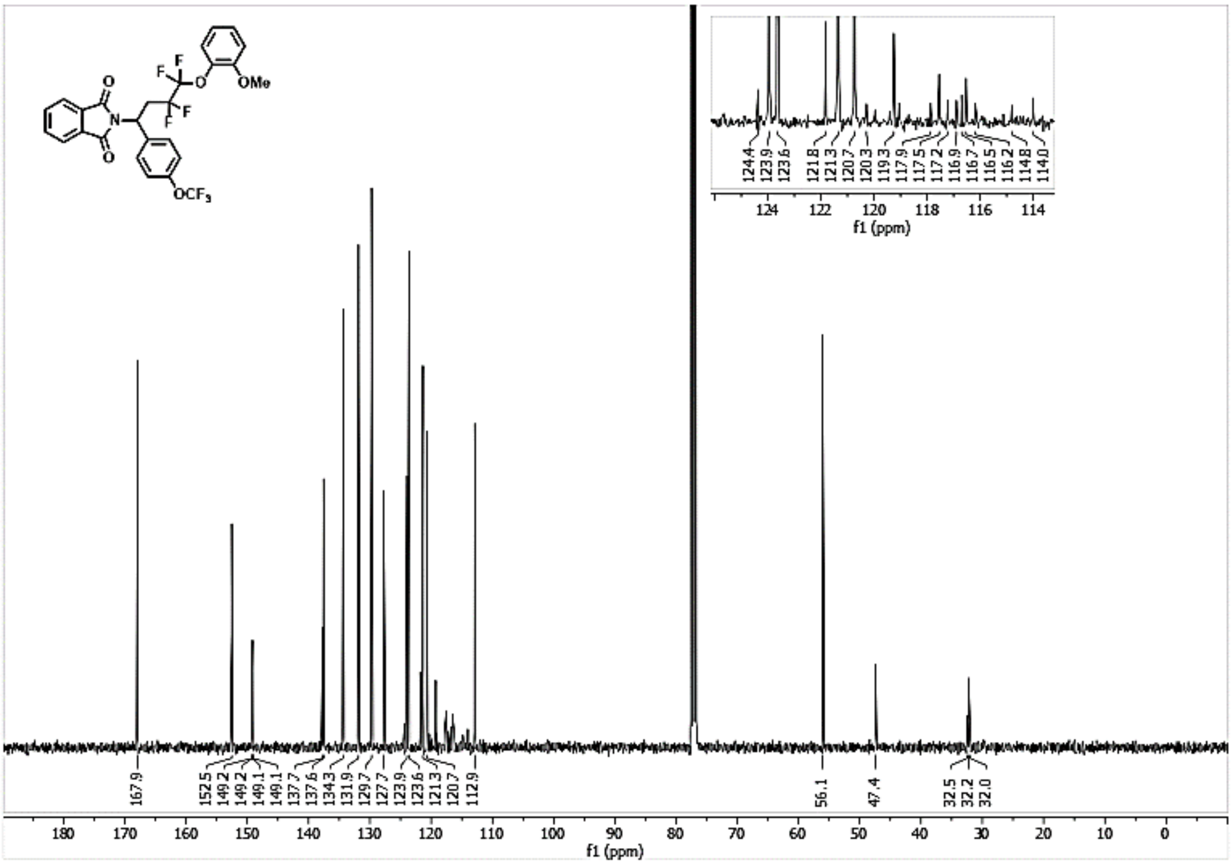

Compound 4c. ^{19}F NMR ($CDCl_3$, 376 MHz).

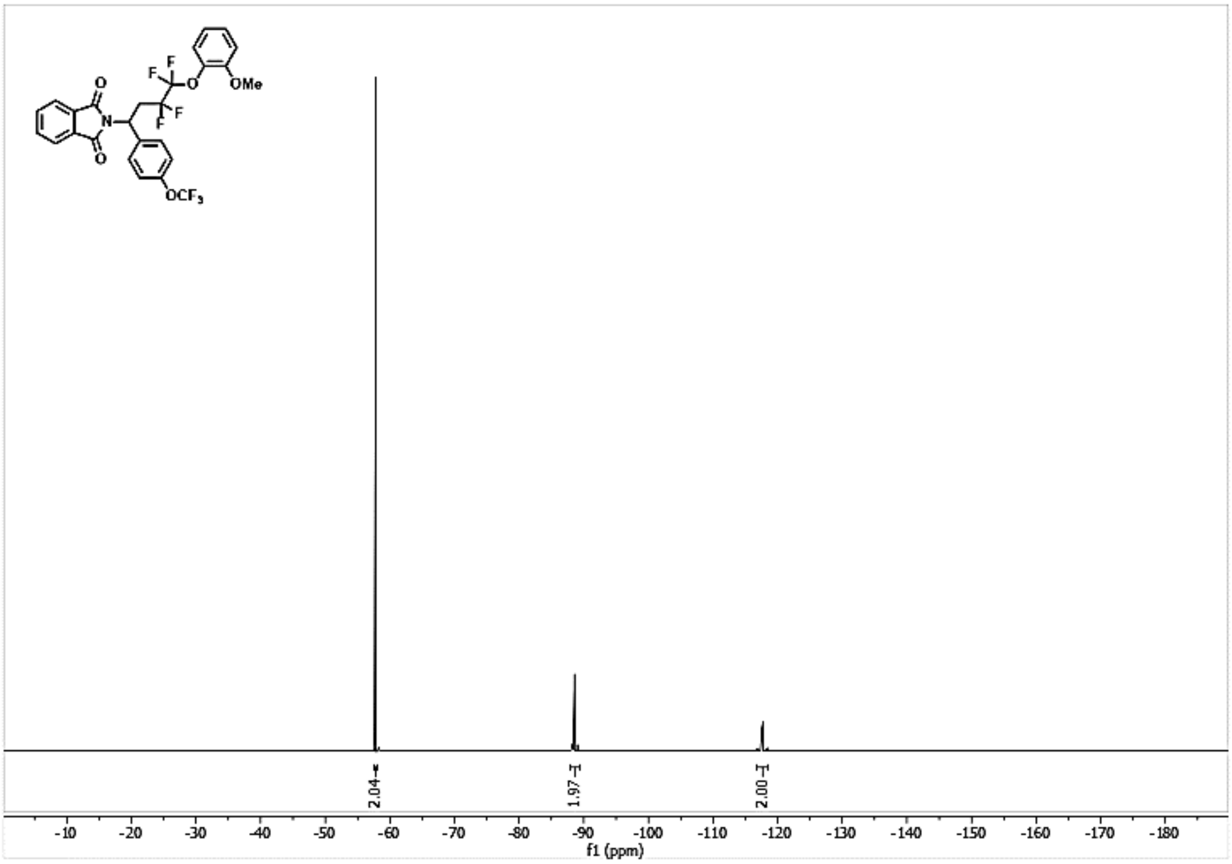

Compound 4d. Top: ^{1}H NMR ($CDCl_3$, 400 MHz). Bottom: ^{13}C NMR ($CDCl_3$, 100 MHz).

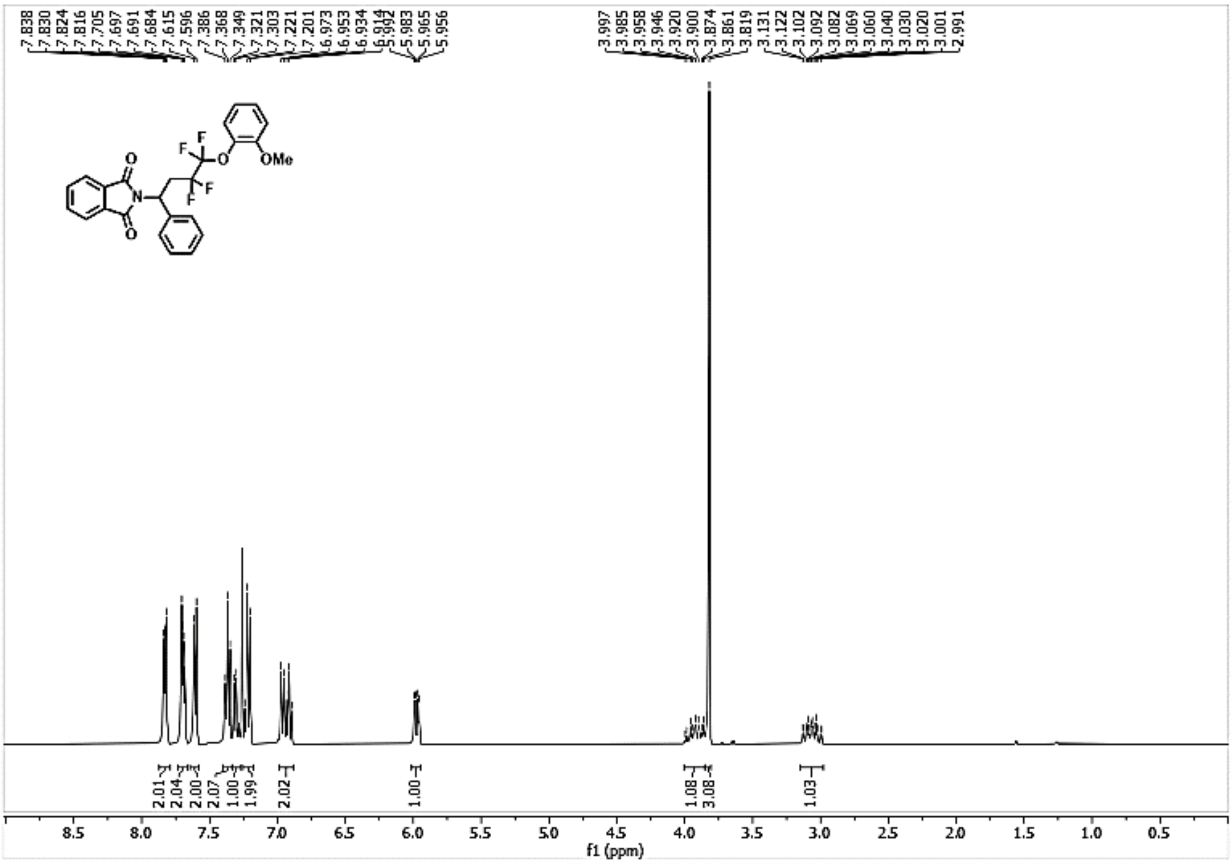

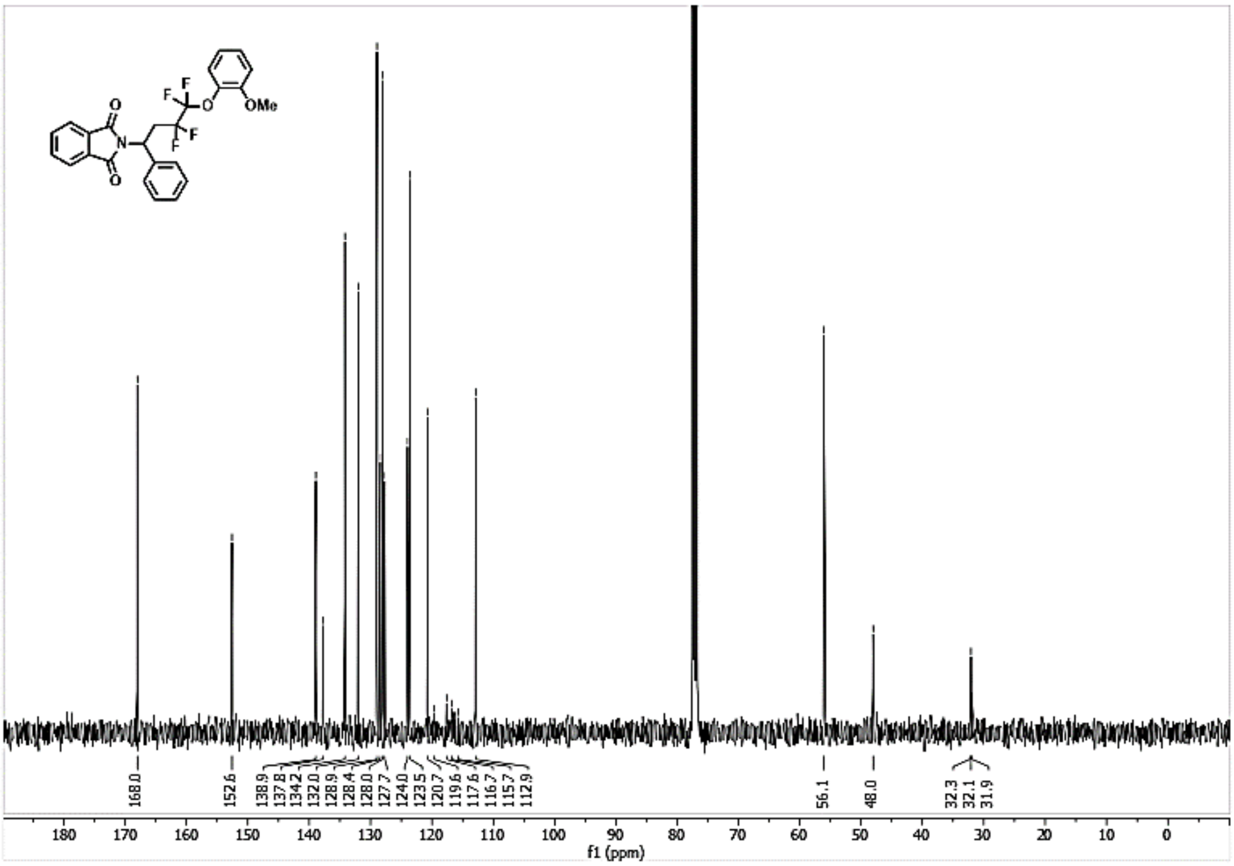

Compound 4d. ^{19}F NMR ($CDCl_3$, 376 MHz).

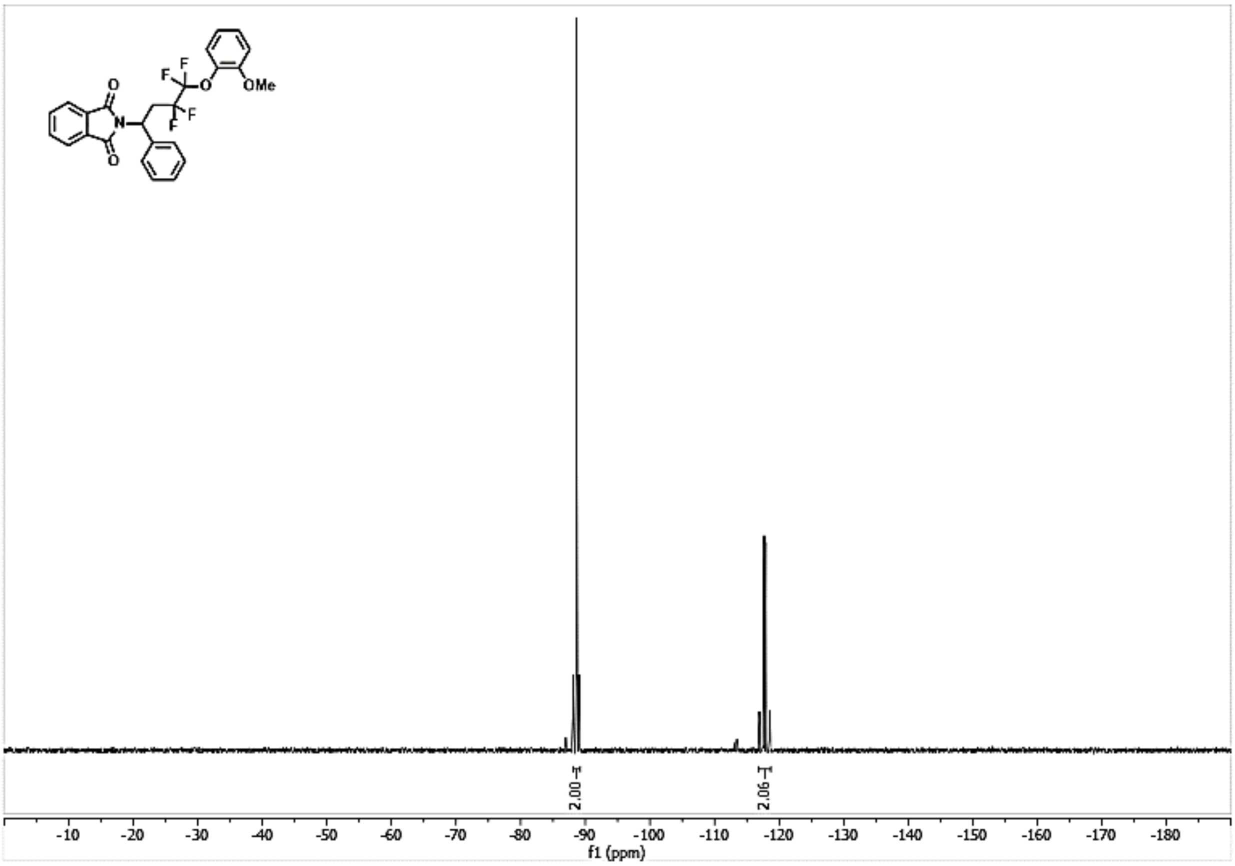

Compound 4e. Top: ^{1}H NMR ($CDCl_3$, 400 MHz). Bottom: ^{13}C NMR ($CDCl_3$, 100 MHz).

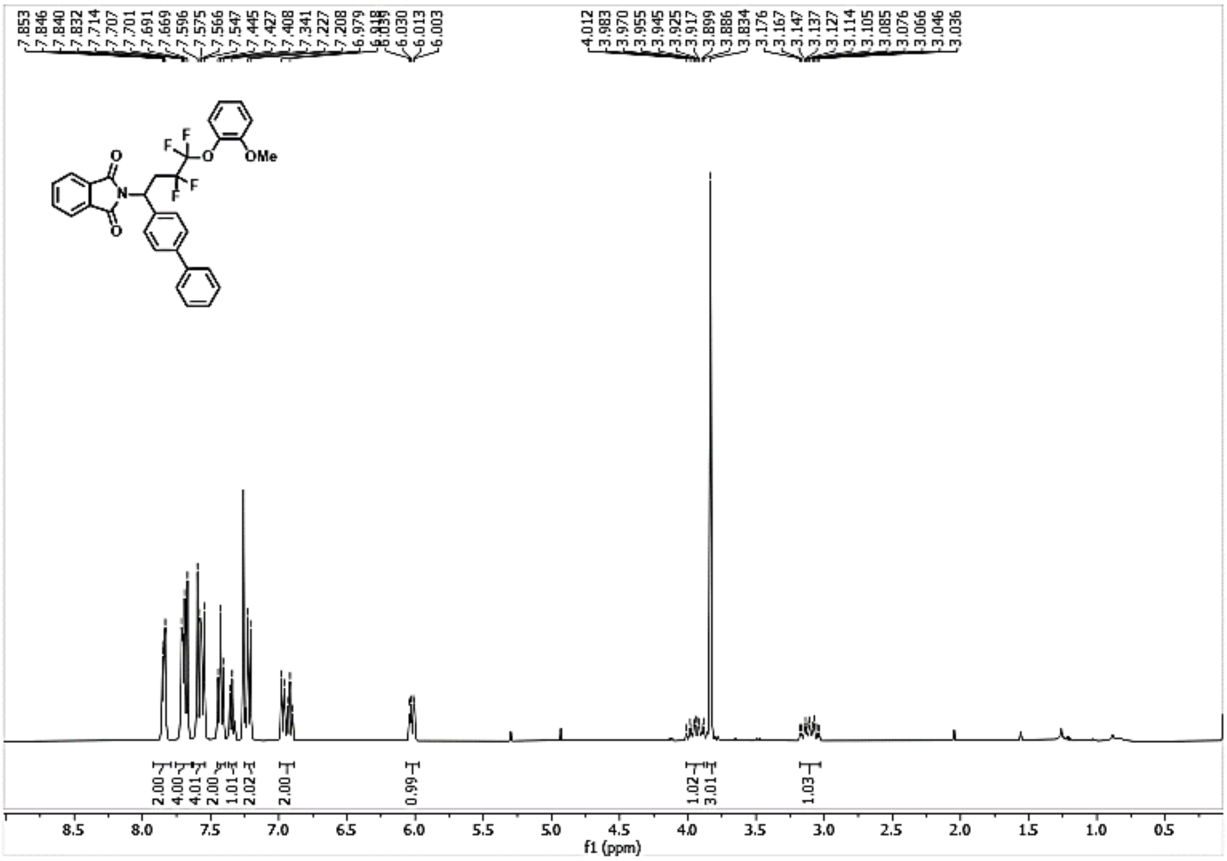

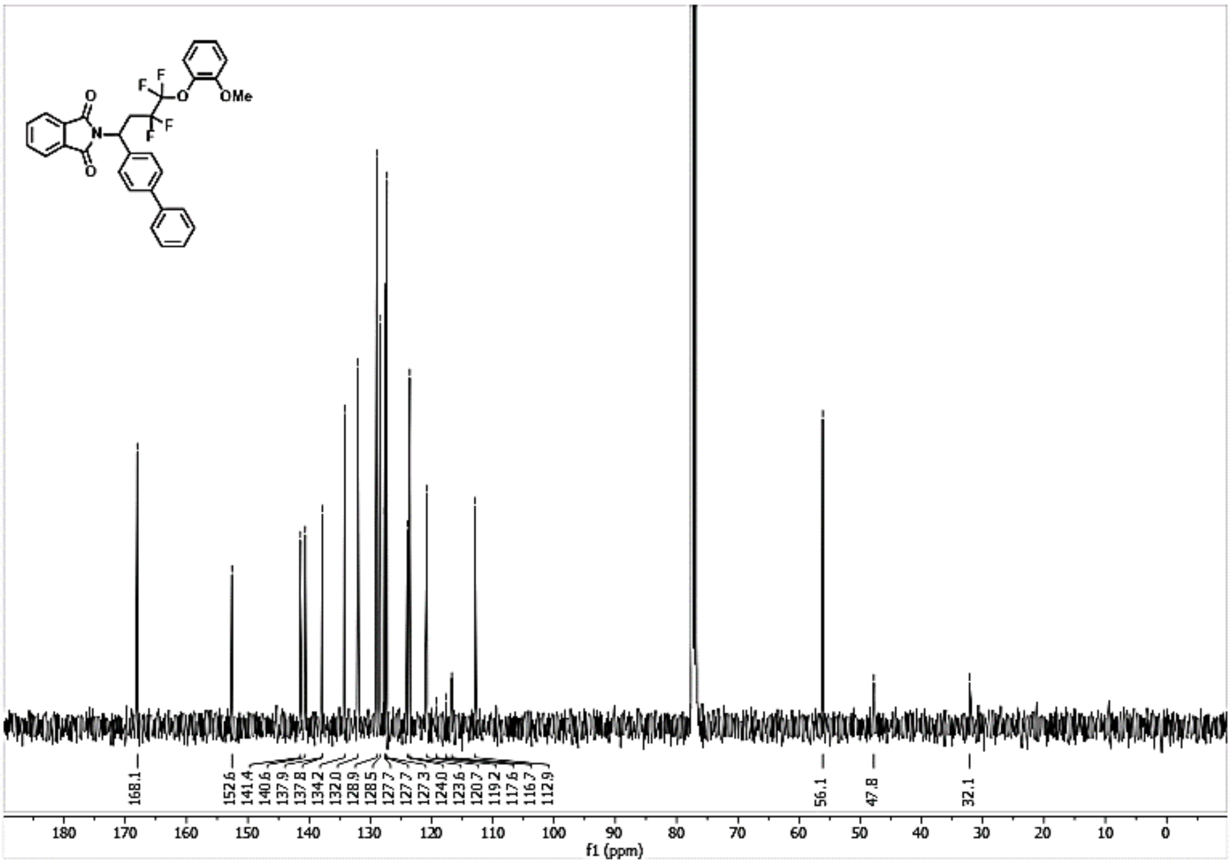

Compound 4e. ^{19}F NMR ($CDCl_3$, 376 MHz).

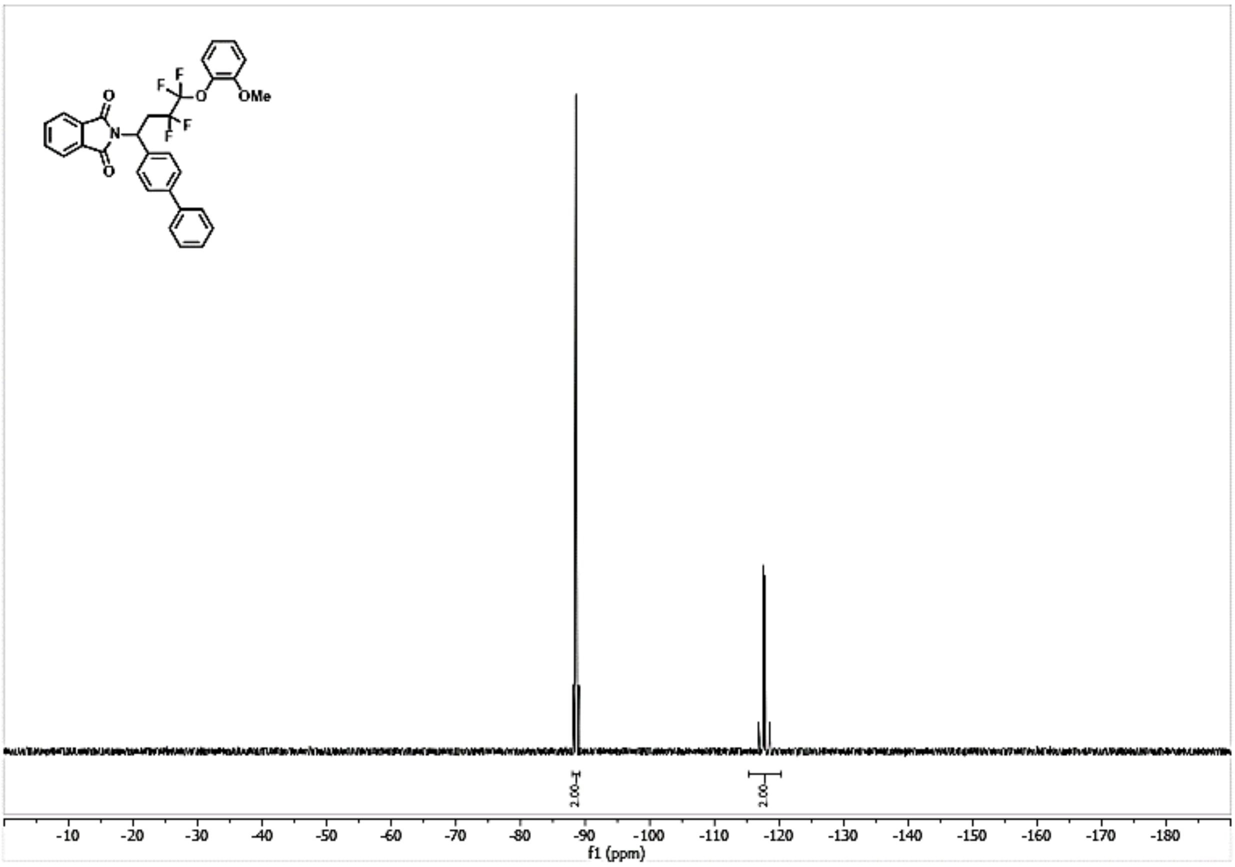

Compound 4f. Top: ^{1}H NMR ($CDCl_3$, 400 MHz). Bottom: ^{13}C NMR ($CDCl_3$, 100 MHz).

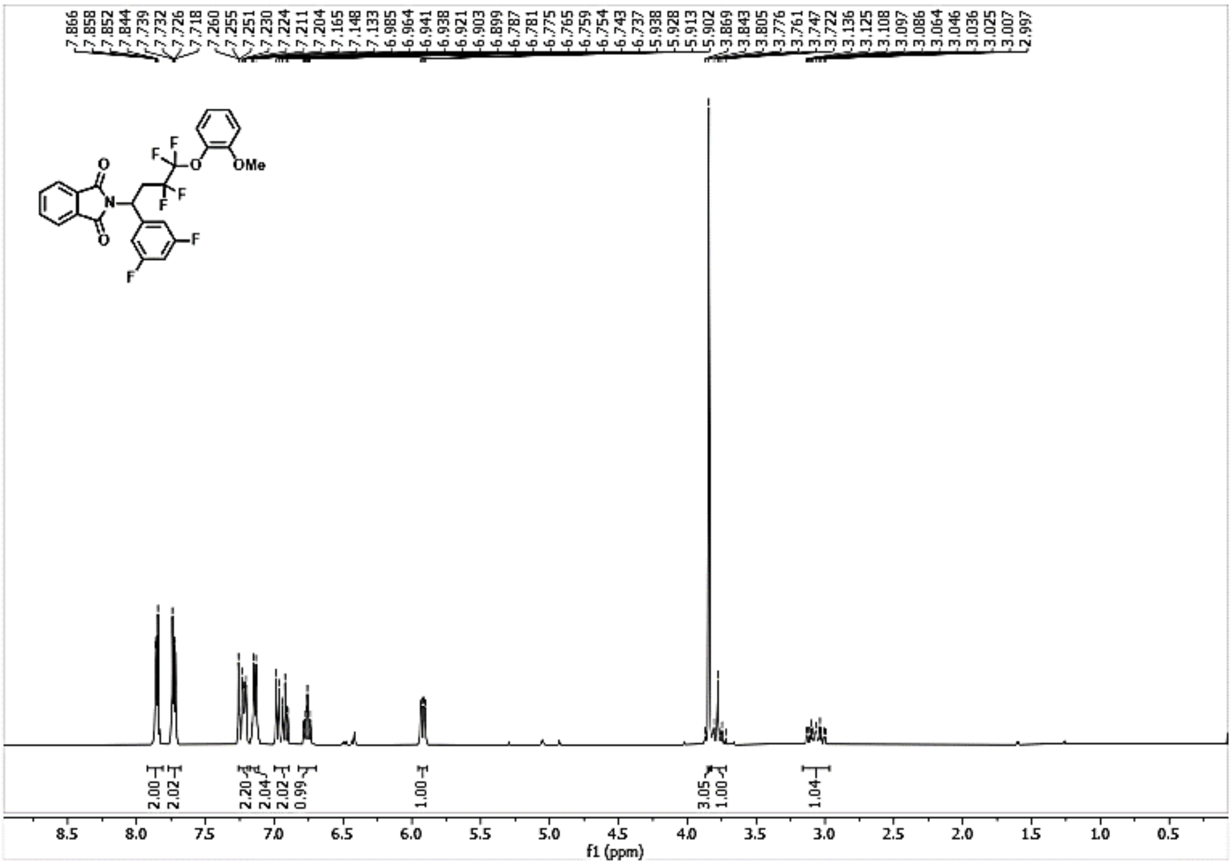

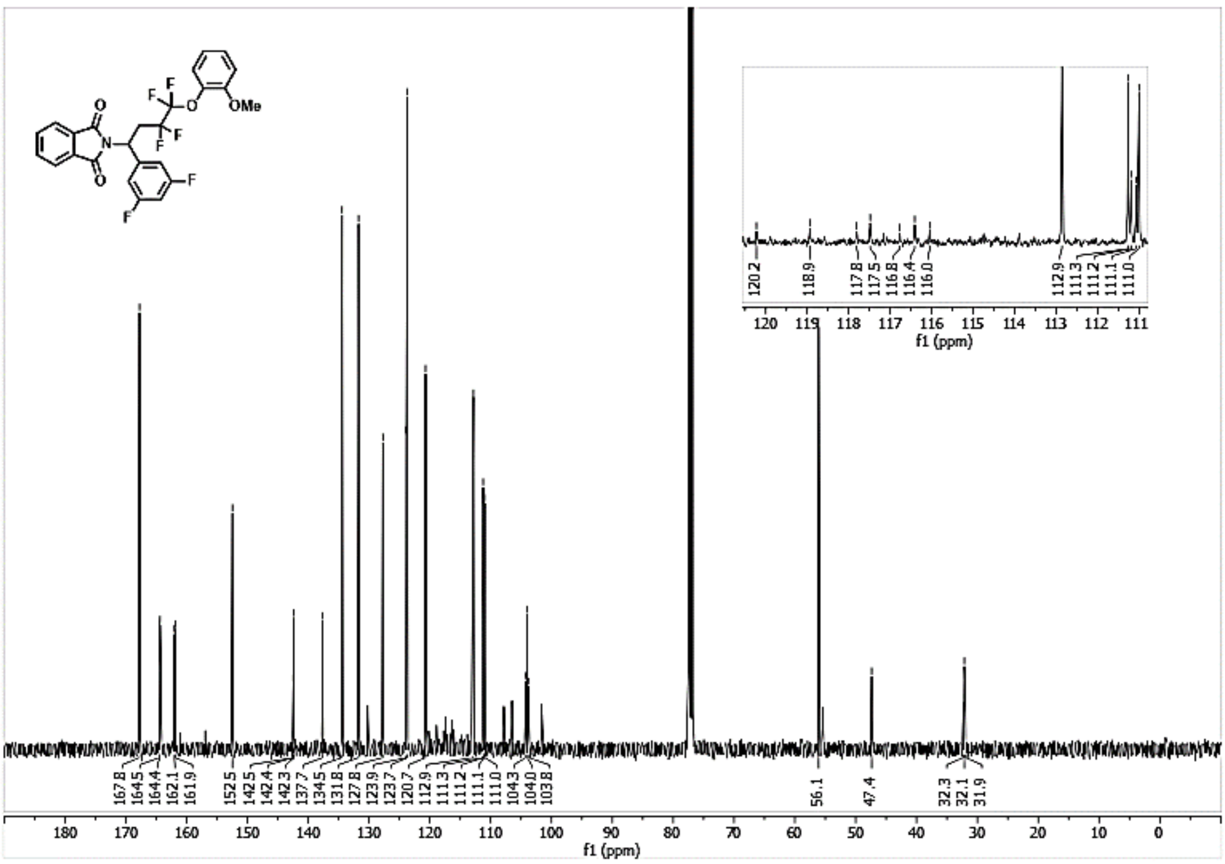

Compound 4f. ^{19}F NMR ($CDCl_3$, 376 MHz).

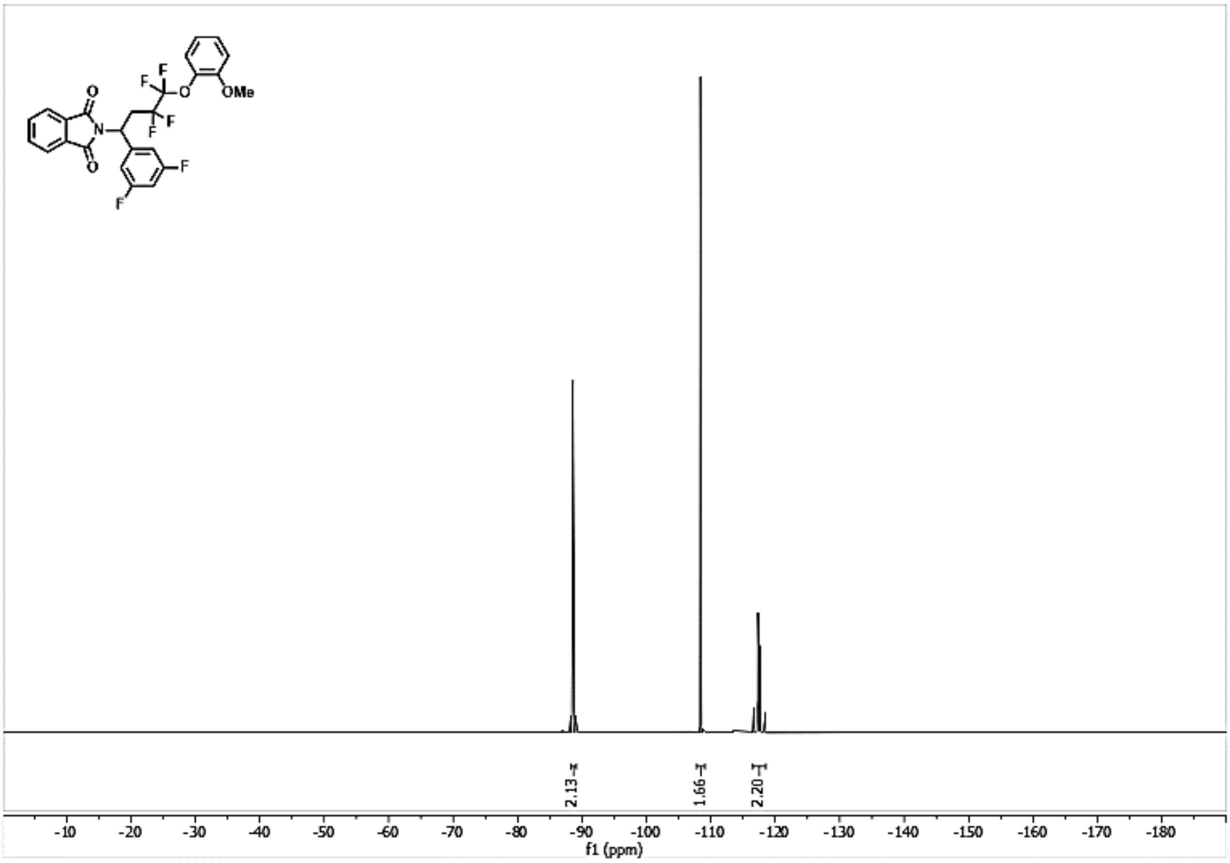

Compound 4g. Top: ^{1}H NMR ($CDCl_3$, 400 MHz). Bottom: ^{13}C NMR ($CDCl_3$, 100 MHz).

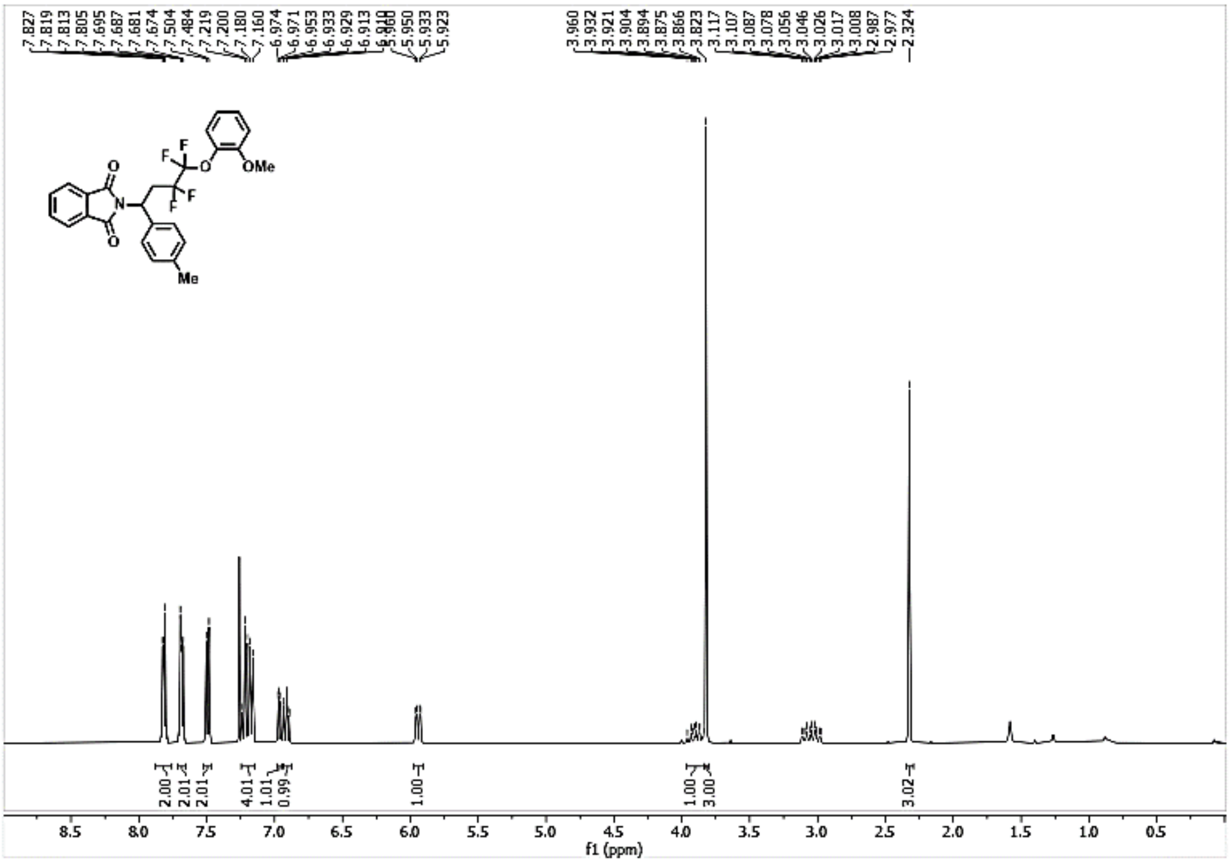

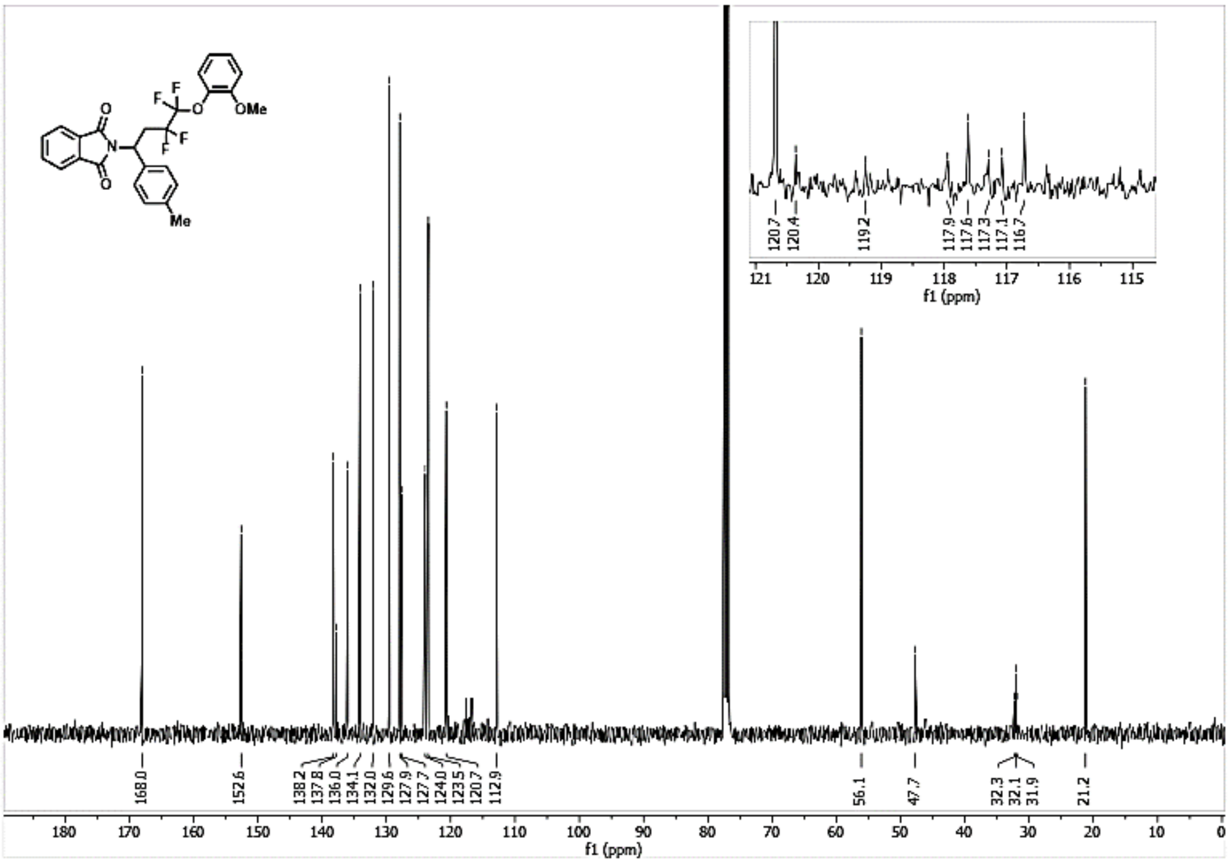

Compound 4g. ^{19}F NMR ($CDCl_3$, 376 MHz).

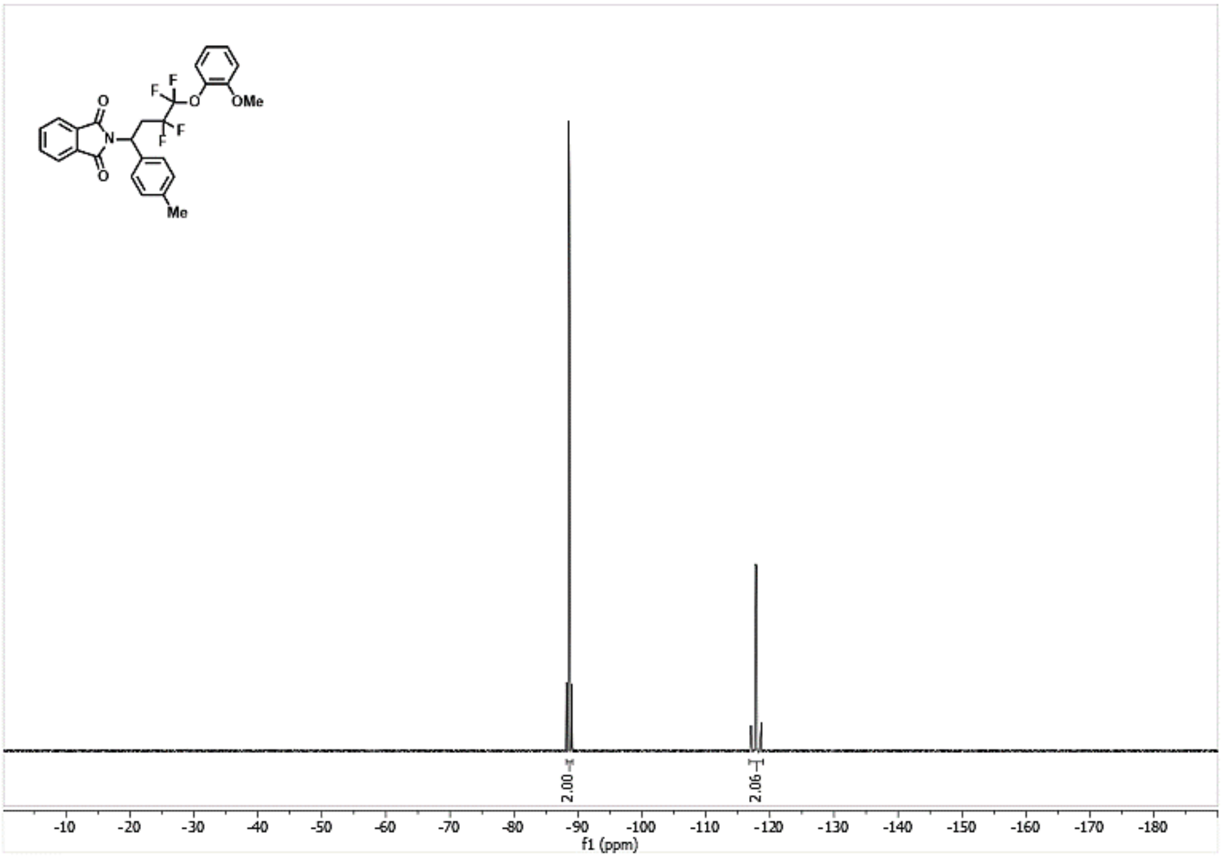

Compound 4h. Top: ^{1}H NMR ($CDCl_3$, 400 MHz). Bottom: ^{13}C NMR ($CDCl_3$, 100 MHz).

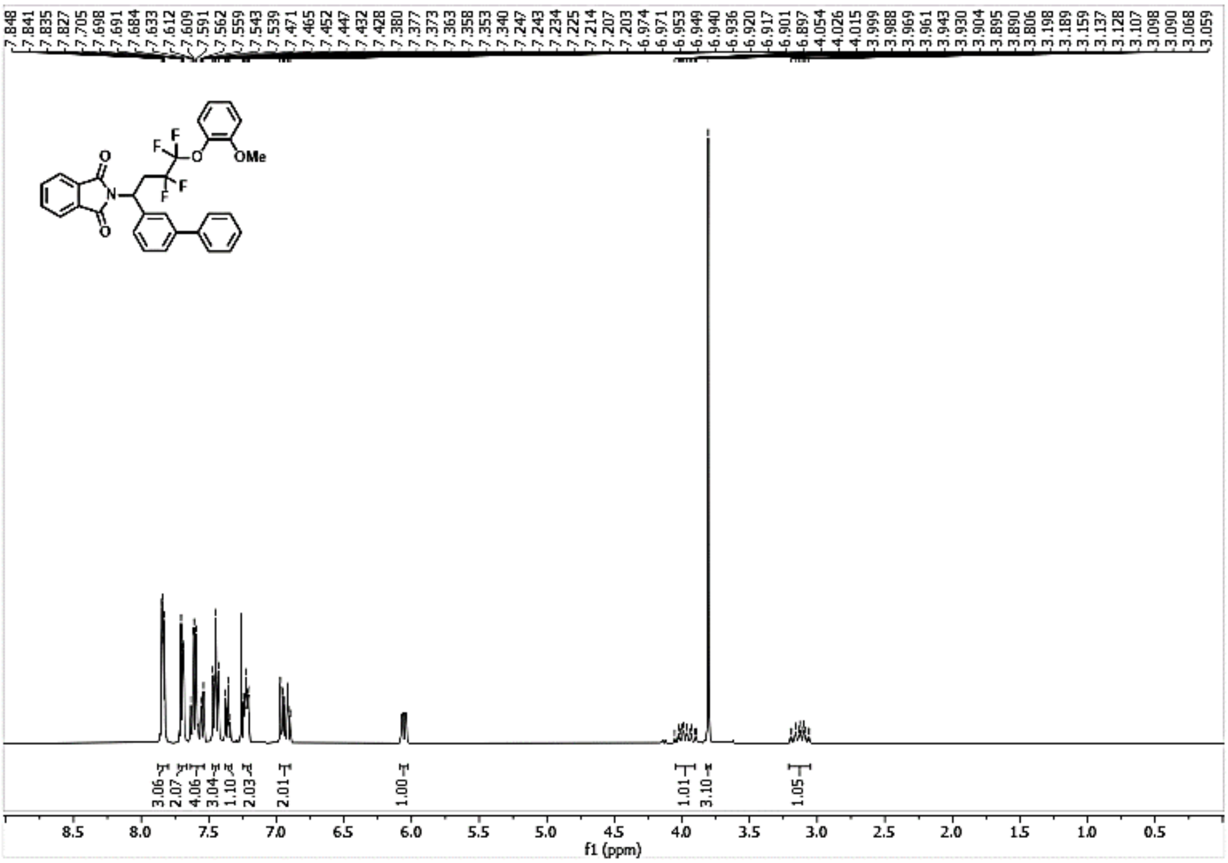

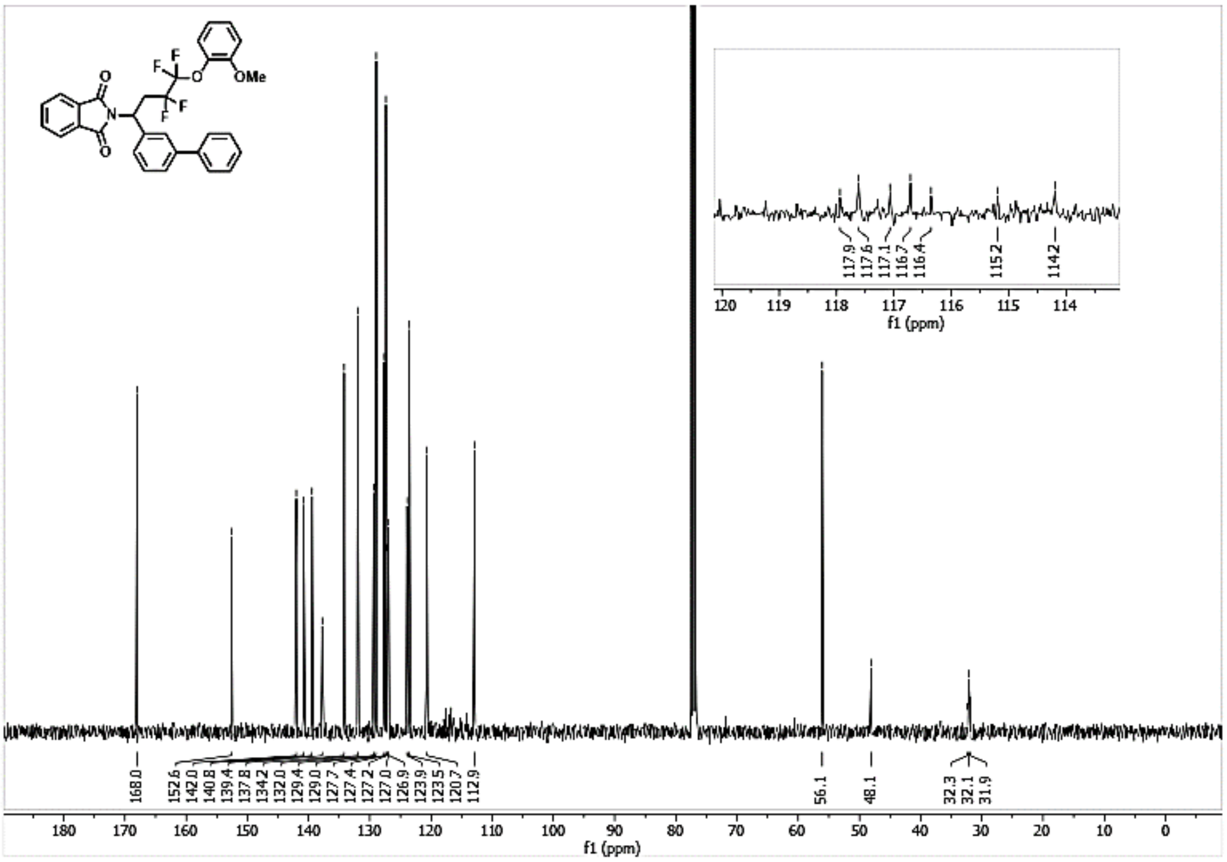

Compound 4h. ^{19}F NMR ($CDCl_3$, 376 MHz).

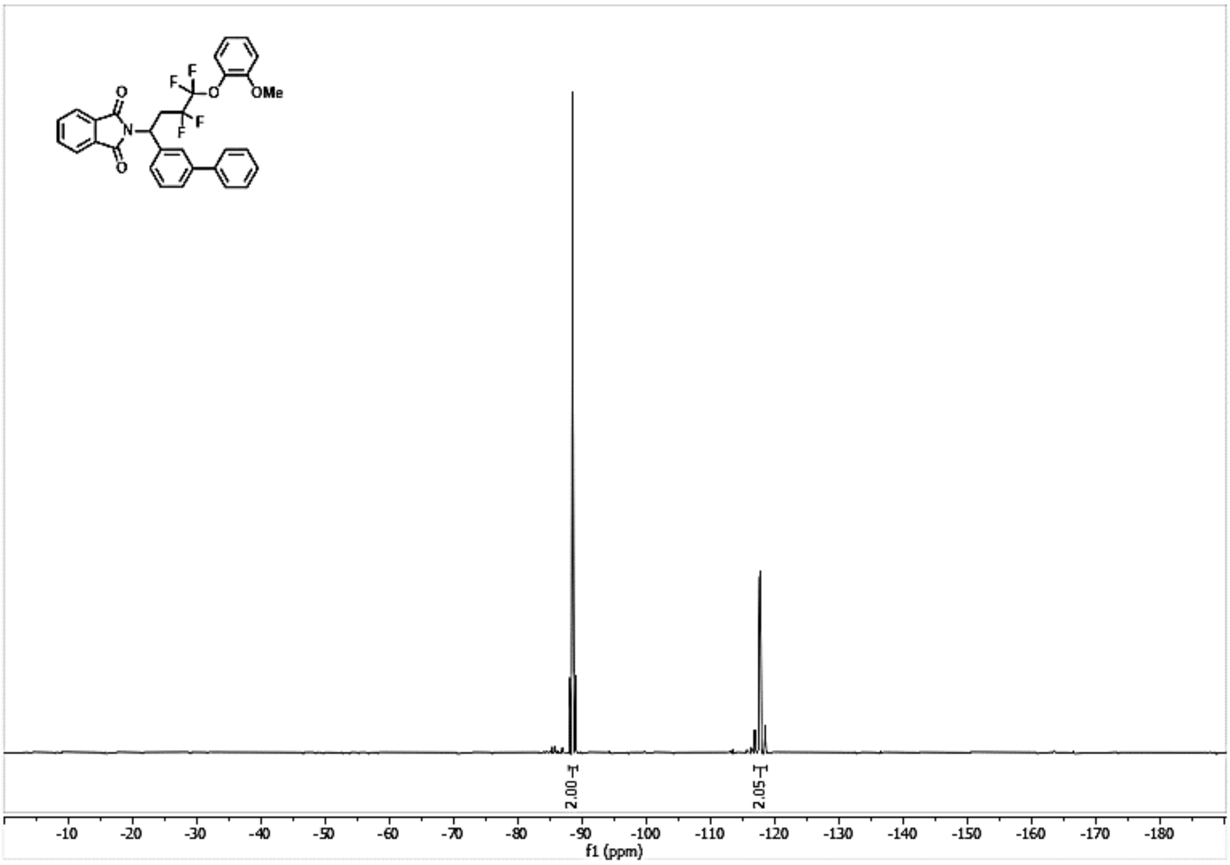

Compound 4i. Top: ^{1}H NMR ($CDCl_3$, 400 MHz). Bottom: ^{13}C NMR ($CDCl_3$, 100 MHz).

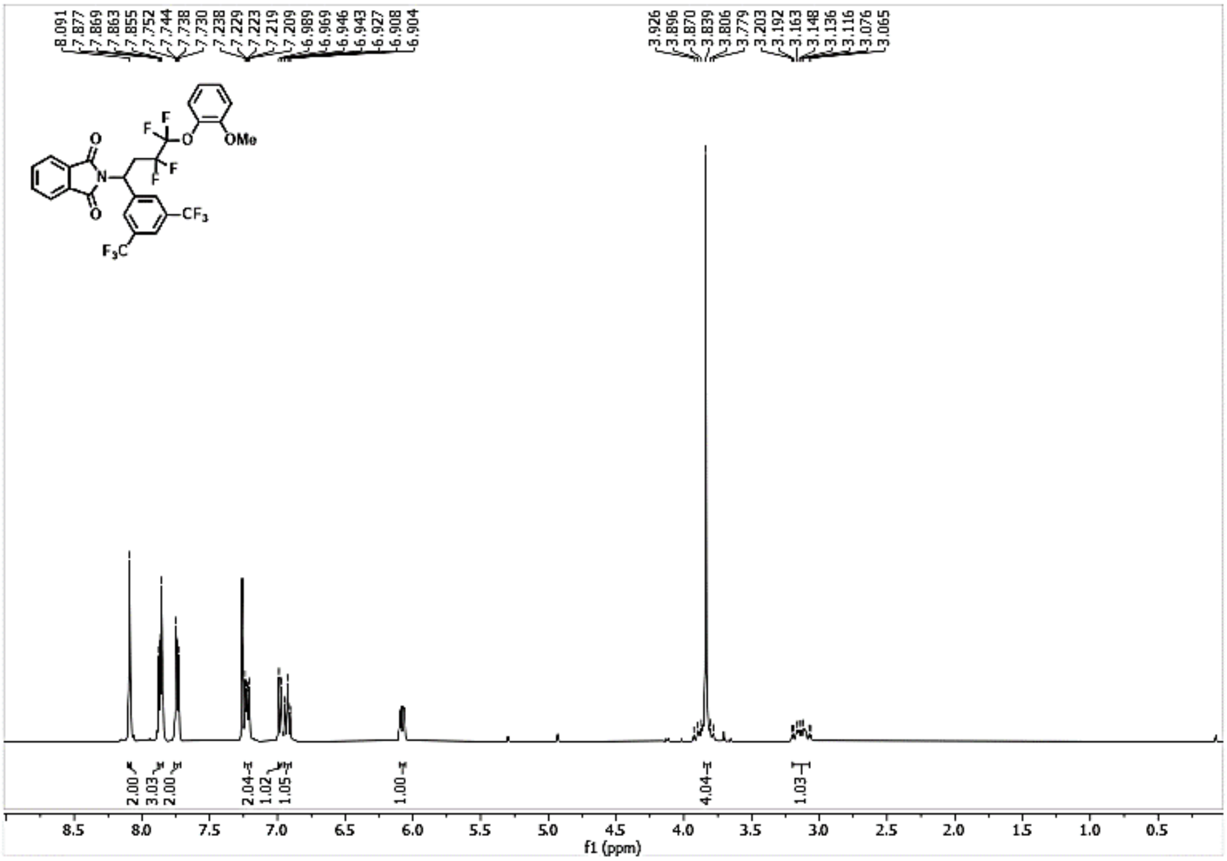

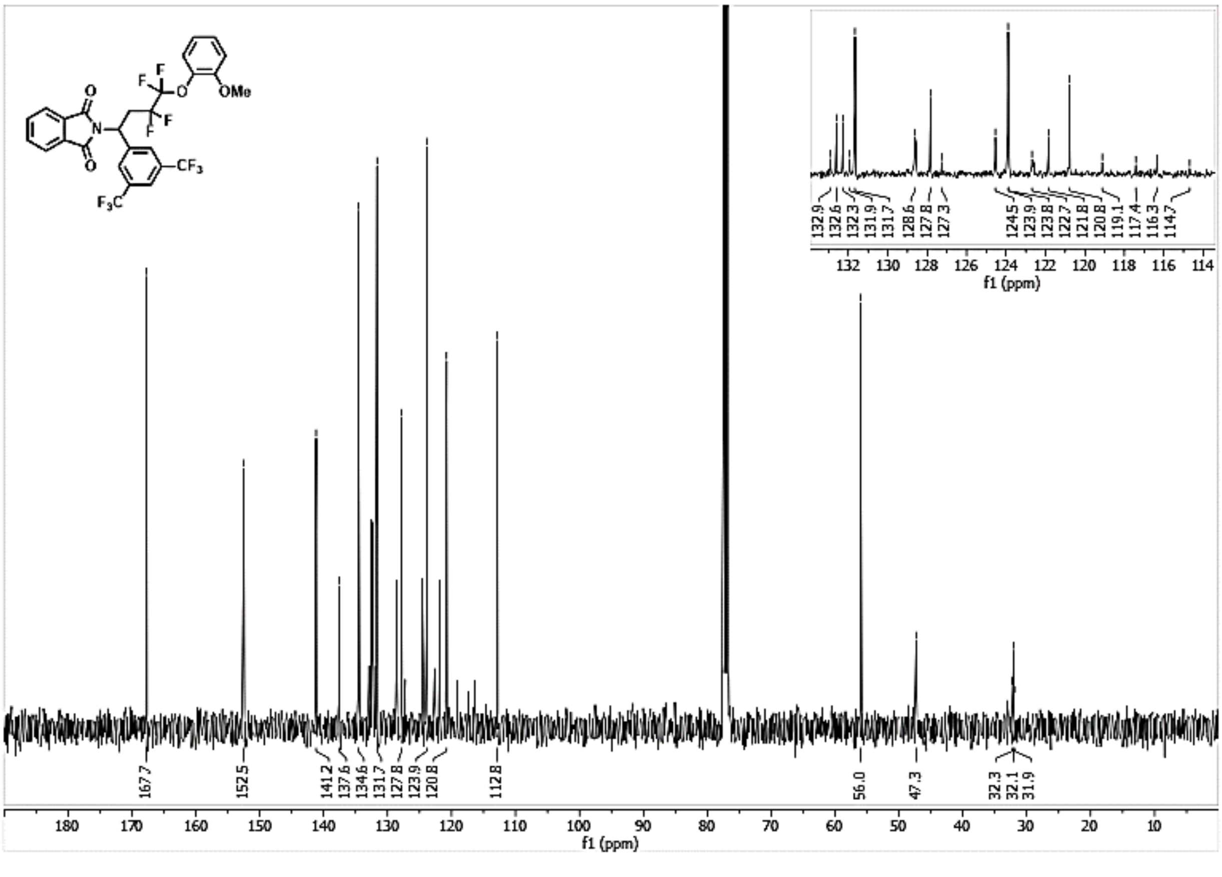

Compound 4i. ^{19}F NMR ($CDCl_3$, 376 MHz).

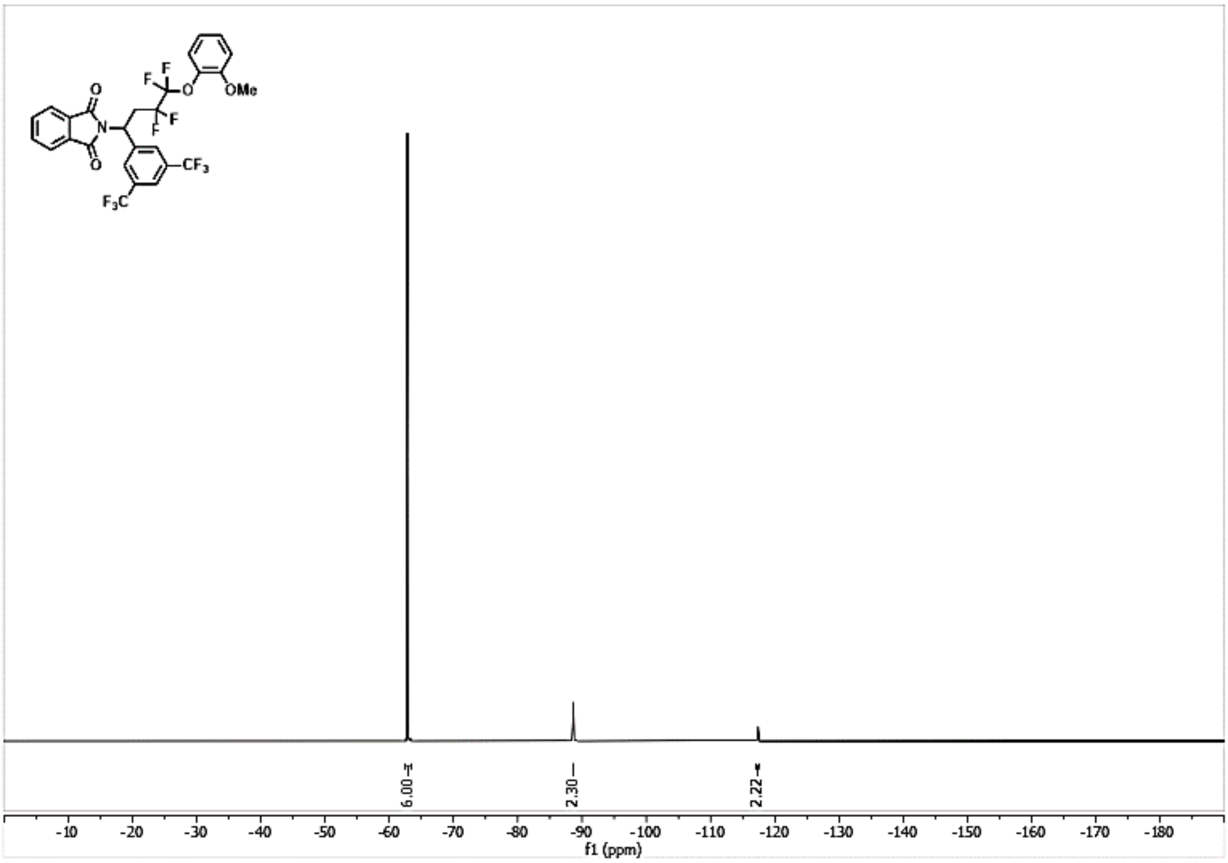

Compound 4j. Top: ^{1}H NMR ($CDCl_3$, 400 MHz). Bottom: ^{13}C NMR ($CDCl_3$, 100 MHz).

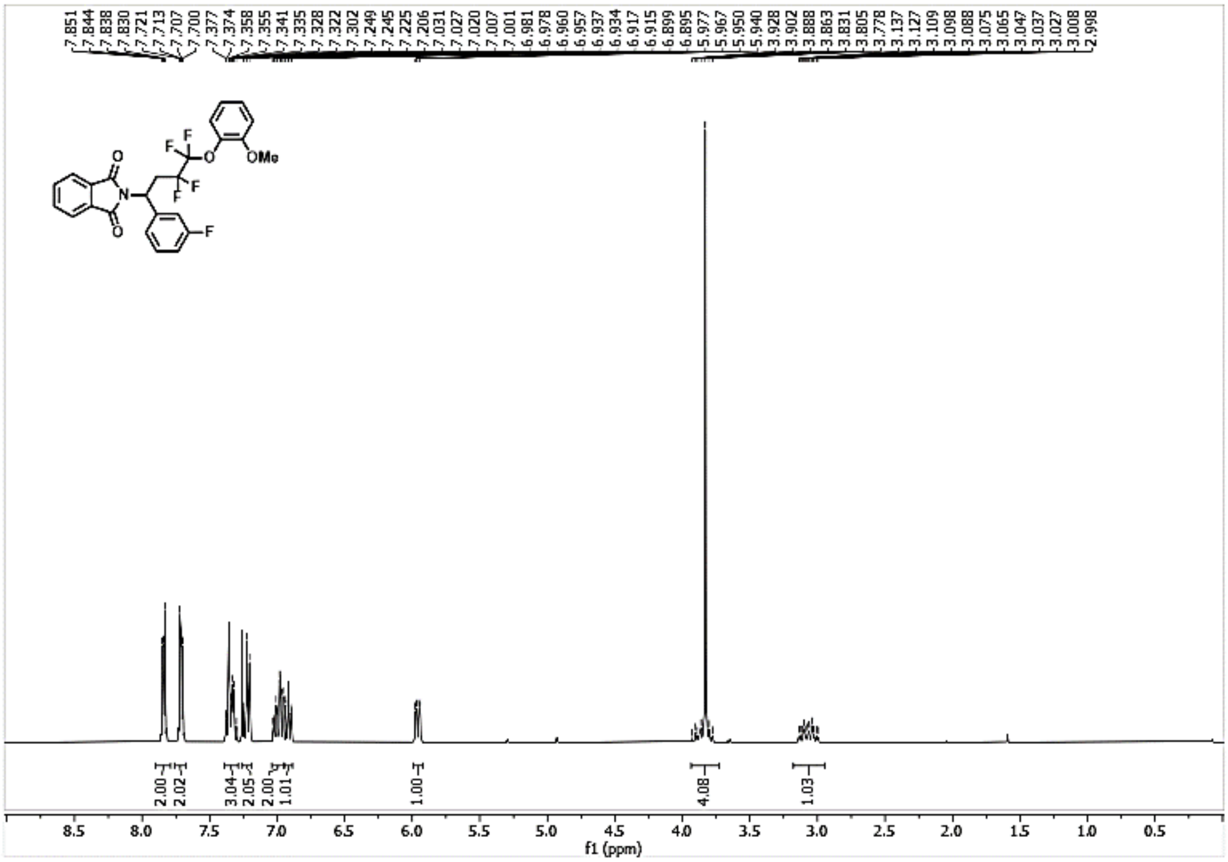

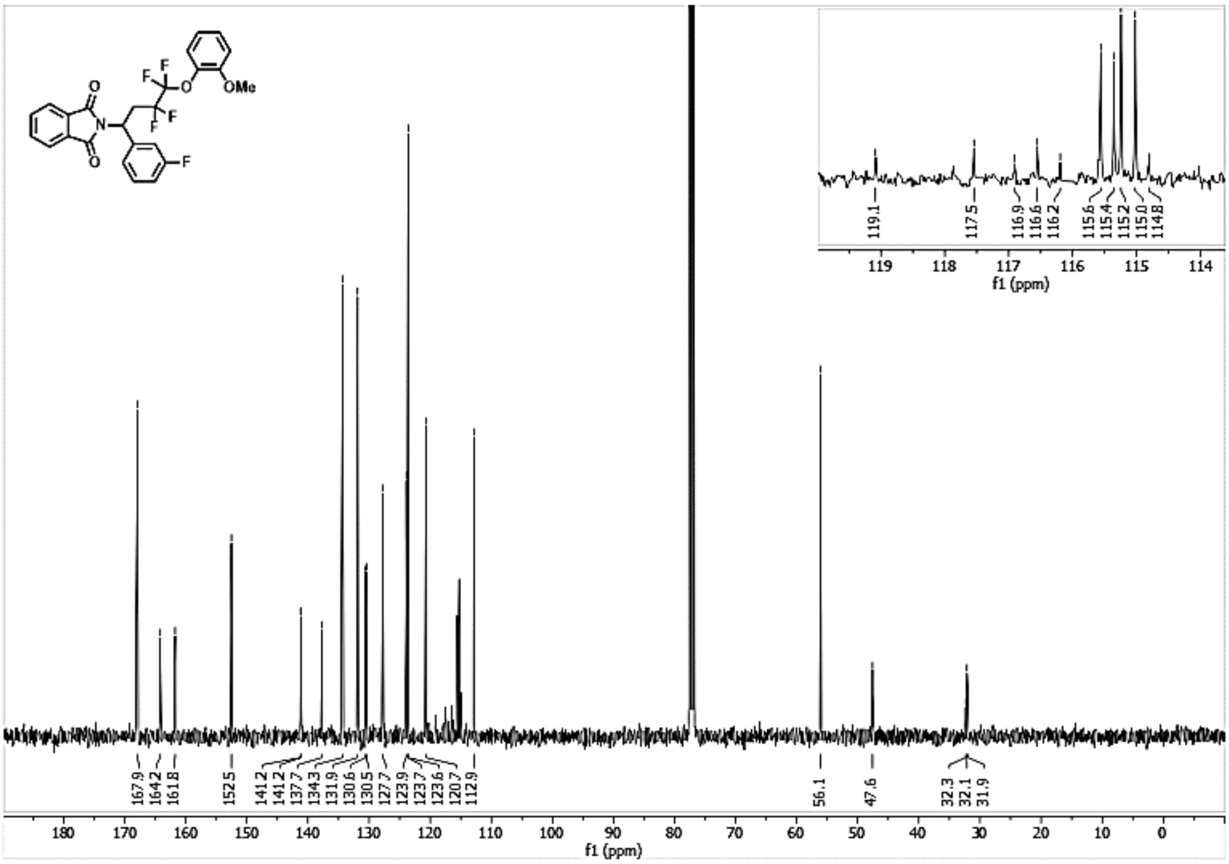

Compound 4j. ^{19}F NMR ($CDCl_3$, 376 MHz).

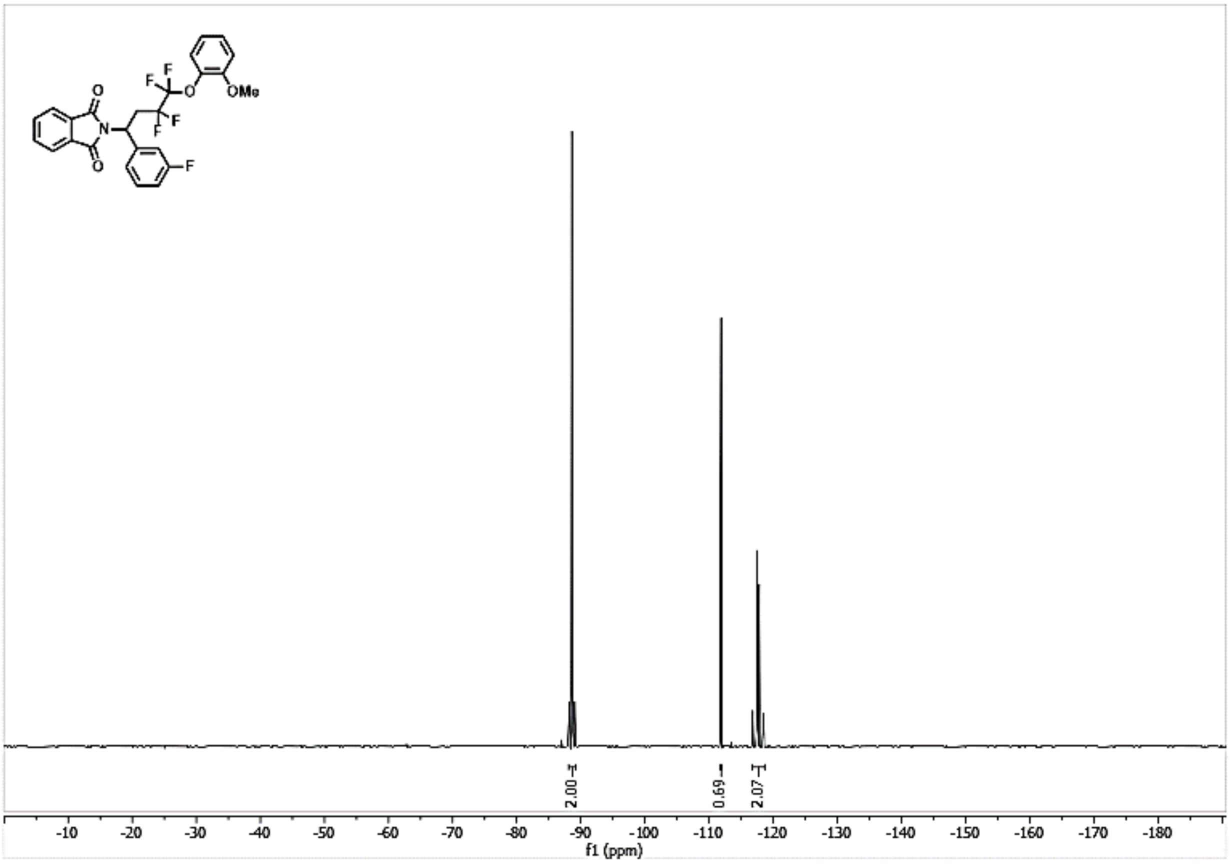

Compound 4k. Top: ^{1}H NMR ($CDCl_3$, 400 MHz). Bottom: ^{13}C NMR ($CDCl_3$, 100 MHz).

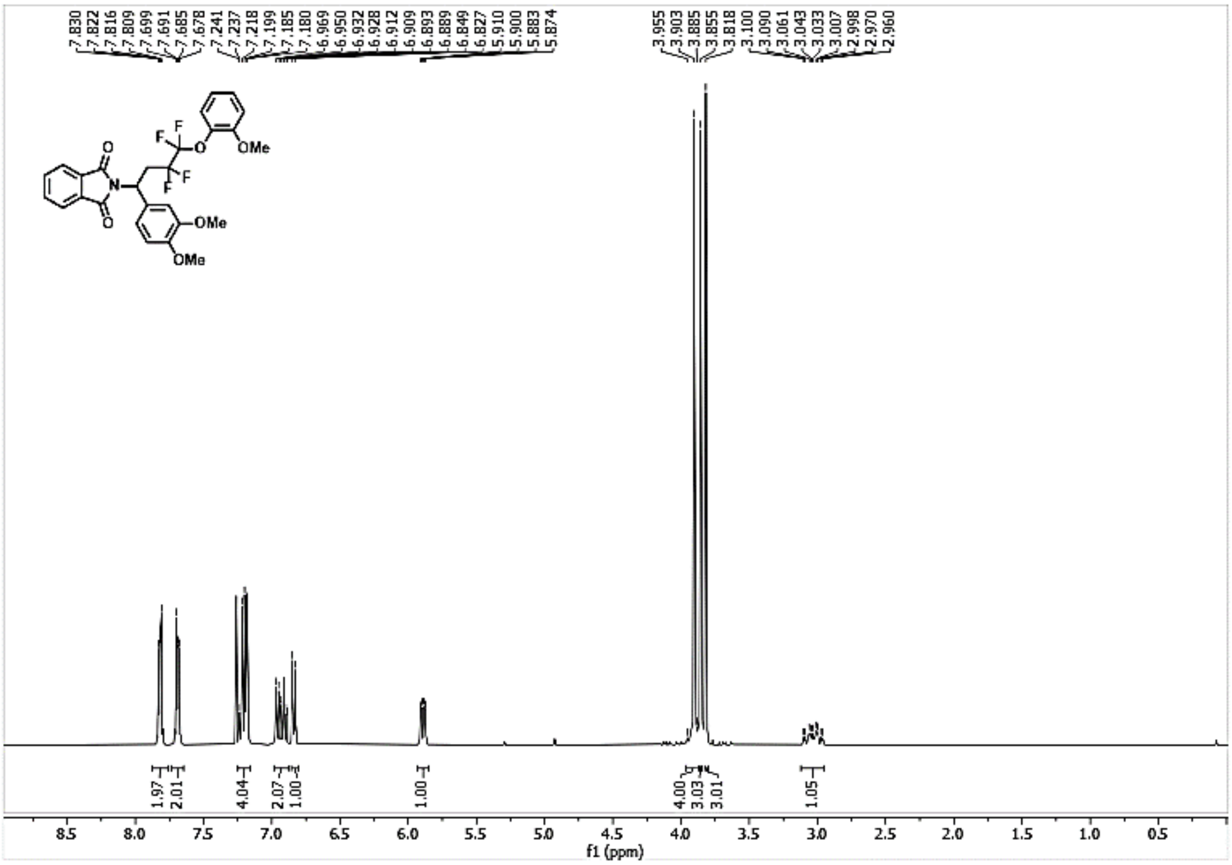

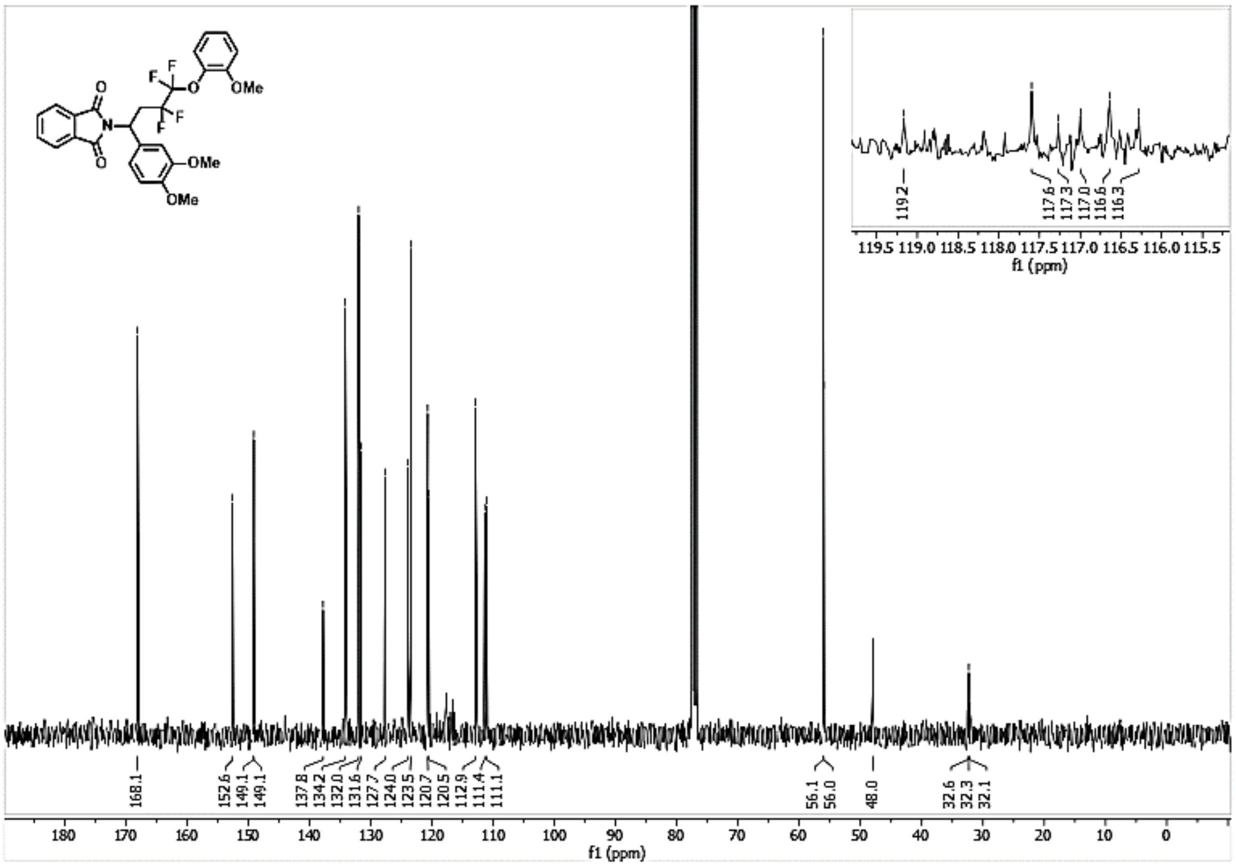

Compound 4k. ^{19}F NMR ($CDCl_3$, 376 MHz).

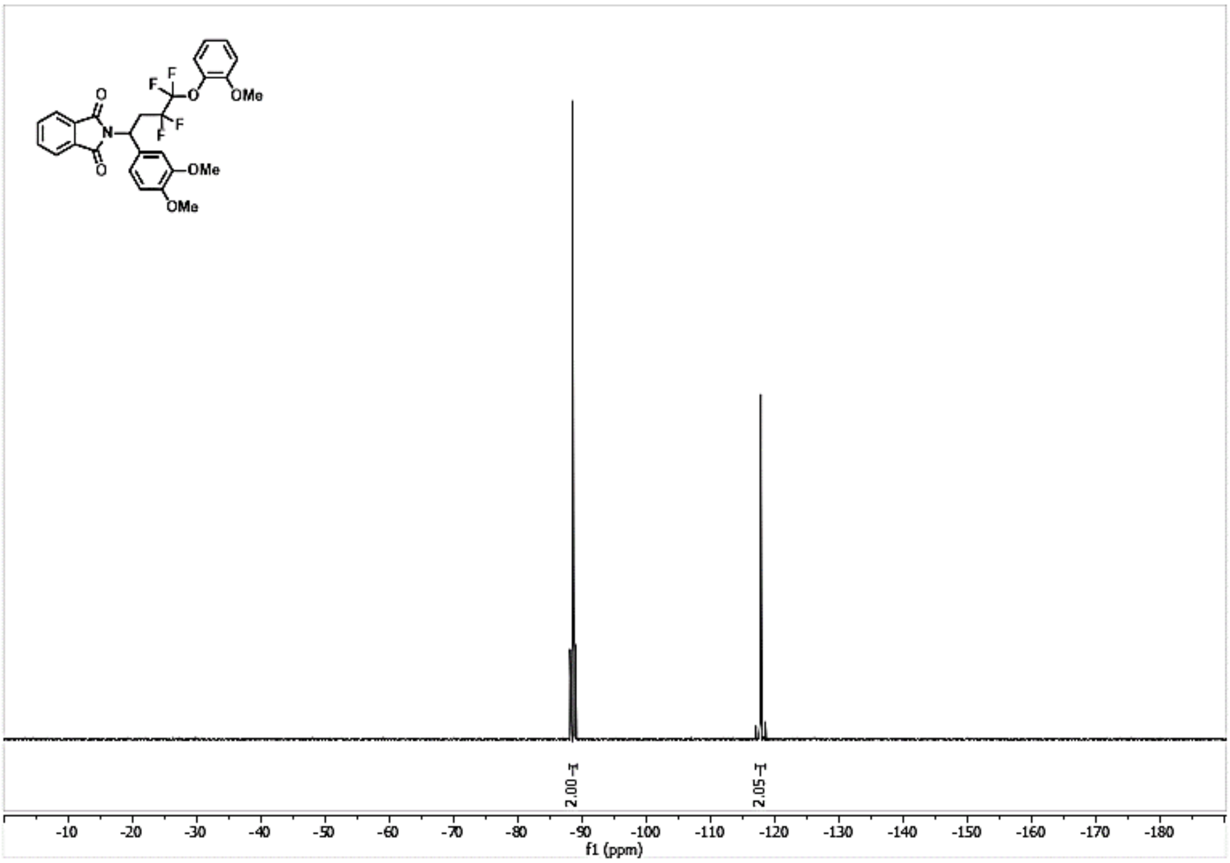

F F
O
OMe
O
N
F F
O
OMe
OMe
2.00
2.05
-10 -20 -30 -40 -50 -60 -70 -80 -90 -100 -110 -120 -130 -140 -150 -160 -170 -180
f1 (ppm)

Compound 4l. Top: ^{1}H NMR ($CDCl_3$, 400 MHz). Bottom: ^{13}C NMR ($CDCl_3$, 100 MHz).

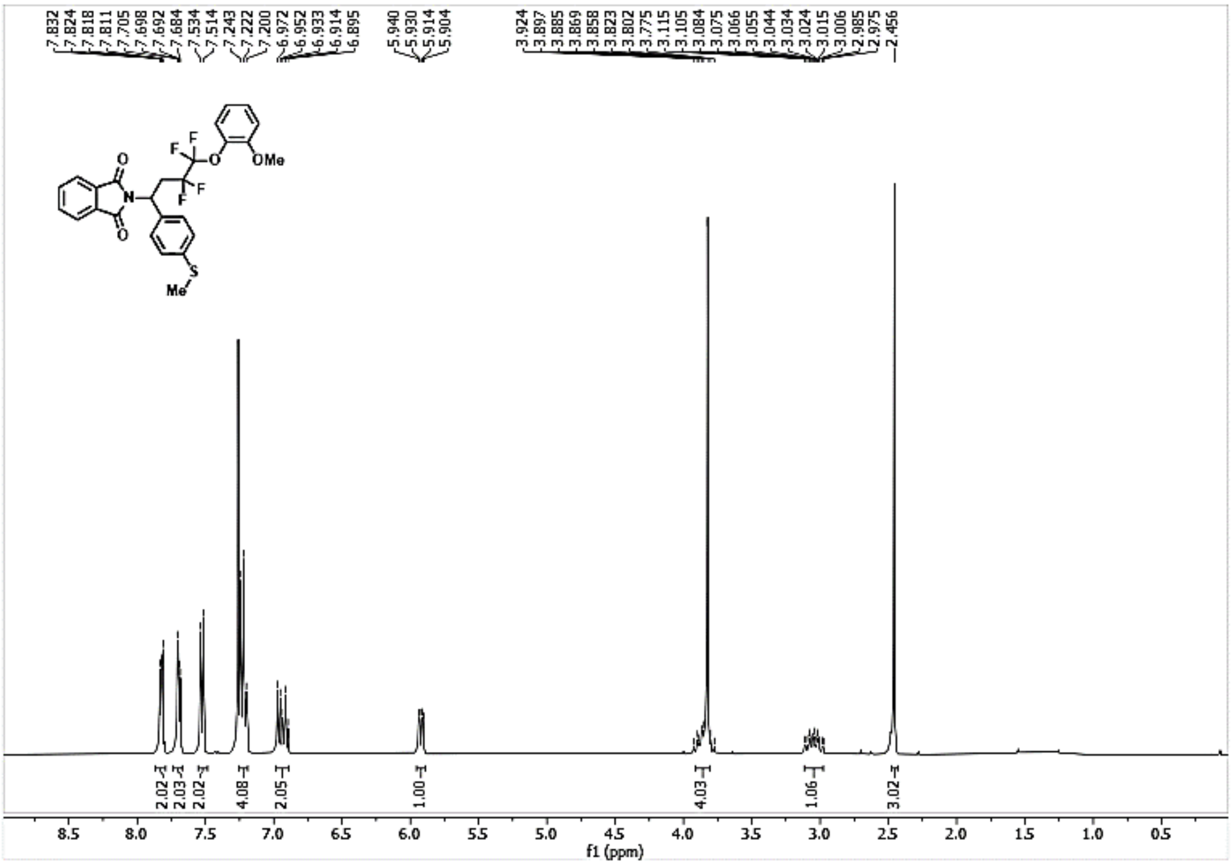

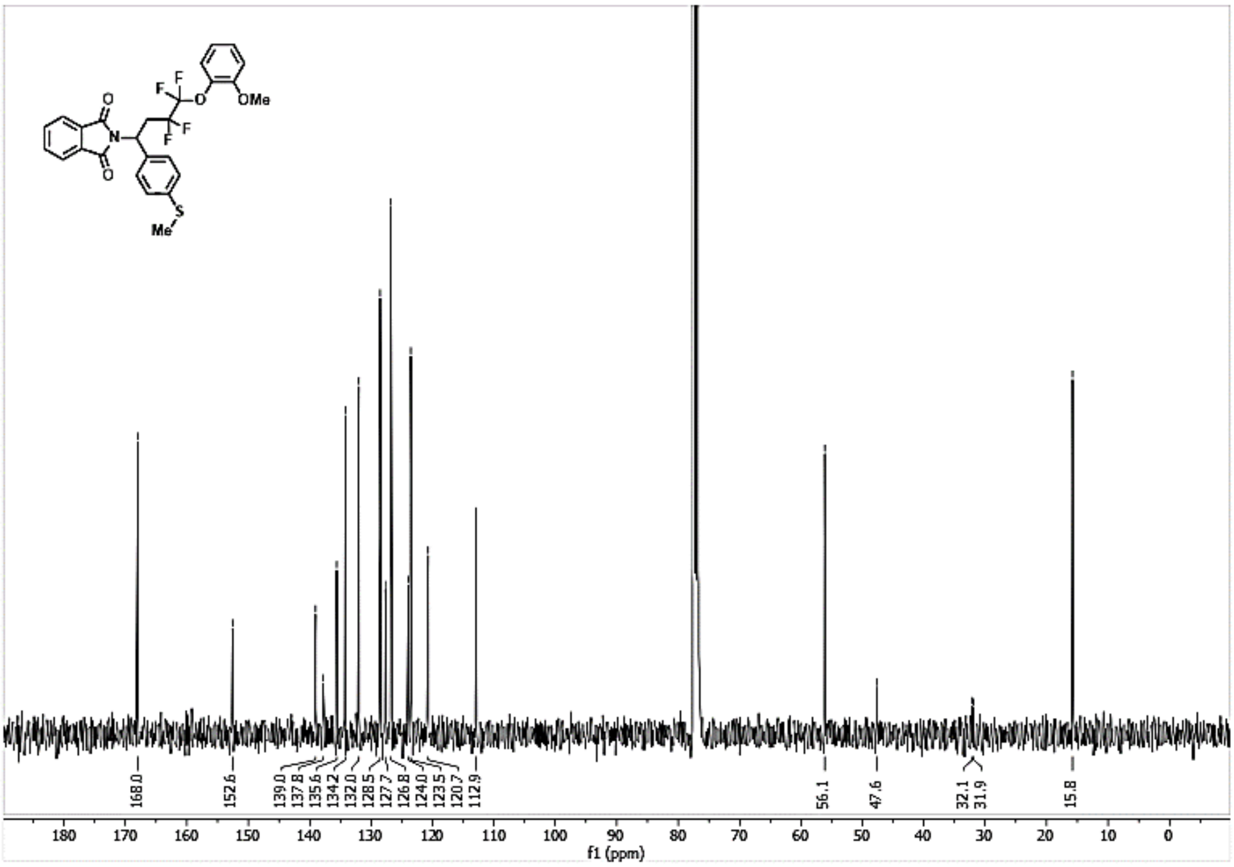

Compound 4l. ^{19}F NMR ($CDCl_3$, 376 MHz).

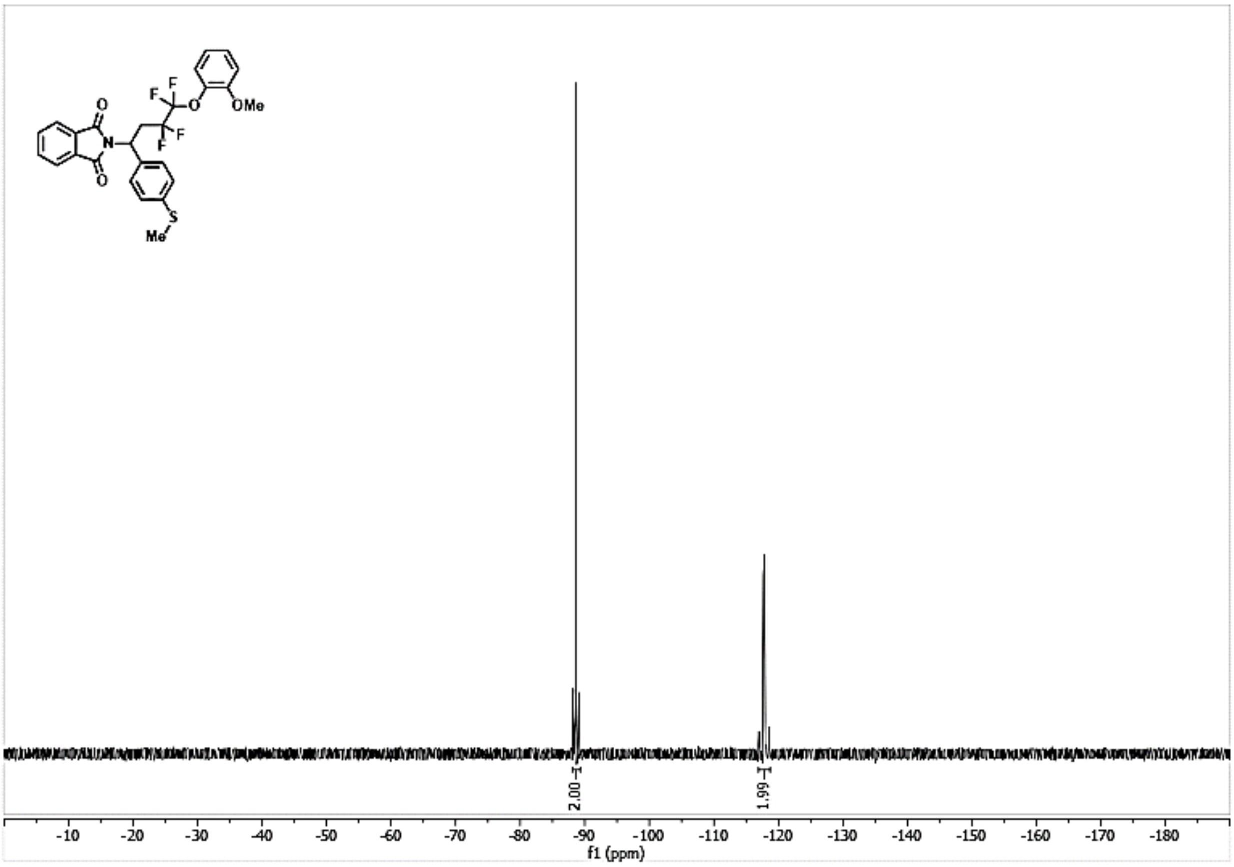

Compound 4m. Top: ^{1}H NMR ($CDCl_3$, 400 MHz). Bottom: ^{13}C NMR ($CDCl_3$, 100 MHz).

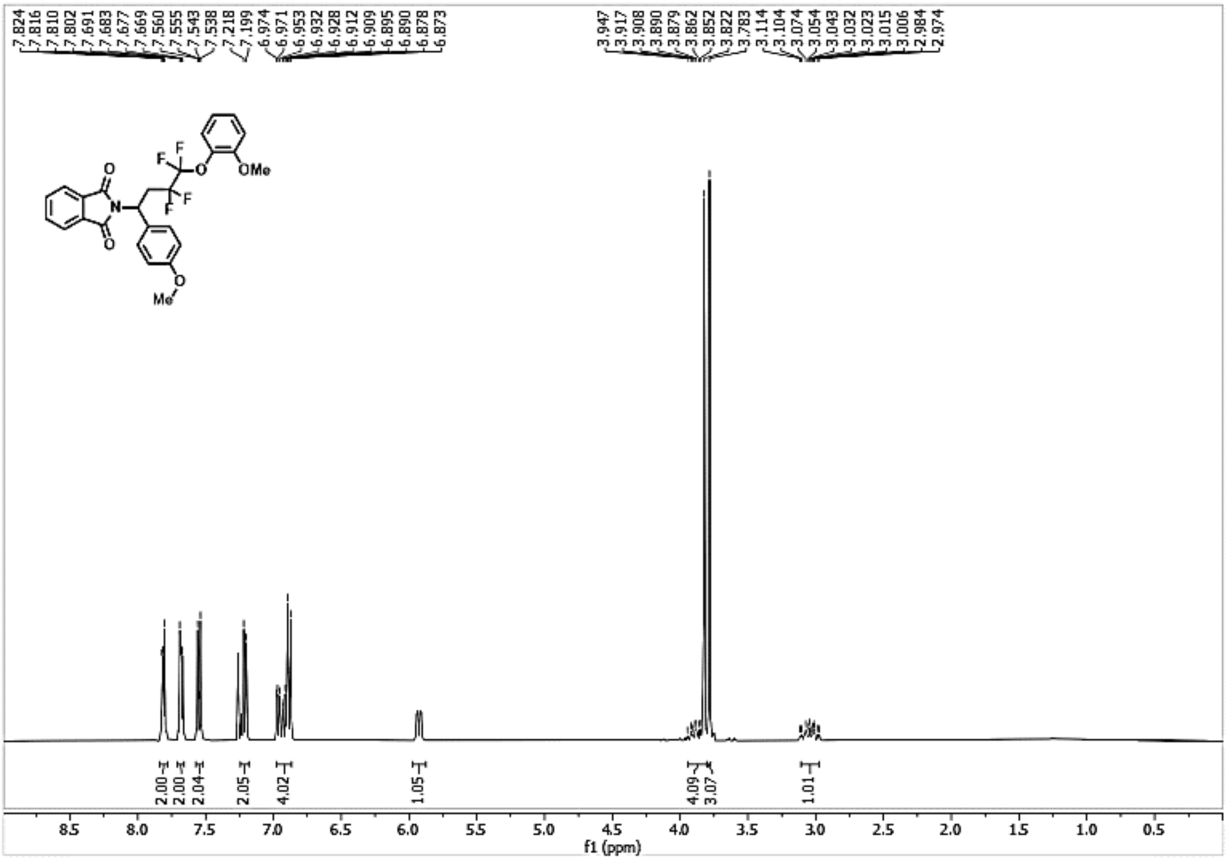

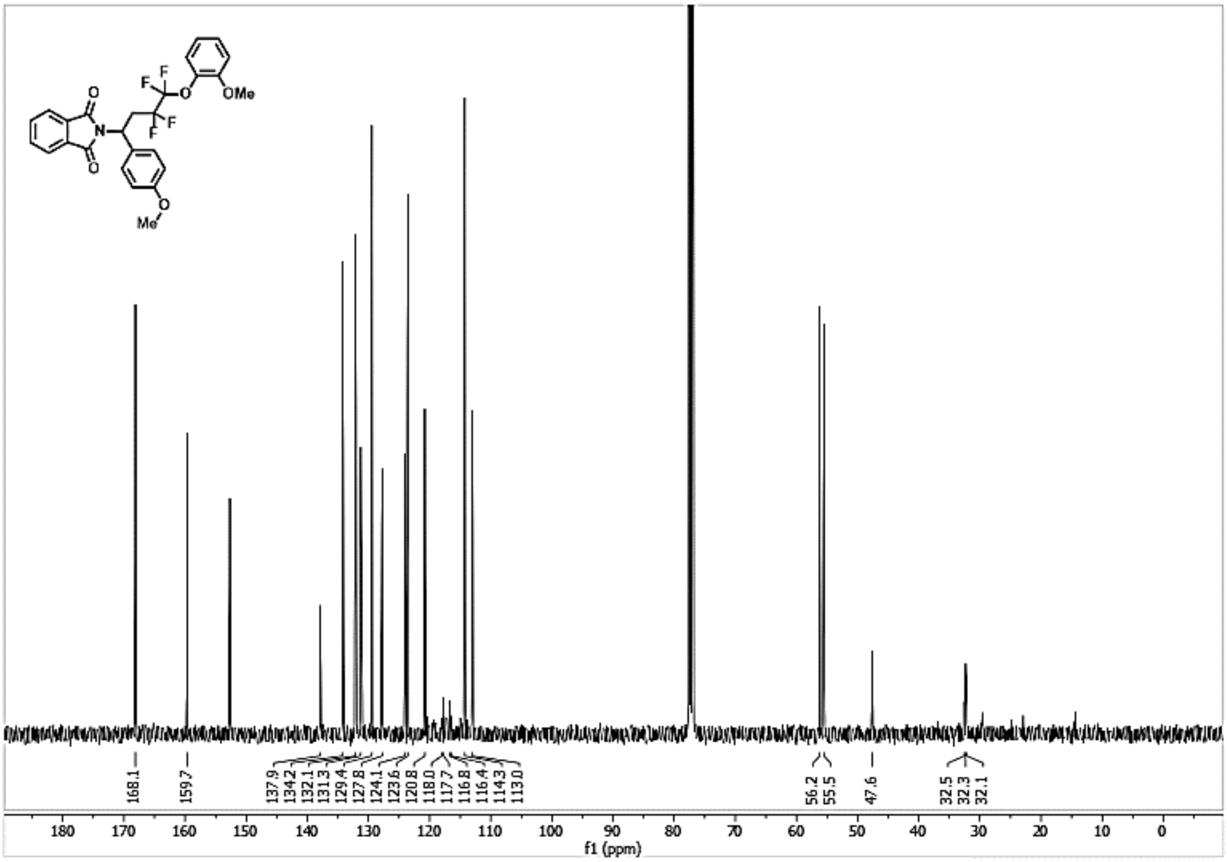

Compound 4m. ^{19}F NMR ($CDCl_3$, 376 MHz).

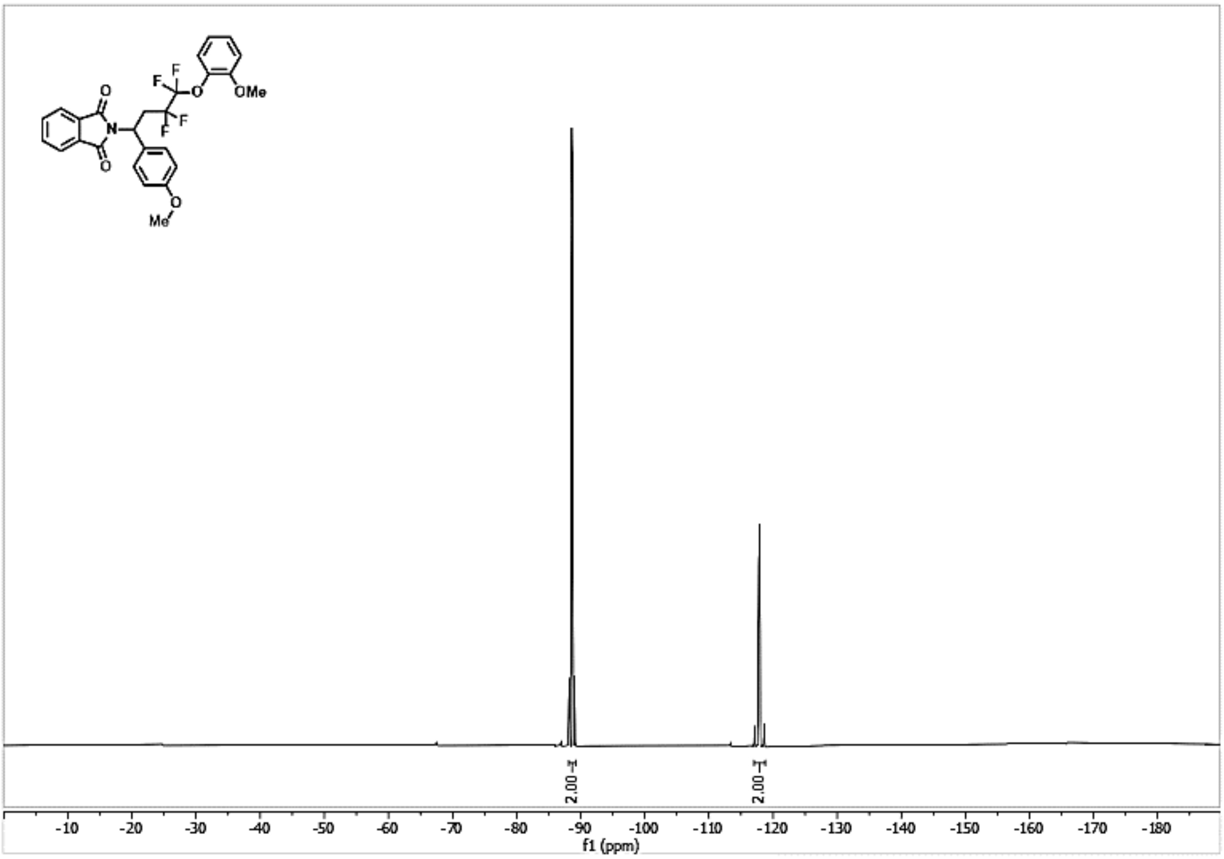

Compound 4n. Top: ^{1}H NMR ($CDCl_3$, 400 MHz). Bottom: ^{13}C NMR ($CDCl_3$, 100 MHz).

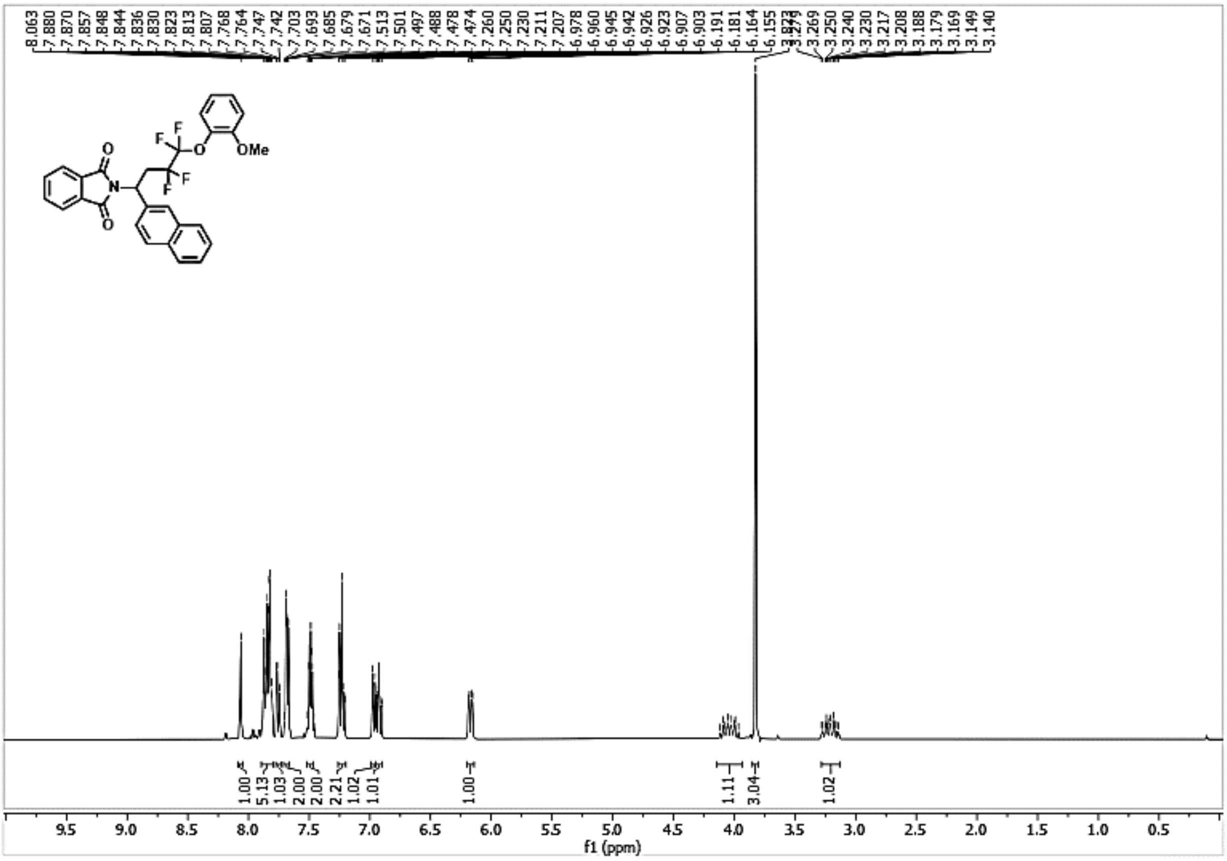

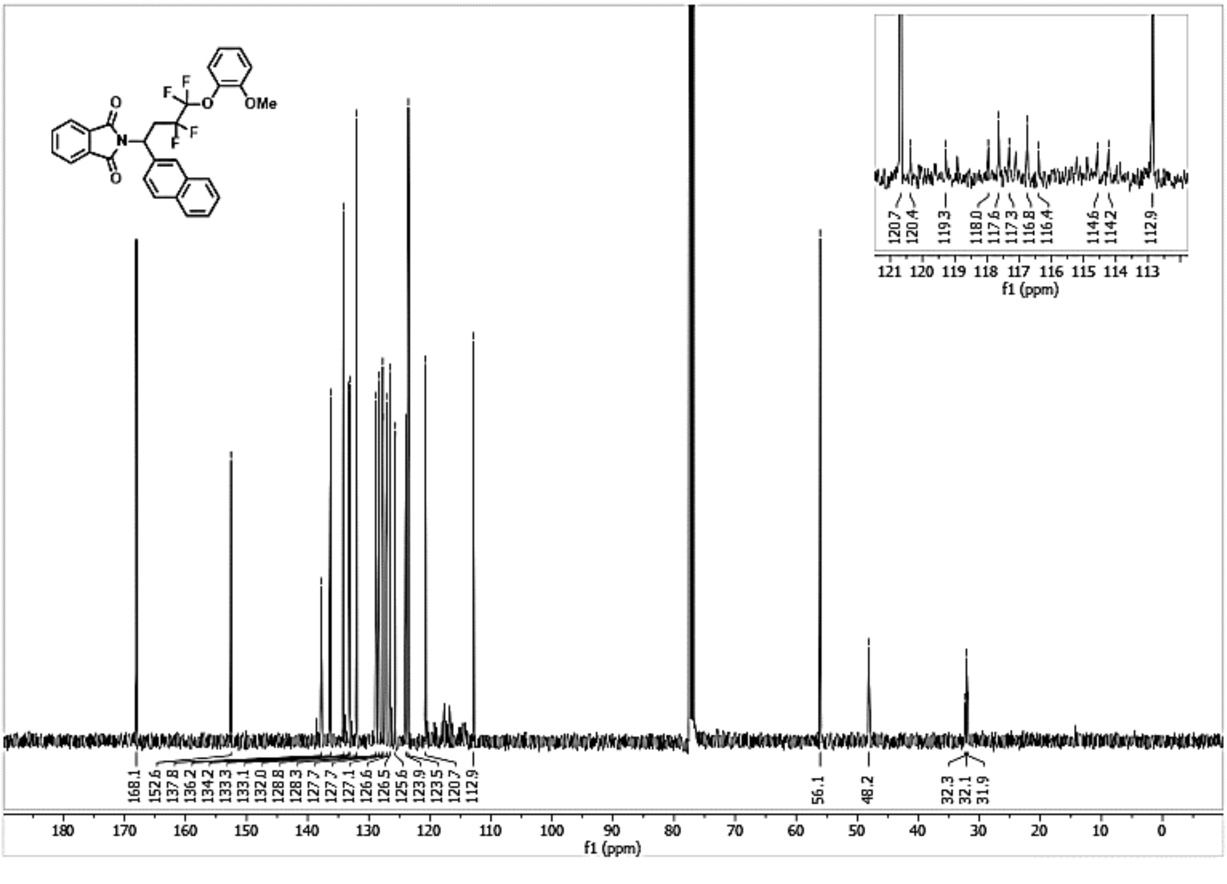

Compound 4n. ^{19}F NMR ($CDCl_3$, 376 MHz).

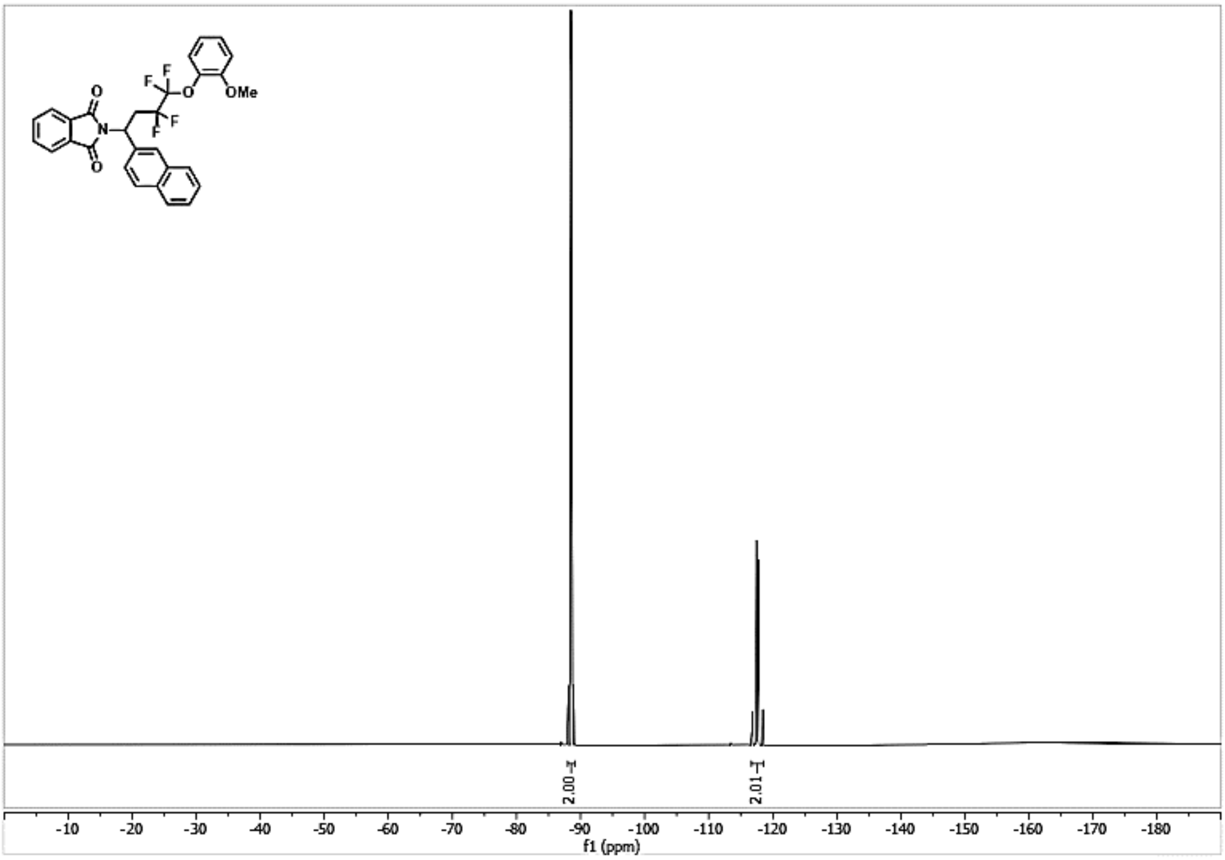

Compound 5a. Top: ^{1}H NMR ($CDCl_3$, 400 MHz). Bottom: ^{13}C NMR ($CDCl_3$, 100 MHz).

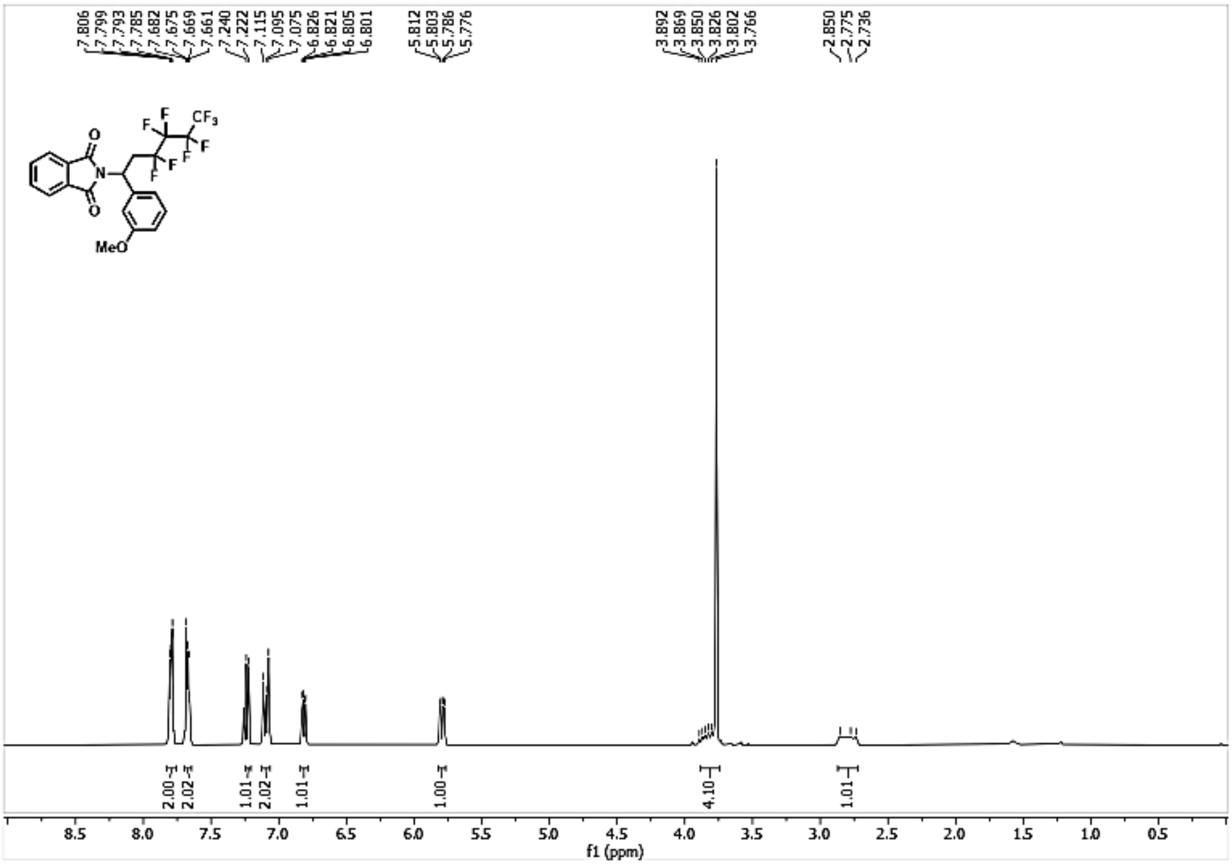

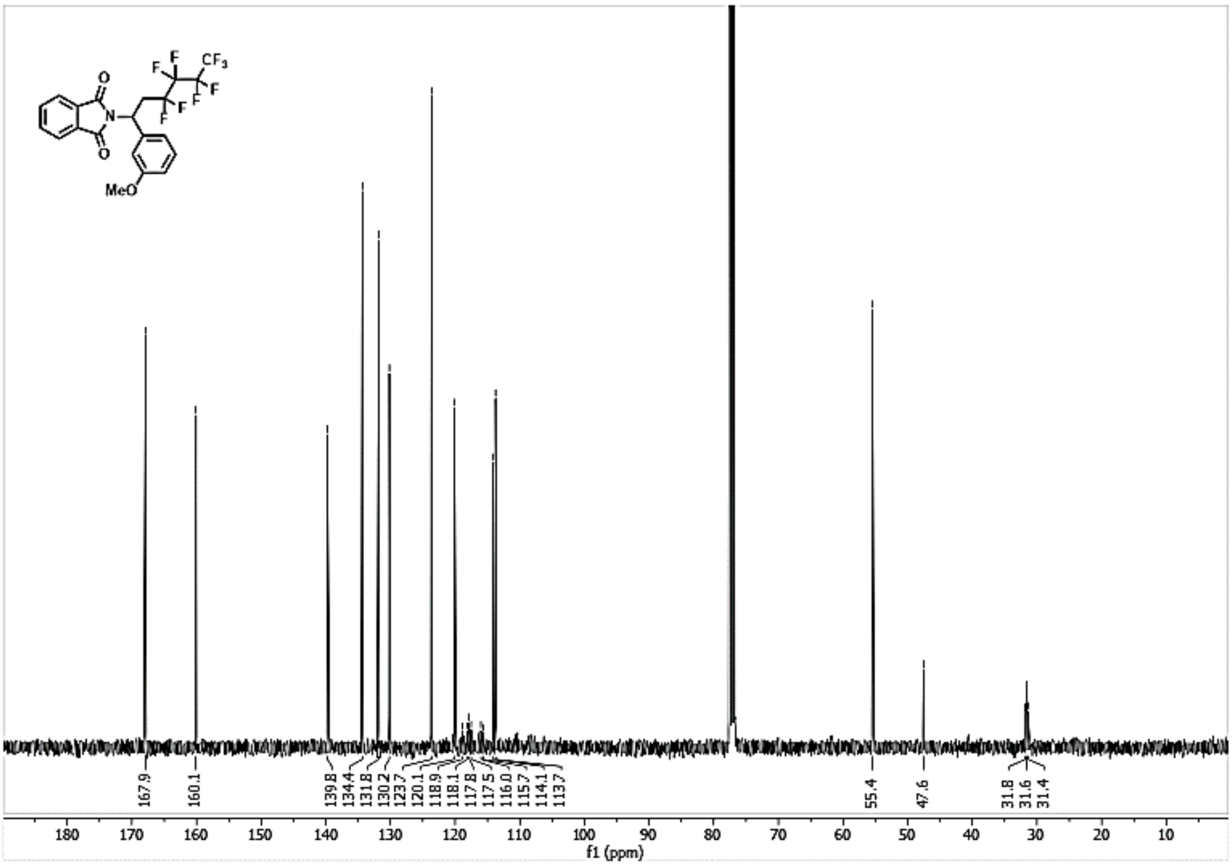

Compound 5a. ^{19}F NMR ($CDCl_3$, 376 MHz).

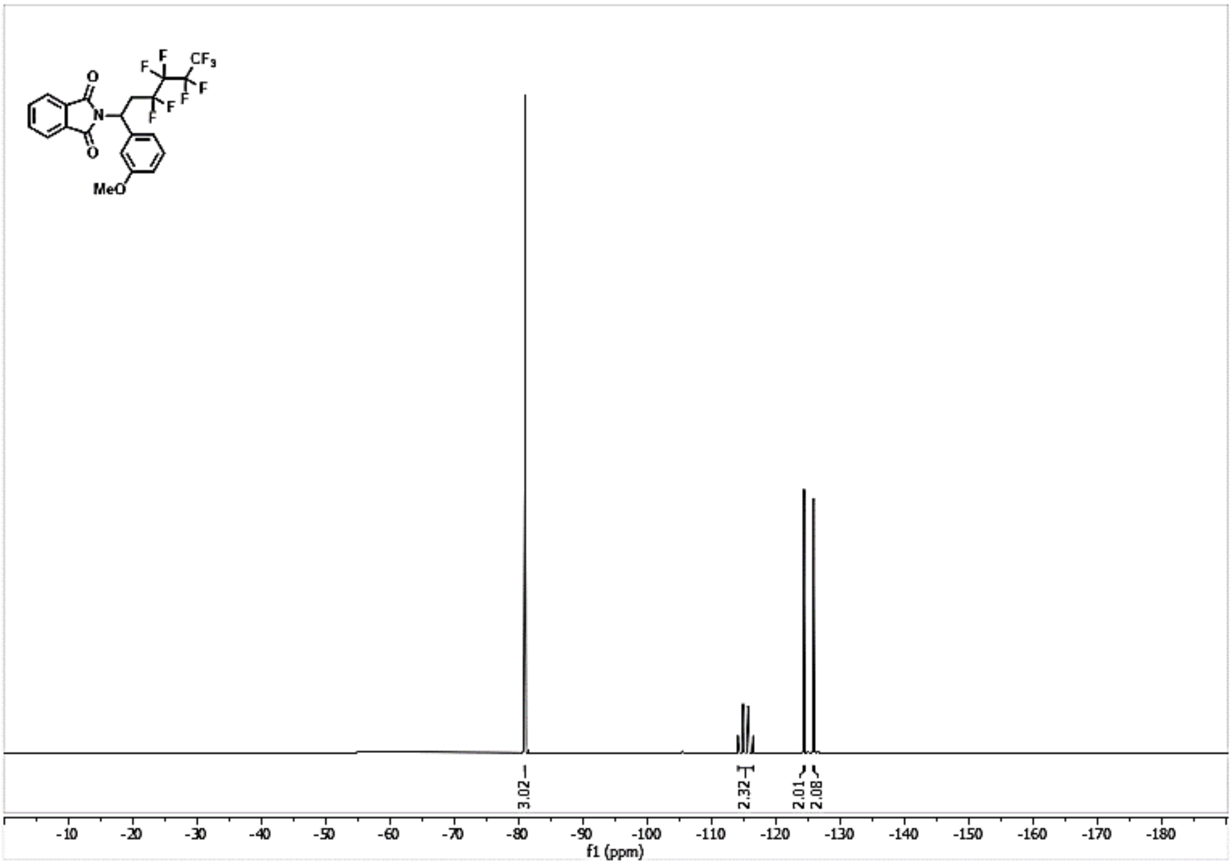

Compound 5b. Top: ^{1}H NMR ($CDCl_3$, 400 MHz). Bottom: ^{13}C NMR ($CDCl_3$, 100 MHz).

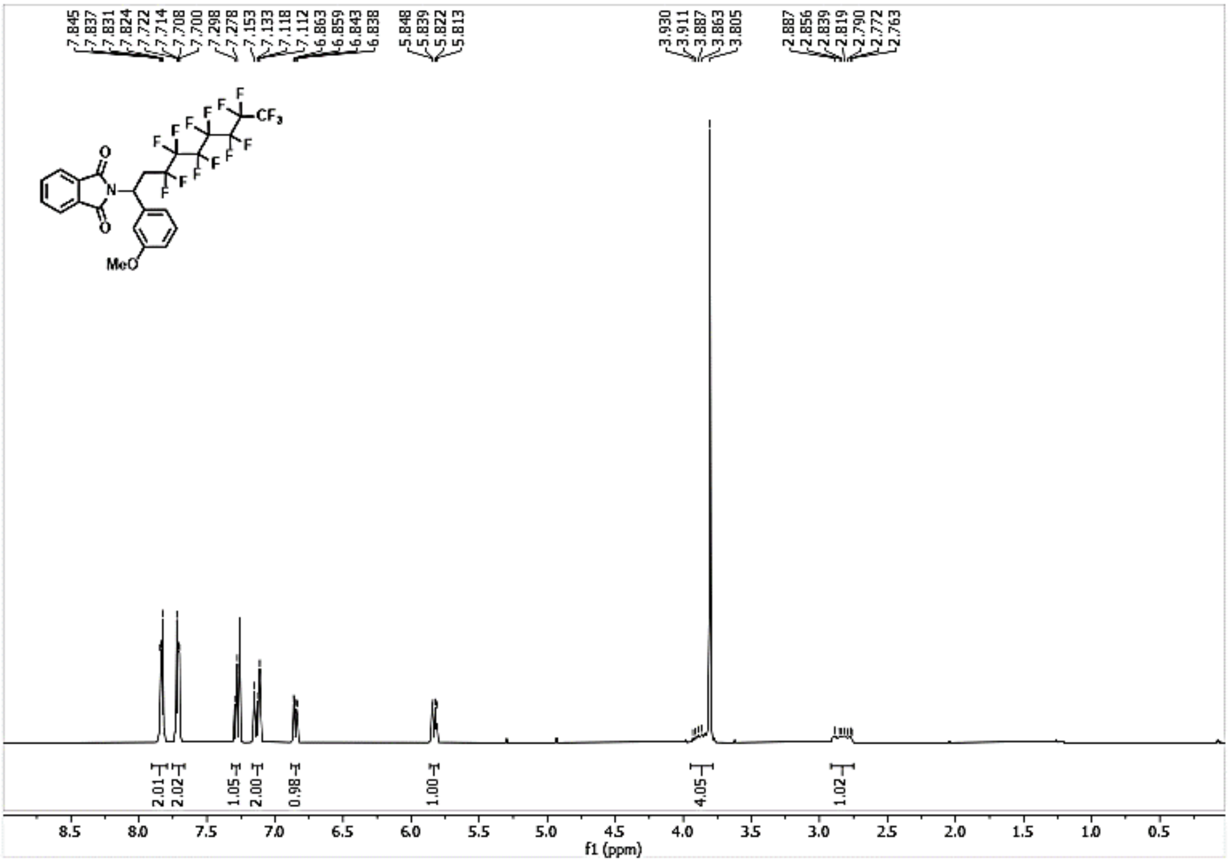

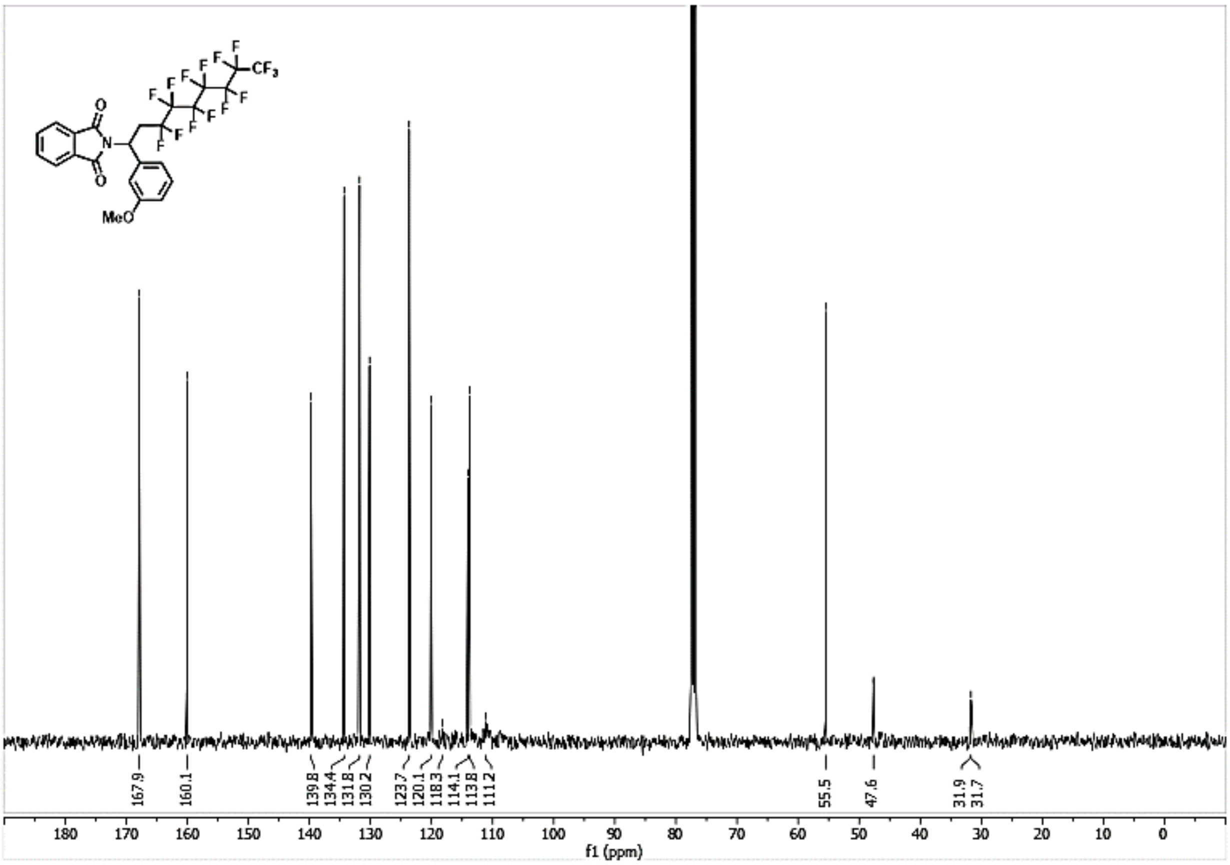

Compound 5b. ^{19}F NMR ($CDCl_3$, 376 MHz).

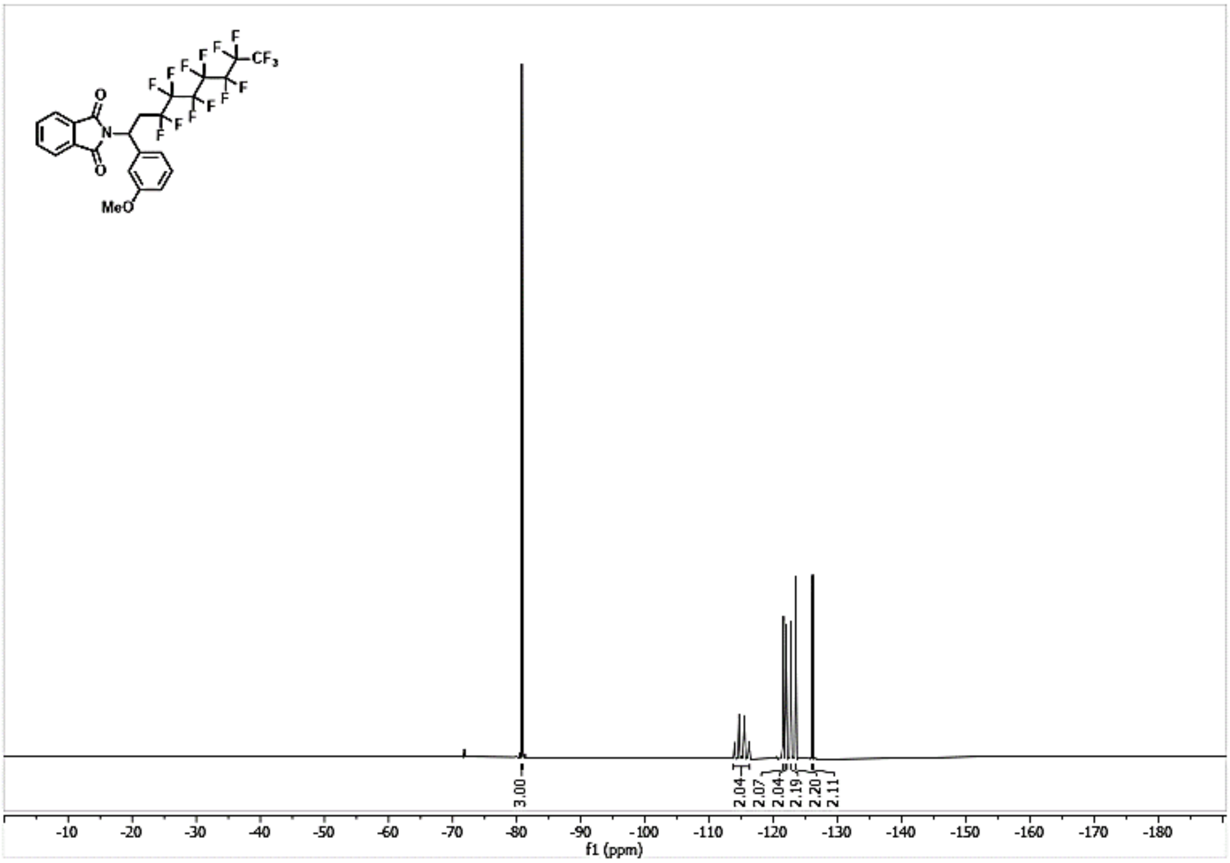

Compound 5c. Top: ^{1}H NMR ($CDCl_3$, 400 MHz). Bottom: ^{13}C NMR ($CDCl_3$, 100 MHz).

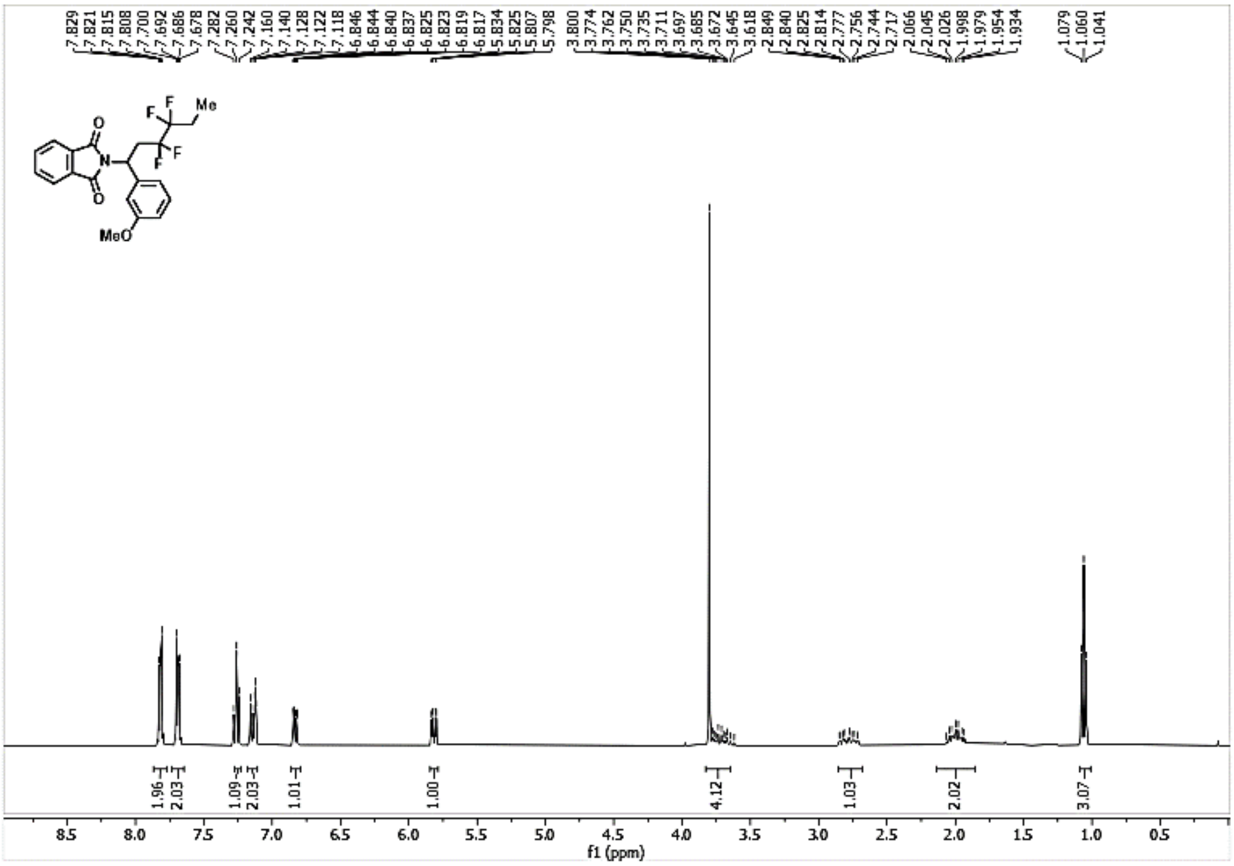

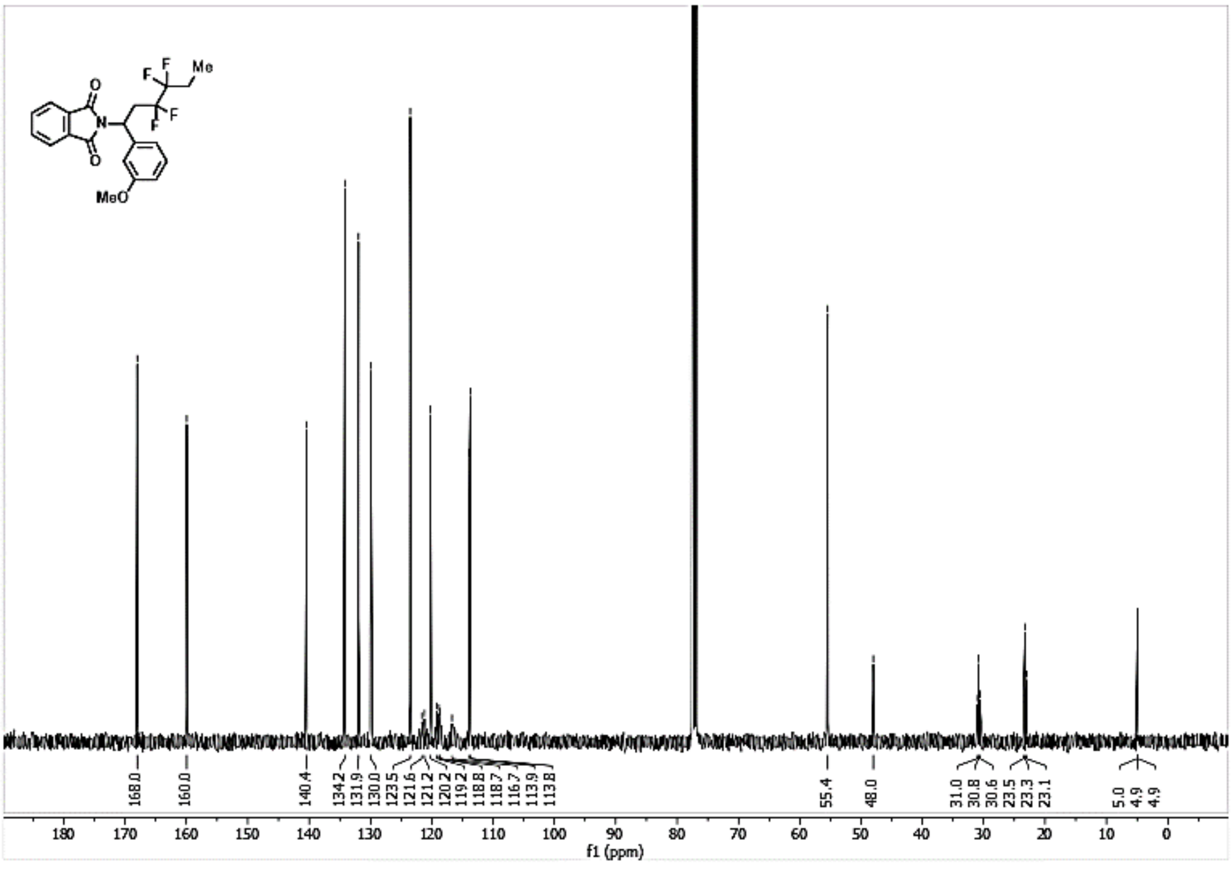

Compound 5c. ^{19}F NMR ($CDCl_3$, 376 MHz).

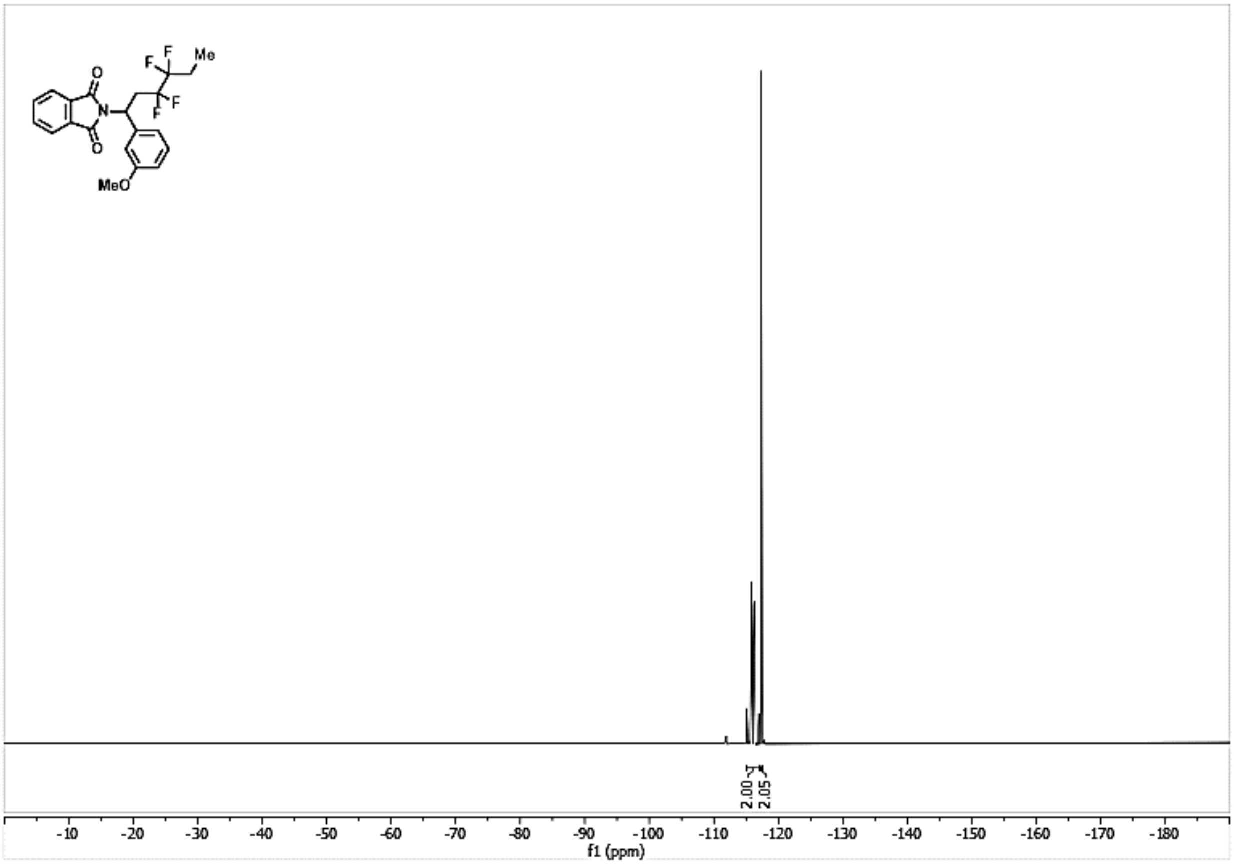

Compound 5d. Top: ^{1}H NMR ($CDCl_3$, 400 MHz). Bottom: ^{13}C NMR ($CDCl_3$, 100 MHz).

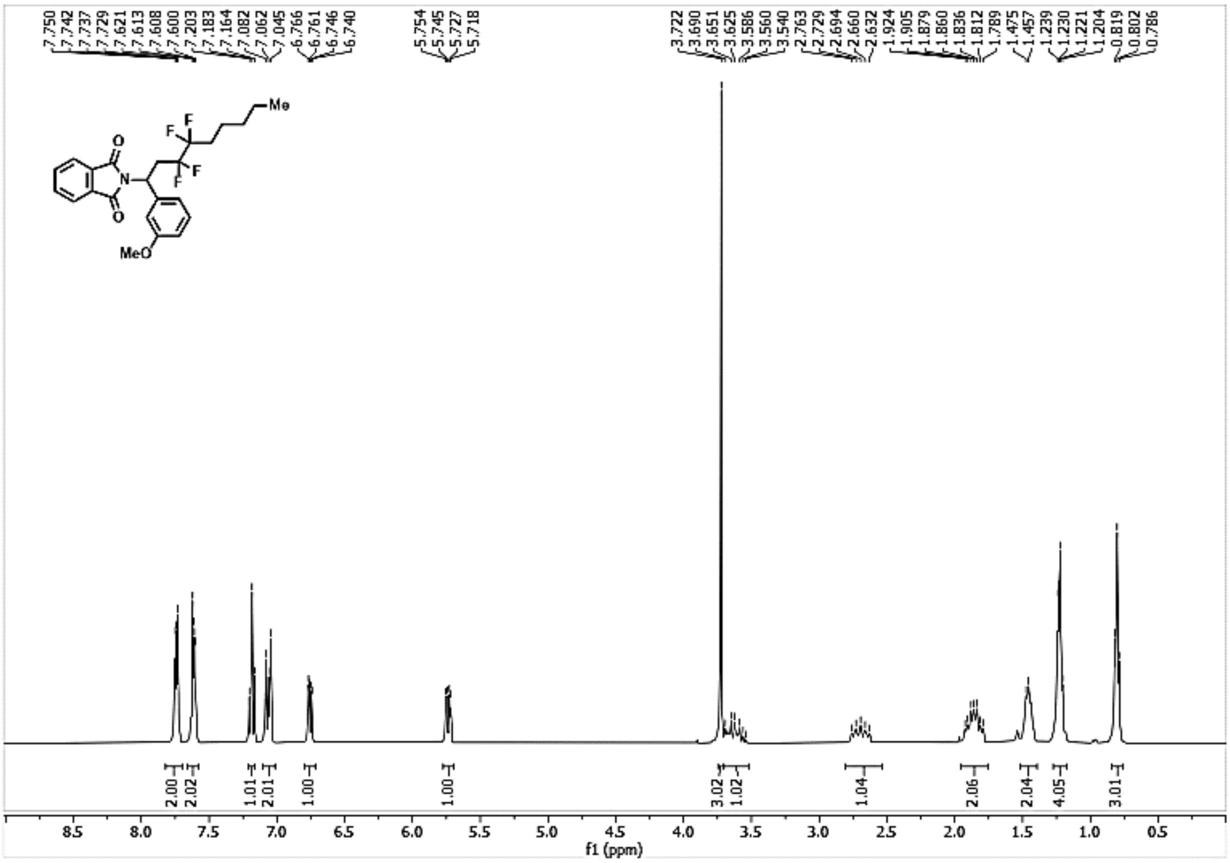

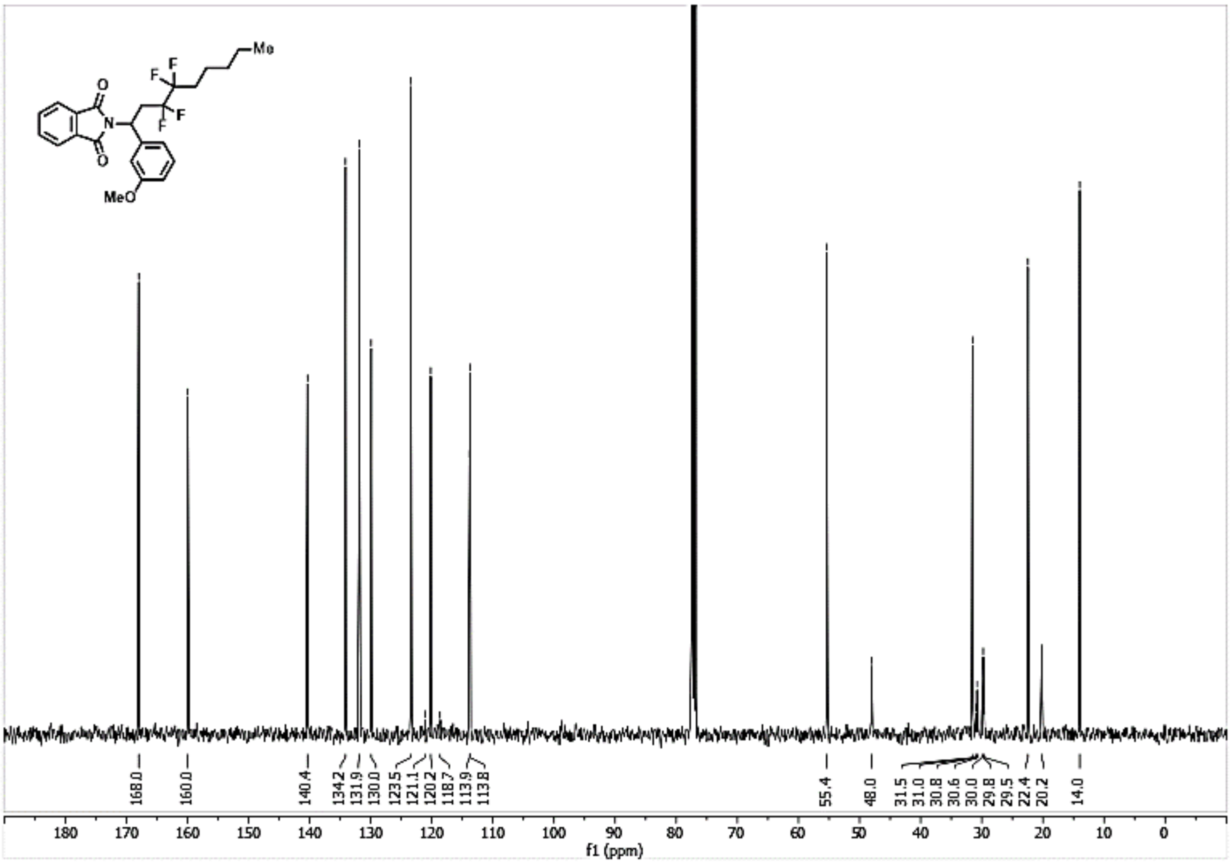

Compound 5d. ^{19}F NMR ($CDCl_3$, 376 MHz).

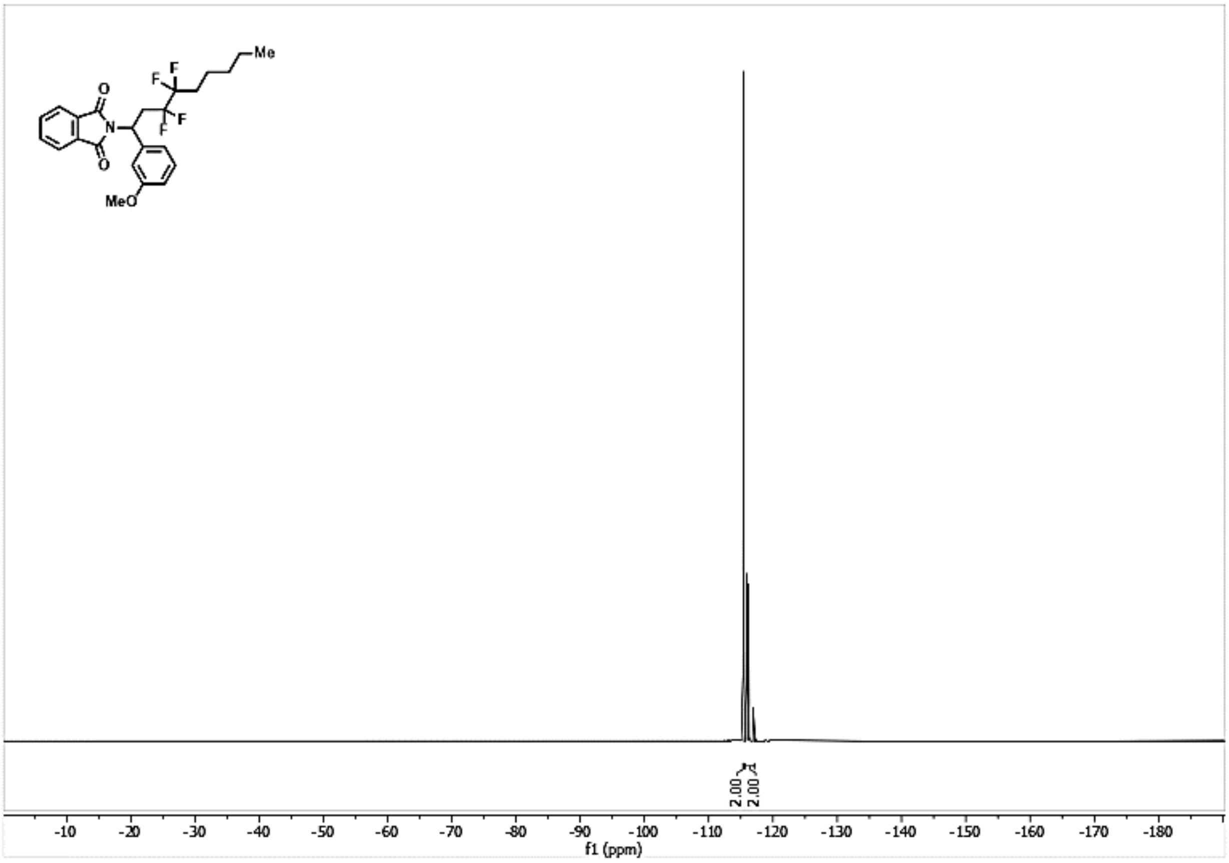

Compound 5e. Top: ^1H NMR ($CDCl_3$, 400 MHz). Bottom: ^{13}C NMR ($CDCl_3$, 100 MHz).

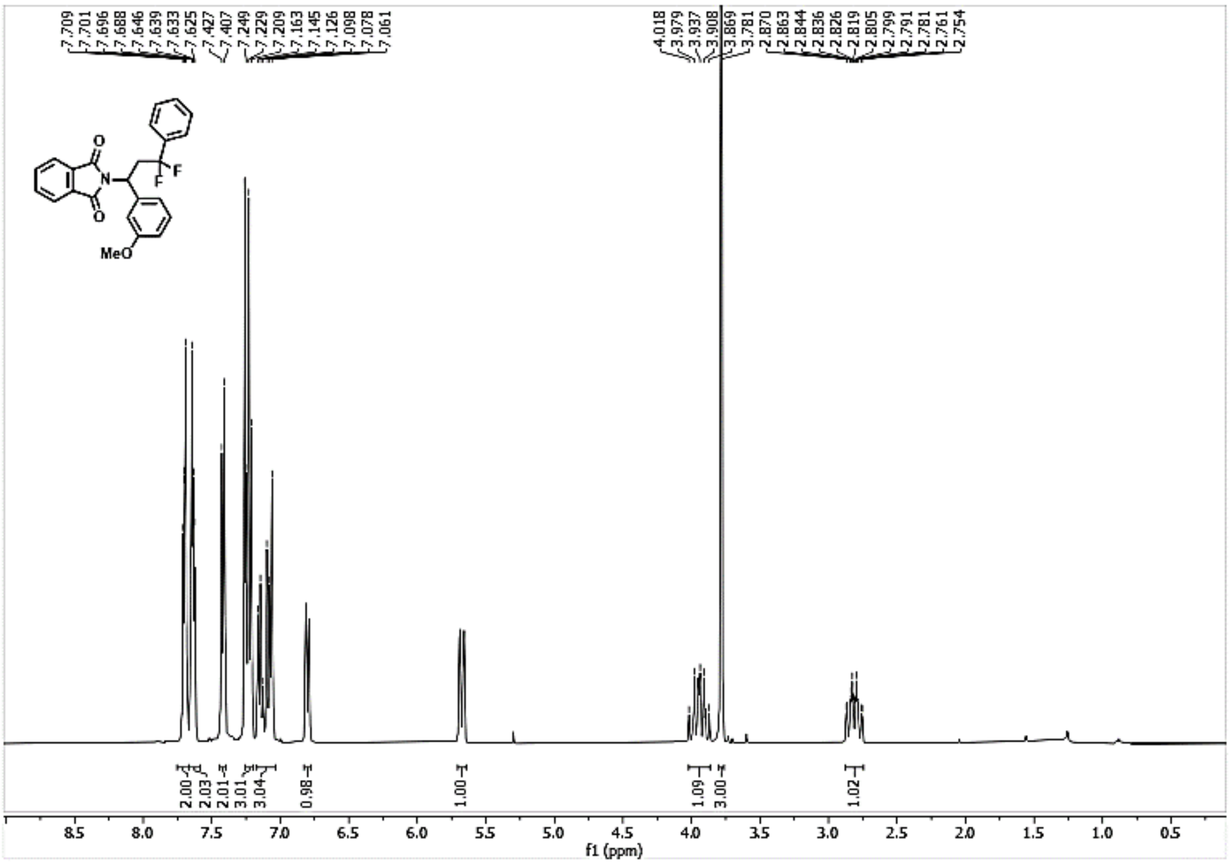

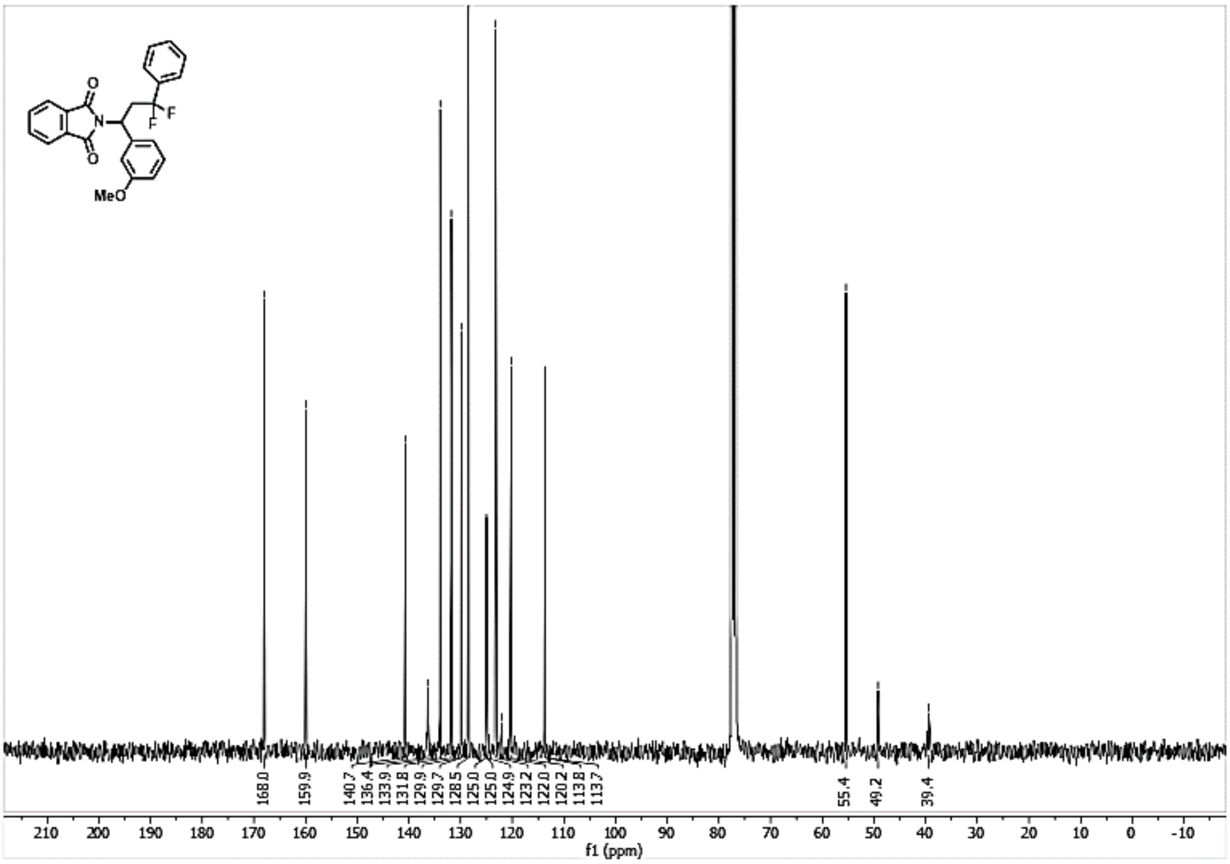

Compound 5e. ^{19}F NMR ($CDCl_3$, 376 MHz).

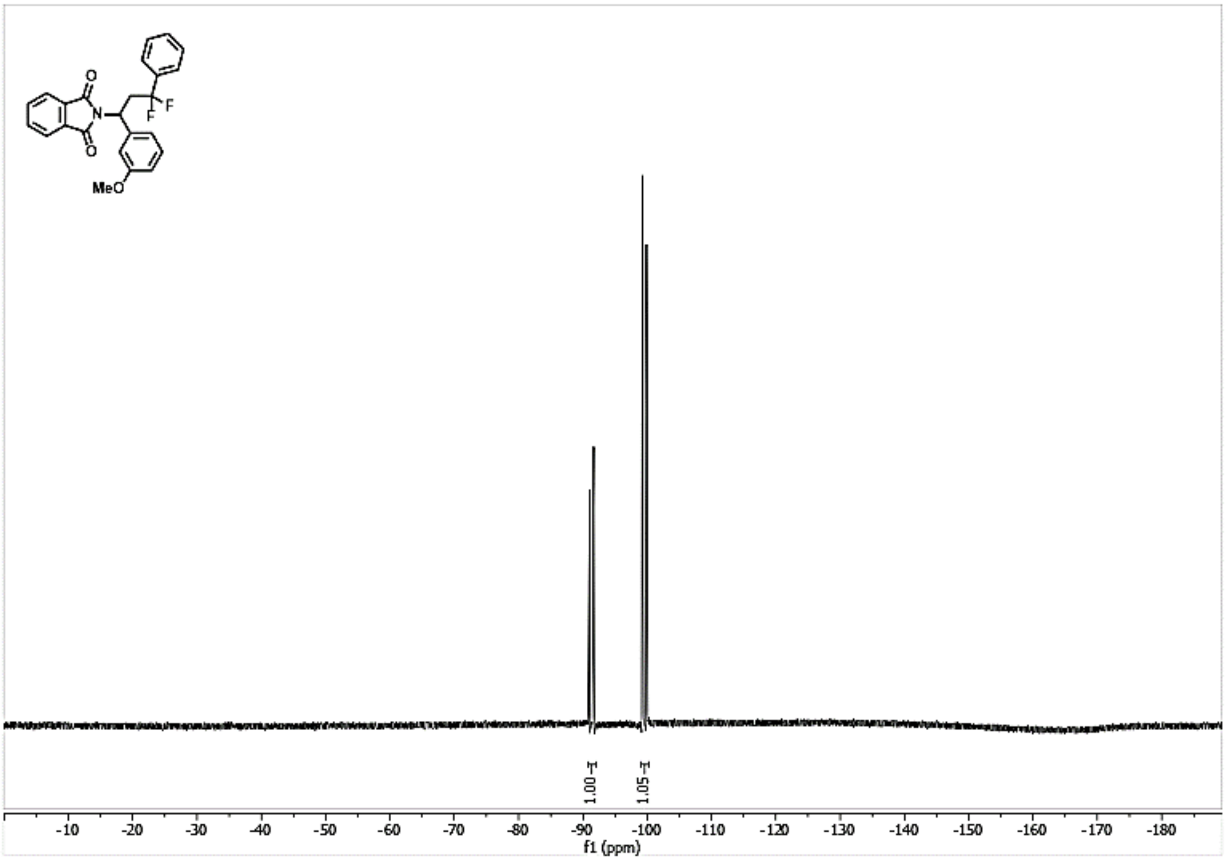

Compound 5f. Top: ^{1}H NMR ($CDCl_3$, 400 MHz). Bottom: ^{13}C NMR ($CDCl_3$, 100 MHz).

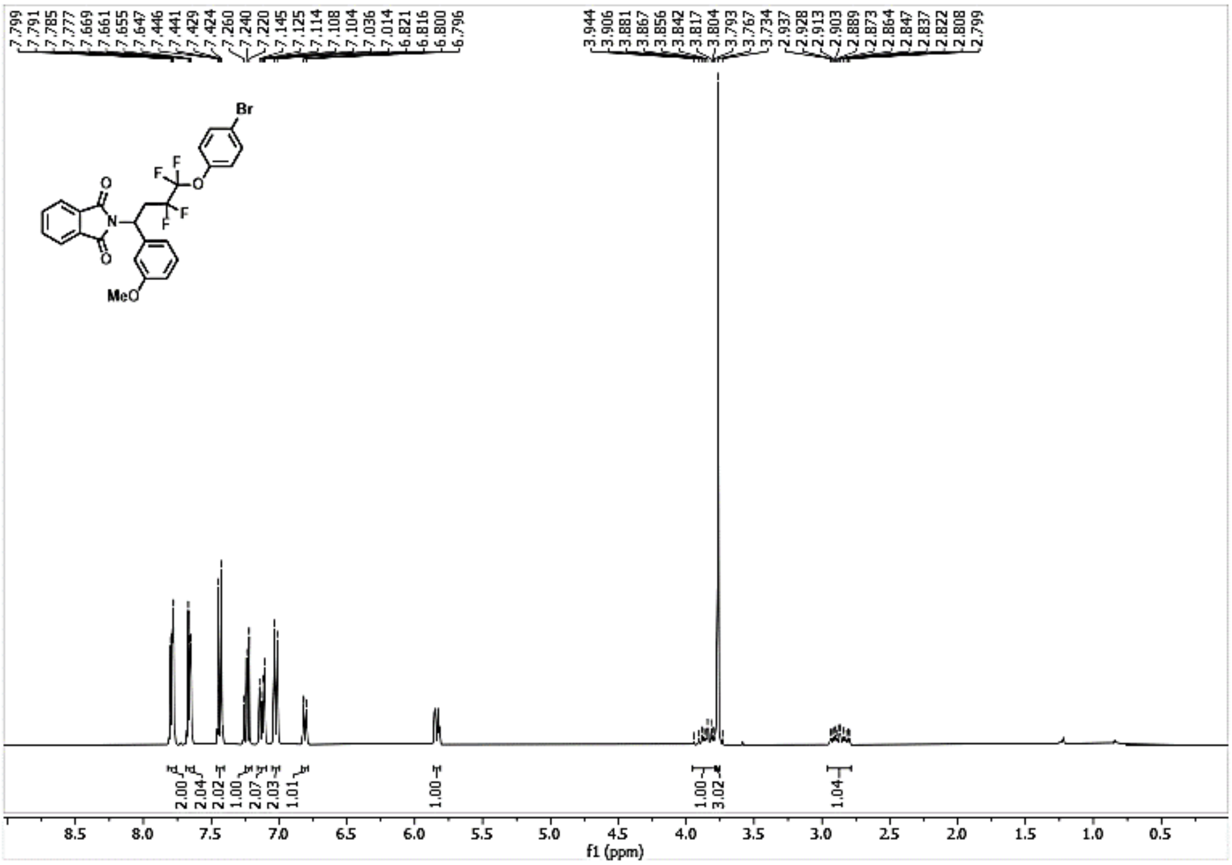

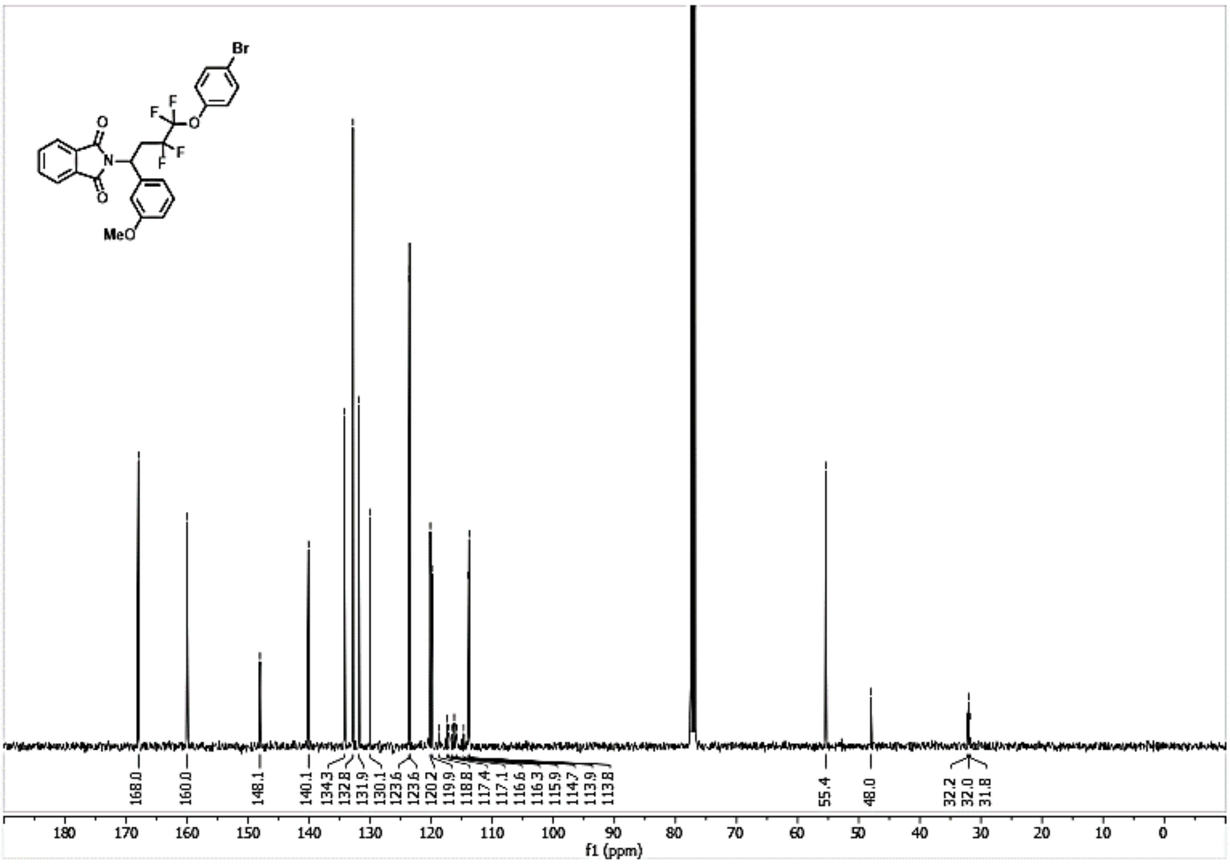

Compound 5f. ^{19}F NMR ($CDCl_3$, 376 MHz).

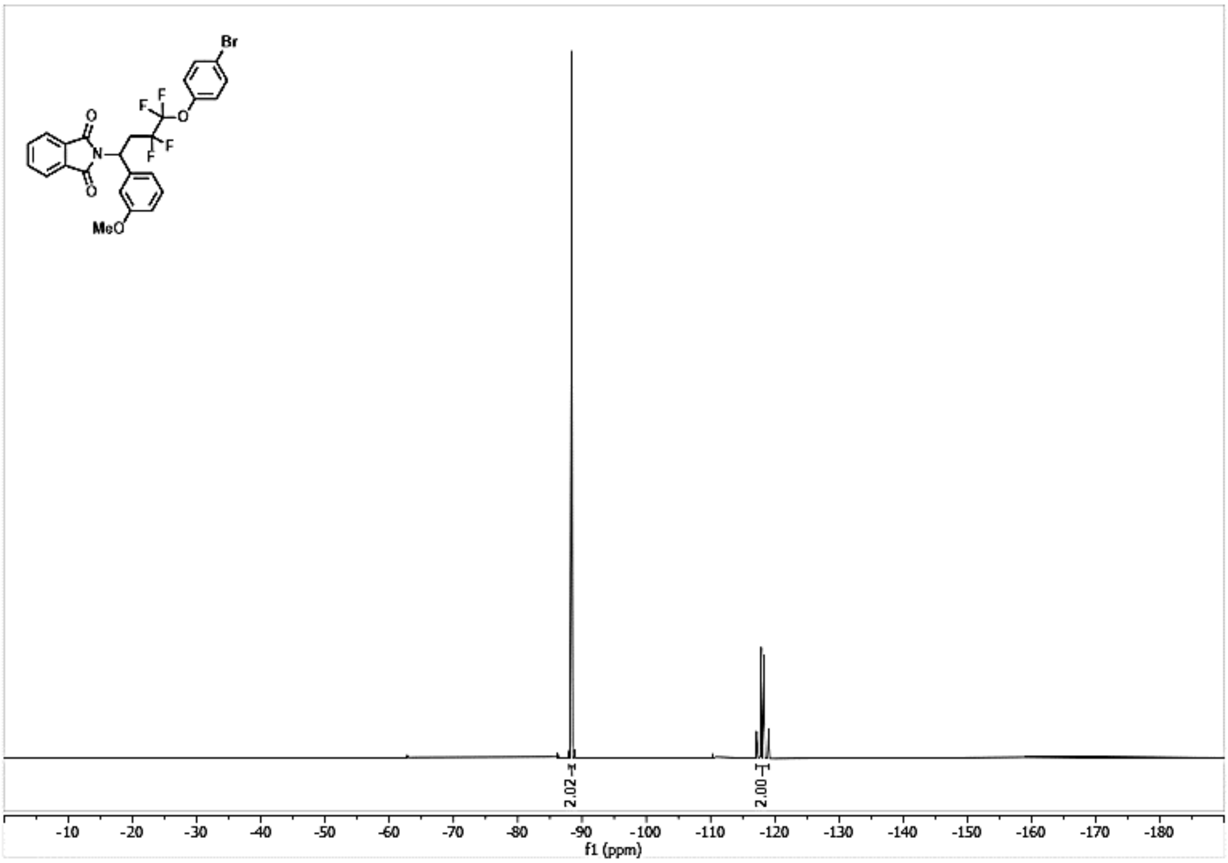

Compound 5g. Top: ^{1}H NMR ($CDCl_3$, 400 MHz). Bottom: ^{13}C NMR ($CDCl_3$, 100 MHz).

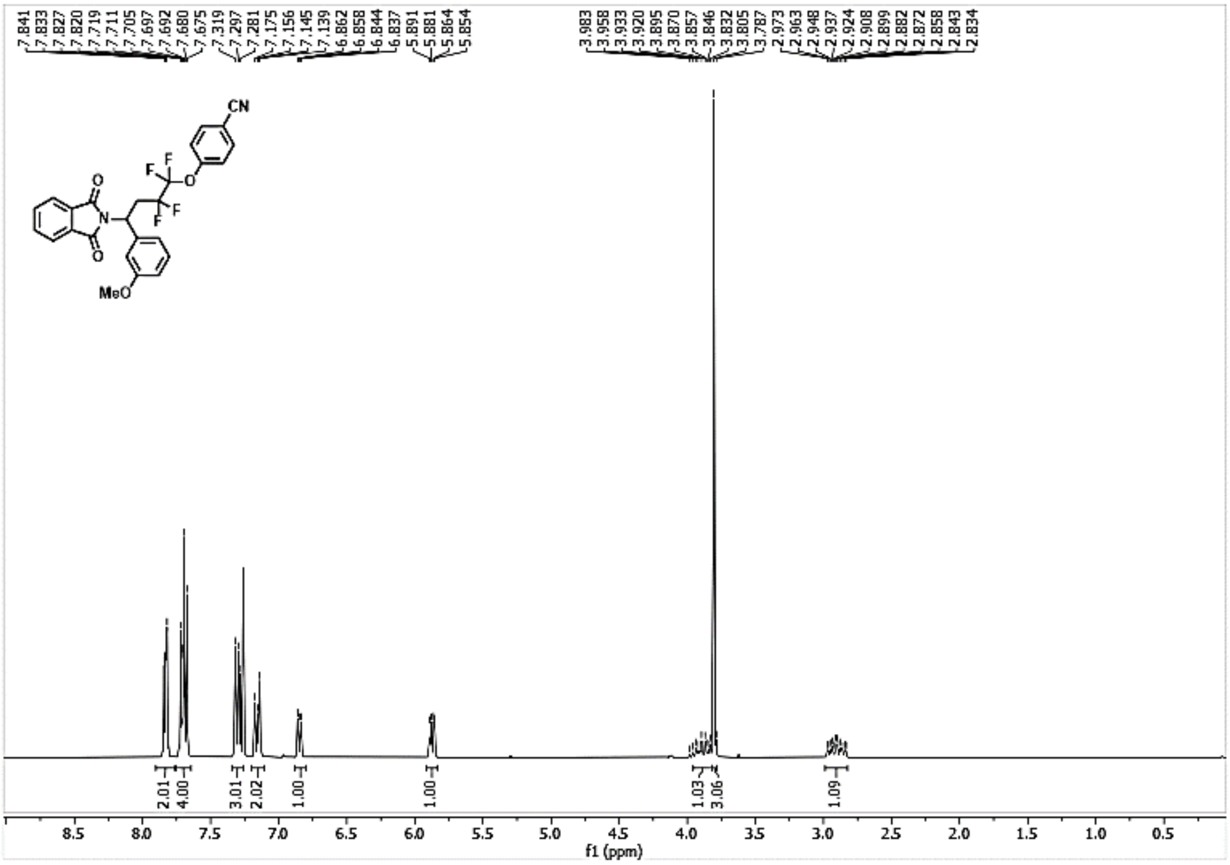

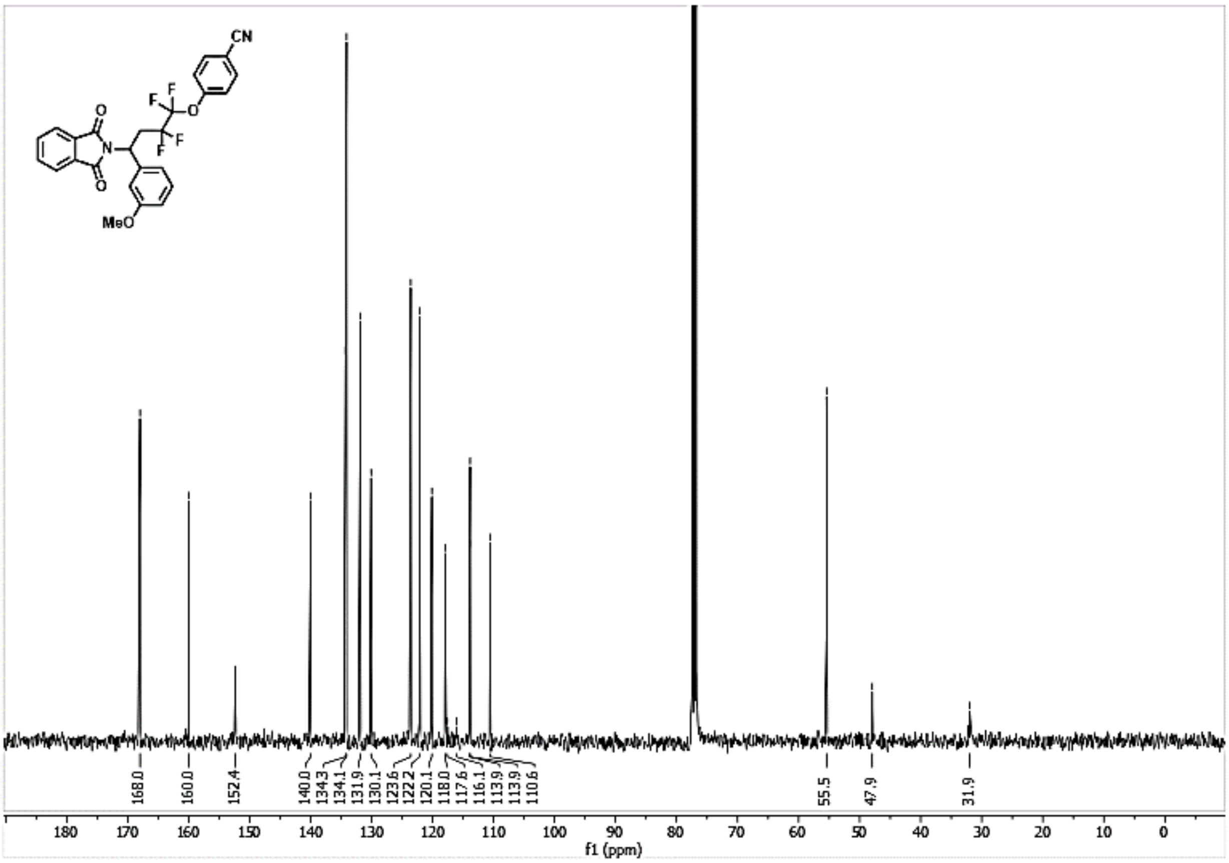

Compound 5g. ^{19}F NMR ($CDCl_3$, 376 MHz).

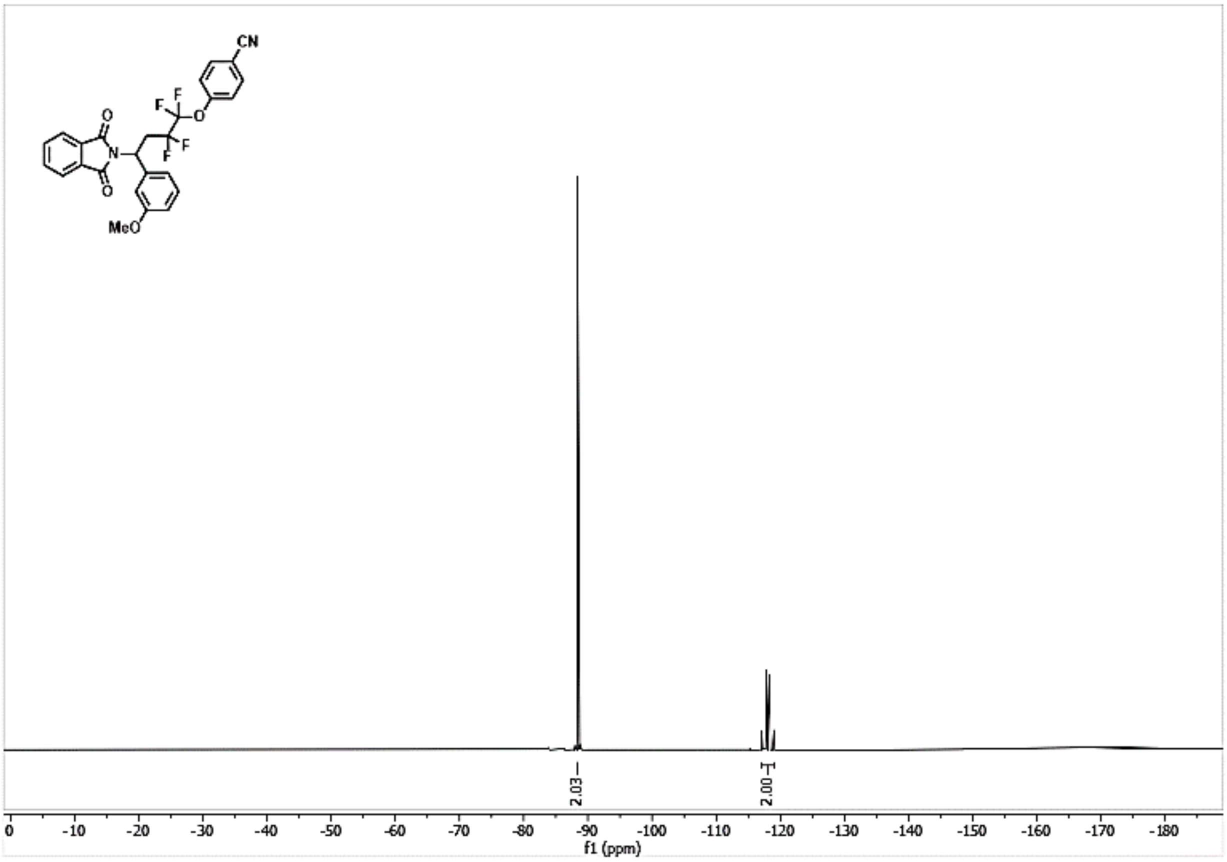

Compound 5h. Top: ^{1}H NMR ($CDCl_3$, 400 MHz). Bottom: ^{13}C NMR ($CDCl_3$, 100 MHz).

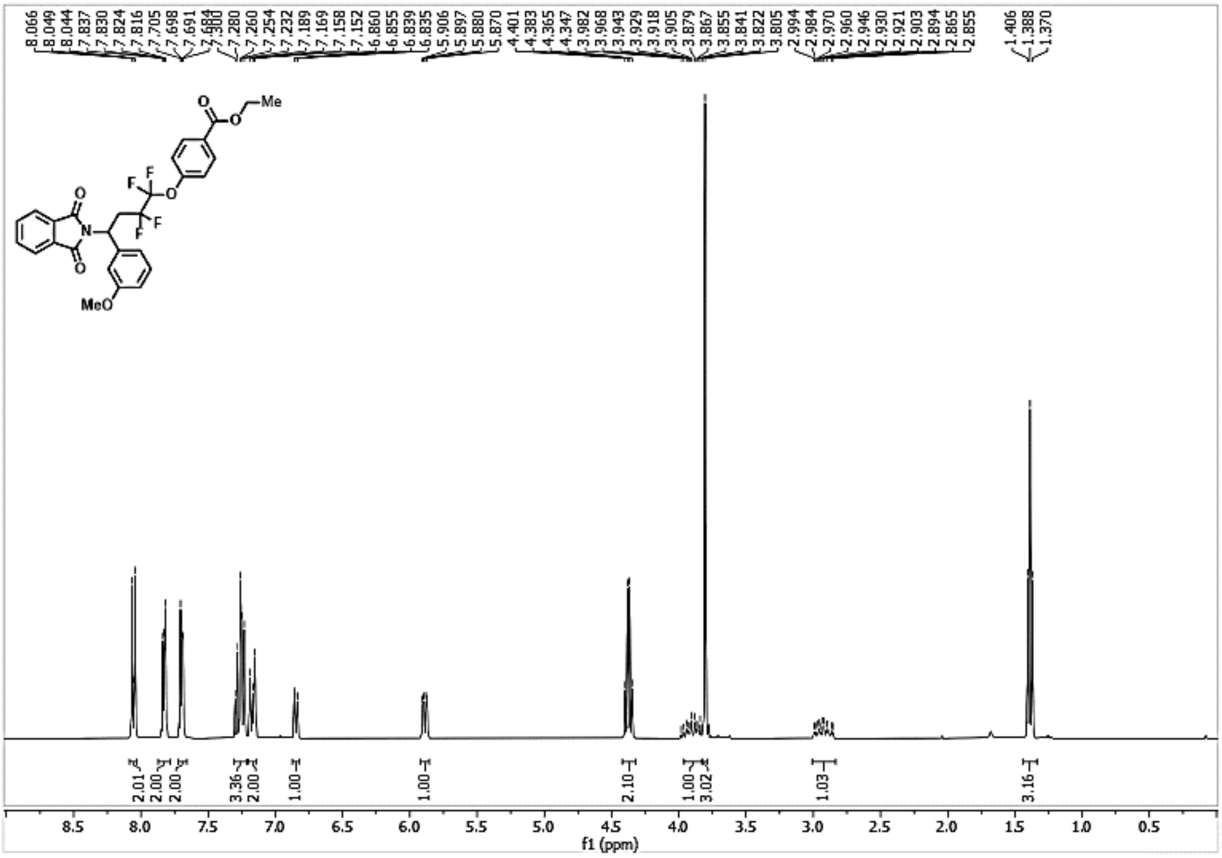

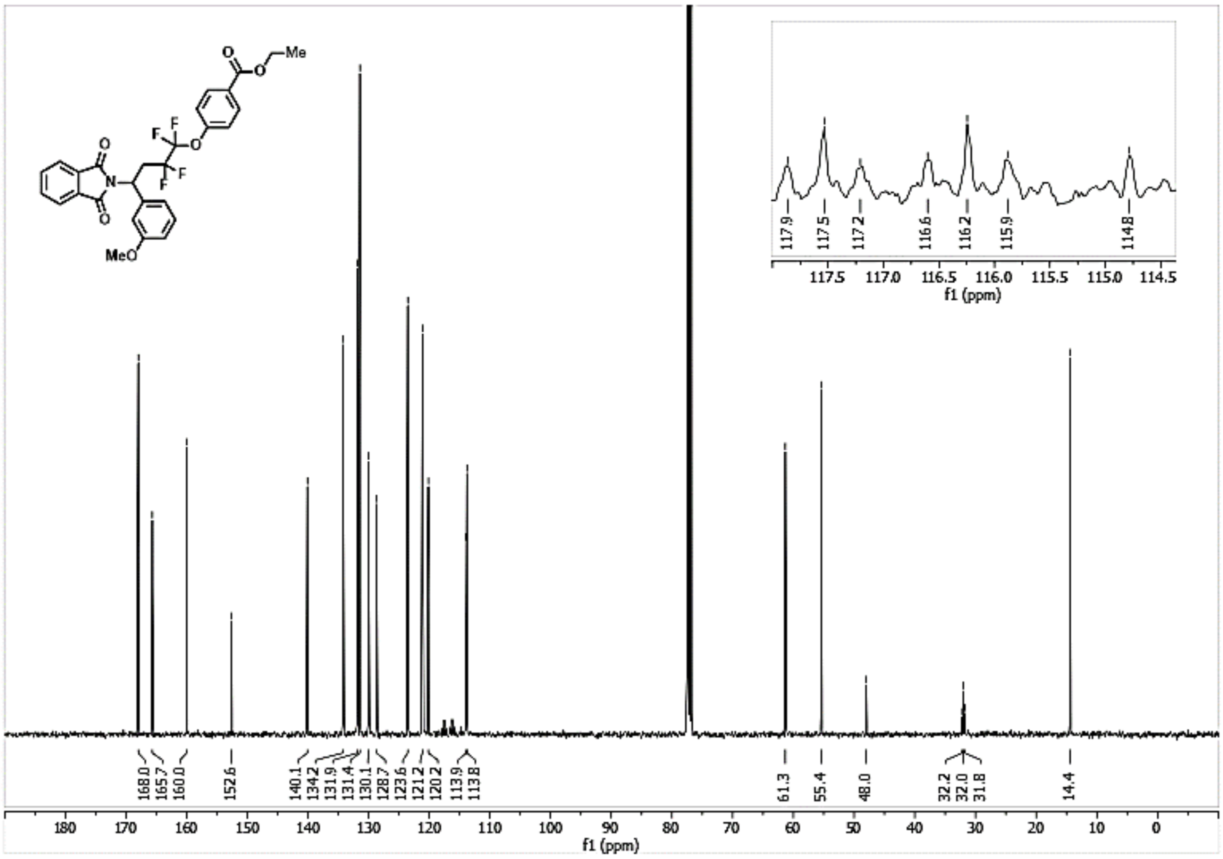

Compound 5h. ^{19}F NMR ($CDCl_3$, 376 MHz).

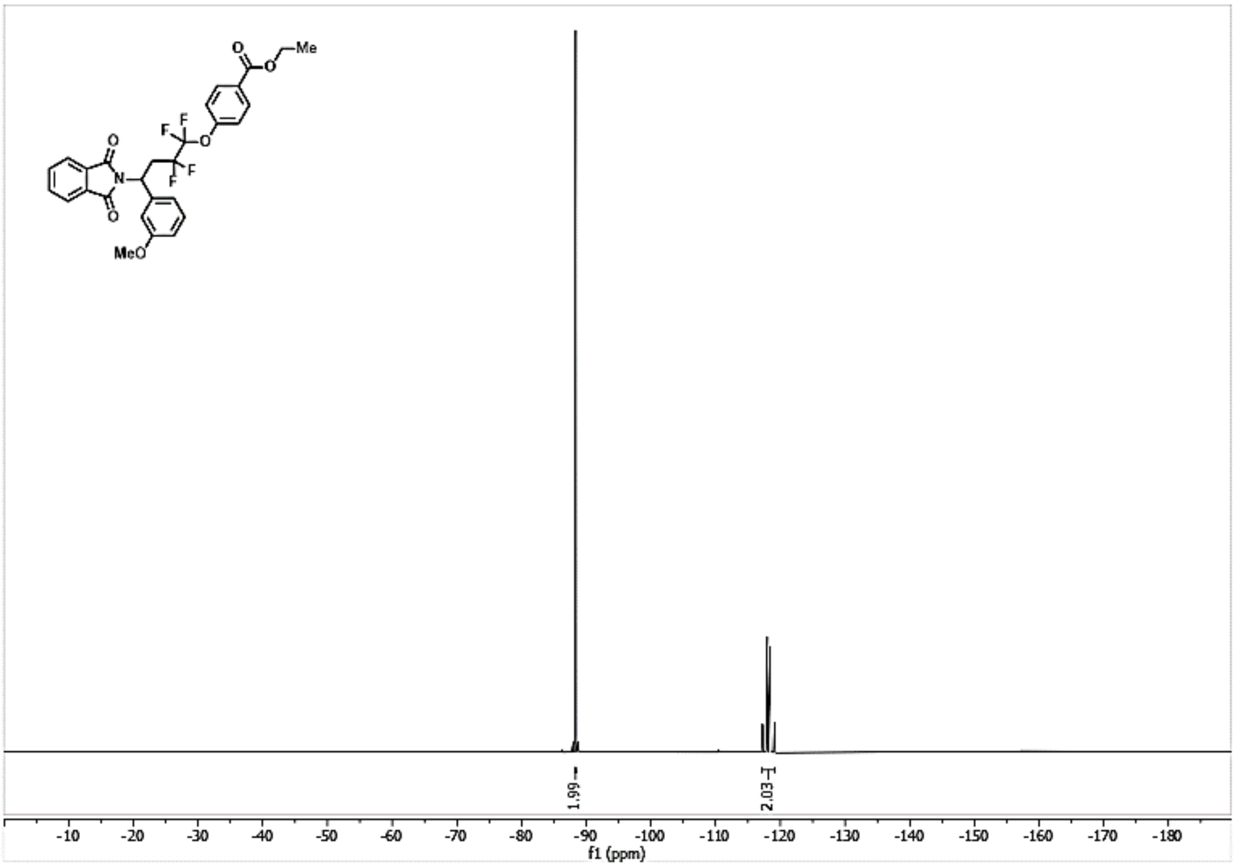

Compound 5i. Top: ^{1}H NMR ($CDCl_3$, 400 MHz). Bottom: ^{13}C NMR ($CDCl_3$, 100 MHz).

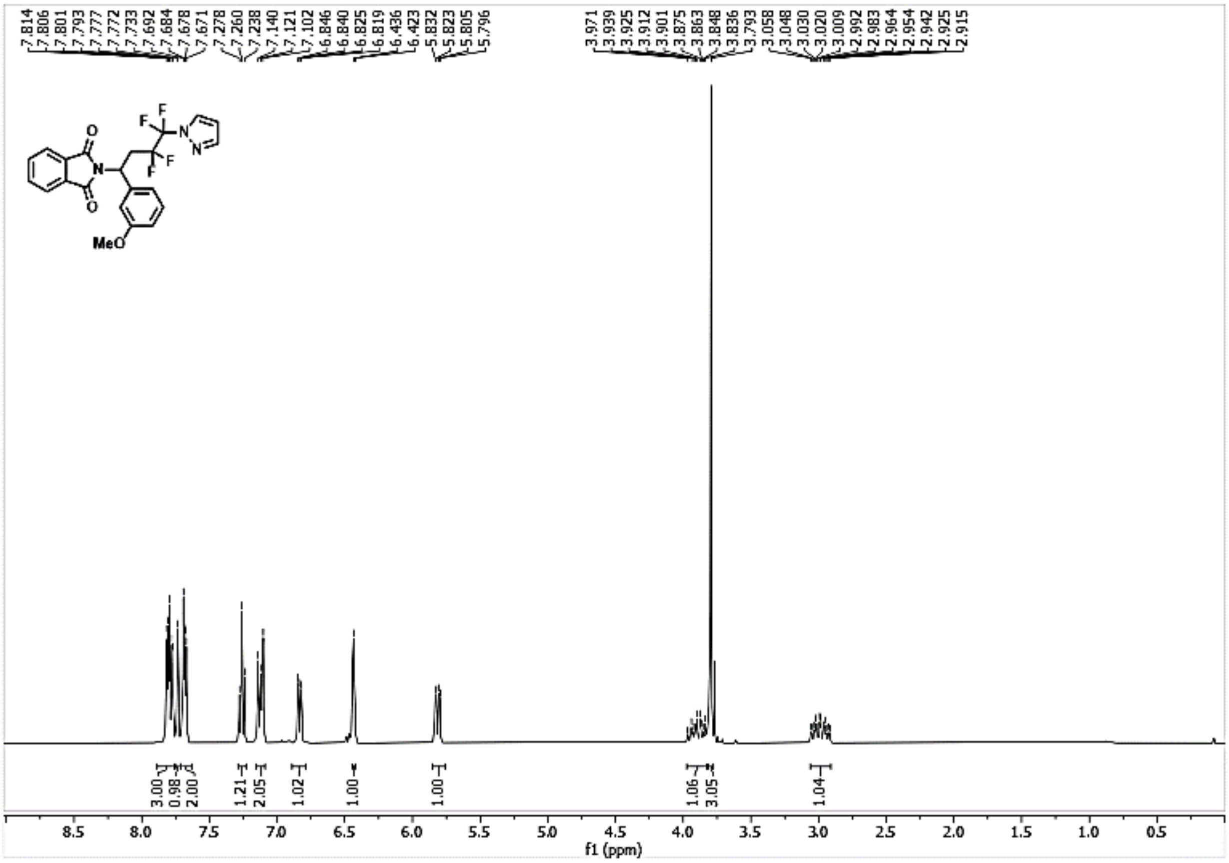

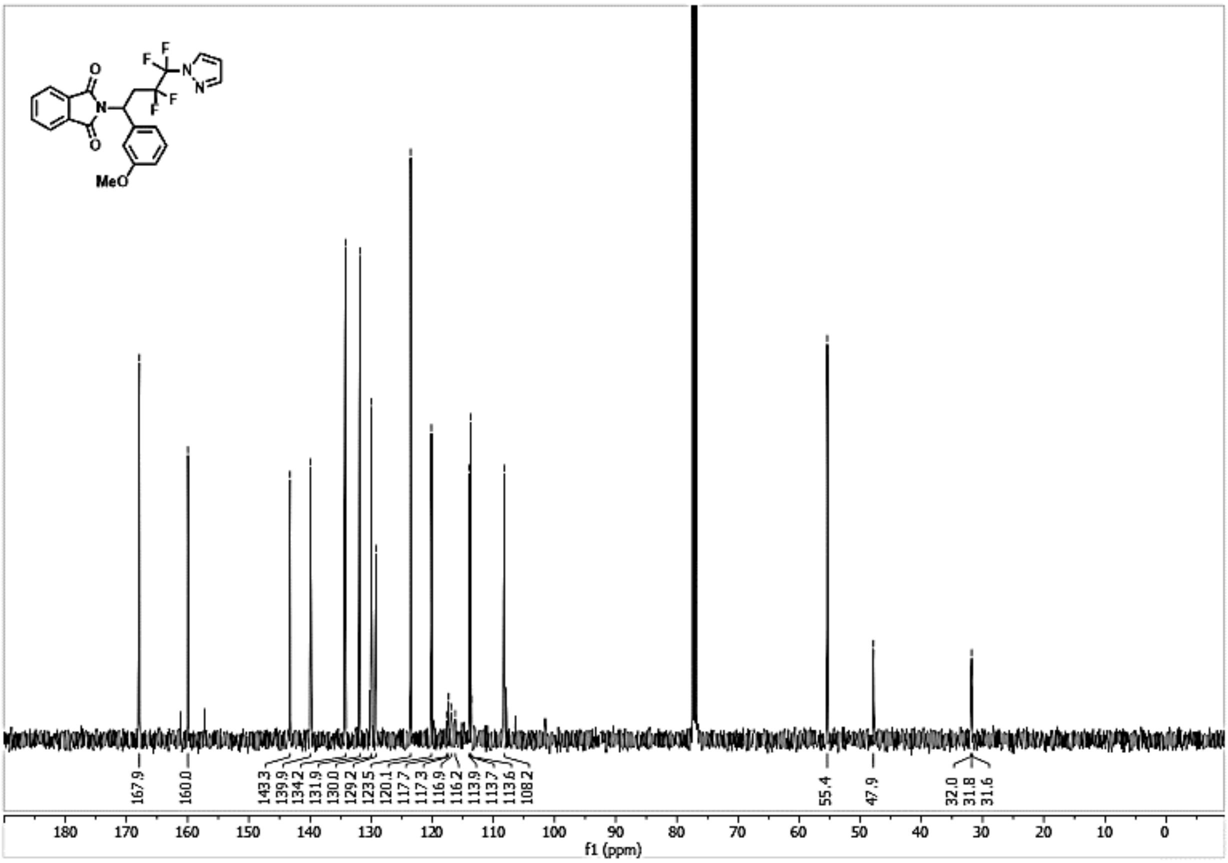

Compound 5i. ^{19}F NMR ($CDCl_3$, 376 MHz).

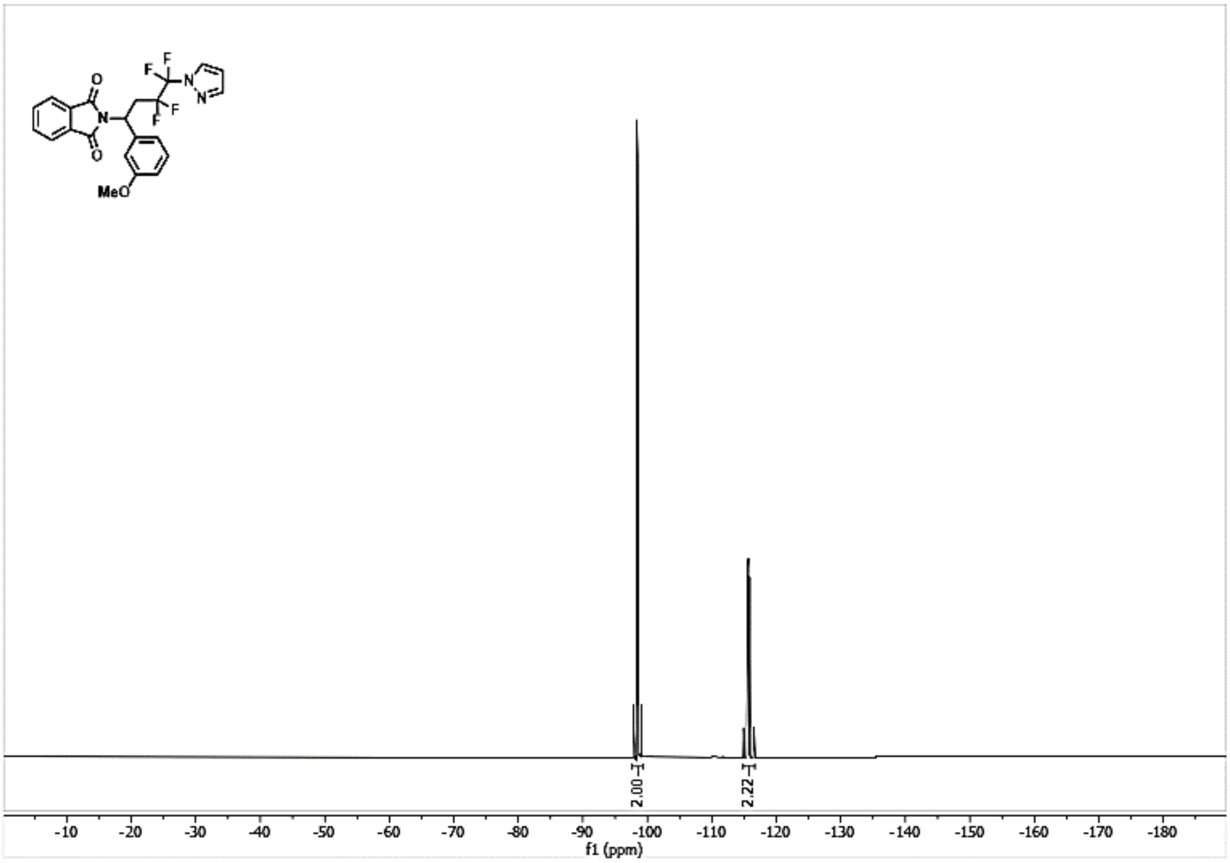

Compound 5j. Top: ^{1}H NMR ($CDCl_3$, 400 MHz). Bottom: ^{13}C NMR ($CDCl_3$, 100 MHz).

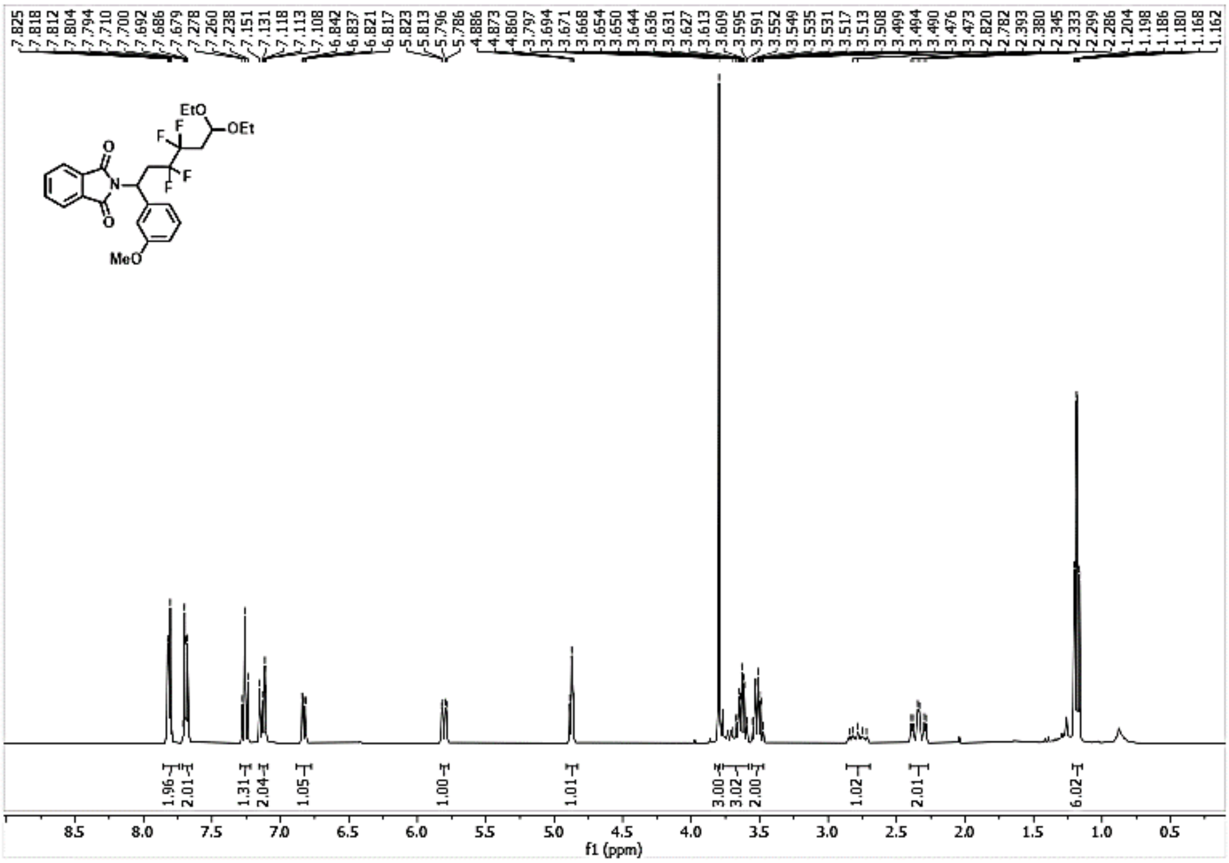

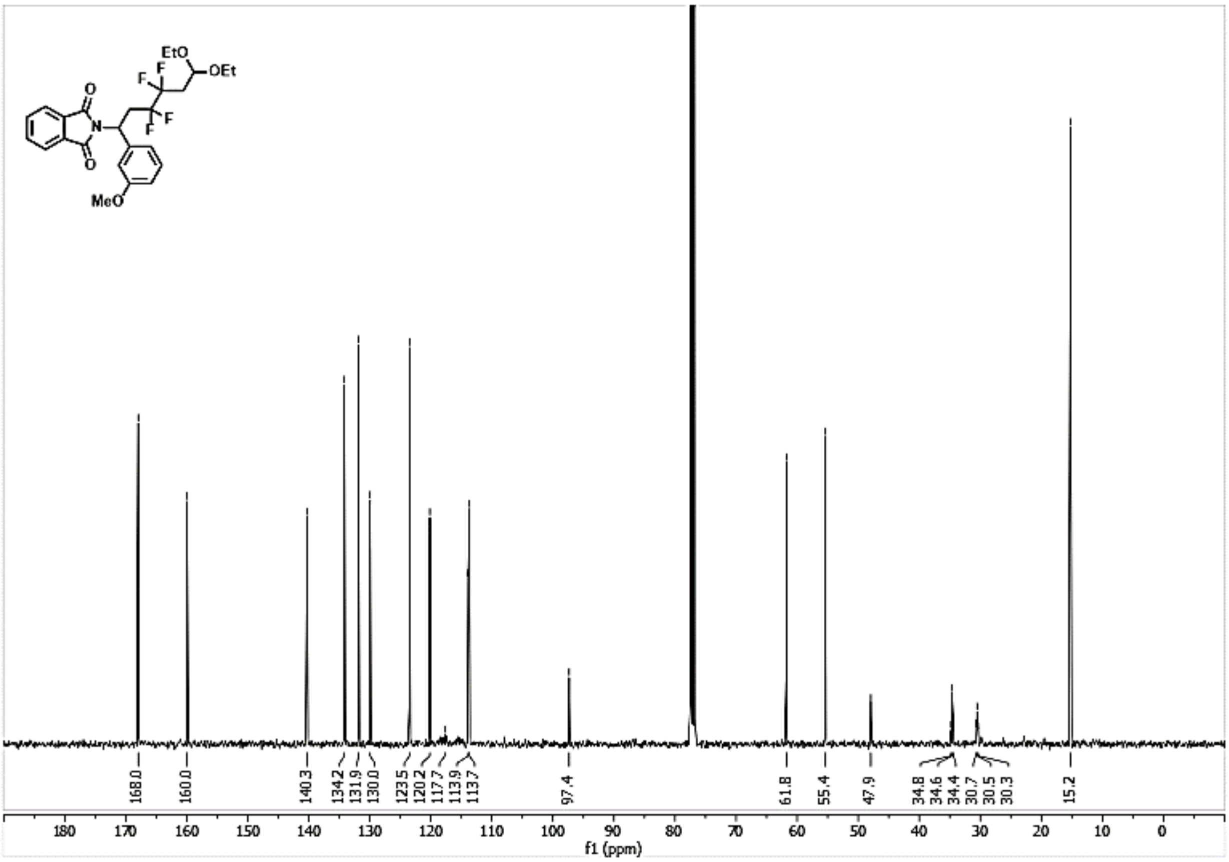

Compound 5j. ^{19}F NMR ($CDCl_3$, 376 MHz).

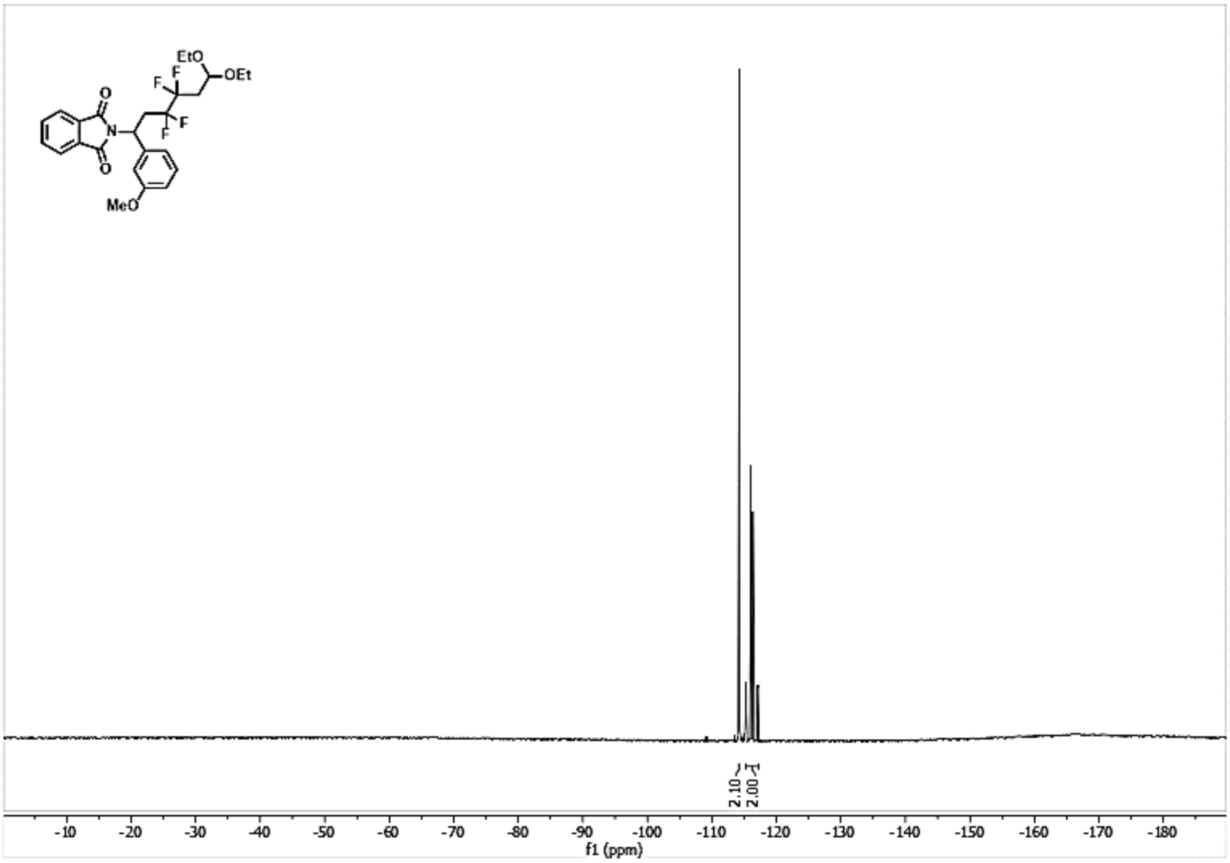

Compound 5k. Top: ^{1}H NMR ($CDCl_3$, 400 MHz). Bottom: ^{13}C NMR ($CDCl_3$, 100 MHz).

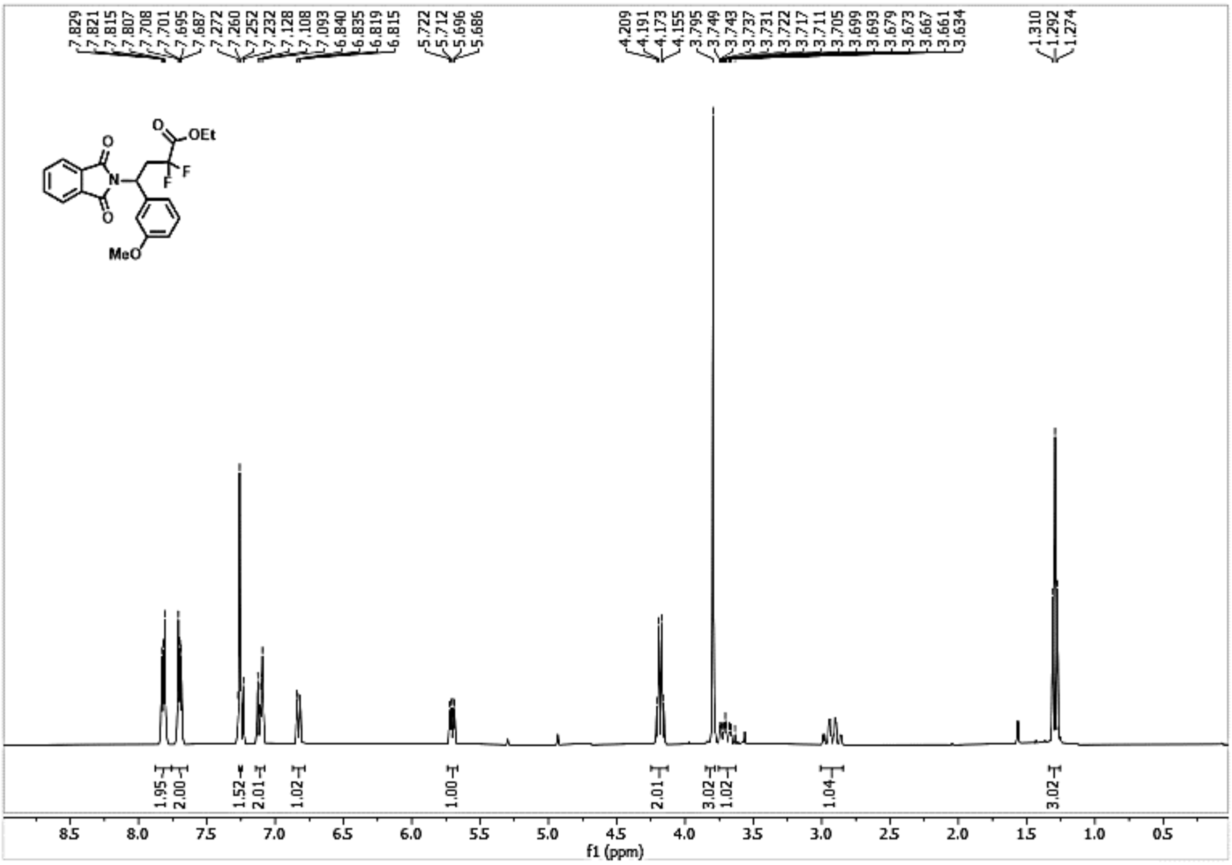

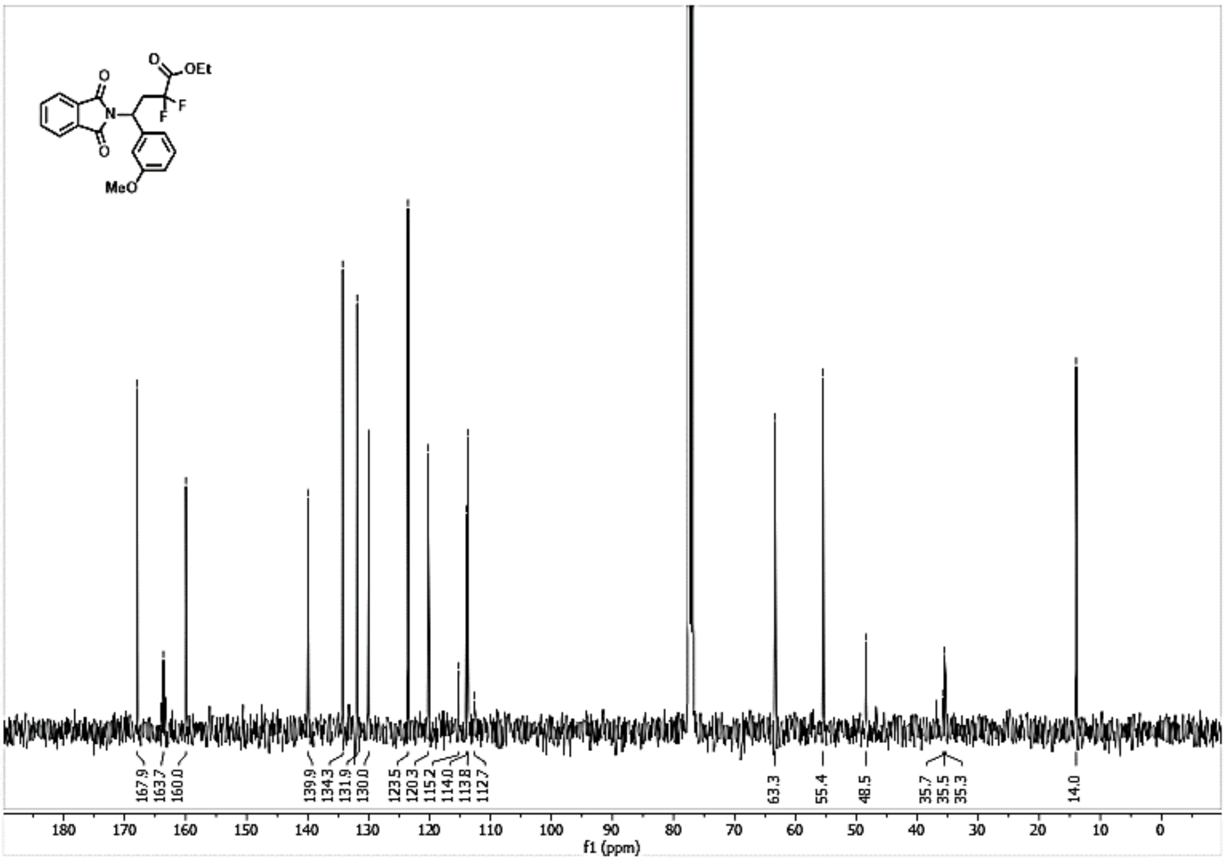

Compound 5k. ^{19}F NMR ($CDCl_3$, 376 MHz).

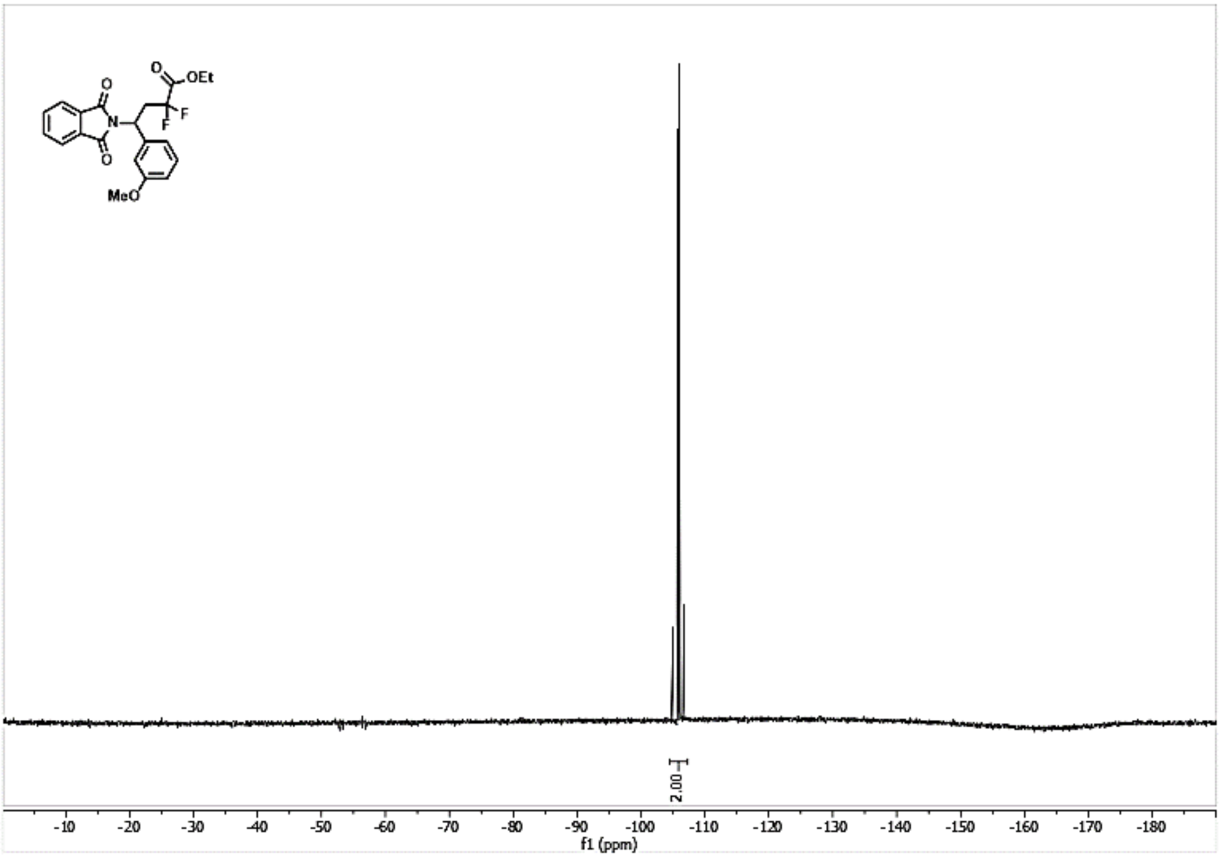

Compound 5l. Top: ^1H NMR ($CDCl_3$, 400 MHz). Bottom: ^{13}C NMR ($CDCl_3$, 100 MHz).

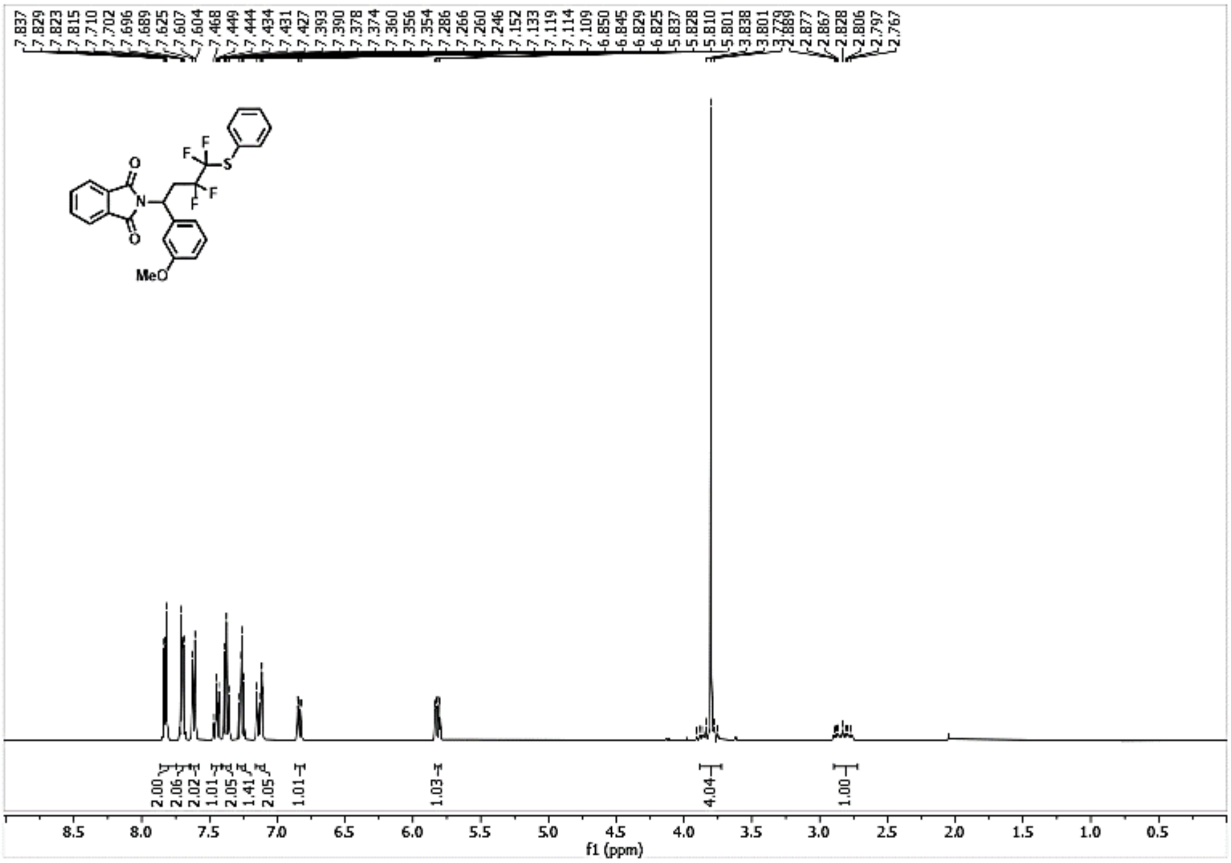

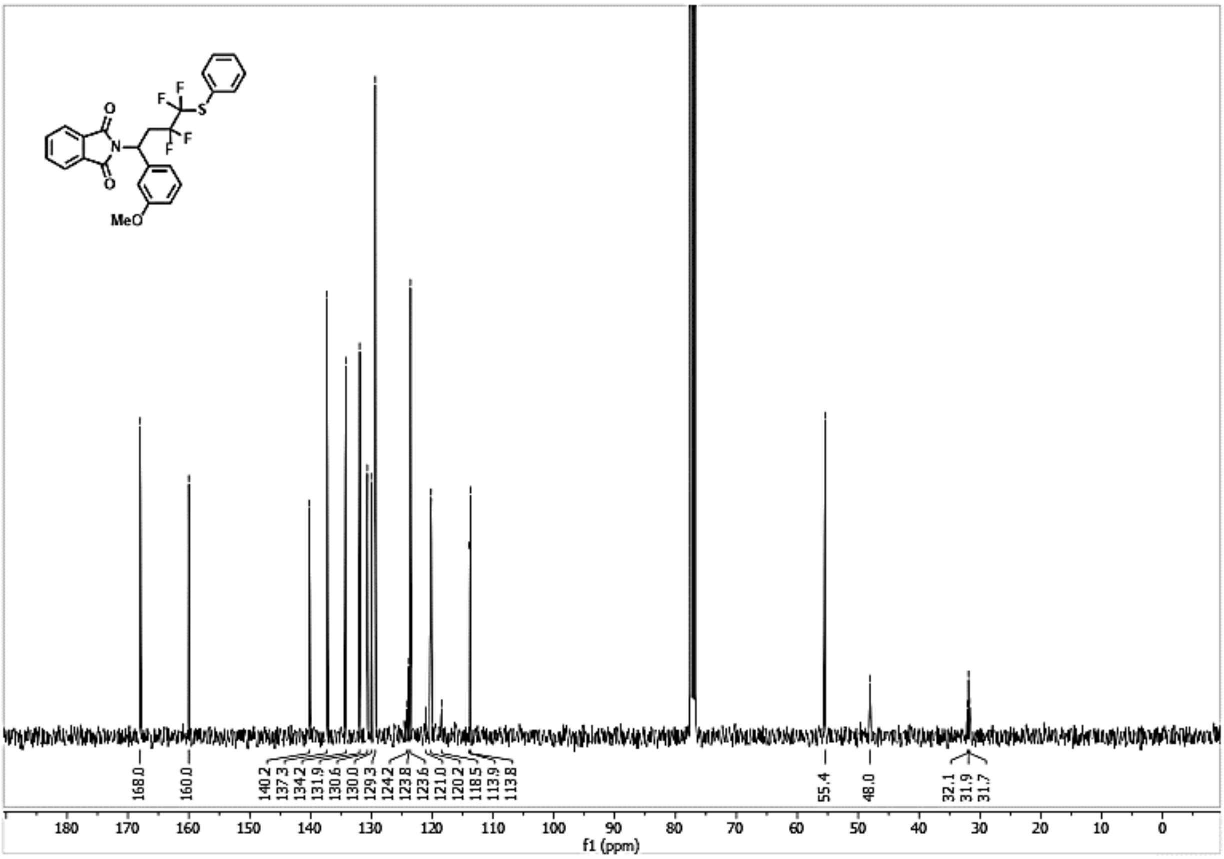

Compound 5l. ^{19}F NMR ($CDCl_3$, 376 MHz).

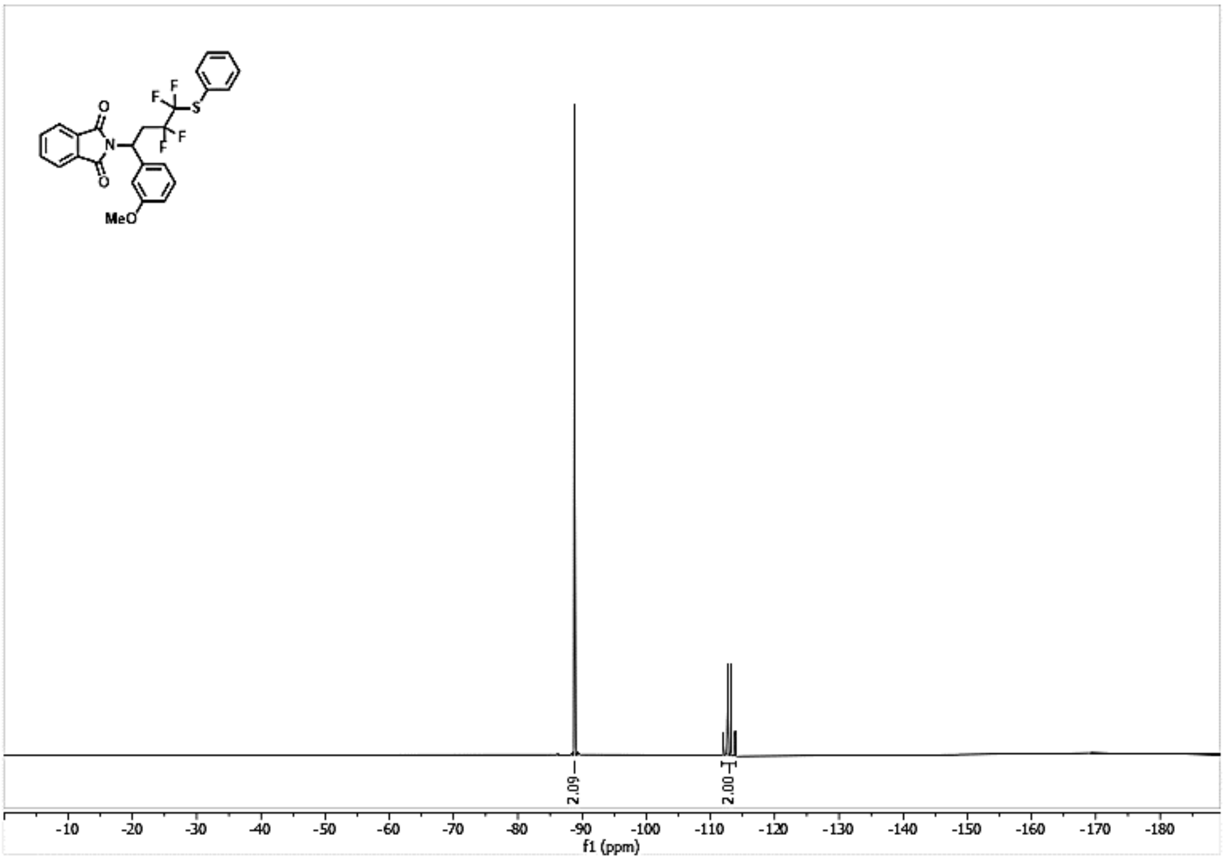

Compound 5m. Top: ^{1}H NMR ($CDCl_3$, 400 MHz). Bottom: ^{13}C NMR ($CDCl_3$, 100 MHz).

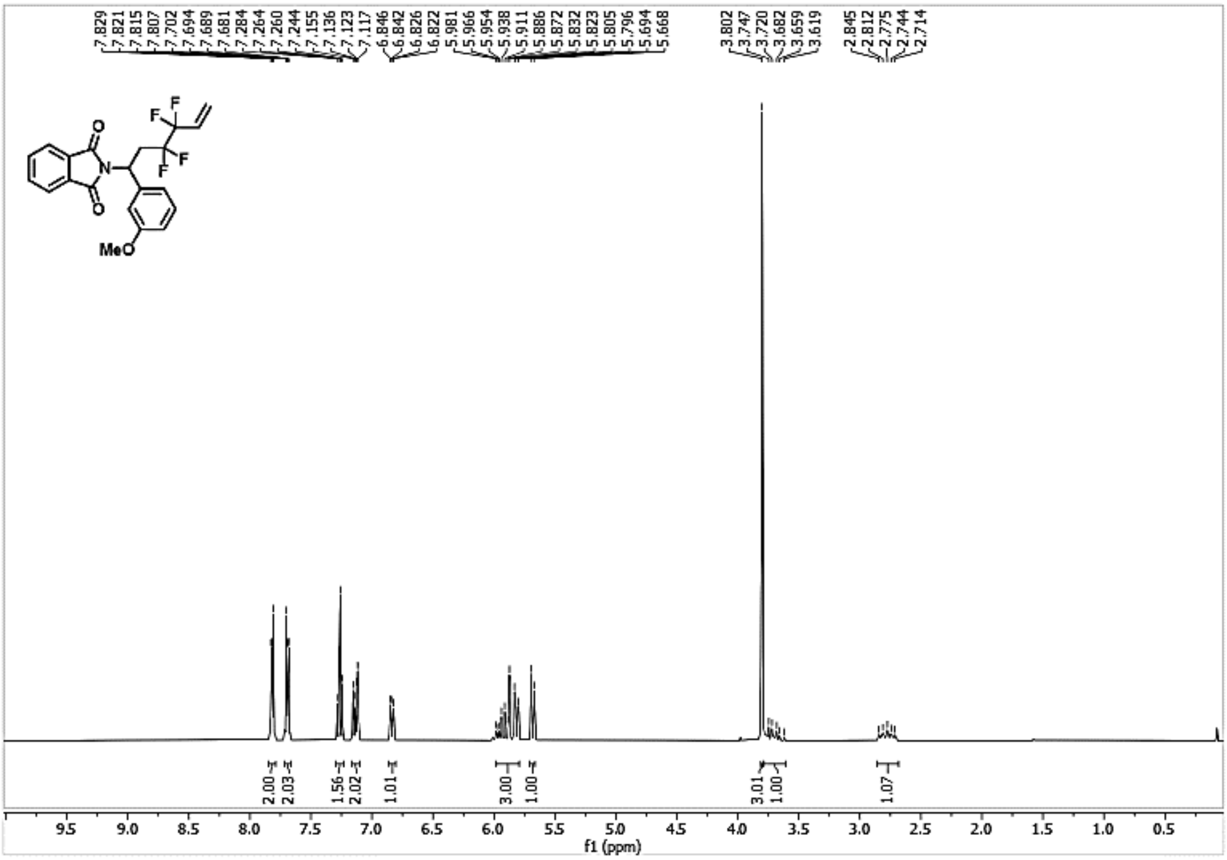

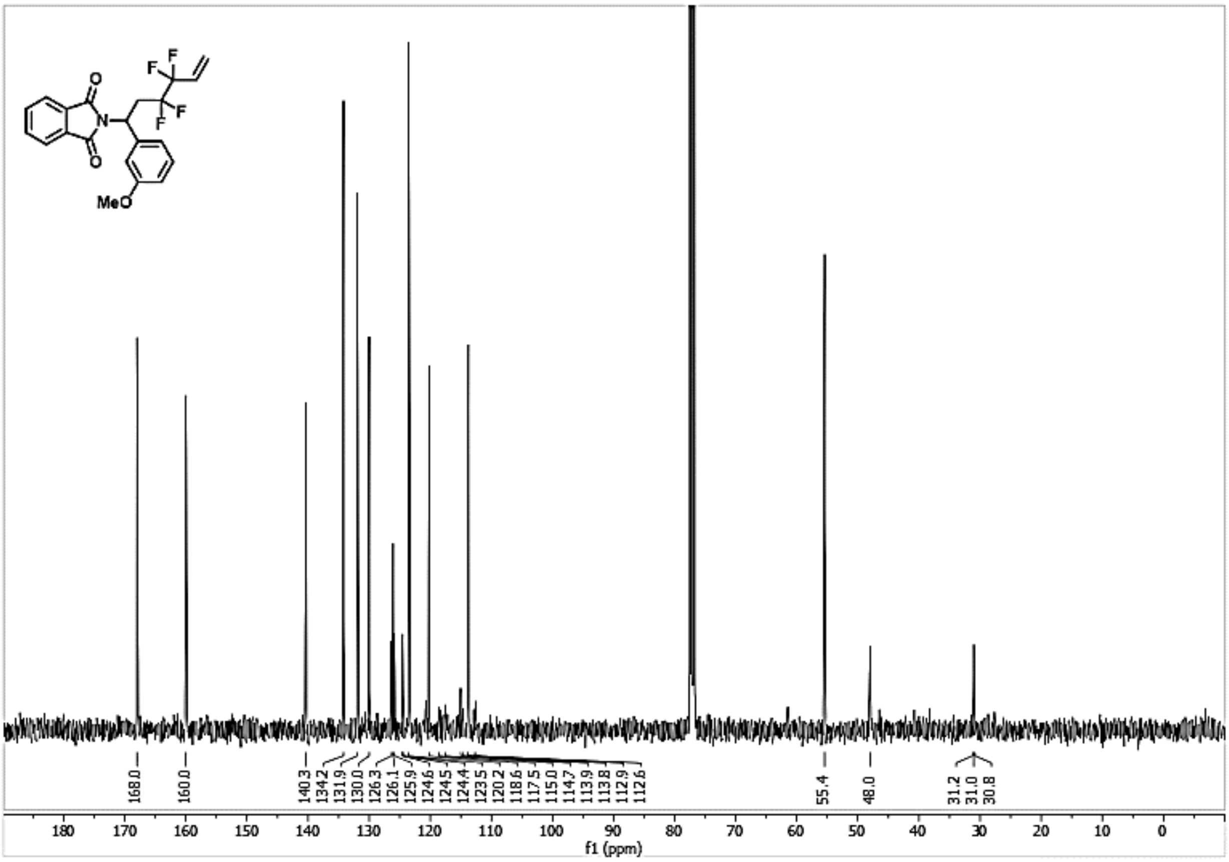

Compound 5m. ^{19}F NMR ($CDCl_3$, 376 MHz).

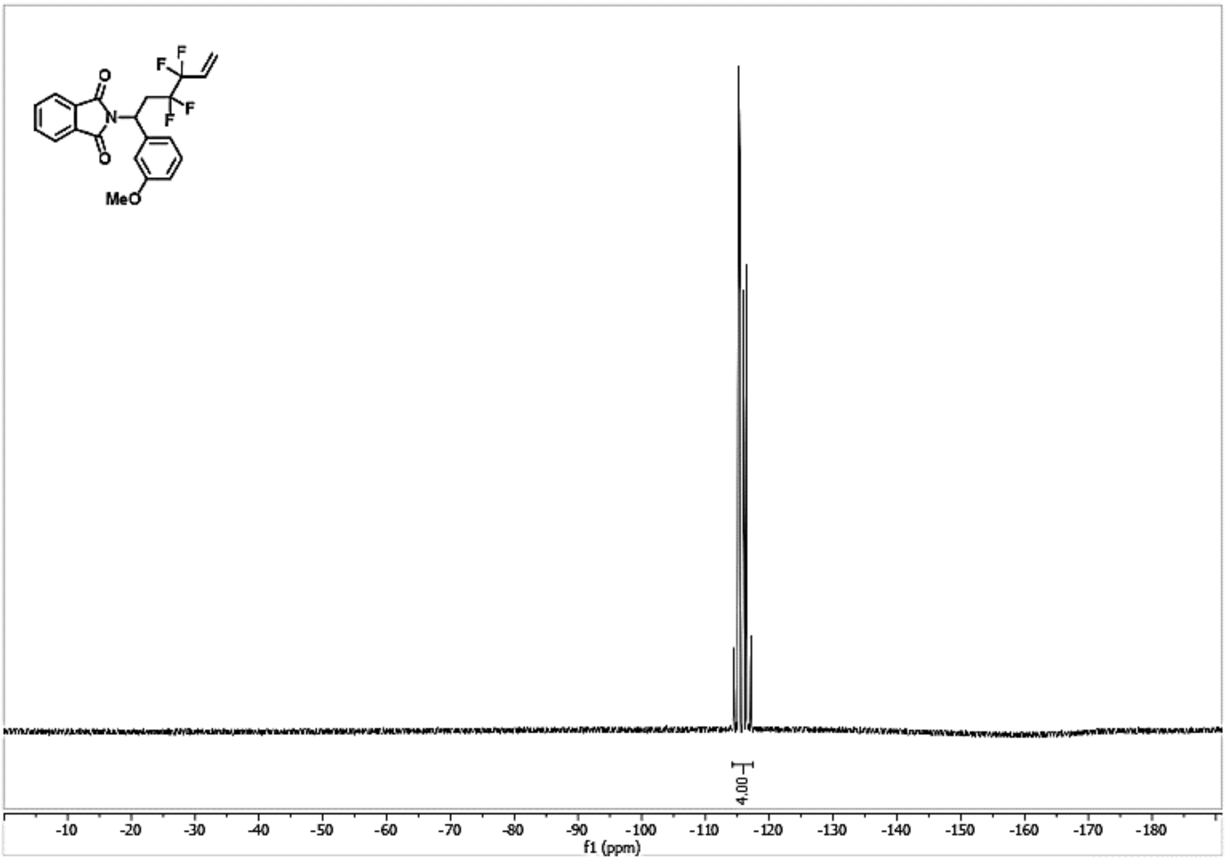

Compound 5n. Top: ^{1}H NMR ($CDCl_3$, 400 MHz). Bottom: ^{13}C NMR ($CDCl_3$, 100 MHz).

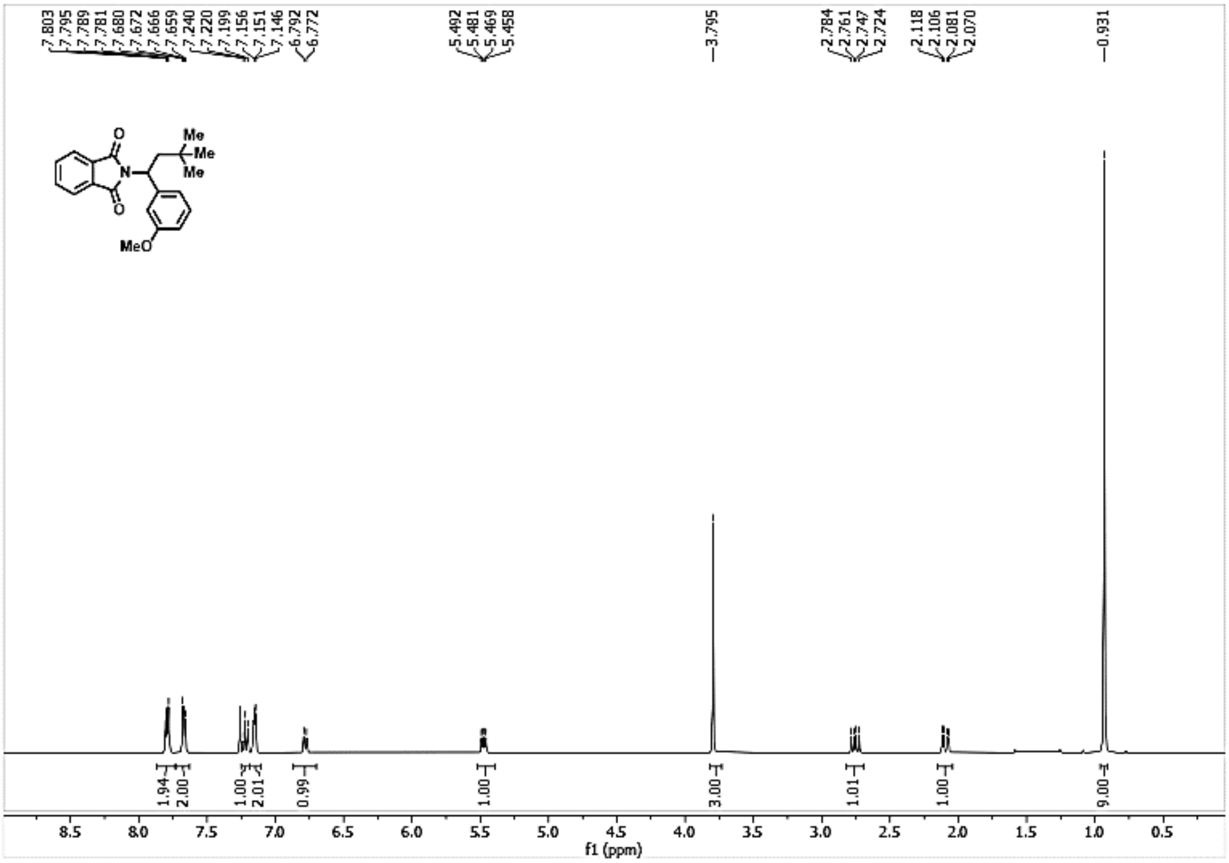

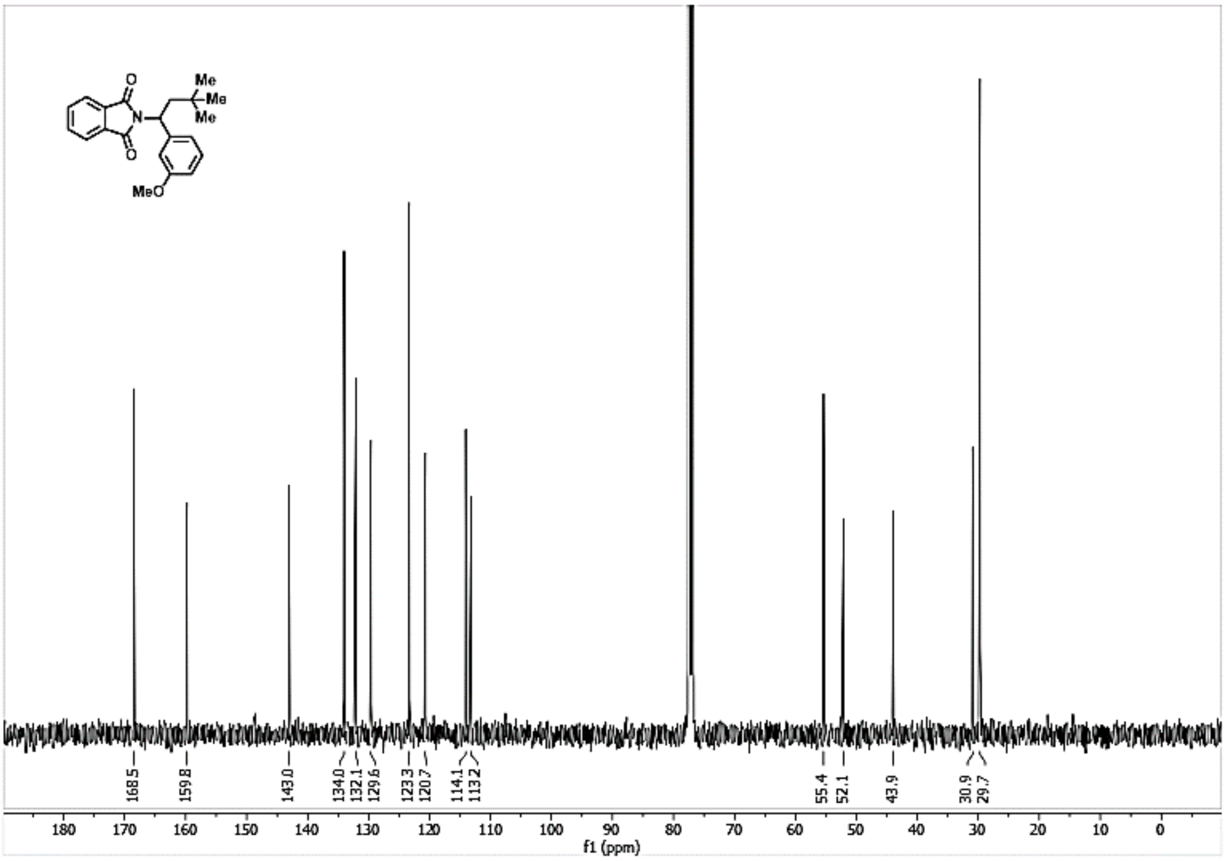

Compound 6a. Top: ^{1}H NMR ($CDCl_3$, 400 MHz). Bottom: ^{13}C NMR ($CDCl_3$, 100 MHz).

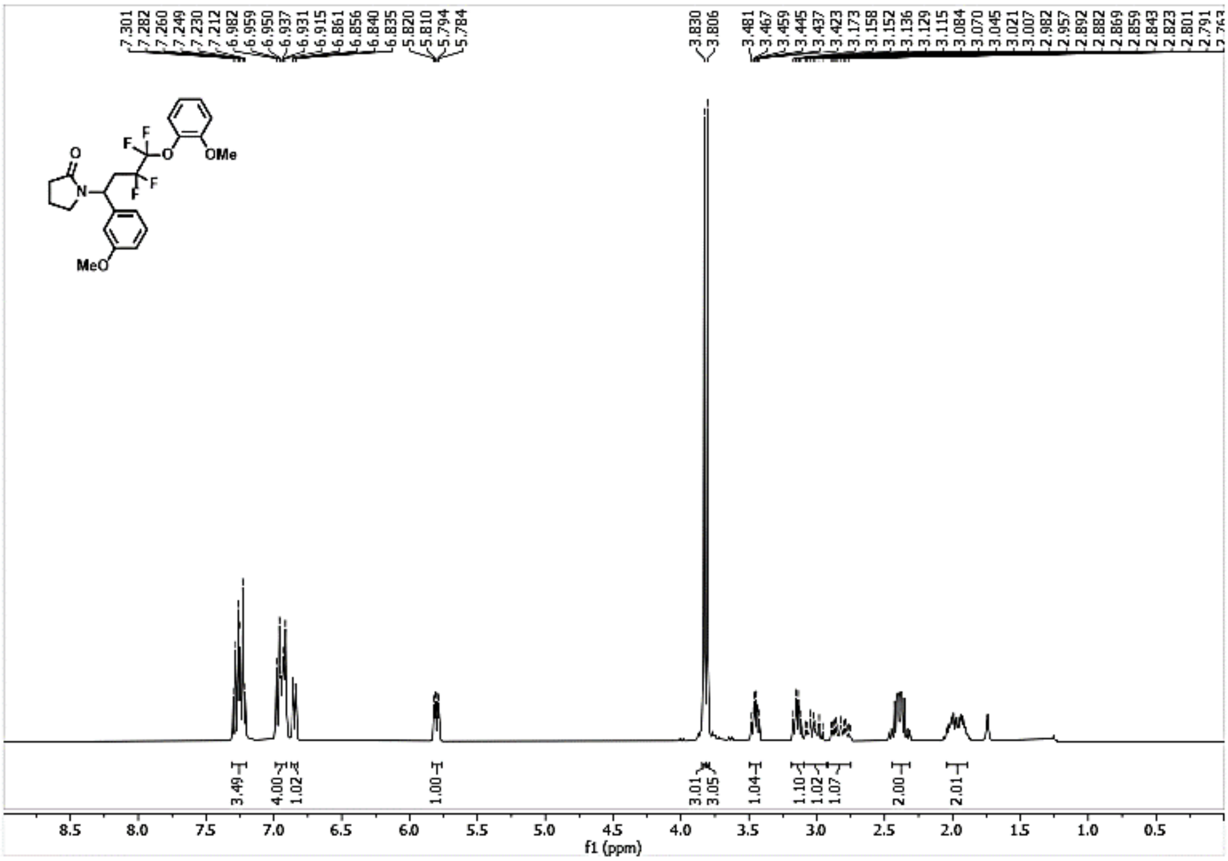

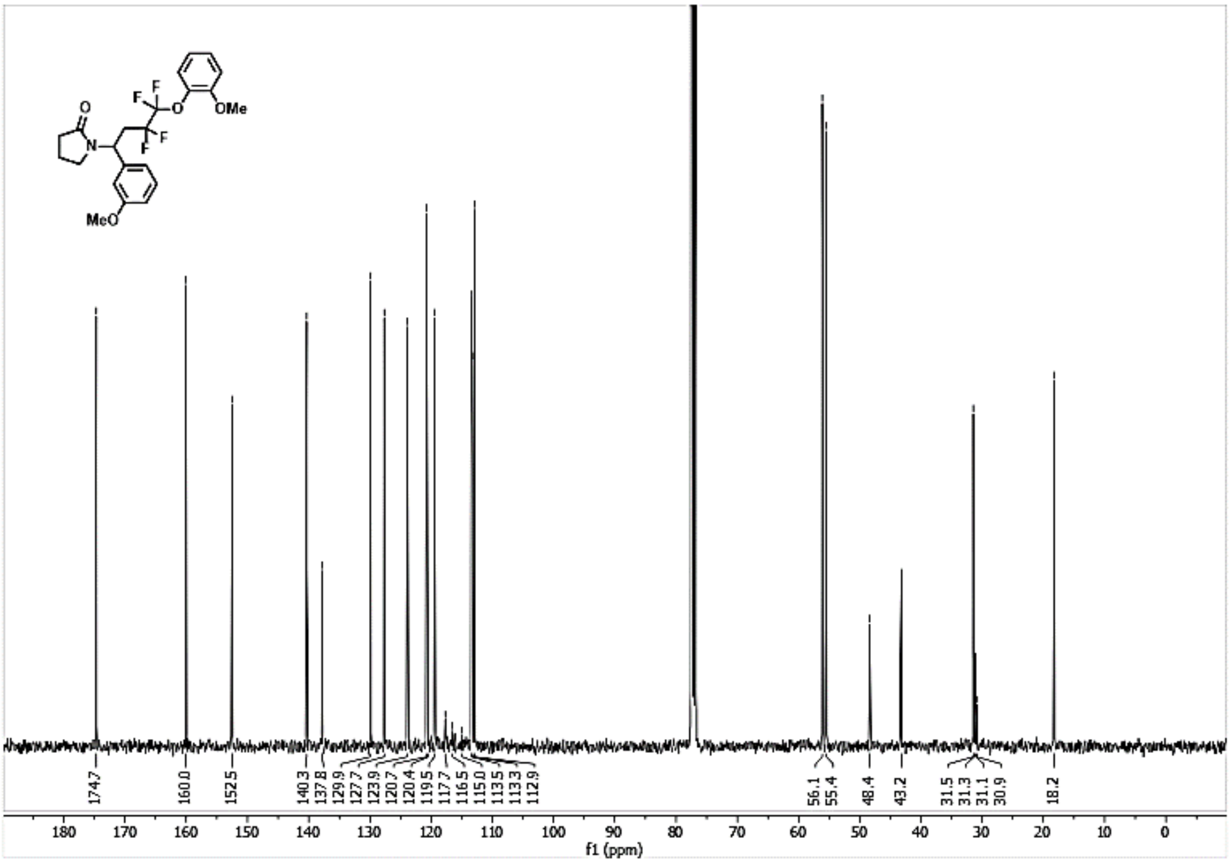

Compound 6a. ^{19}F NMR ($CDCl_3$, 376 MHz).

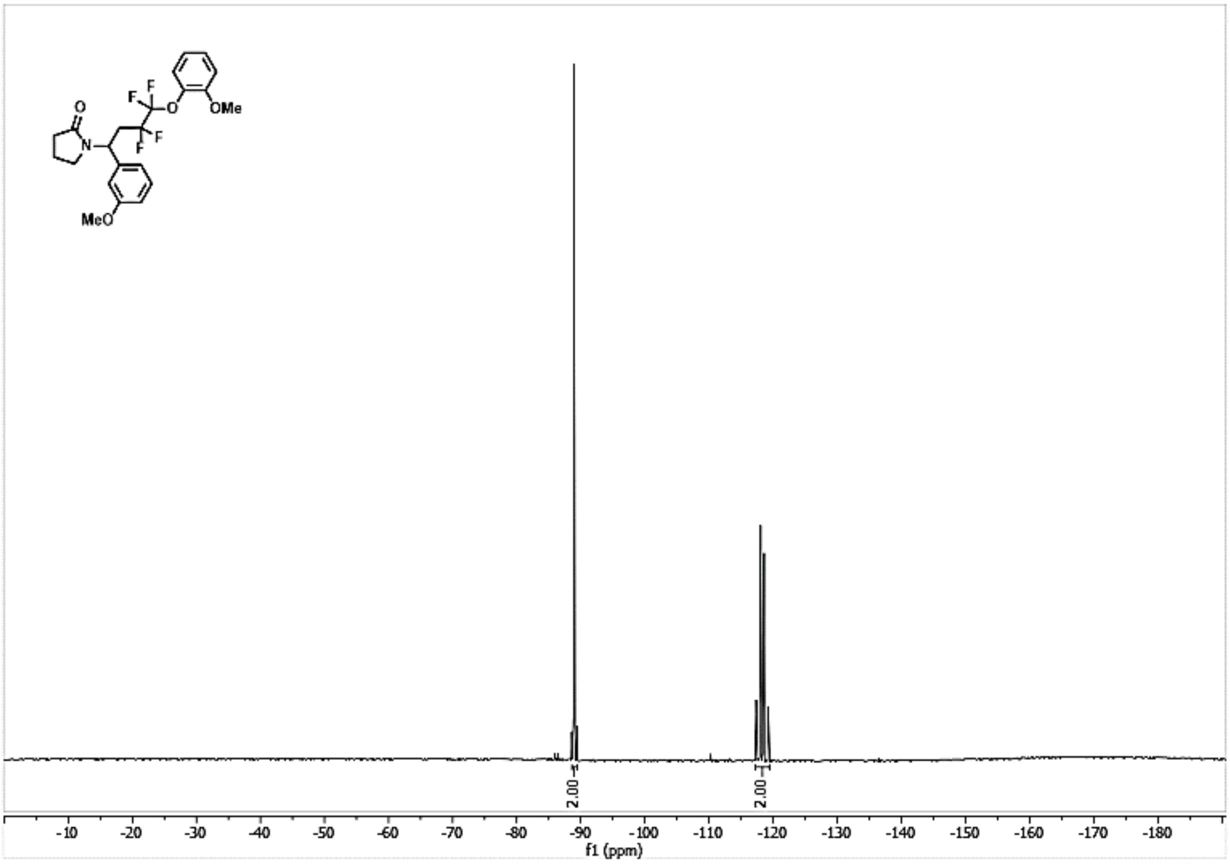

Compound 6b. Top: ^{1}H NMR ($CDCl_3$, 400 MHz). Bottom: ^{13}C NMR ($CDCl_3$, 100 MHz).

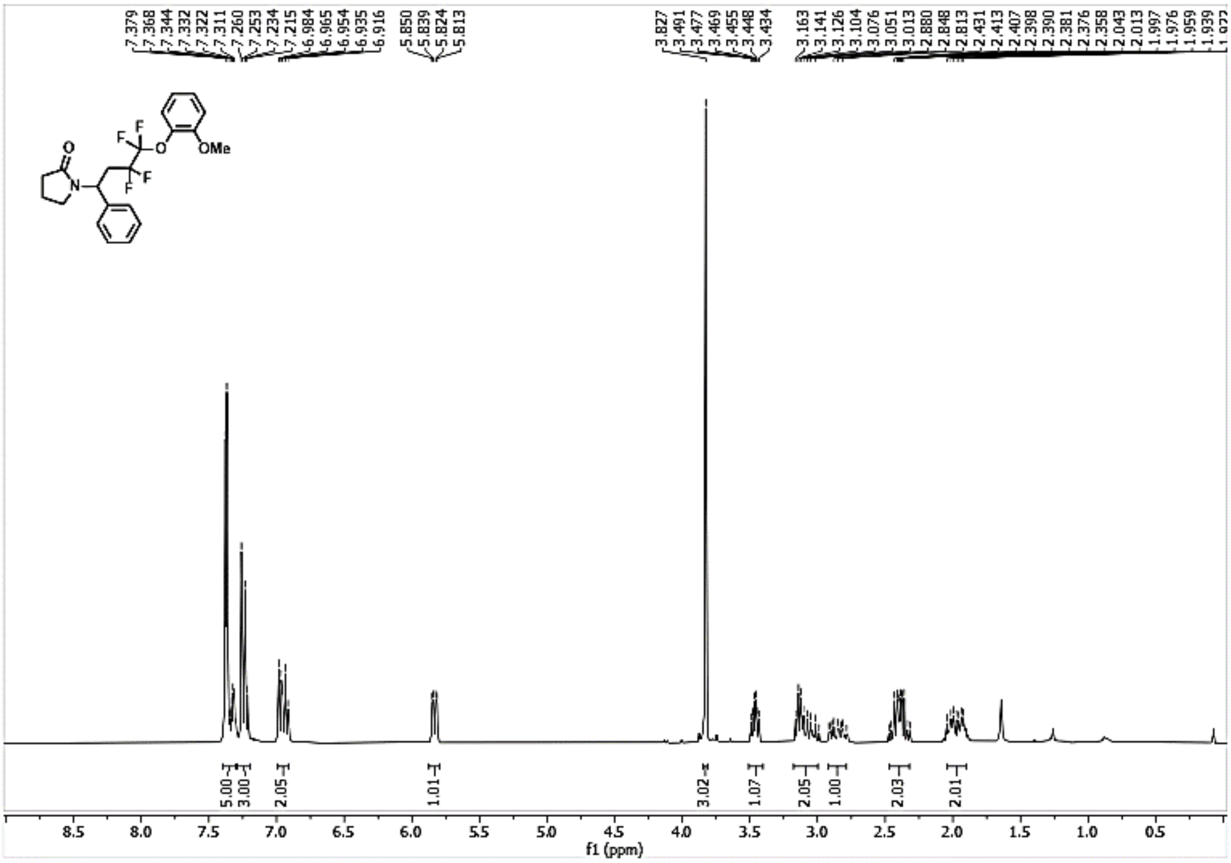

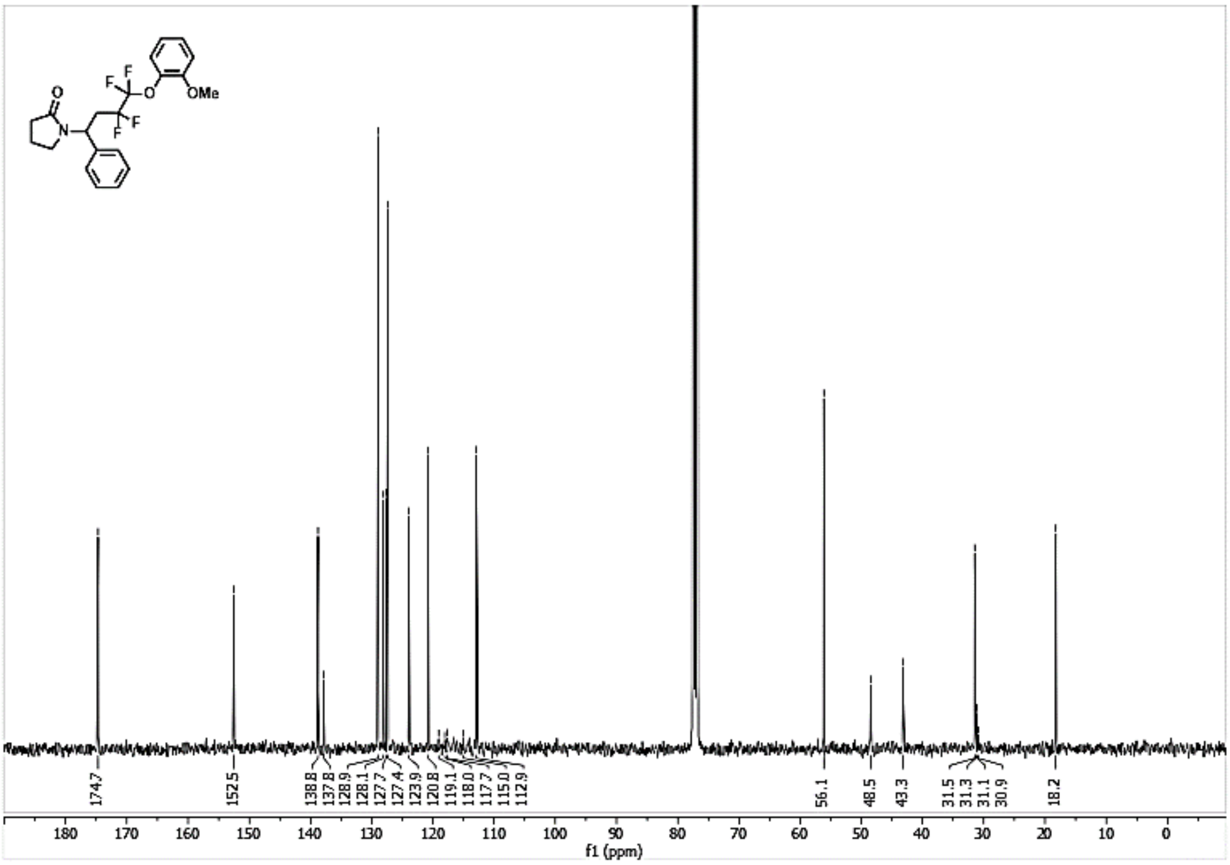

Compound 6b. ^{19}F NMR ($CDCl_3$, 376 MHz).

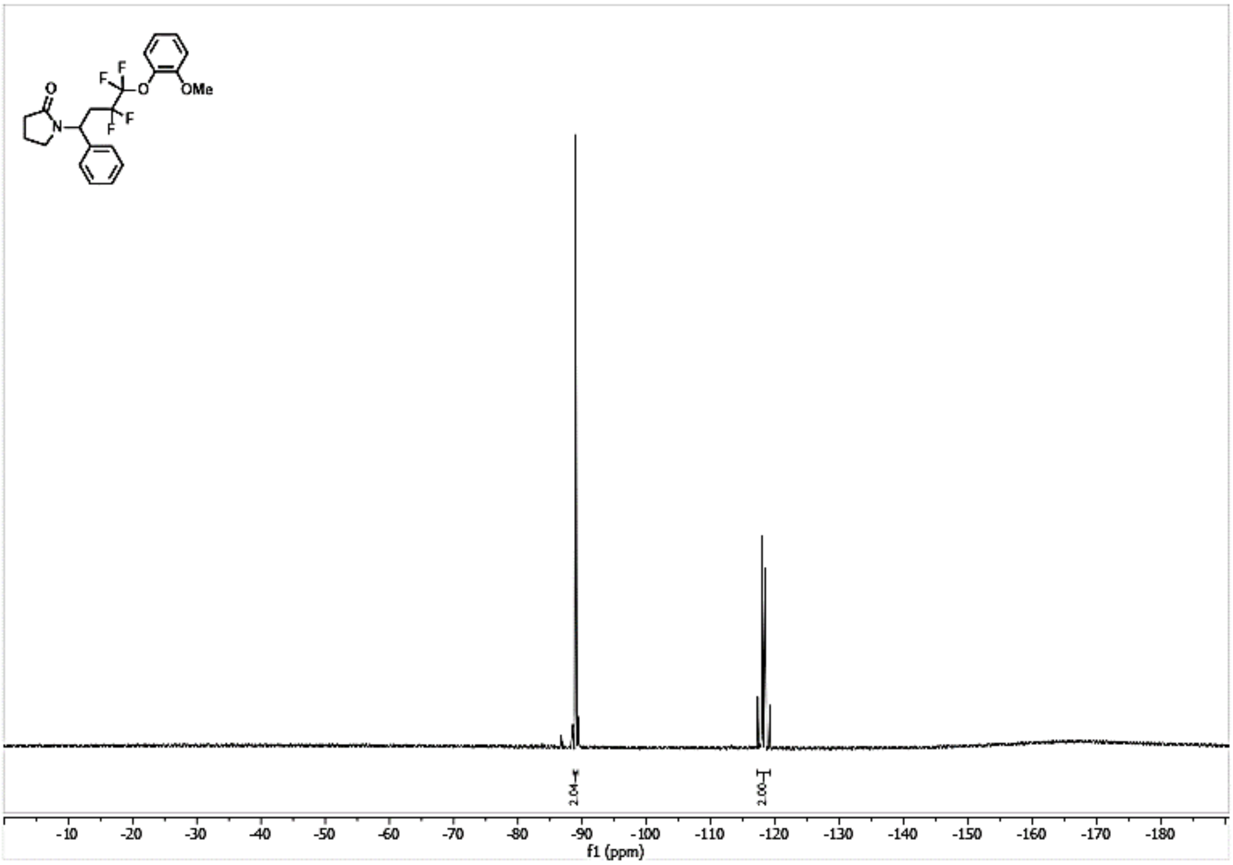

Compound 6c. Top: ^{1}H NMR ($CDCl_3$, 400 MHz). Bottom: ^{13}C NMR ($CDCl_3$, 100 MHz).

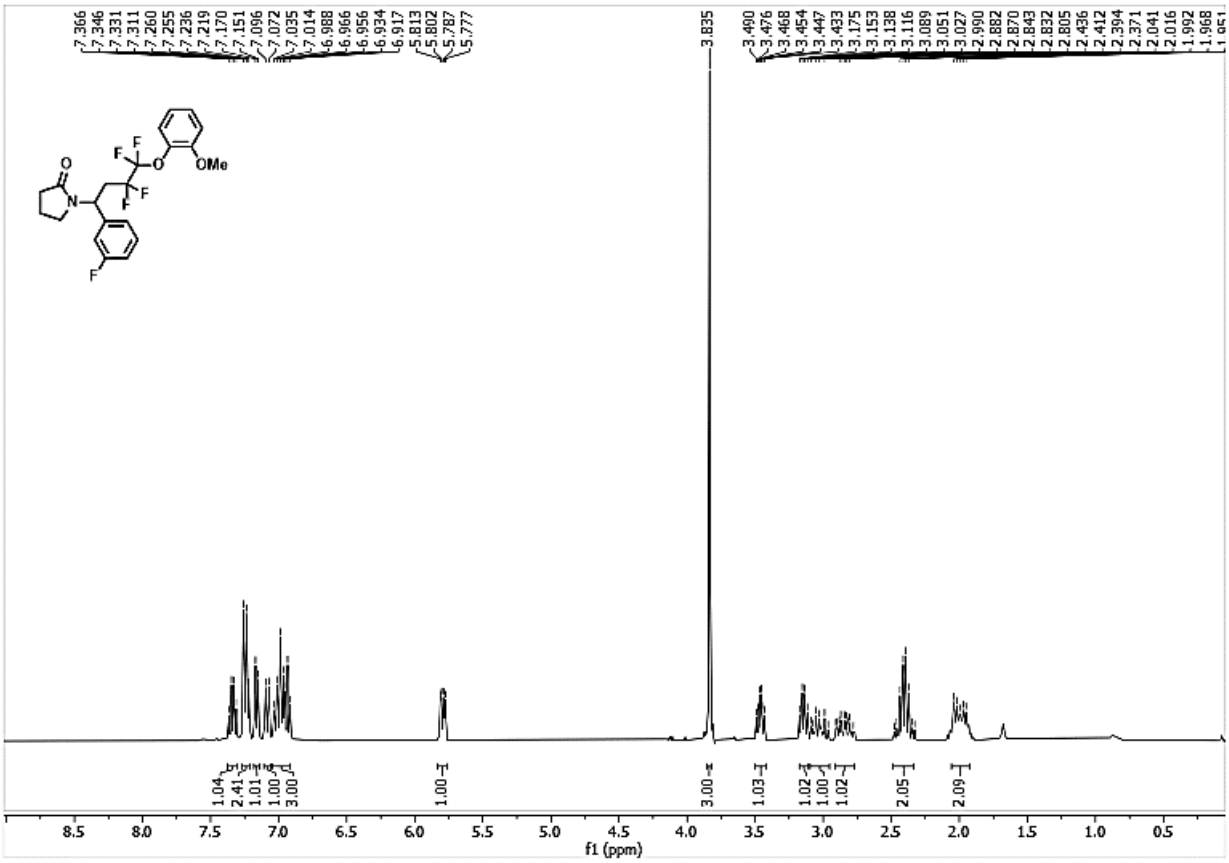

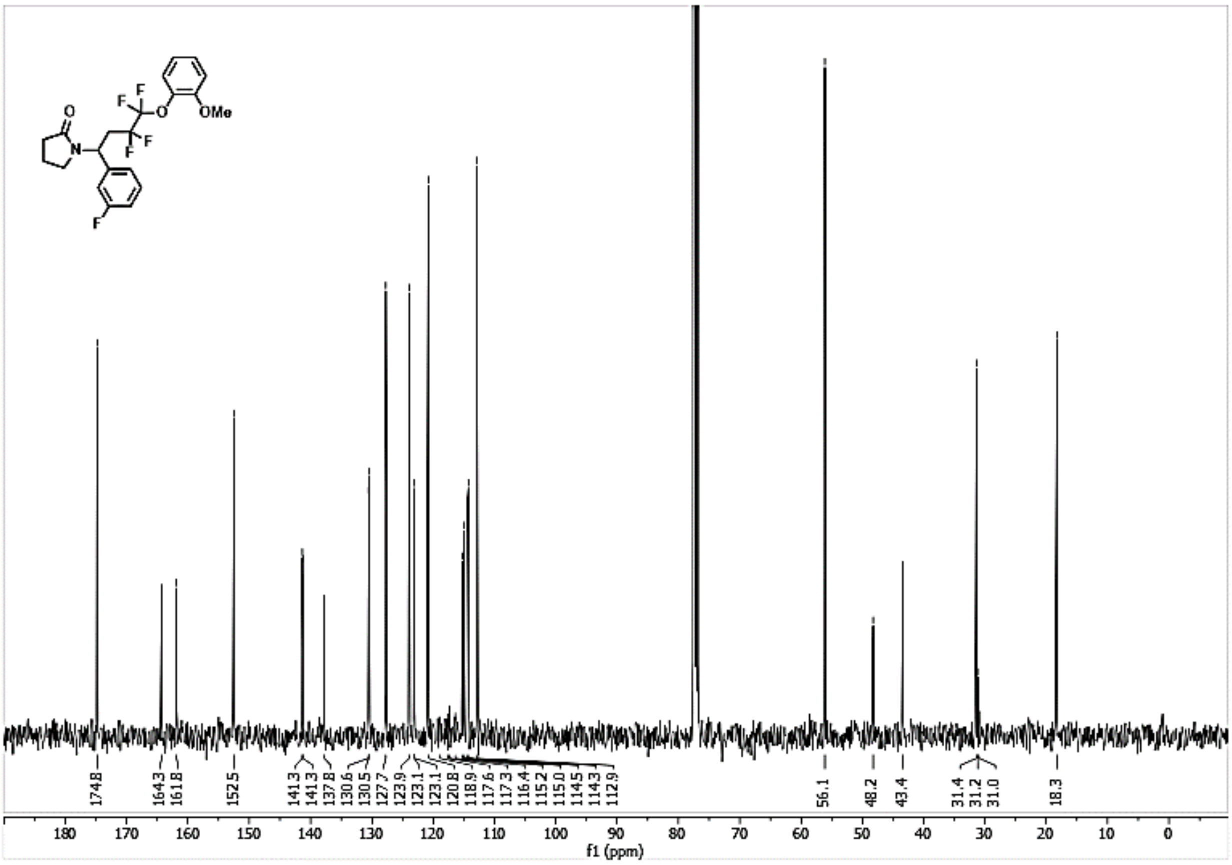

Compound 6c. ^{19}F NMR ($CDCl_3$, 376 MHz).

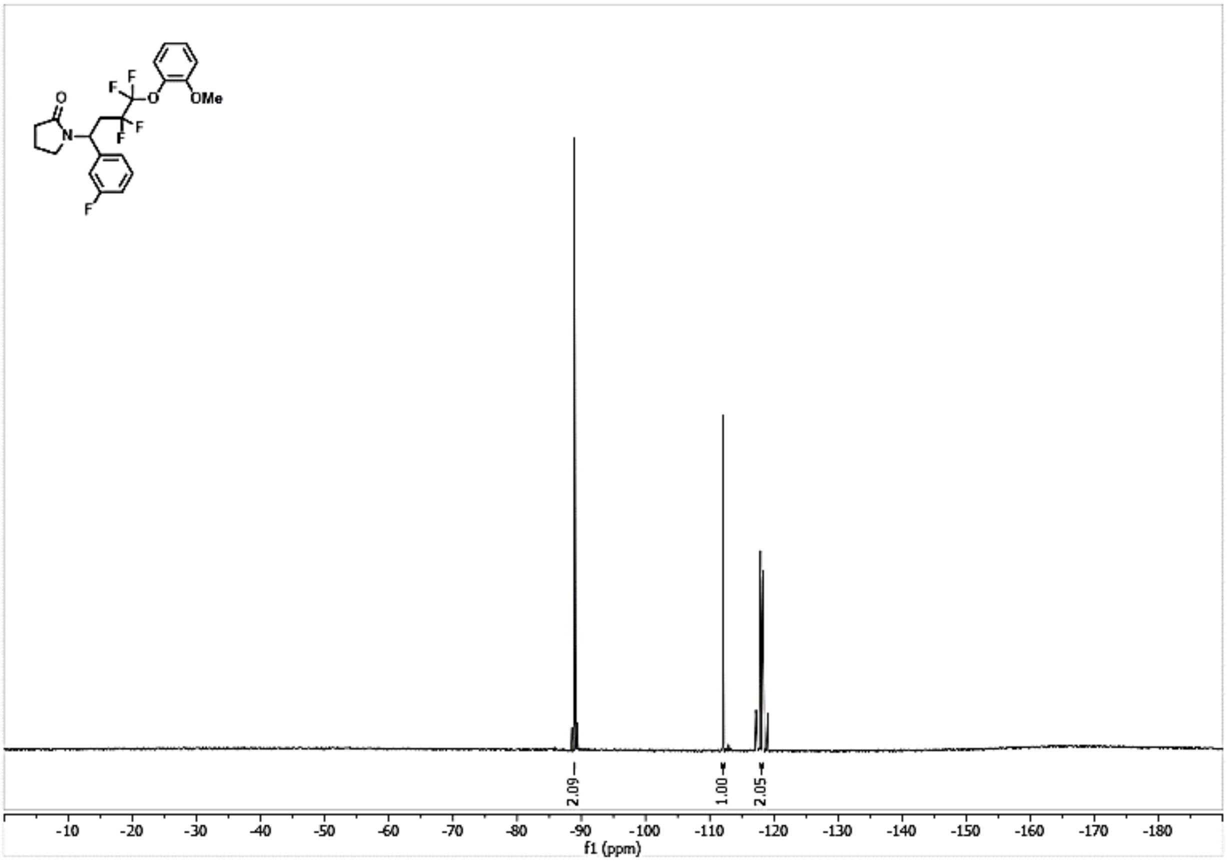

Compound 6d. Top: ^{1}H NMR ($CDCl_3$, 400 MHz). Bottom: ^{13}C NMR ($CDCl_3$, 100 MHz).

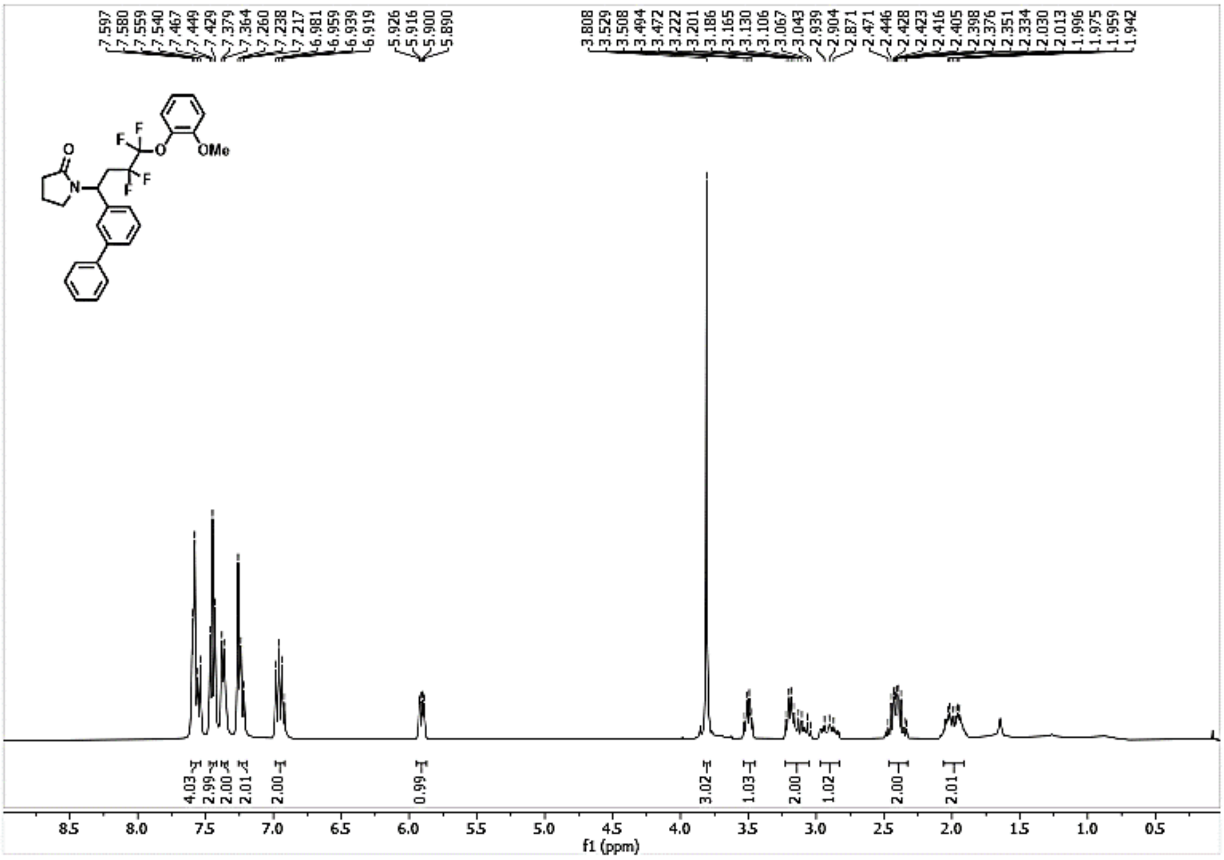

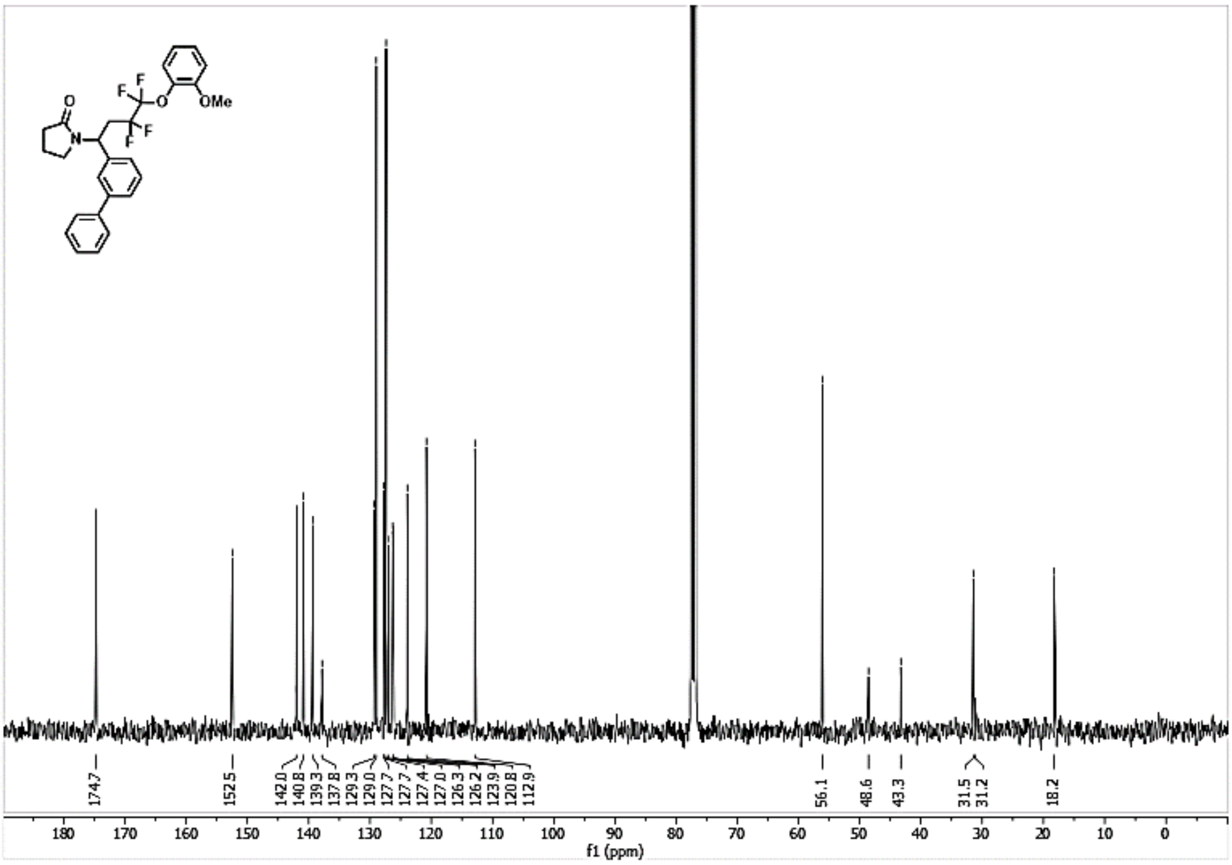

Compound 6d. ^{19}F NMR ($CDCl_3$, 376 MHz).

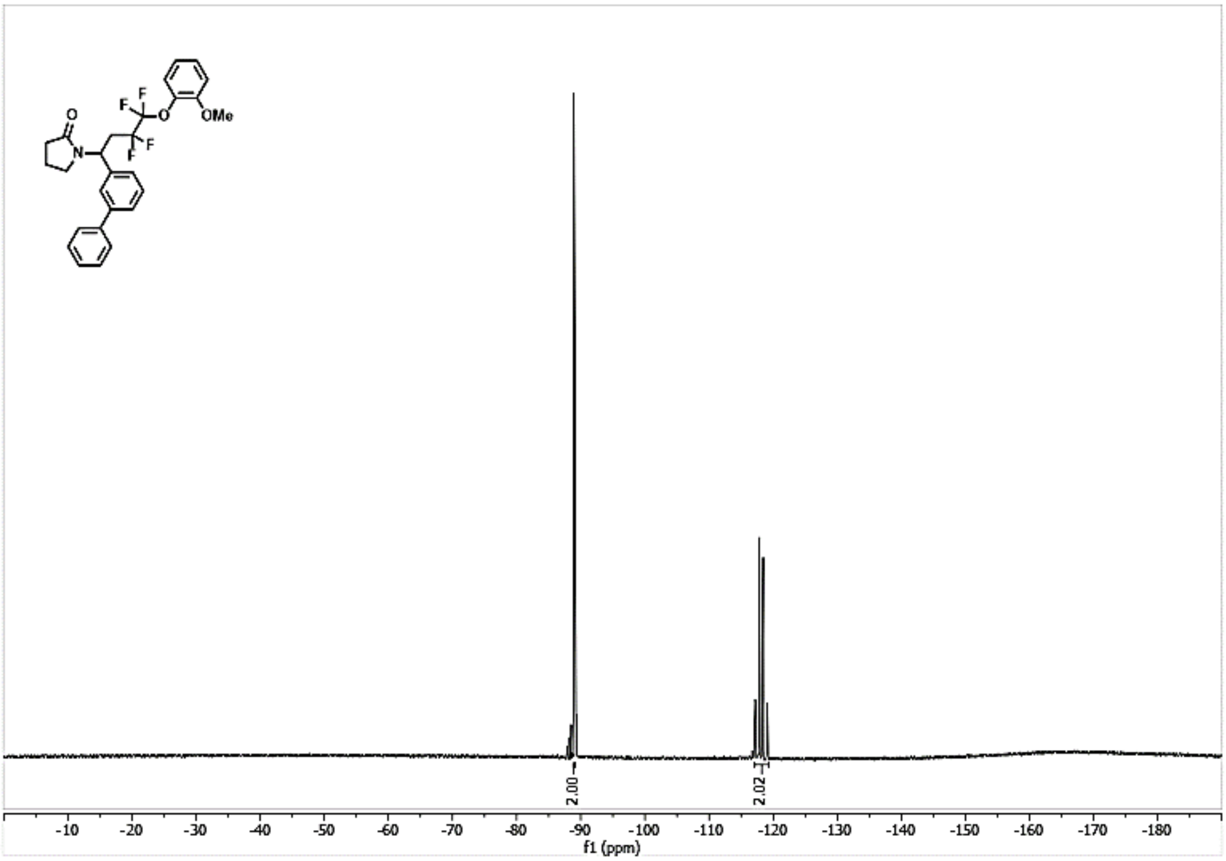

Compound 6e. Top: ^{1}H NMR ($CDCl_3$, 400 MHz). Bottom: ^{13}C NMR ($CDCl_3$, 100 MHz).

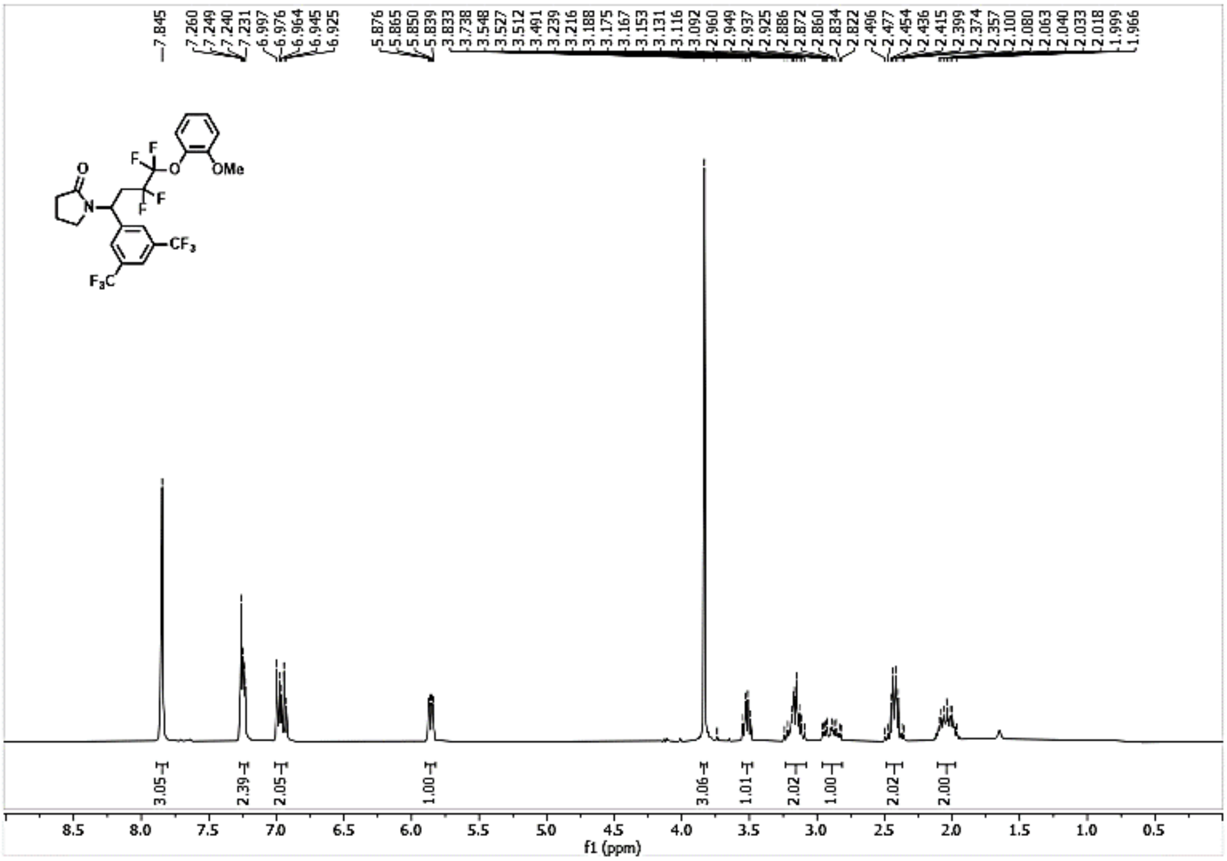

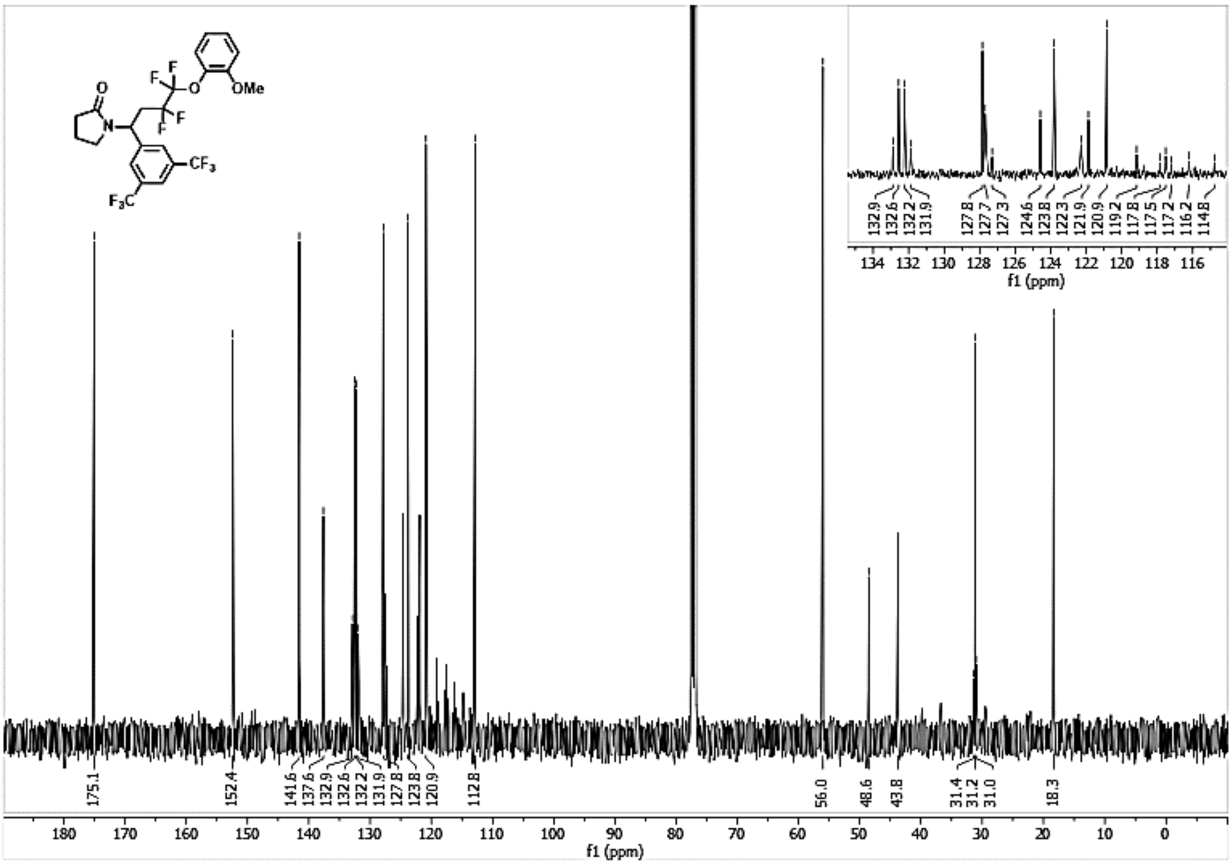

Compound 6e. ^{19}F NMR ($CDCl_3$, 376 MHz).

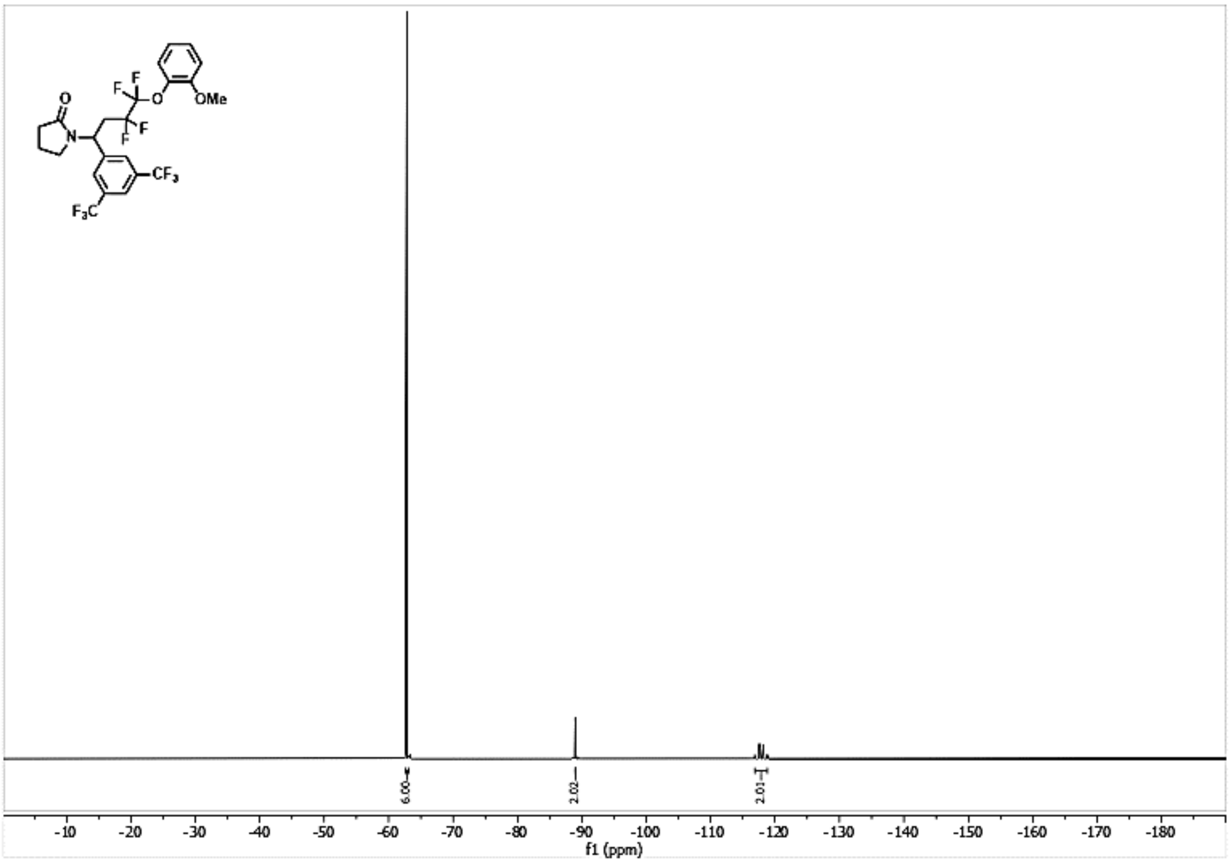

Compound 5f$_{major}$. Top: ^1H NMR ($CDCl_3$, 400 MHz). Bottom: ^{13}C NMR ($CDCl_3$, 100 MHz).

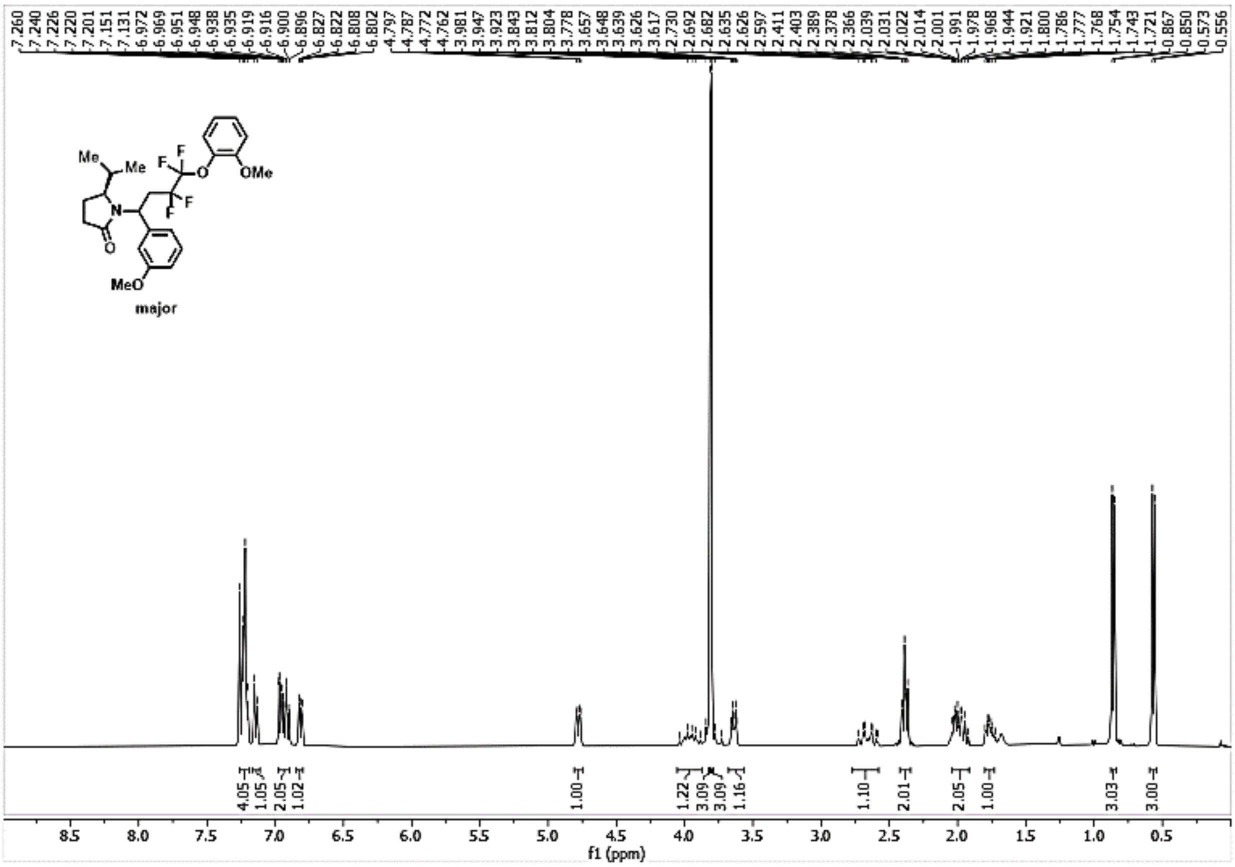

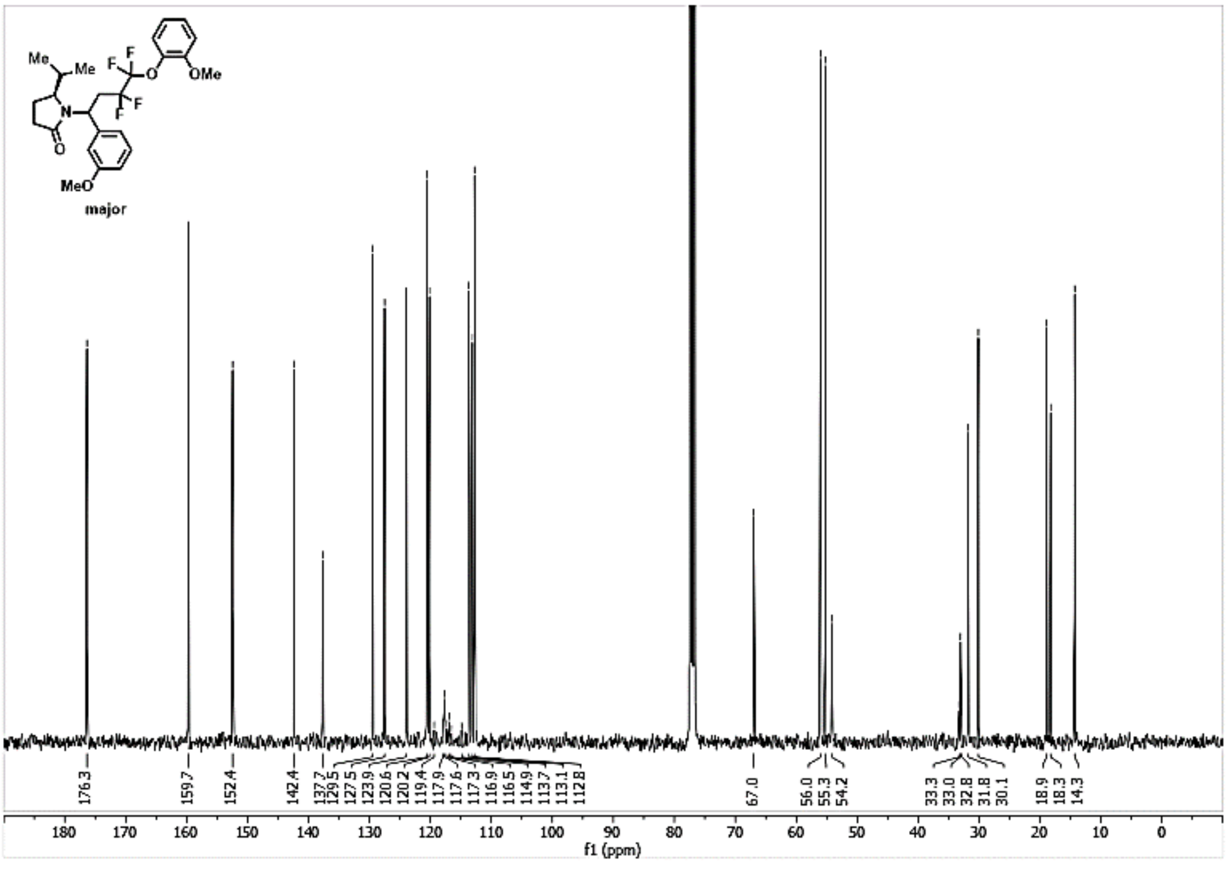

Compound 6f$_{major}$. ^{19}F NMR ($CDCl_3$, 376 MHz).

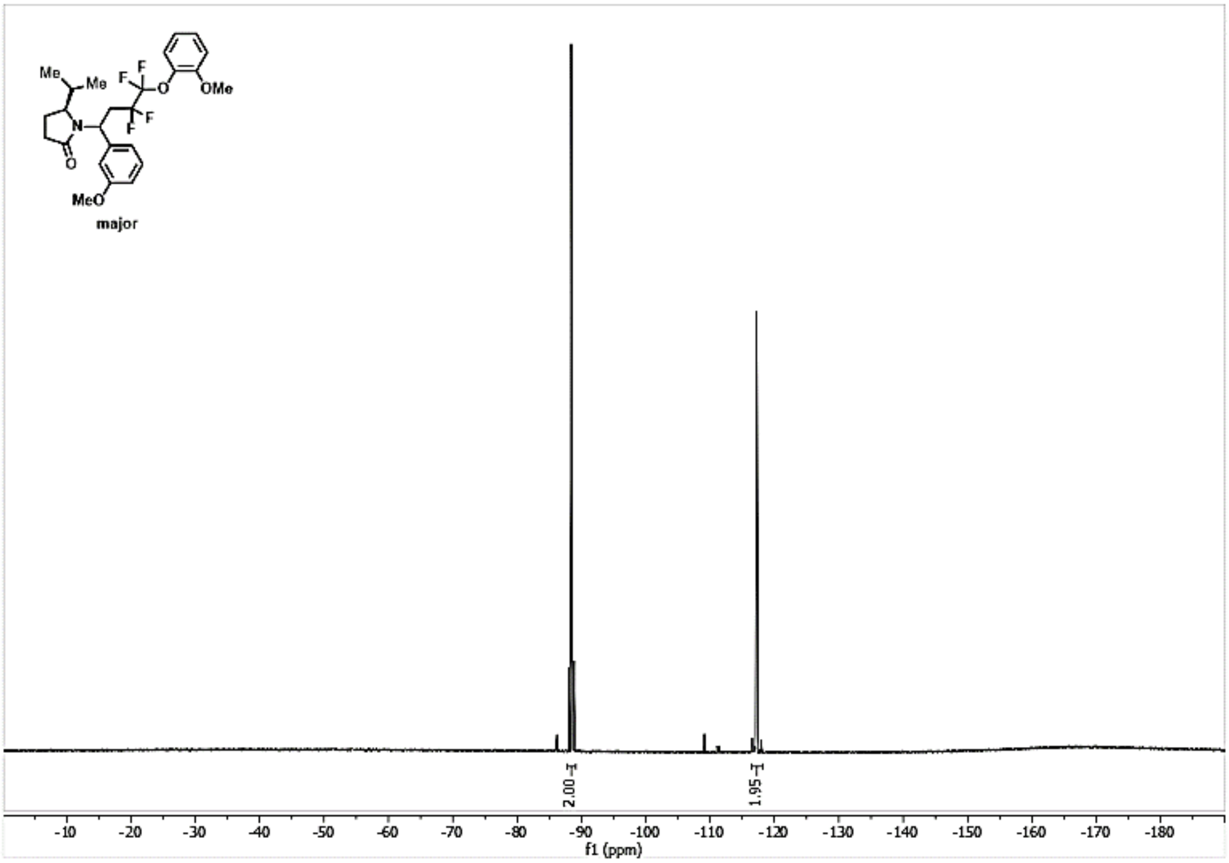

Compound 6f$_{minor}$. Top: ^{1}H NMR ($CDCl_3$, 400 MHz). Bottom: ^{13}C NMR ($CDCl_3$, 100 MHz).

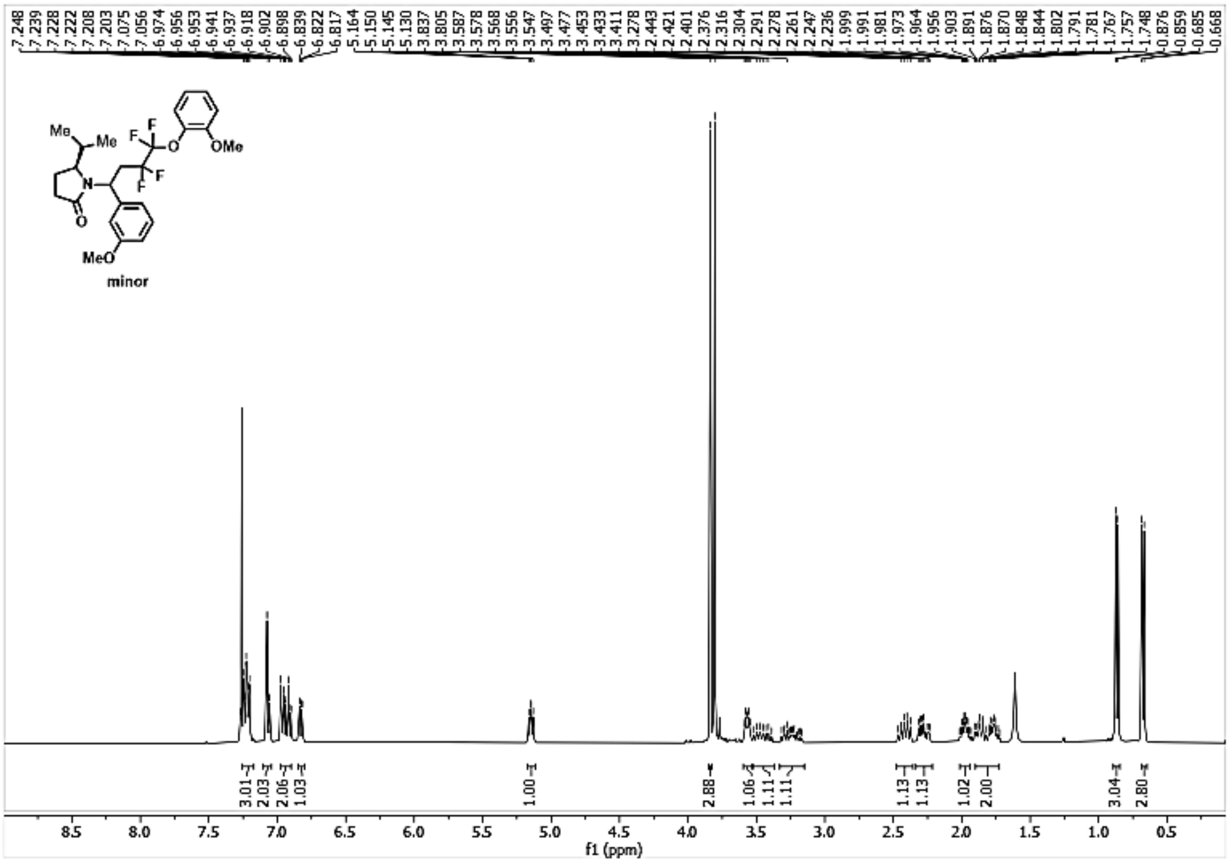

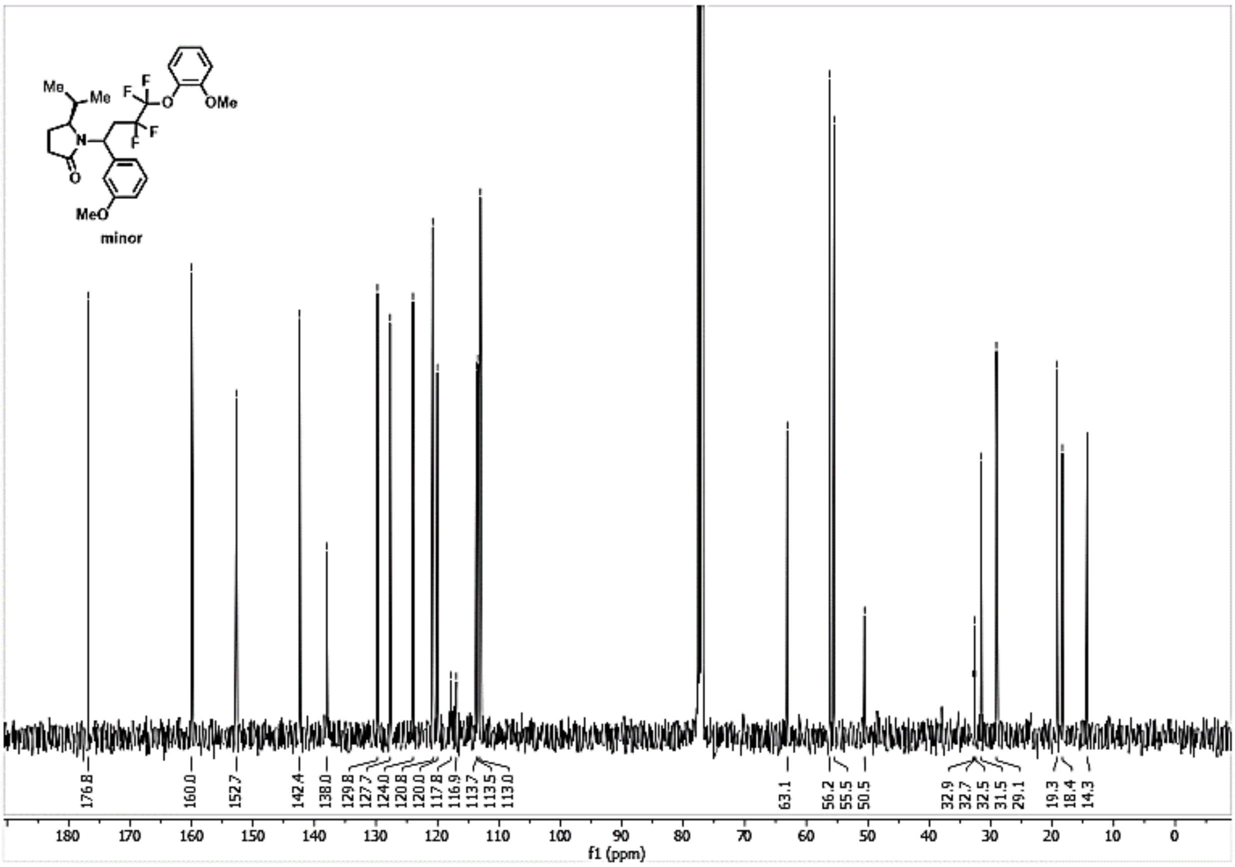

Compound 6f$_{minor}$. ^{19}F NMR ($CDCl_3$, 376 MHz).

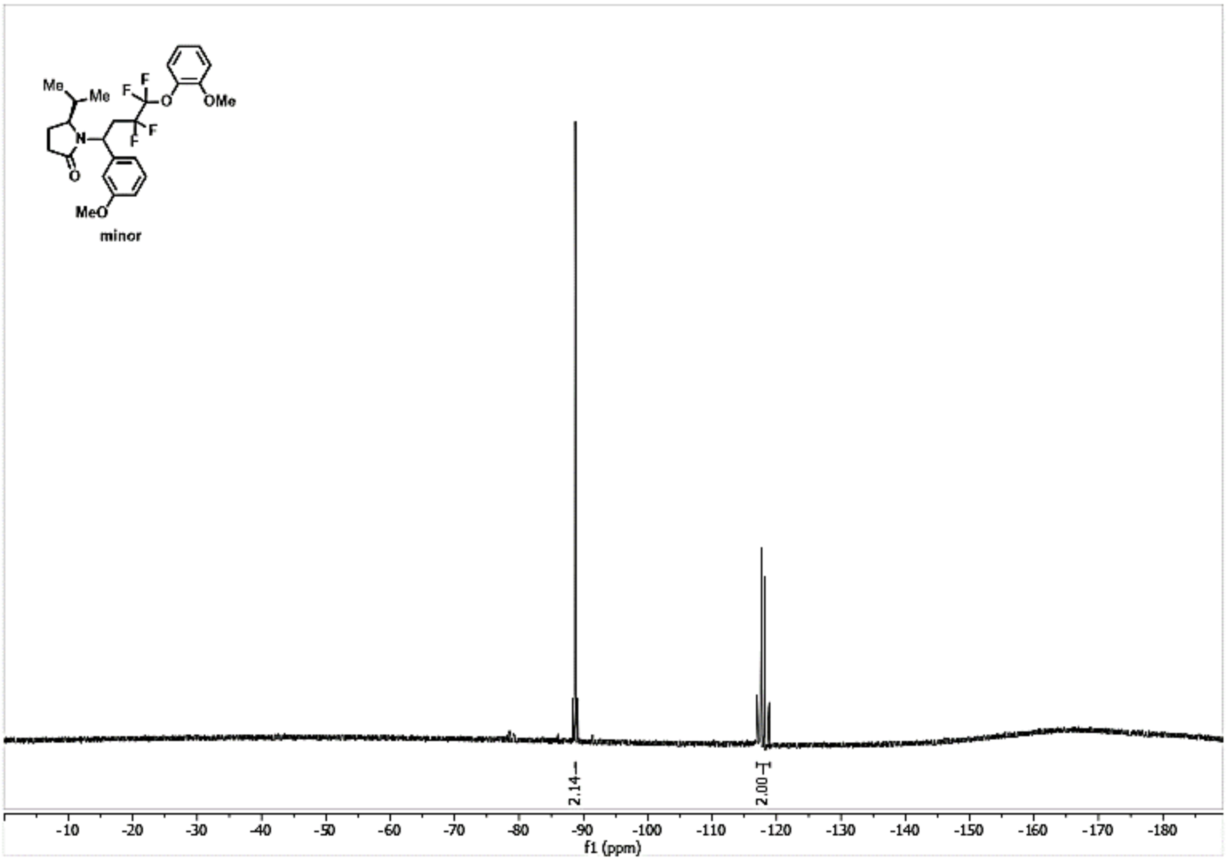

Compound 6g. Top: ^{1}H NMR ($CDCl_3$, 400 MHz). Bottom: ^{13}C NMR ($CDCl_3$, 100 MHz).

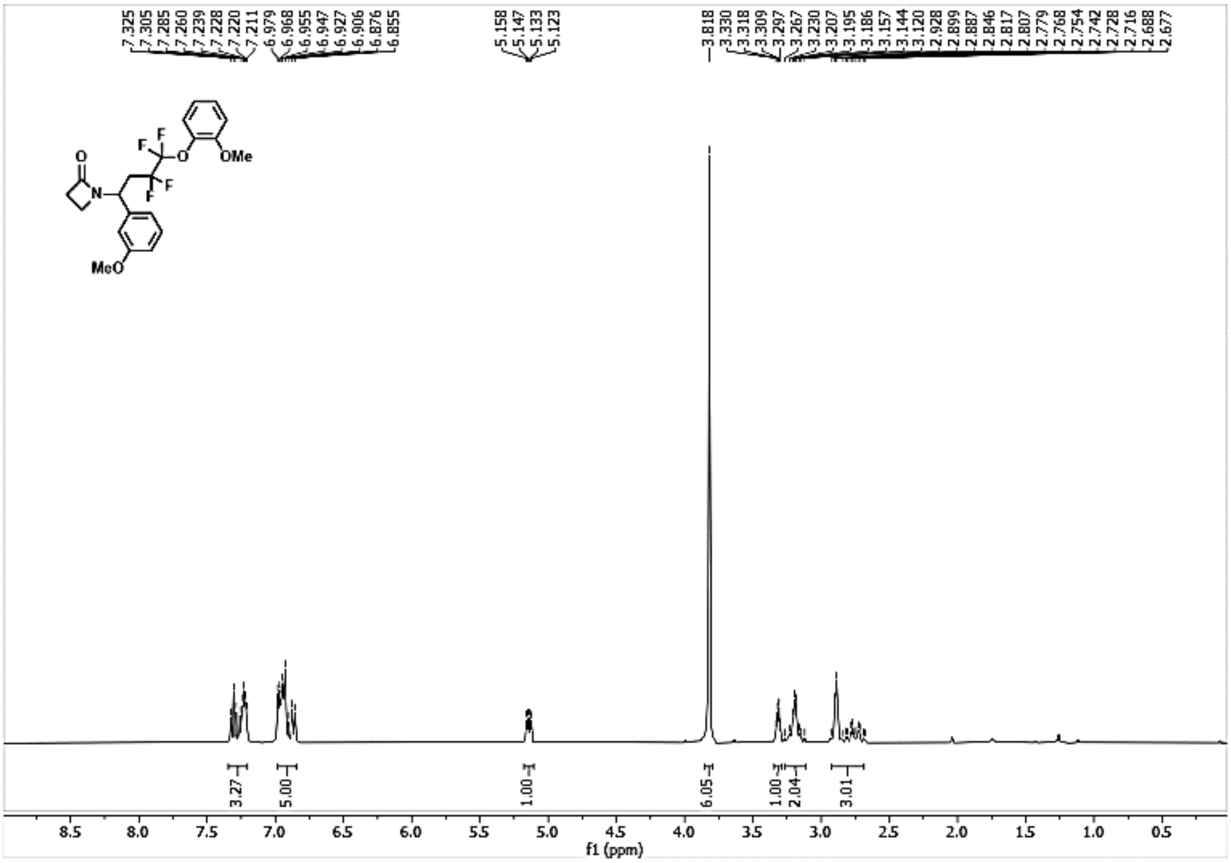

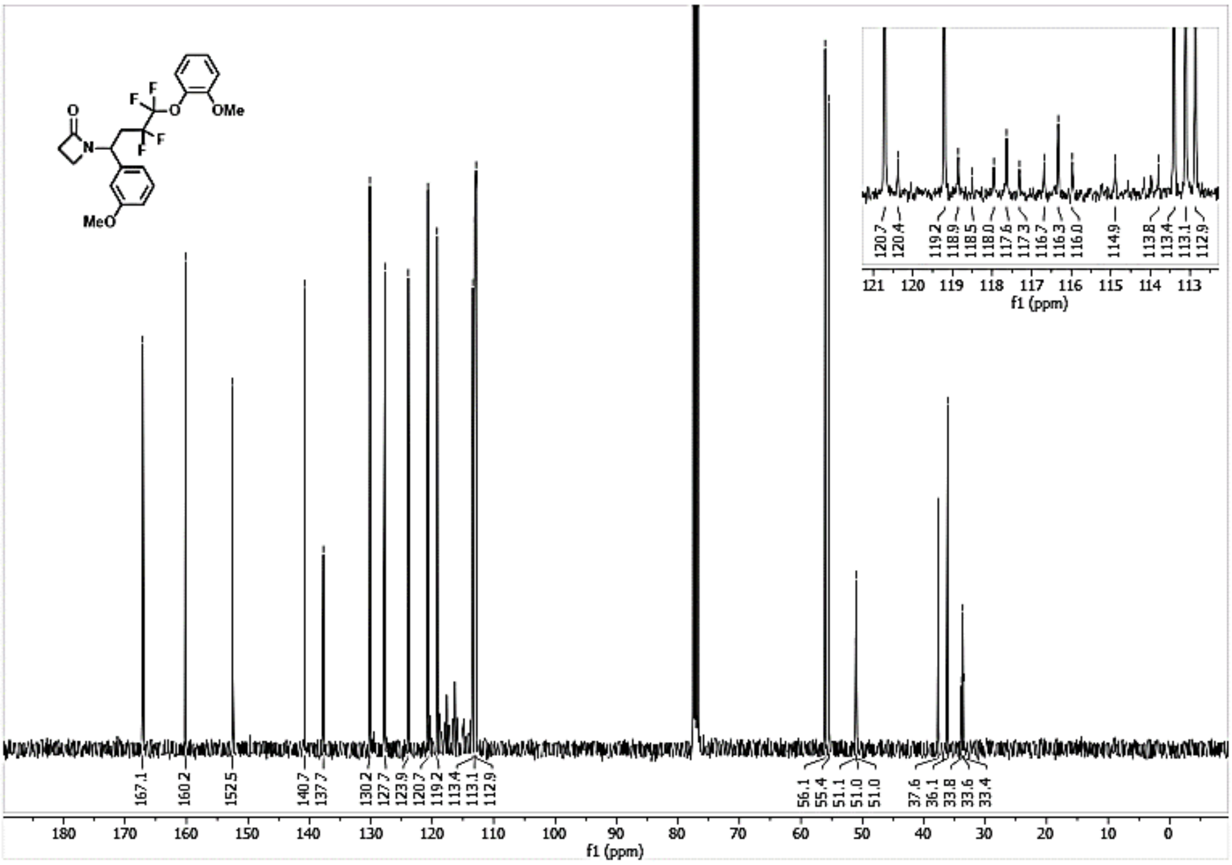

Compound 6g. ^{19}F NMR ($CDCl_3$, 376 MHz).

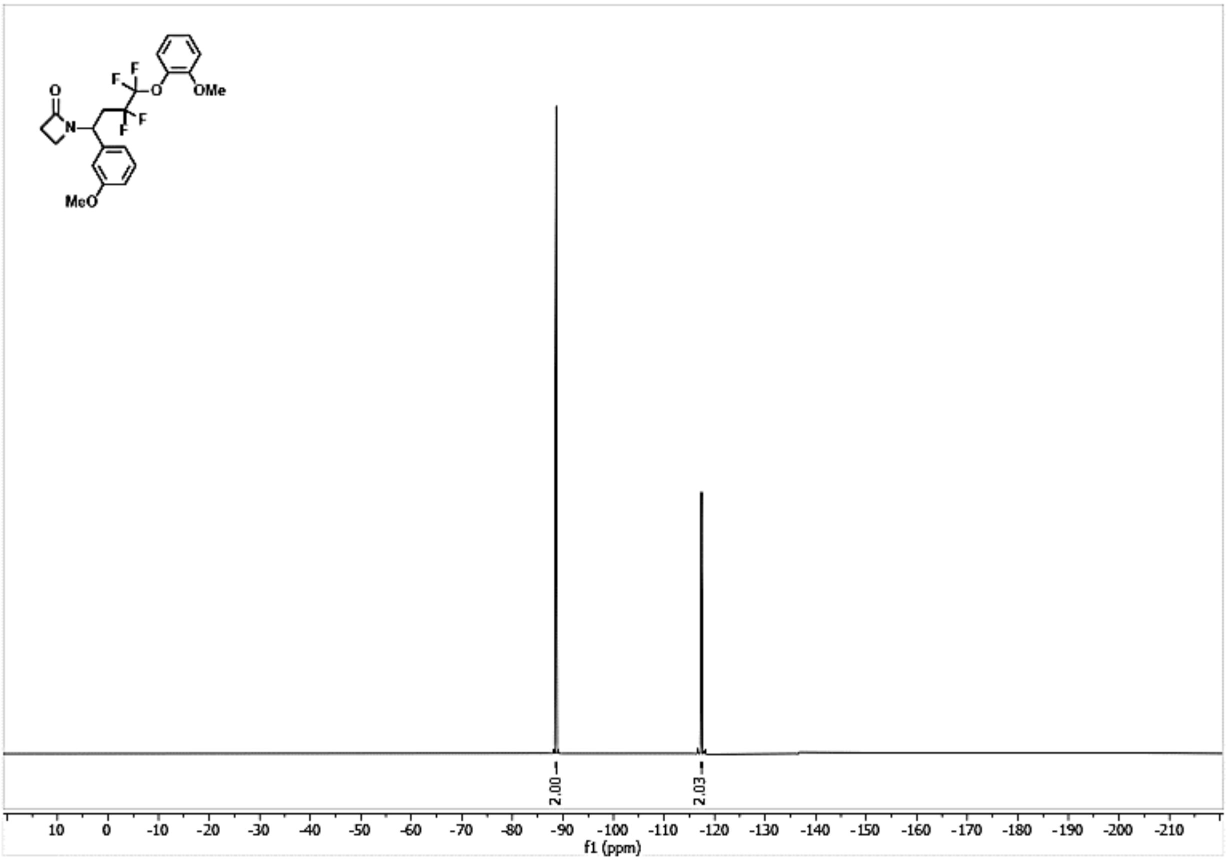

Compound 6h. Top: ^{1}H NMR ($CDCl_3$, 400 MHz). Bottom: ^{13}C NMR ($CDCl_3$, 100 MHz).

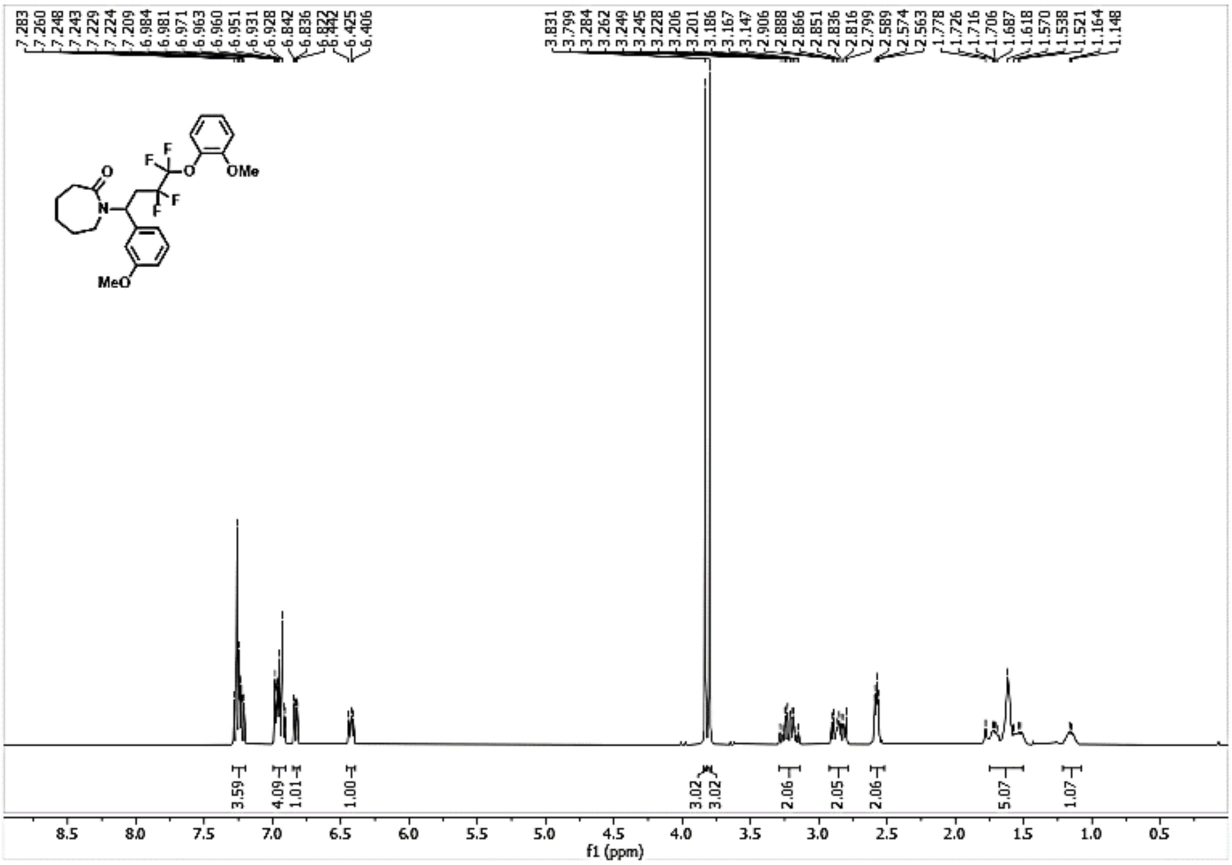

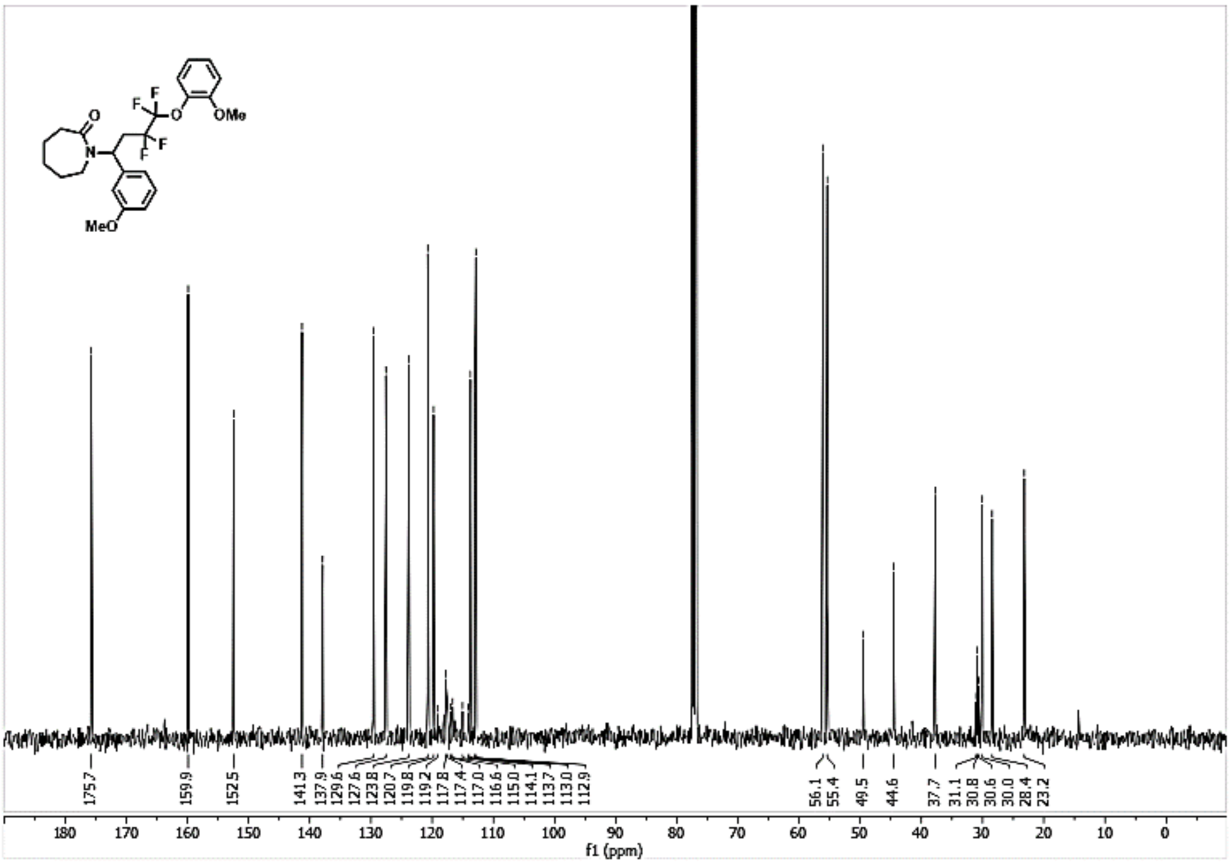

Compound 6h. ^{19}F NMR ($CDCl_3$, 376 MHz).

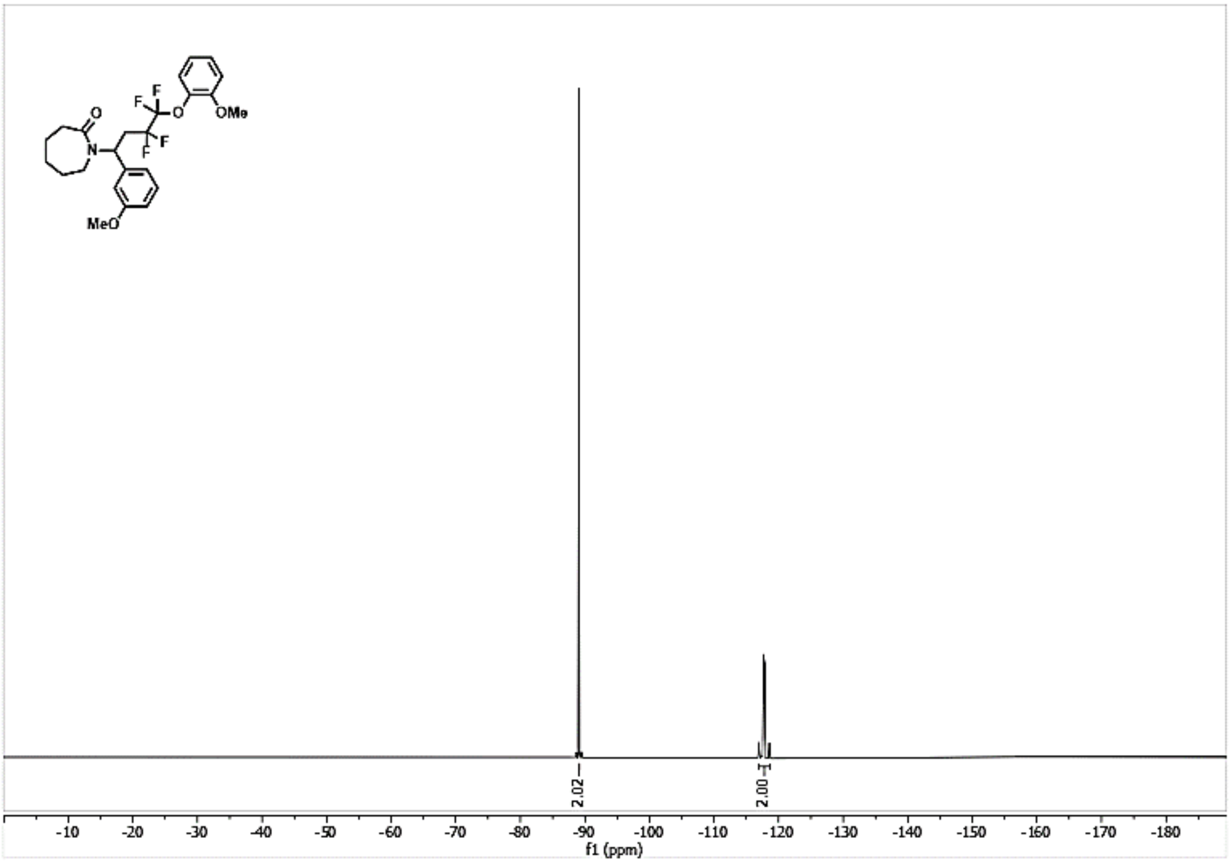

Compound 6i. Top: ^{1}H NMR ($CDCl_3$, 400 MHz). Bottom: ^{13}C NMR ($CDCl_3$, 100 MHz).

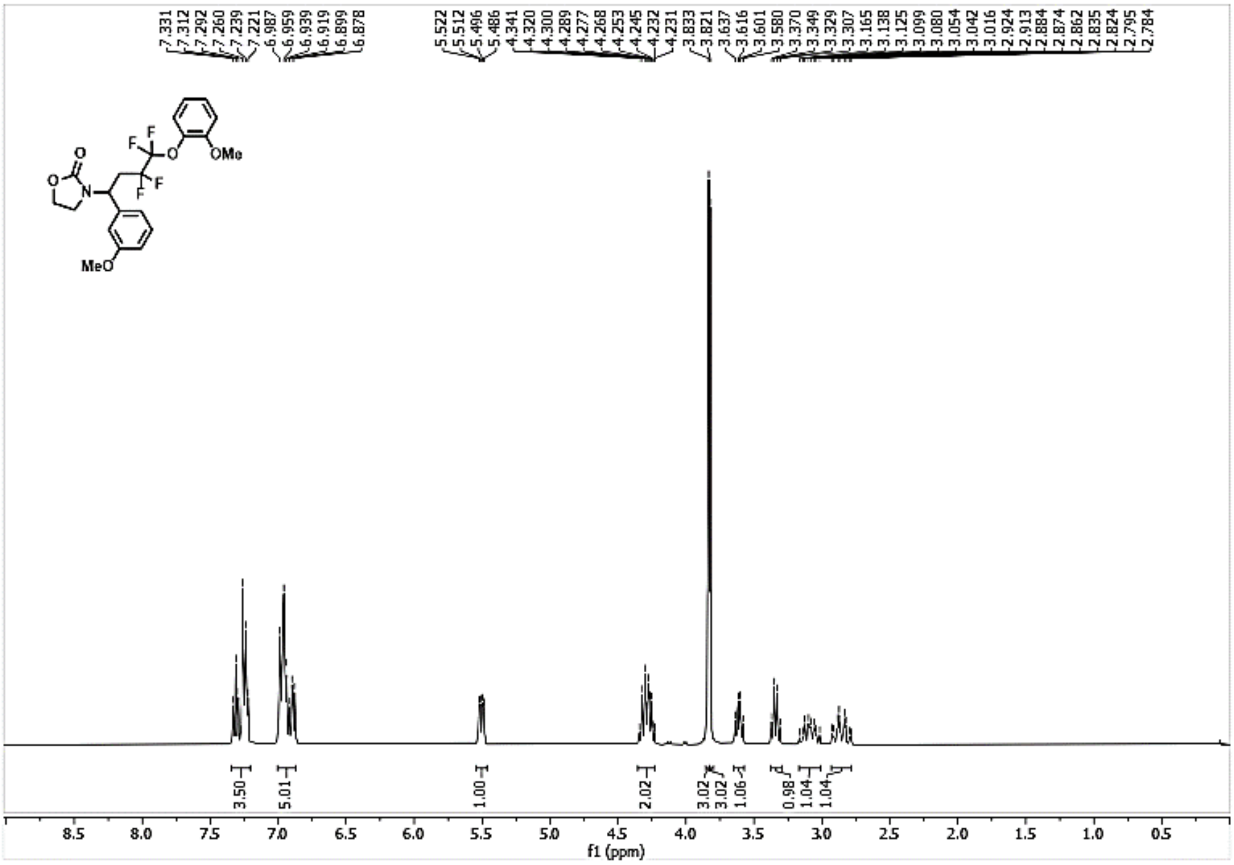

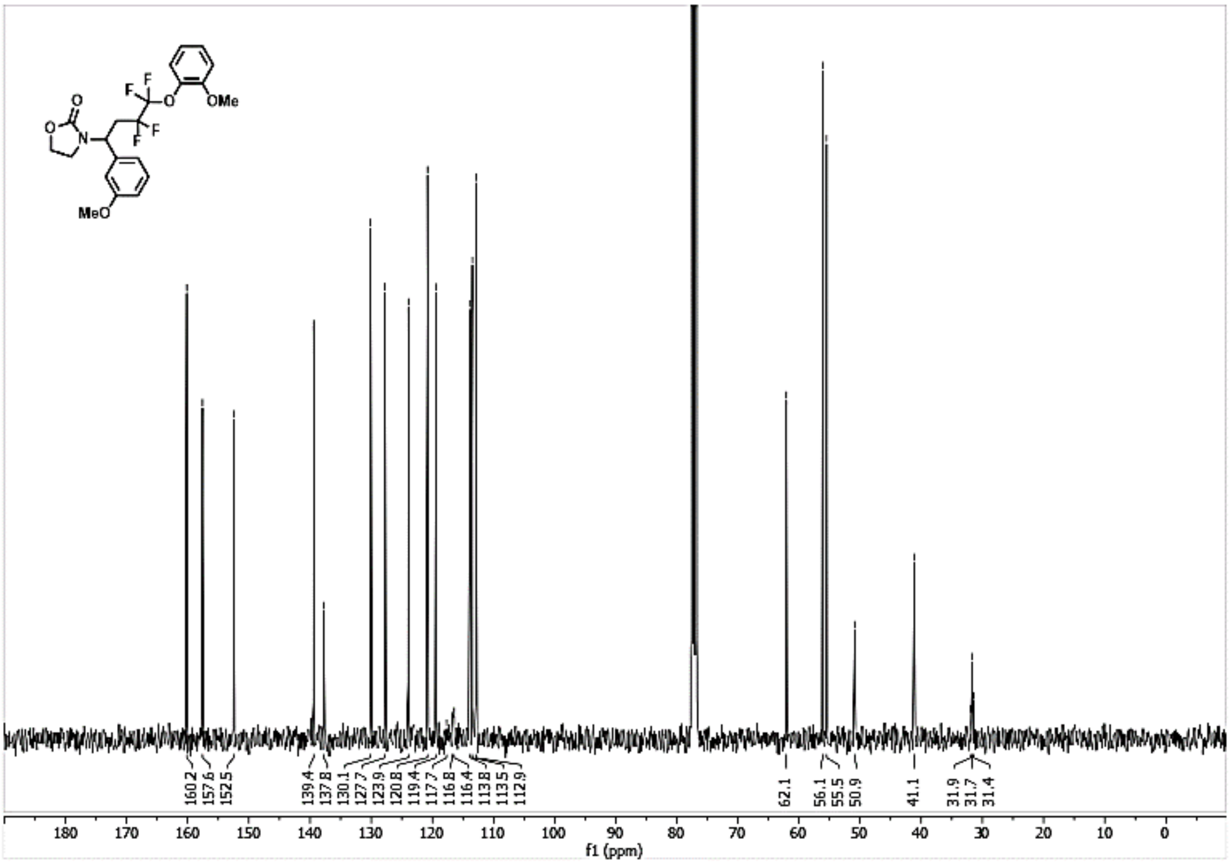

Compound 6i. ^{19}F NMR ($CDCl_3$, 376 MHz).

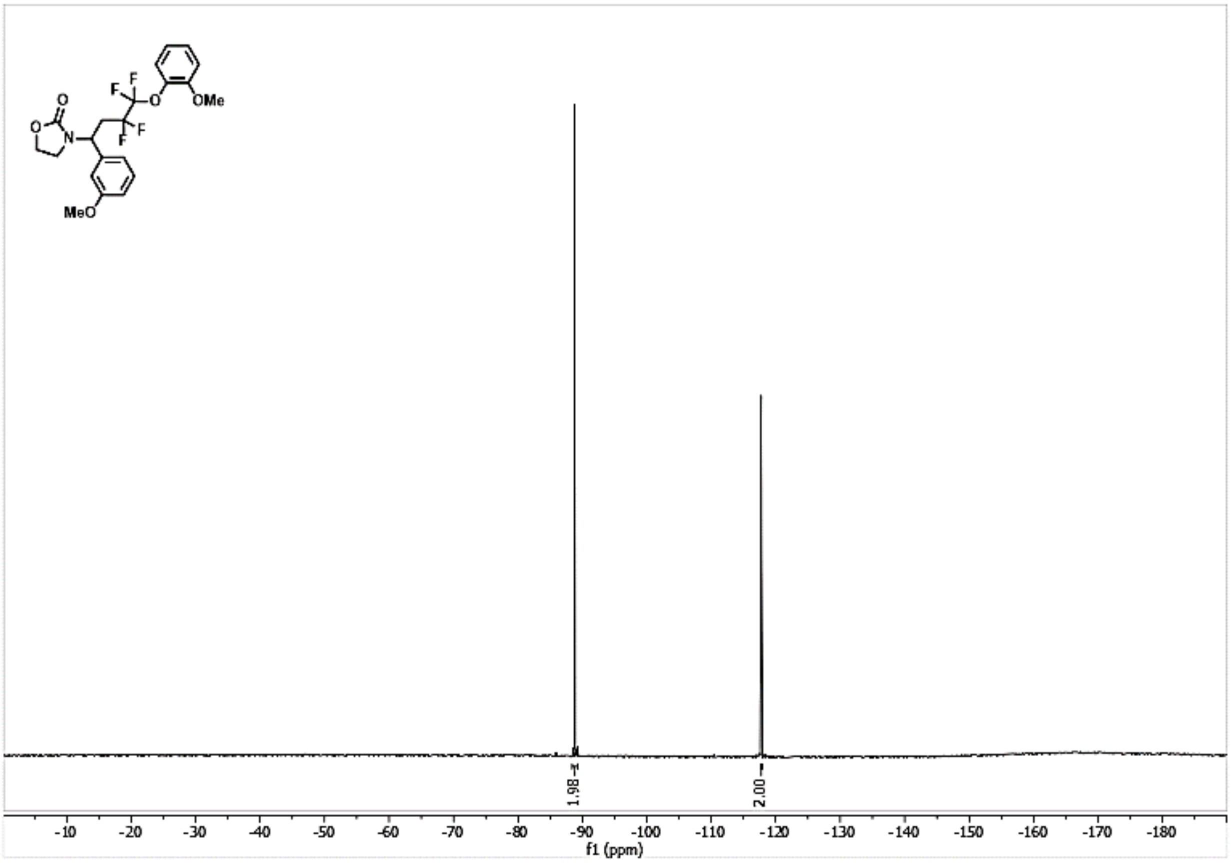

Compound 6j. Top: ^{1}H NMR ($CDCl_3$, 400 MHz). Bottom: ^{13}C NMR ($CDCl_3$, 100 MHz).

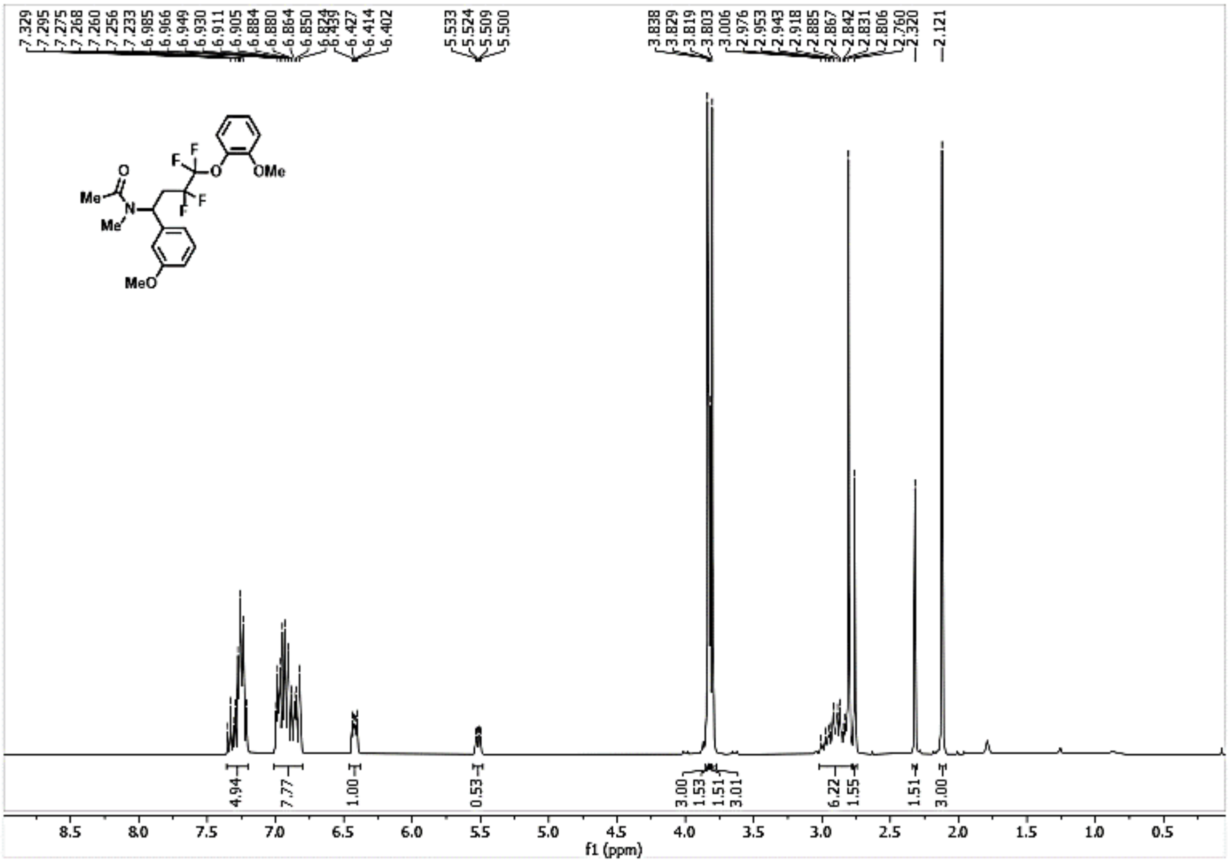

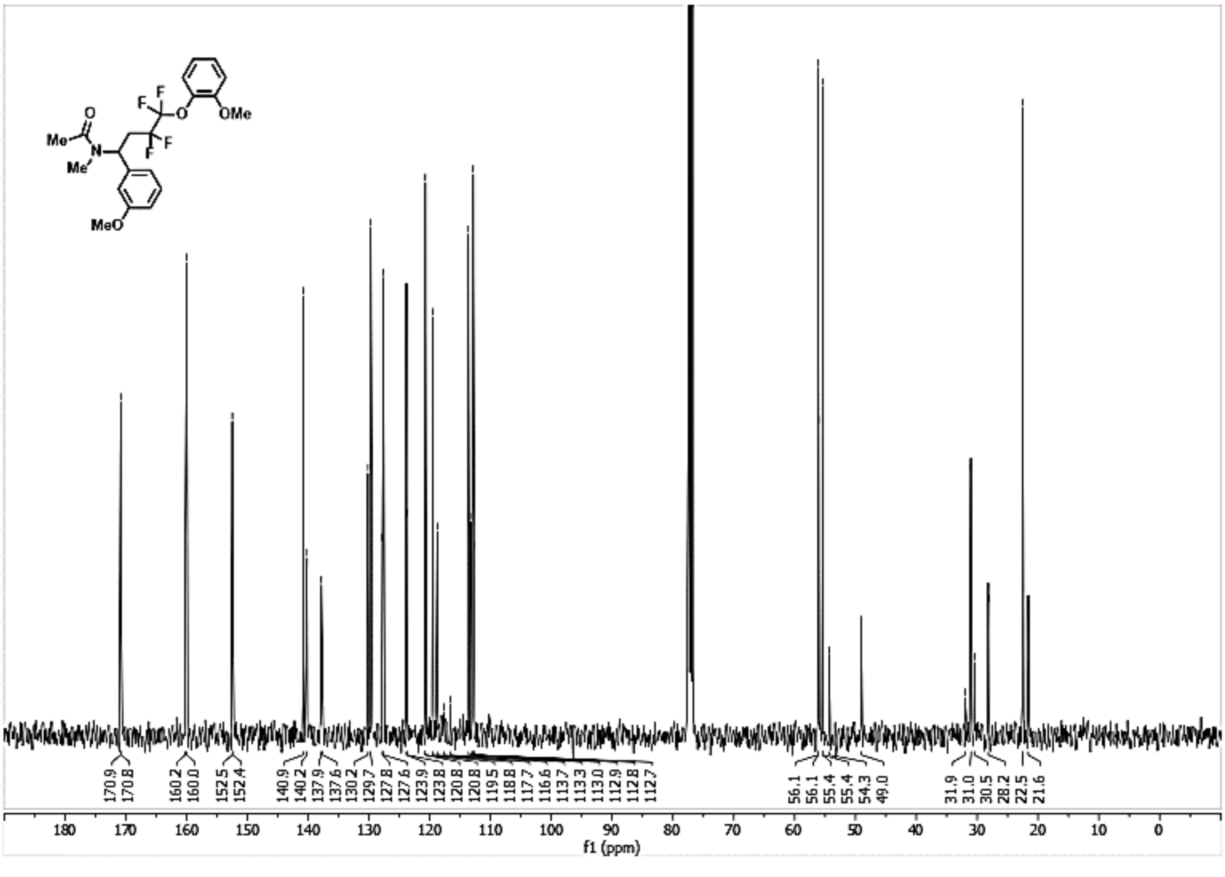

Compound 6j. ^{19}F NMR ($CDCl_3$, 376 MHz).

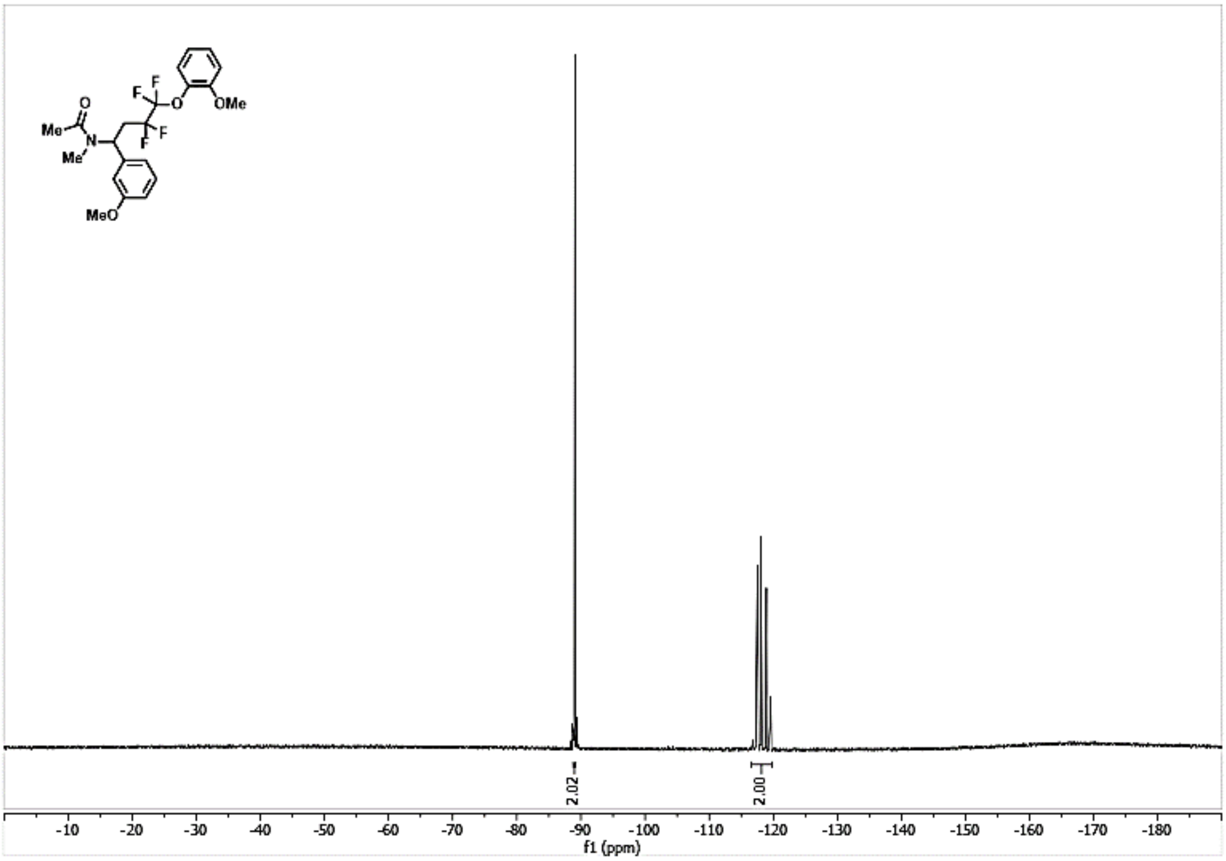

Compound 7. Top: ^{1}H NMR ($CDCl_3$, 400 MHz). Bottom: ^{13}C NMR ($CDCl_3$, 100 MHz).

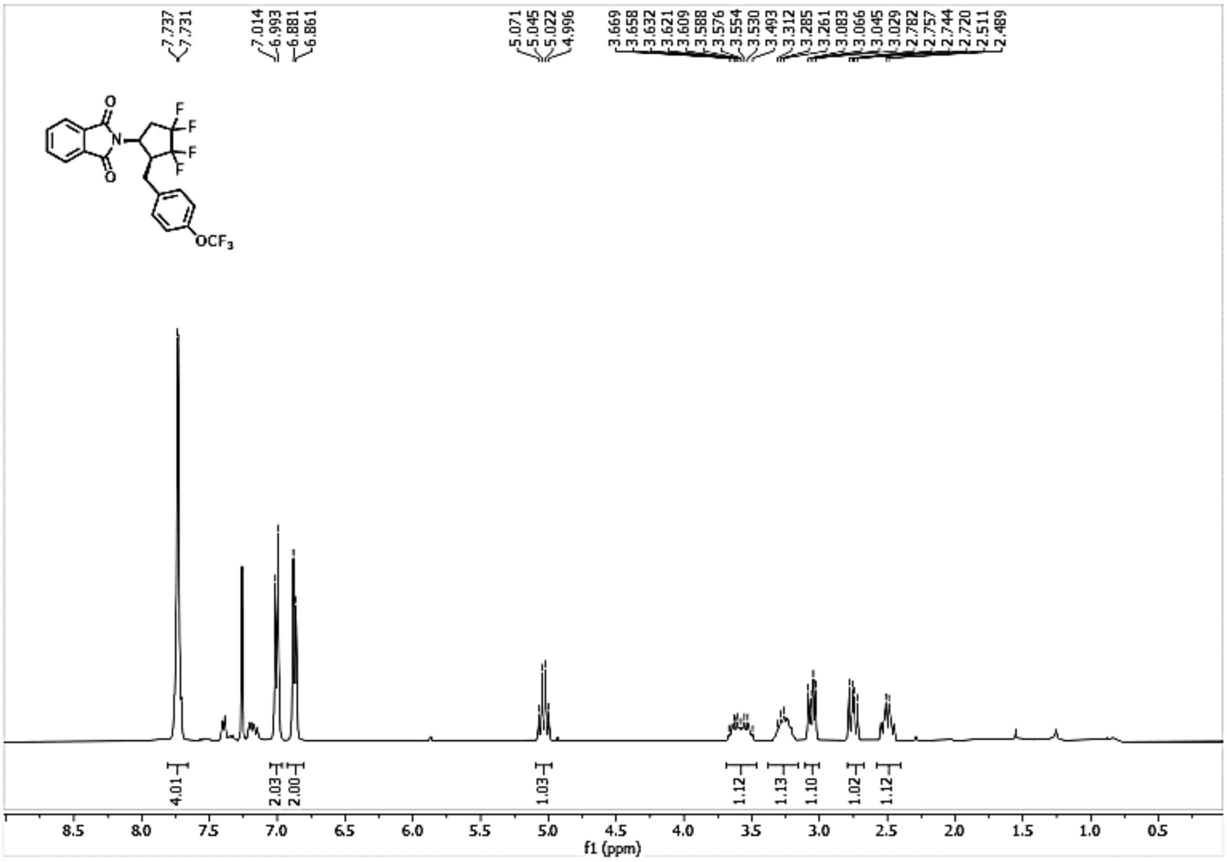

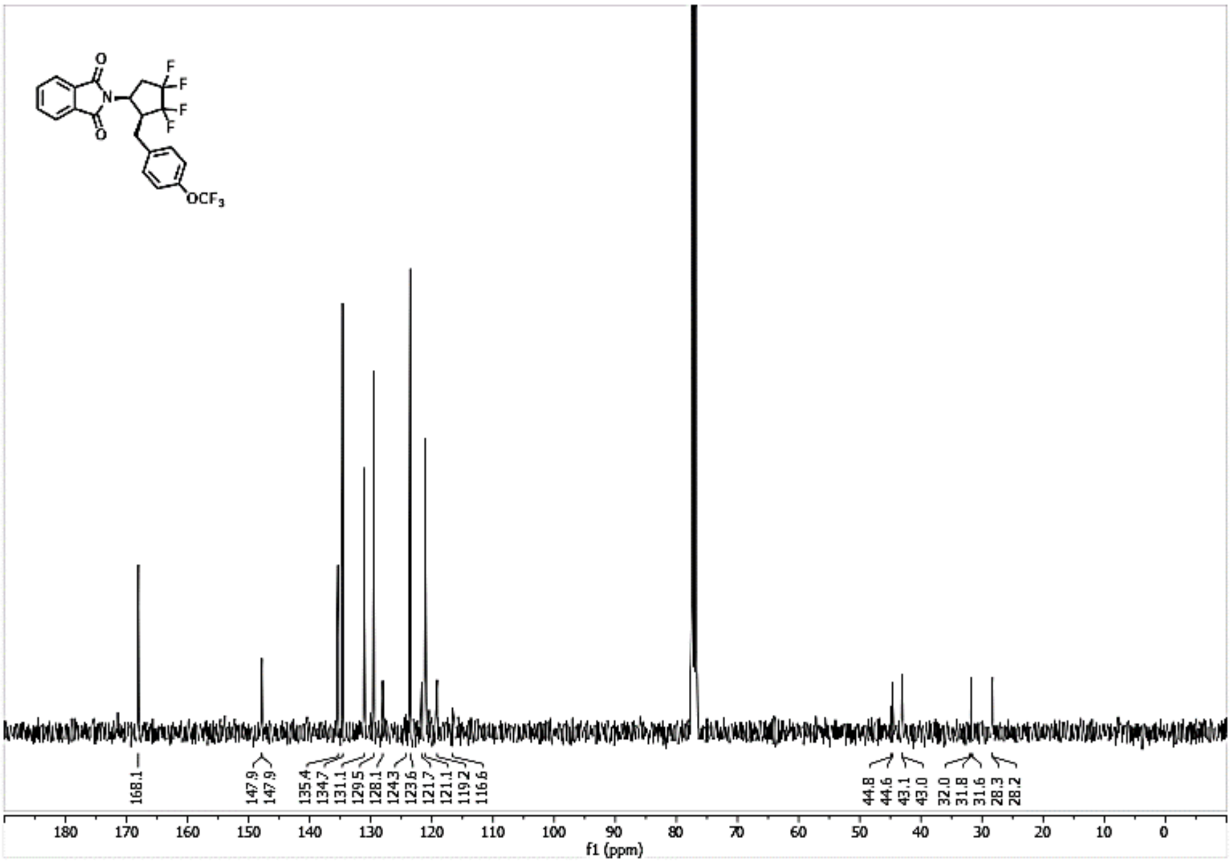

Compound 7. ^{19}F NMR ($CDCl_3$, 376 MHz).

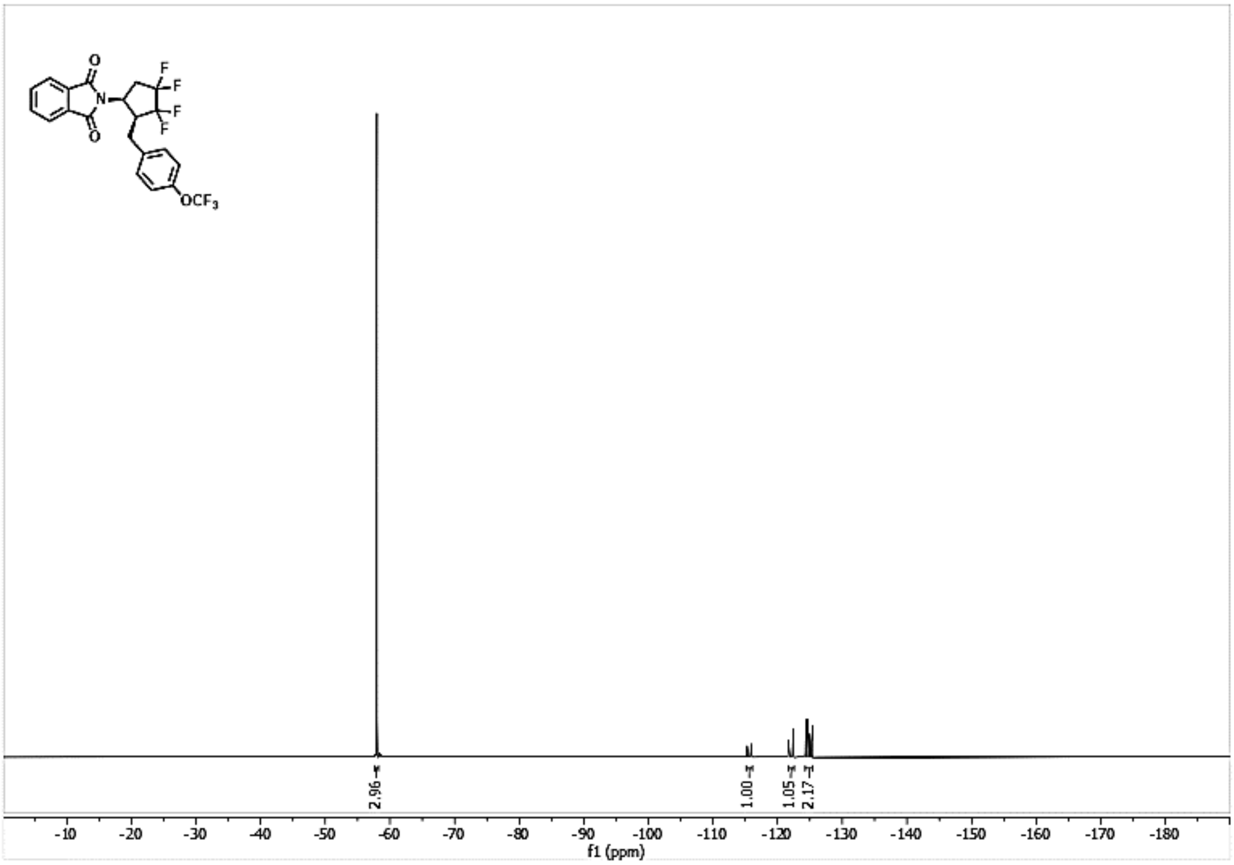

Compound 8. Top: ^{1}H NMR ($CDCl_3$, 400 MHz). Bottom: ^{13}C NMR ($CDCl_3$, 100 MHz).

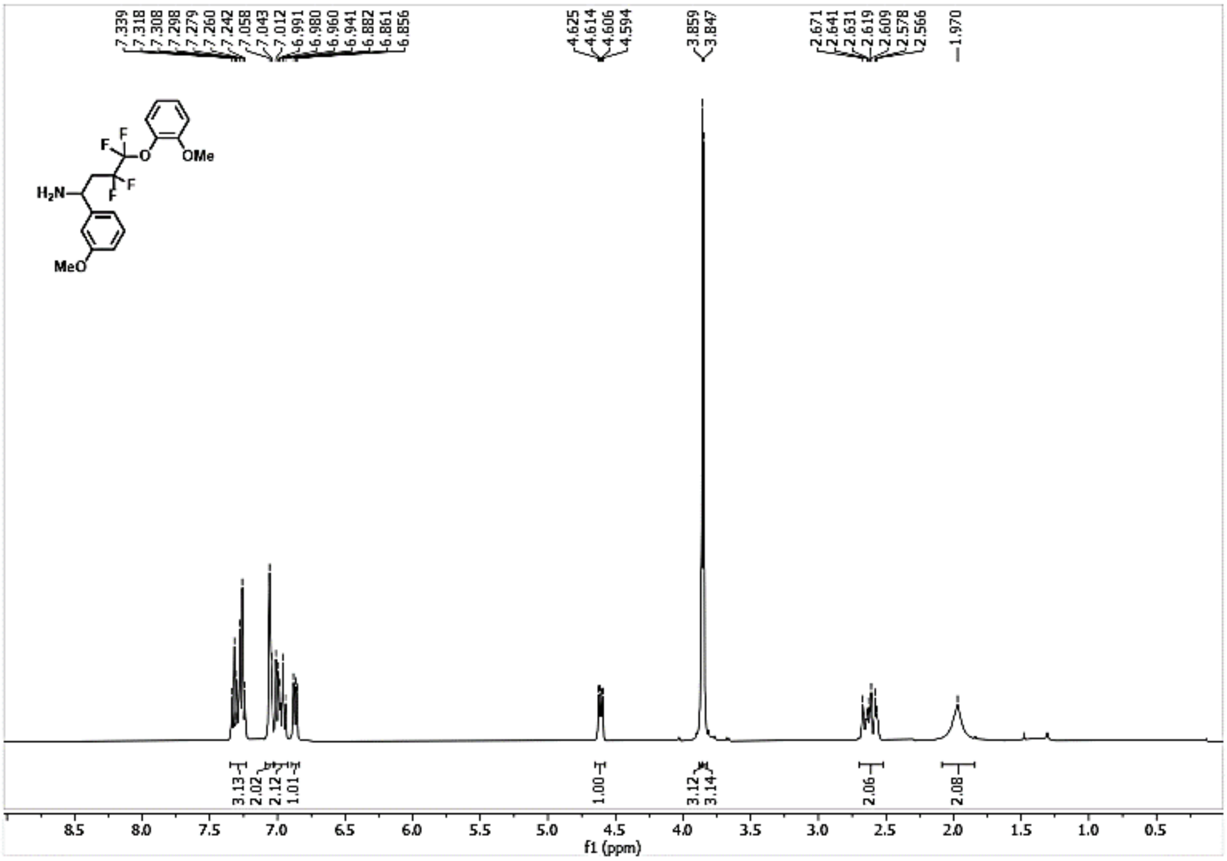

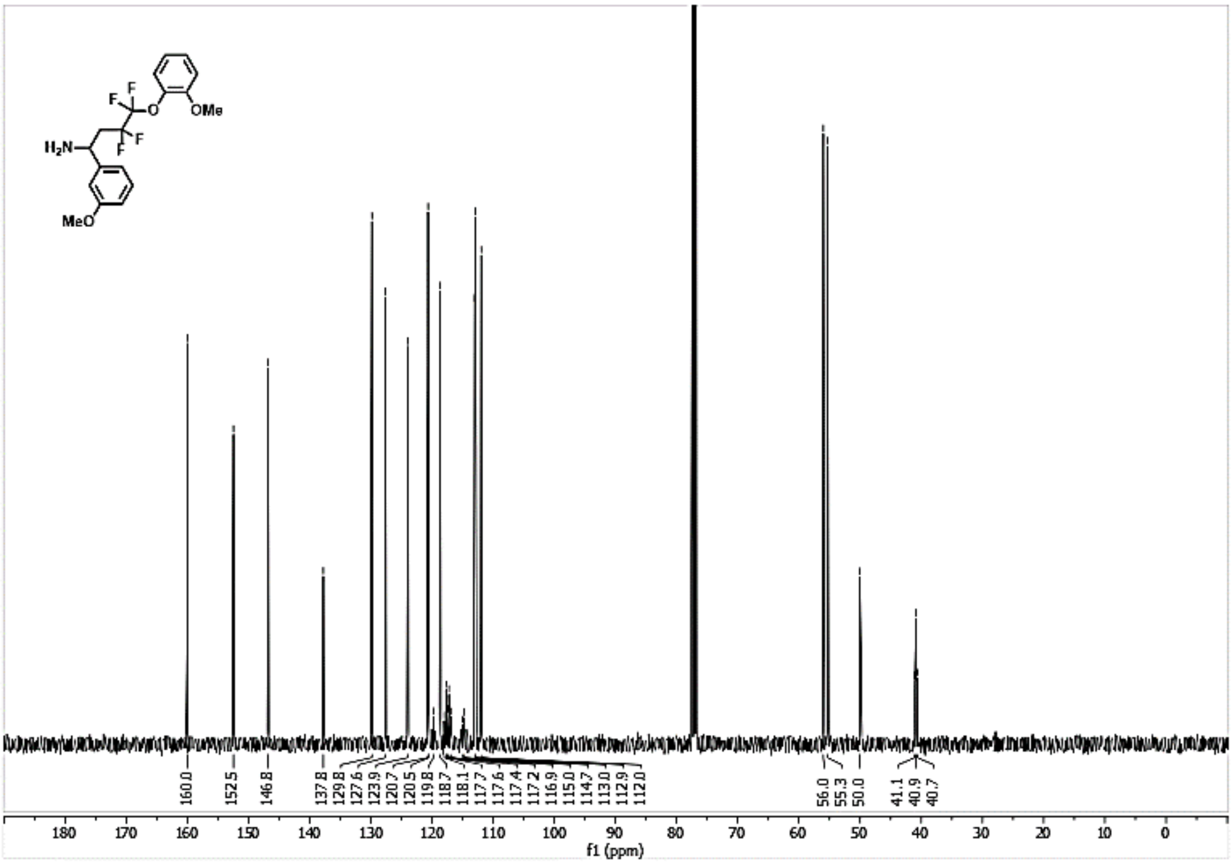

Compound 8. ^{19}F NMR ($CDCl_3$, 376 MHz).

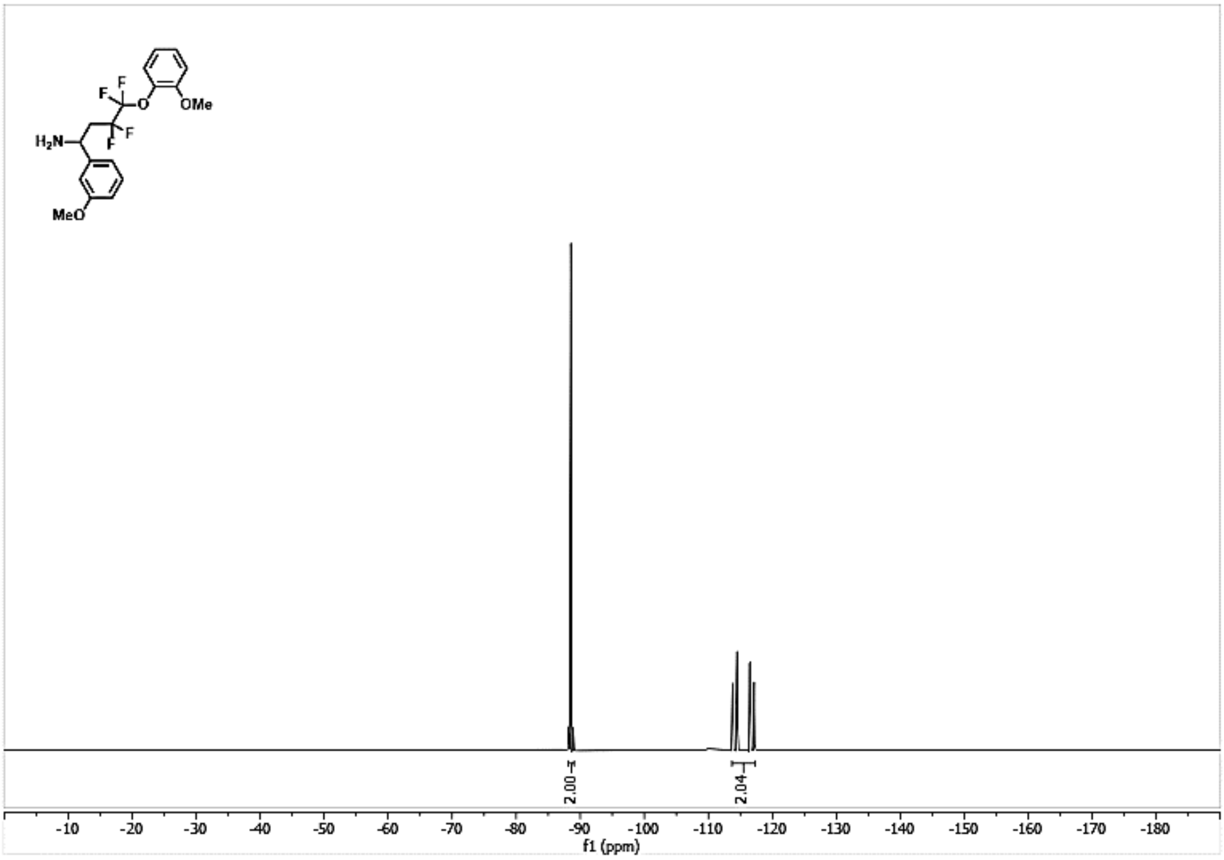

8. Crystallographic data

Compound 7

The single crystal of **7** (**CCDC 2284150**) was prepared by recrystallization in chloroform hexane. ORTEP diagram of **7** with 50% ellipsoid (crystal size 0.12×0.08×0.06 mm^3).

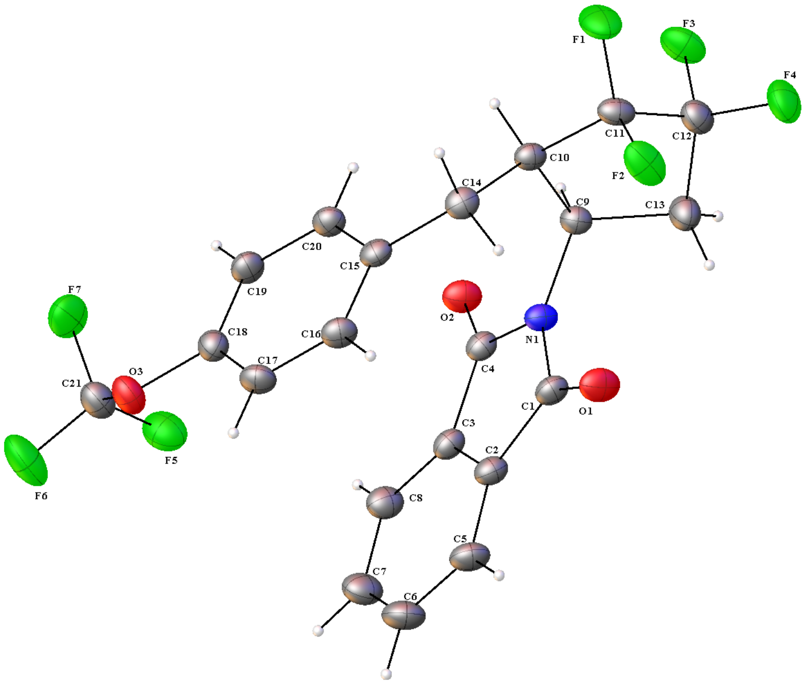

Crystal Data. $C_{21}H_{14}F_7NO_3$, M_r = 461.33, monoclinic, *C*2/*c* (No. 15), a = 22.4393(14) Å, b = 7.6575(5) Å, c = 22.8055(14) Å, β = 106.347(2)°, $\alpha = \gamma$ = 90°, *V* = 3760.2(4) $Å^3$, *T* = 110.0 K, *Z* = 8, *Z'* = 1, $\mu(CuK_\alpha)$ = 1.376, 42921 reflections measured, 3585 unique (R_{int} = 0.0360) which were used in all calculations. The final wR_2 was 0.1147 (all data) and R_1 was 0.0421 (I≥2 σ(I)).

Table S1. Crystal data and structure refinement for **7.**

Formula	$C_{21}H_{14}F_7NO_3$
$D_{calc.}$/ g cm^{-3}	1.630
μ/mm^{-1}	1.376
Formula Weight	461.33
Colour	colourless
Shape	block-shaped
Size/mm^3	0.12×0.08×0.06
T/K	110.0
Crystal System	monoclinic
Space Group	*C*2/*c*
a/Å	22.4393(14)
b/Å	7.6575(5)
c/Å	22.8055(14)
α/°	90
β/°	106.347(2)
γ/°	90
V/Å^3	3760.2(4)
Z	8
Z'	1
Wavelength/Å	1.54178
Radiation type	CuK□
Θ_{min}/°	4.040
Θ_{max}/°	70.195
Measured Refl's.	42921
Indep't Refl's	3585
Refl's I≥2 □(I)	3147
R_{int}	0.0360
Parameters	289
Restraints	0
Largest Peak	0.397
Deepest Hole	-0.262
GooF	1.154
wR_2 (all data)	0.1147
wR_2	0.1064
R_1 (all data)	0.0488
R_1	0.0421

Table S2: Fractional Atomic Coordinates (×10^4) and Equivalent Isotropic Displacement Parameters (Å^2×10^3) for **7**. U_{eq} is defined as 1/3 of the trace of the orthogonalised U_{ij}.

Atom	x	y	z	U_{eq}
F1	5885.0(6)	5611.6(17)	3231.4(5)	34.8(3)
F2	6799.1(5)	5045.8(17)	3835.8(5)	33.1(3)
F3	5412.6(5)	2746.5(17)	3580.5(5)	33.0(3)
F4	6305.7(6)	1961.3(17)	3464.9(5)	35.0(3)
F5	5611.3(6)	9128.7(17)	7058.8(5)	36.3(3)
F006	5025.5(6)	11116.6(18)	6542.3(6)	39.2(3)
F6	5725.9(6)	11793(2)	7366.3(6)	46.0(4)
O1	7367.2(6)	4648(2)	5352.1(6)	27.1(3)
O2	5483.1(6)	4474.8(18)	5761.0(6)	24.1(3)
O3	6031.5(6)	11111.7(18)	6585.2(6)	27.2(3)
N1	6350.2(7)	4463(2)	5400.2(7)	18.5(3)
C1	6988.7(8)	4757(2)	5637.7(8)	20.2(4)
C2	7083.8(8)	5263(2)	6288.2(8)	20.6(4)

Atom	x	y	z	U_{eq}
C3	6512.8(8)	5214(2)	6414.6(8)	19.5(4)
C4	6034.8(8)	4678(2)	5849.9(8)	18.3(4)
C5	7614.8(9)	5820(3)	6718.4(9)	25.6(4)
C6	7552.0(9)	6351(3)	7281.2(9)	30.9(4)
C7	6979.8(10)	6282(3)	7409.8(9)	30.3(4)
C8	6448.4(9)	5700(3)	6976.7(9)	25.1(4)
C9	6015.0(8)	4161(2)	4764.5(8)	18.6(4)
C10	5999.0(8)	5740(2)	4311.7(8)	18.4(4)
C11	6176.0(8)	4901(3)	3777.5(8)	21.6(4)
C12	6038.9(9)	2958(3)	3812.3(8)	24.2(4)
C13	6244.3(9)	2555(3)	4484.7(8)	25.5(4)
C14	6384.1(8)	7366(2)	4547.6(8)	20.5(4)
C15	6260.6(8)	8266(2)	5095.0(8)	18.8(4)
C16	6750.4(8)	9114(2)	5510.5(8)	21.6(4)
C17	6661.2(8)	10025(2)	6005.6(8)	22.9(4)
C18	6075.8(9)	10073(2)	6088.8(8)	21.6(4)
C19	5578.9(8)	9238(3)	5691.3(9)	22.3(4)
C20	5676.7(8)	8337(2)	5194.6(8)	20.8(4)
C21	5602.1(9)	10778(3)	6881.1(9)	28.0(4)

Table S3: Anisotropic Displacement Parameters (×10^4) for **7**. The anisotropic displacement factor exponent takes the form: $-2\pi^2[h^2a^{*2} \times U_{11} + ... +2hka^* \times b^* \times U_{12}]$.

Atom	U_{11}	U_{22}	U_{33}	U_{23}	U_{13}	U_{12}
F1	50.0(8)	35.9(7)	17.0(5)	5.1(5)	6.8(5)	0.8(6)
F2	24.6(6)	45.9(7)	35.2(6)	-11.9(5)	18.9(5)	-10.9(5)
F3	26.7(6)	41.4(7)	29.1(6)	-7.1(5)	4.9(5)	-13.2(5)
F4	45.1(7)	33.6(7)	29.8(6)	-8.7(5)	16.4(5)	2.3(5)
F5	43.1(7)	38.2(7)	27.8(6)	1.8(5)	10.1(5)	-5.2(5)
F006	25.3(6)	48.1(8)	44.9(7)	-7.4(6)	11.1(5)	8.8(5)
F6	47.5(8)	54.0(9)	41.8(7)	-25.9(7)	21.4(6)	-14.9(7)
O1	16.3(6)	43.3(8)	24.2(7)	0.7(6)	9.7(5)	2.0(6)
O2	15.7(6)	33.9(7)	23.7(6)	1.9(6)	7.2(5)	-2.5(5)
O3	25.6(7)	28.3(7)	28.9(7)	-9.7(6)	9.5(5)	-5.1(5)
N1	15.1(7)	25.1(8)	15.9(7)	2.3(6)	5.2(6)	0.3(6)
C1	15.0(8)	23.1(9)	22.2(9)	3.3(7)	4.6(7)	1.8(7)
C2	18.1(8)	24.0(9)	19.6(9)	4.5(7)	5.1(7)	2.5(7)
C3	16.9(8)	21.0(9)	21.6(9)	4.7(7)	7.0(7)	2.8(7)
C4	17.2(8)	19.2(8)	19.5(8)	4.3(7)	6.6(7)	1.5(7)
C5	17.0(9)	34.2(11)	23.0(9)	4.2(8)	1.5(7)	0.0(8)
C6	25.3(10)	40.3(12)	22.4(9)	1.6(9)	-1.0(8)	-1.3(9)
C7	30.1(10)	41.4(12)	18.7(9)	-0.6(8)	5.6(8)	2.1(9)
C8	23.3(9)	32.7(10)	20.7(9)	3.0(8)	8.4(7)	2.2(8)
C9	17.0(8)	21.7(9)	17.2(8)	0.7(7)	4.9(6)	-1.7(7)
C10	15.1(8)	23.0(9)	17.2(8)	1.7(7)	4.6(6)	0.3(7)
C11	17.6(8)	31.6(10)	15.6(8)	1.9(7)	4.7(7)	-2.2(7)
C12	23.8(9)	27.8(10)	22.1(9)	-4.7(8)	8.1(7)	-1.2(7)
C13	32.6(10)	21.3(9)	23.0(9)	0.2(7)	8.2(8)	1.5(8)
C14	18.7(8)	23.1(9)	21.5(9)	4.0(7)	8.8(7)	-1.6(7)
C15	18.1(8)	18.9(8)	18.8(8)	5.3(7)	4.5(7)	0.9(7)
C16	15.0(8)	26.7(9)	23.0(9)	2.5(7)	5.1(7)	-2.2(7)
C17	18.1(9)	26.2(10)	21.9(9)	-0.6(7)	1.7(7)	-4.6(7)
C18	23.2(9)	20.9(9)	20.8(9)	0.3(7)	6.3(7)	1.2(7)
C19	15.8(8)	25.5(9)	25.4(9)	-0.6(7)	5.4(7)	0.6(7)
C20	16.7(8)	22.0(9)	22.6(9)	-0.1(7)	3.5(7)	-1.9(7)
C21	26.2(10)	31.2(11)	27.0(10)	-7.2(8)	8.3(8)	-3.1(8)

Table S4: Bond Lengths in Å for **7**.

Atom	Atom	Length/Å
F1	C11	1.348(2)
F2	C11	1.371(2)
F3	C12	1.365(2)
F4	C12	1.355(2)
F5	C21	1.324(3)
F006	C21	1.332(2)
F6	C21	1.316(2)
O1	C1	1.210(2)
O2	C4	1.207(2)
O3	C18	1.410(2)
O3	C21	1.347(2)
N1	C1	1.400(2)
N1	C4	1.410(2)
N1	C9	1.452(2)
C1	C2	1.490(3)
C2	C3	1.391(2)
C2	C5	1.381(3)
C3	C4	1.483(3)
C3	C8	1.381(3)
C5	C6	1.391(3)
C6	C7	1.396(3)
C7	C8	1.390(3)
C9	C10	1.584(2)
C9	C13	1.539(3)
C10	C11	1.526(2)
C10	C14	1.525(2)
C11	C12	1.526(3)
C12	C13	1.504(3)
C14	C15	1.518(2)
C15	C16	1.394(3)
C15	C20	1.392(2)
C16	C17	1.389(3)
C17	C18	1.380(3)
C18	C19	1.380(3)
C19	C20	1.396(3)

Table S5: Bond Angles in ° for **7**.

Atom	Atom	Atom	Angle/°
C21	O3	C18	121.13(15)
C1	N1	C4	111.63(14)
C1	N1	C9	126.74(14)
C4	N1	C9	121.32(14)
O1	C1	N1	125.30(17)
O1	C1	C2	128.94(17)
N1	C1	C2	105.74(14)
C3	C2	C1	108.49(16)
C5	C2	C1	129.89(17)
C5	C2	C3	121.48(17)
C2	C3	C4	108.11(15)
C8	C3	C2	121.88(17)
C8	C3	C4	129.91(16)
O2	C4	N1	124.34(16)
O2	C4	C3	129.67(16)
N1	C4	C3	105.98(14)
C2	C5	C6	117.06(18)
C5	C6	C7	121.38(18)
C8	C7	C6	121.24(18)
C3	C8	C7	116.93(17)
N1	C9	C10	116.07(14)
N1	C9	C13	113.69(15)
C13	C9	C10	106.78(14)
C11	C10	C9	103.61(14)
C14	C10	C9	119.14(14)
C14	C10	C11	112.14(14)
F1	C11	F2	105.82(14)
F1	C11	C10	113.59(15)
F1	C11	C12	112.94(15)
F2	C11	C10	112.07(14)
F2	C11	C12	106.80(15)
C10	C11	C12	105.53(14)
F3	C12	C11	107.22(15)
F3	C12	C13	110.94(15)
F4	C12	F3	106.44(15)
F4	C12	C11	112.95(15)
F4	C12	C13	114.96(16)
C13	C12	C11	104.16(15)
C12	C13	C9	102.51(15)
C15	C14	C10	116.36(14)
C16	C15	C14	118.62(15)
C20	C15	C14	123.37(16)
C20	C15	C16	117.98(16)
C17	C16	C15	121.35(17)
C18	C17	C16	119.08(17)
C17	C18	O3	114.24(16)
C17	C18	C19	121.46(17)
C19	C18	O3	124.21(17)
C18	C19	C20	118.66(17)
C15	C20	C19	121.46(16)
F5	C21	F006	106.99(16)
F5	C21	O3	112.48(17)
F006	C21	O3	112.78(17)
F6	C21	F5	108.94(17)
F6	C21	F006	108.25(17)
F6	C21	O3	107.29(16)

Table S6: Torsion Angles in ° for **7**.

Atom	Atom	Atom	Atom	Angle/°
F1	C11	C12	F3	-46.65(19)
F1	C11	C12	F4	70.3(2)
F1	C11	C12	C13	-164.30(15)
F2	C11	C12	F3	-162.56(13)
F2	C11	C12	F4	-45.6(2)
F2	C11	C12	C13	79.79(17)
F3	C12	C13	C9	-74.07(18)
F4	C12	C13	C9	165.11(15)
O1	C1	C2	C3	179.60(19)
O1	C1	C2	C5	3.8(3)
O3	C18	C19	C20	176.02(17)
N1	C1	C2	C3	1.3(2)
N1	C1	C2	C5	-174.48(19)
N1	C9	C10	C11	131.90(15)
N1	C9	C10	C14	6.5(2)
N1	C9	C13	C12	-156.95(15)
C1	N1	C4	O2	-179.02(17)
C1	N1	C4	C3	2.1(2)
C1	N1	C9	C10	-65.3(2)
C1	N1	C9	C13	59.1(2)
C1	C2	C3	C4	-0.1(2)
C1	C2	C3	C8	-176.92(17)
C1	C2	C5	C6	174.57(19)
C2	C3	C4	O2	-179.97(19)
C2	C3	C4	N1	-1.20(19)
C2	C3	C8	C7	1.4(3)
C2	C5	C6	C7	1.5(3)
C3	C2	C5	C6	-0.8(3)
C4	N1	C1	O1	179.49(18)
C4	N1	C1	C2	-2.2(2)
C4	N1	C9	C10	107.66(18)
C4	N1	C9	C13	-127.90(17)
C4	C3	C8	C7	-174.71(19)
C5	C2	C3	C4	176.16(17)
C5	C2	C3	C8	-0.7(3)
C5	C6	C7	C8	-0.8(3)
C6	C7	C8	C3	-0.6(3)
C8	C3	C4	O2	-3.5(3)
C8	C3	C4	N1	175.30(19)
C9	N1	C1	O1	-6.9(3)
C9	N1	C1	C2	171.42(16)
C9	N1	C4	O2	7.0(3)
C9	N1	C4	C3	-171.85(15)
C9	C10	C11	F1	145.36(15)
C9	C10	C11	F2	-94.77(17)
C9	C10	C11	C12	21.11(17)
C9	C10	C14	C15	-56.7(2)
C10	C9	C13	C12	-27.64(18)
C10	C11	C12	F3	78.01(17)
C10	C11	C12	F4	-165.05(14)
C10	C11	C12	C13	-39.64(18)
C10	C14	C15	C16	148.16(16)
C10	C14	C15	C20	-34.0(2)
C11	C10	C14	C15	-177.85(15)
C11	C12	C13	C9	40.98(18)
C13	C9	C10	C11	3.98(18)

Atom	Atom	Atom	Atom	Angle/°
C13	C9	C10	C14	-121.40(17)
C14	C10	C11	F1	-84.89(18)
C14	C10	C11	F2	35.0(2)
C14	C10	C11	C12	150.86(15)
C14	C15	C16	C17	177.15(17)
C14	C15	C20	C19	-177.38(17)
C15	C16	C17	C18	0.6(3)
C16	C15	C20	C19	0.5(3)
C16	C17	C18	O3	-176.69(16)
C16	C17	C18	C19	0.0(3)
C17	C18	C19	C20	-0.3(3)
C18	O3	C21	F5	51.9(2)
C18	O3	C21	F006	-69.3(2)
C18	O3	C21	F6	171.64(16)
C18	C19	C20	C15	0.1(3)
C20	C15	C16	C17	-0.8(3)
C21	O3	C18	C17	-152.84(18)
C21	O3	C18	C19	30.6(3)

Table S7: Hydrogen Fractional Atomic Coordinates (×10^4) and Equivalent Isotropic Displacement Parameters (Å^2×10^3) for **7**. U_{eq} is defined as 1/3 of the trace of the orthogonalised U_{ij}.

Atom	x	y	z	U_{eq}
H5	8005.71	5840.57	6633.7	31
H6	7905.93	6768.79	7584.62	37
H7	6953.05	6640.01	7800.42	36
H8	6058.91	5640.3	7063.52	30
H9	5574.16	3917.9	4753.73	22
H10	5556.41	6122.85	4155.23	22
H13A	6701.53	2438.67	4635.4	31
H13B	6048.94	1469.22	4577.29	31
H14A	6828.62	7038.3	4656.86	25
H14B	6311.11	8221.94	4209.25	25
H16	7153.72	9068.38	5453.94	26
H17	6998.6	10606.41	6283.15	27
H19	5178.32	9276.47	5754.58	27
H20	5337.31	7759.33	4918.29	25

9. Current Limitations

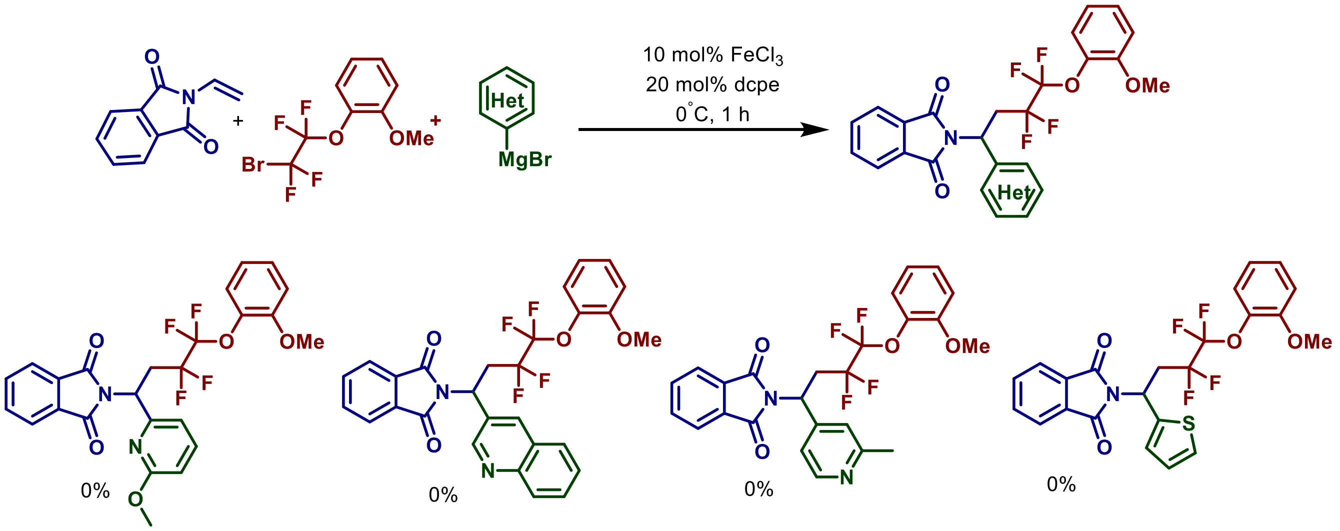

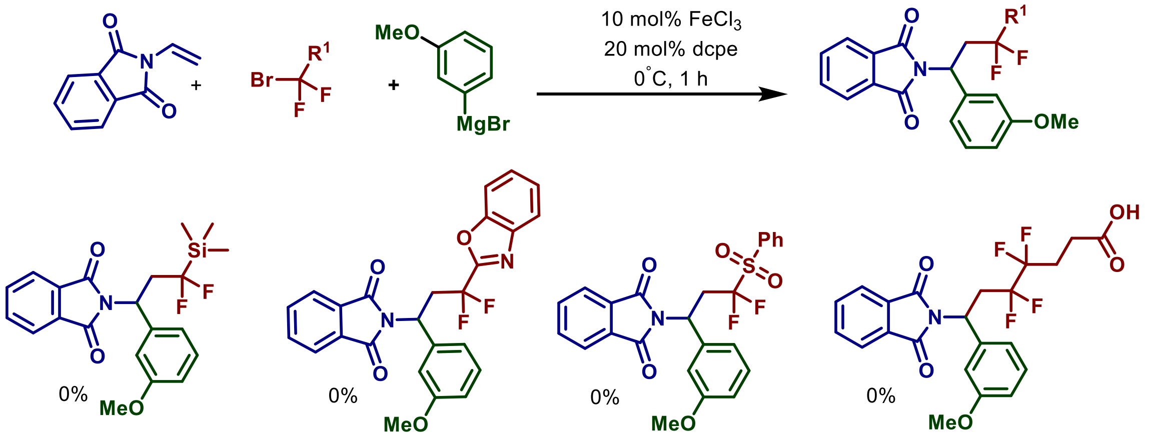

10. References

[1] Nguyen, T. B.; Martel, A.; Dhal, R.; Dujardin, G. 1,3-Dipolar Cycloaddition of *N*-Substituted Dipolarophiles and Nitrones: Highly Efficient Solvent-Free Reaction. *J. Org. Chem.* **2008**, *73*, 2621-2632.

[2] Mondal, K.; Patra, S.; Halder, P.; Mukhopadhyay, N.; Das, P. CuF_2/DMAP-Catalyzed *N*-Vinylation: Scope and Mechanistic Study. *Org.Lett.* **2023**, *25*, 1235−1240.
